# CRISPR/Cas9-mediated inactivation of *miR-34a* and *miR-34b/c* in HCT116 colorectal cancer cells: comprehensive characterization after exposure to 5-FU reveals EMT and autophagy as key processes regulated by miR-34

**DOI:** 10.1038/s41418-023-01193-2

**Published:** 2023-07-24

**Authors:** Zekai Huang, Markus Kaller, Heiko Hermeking

**Affiliations:** 1 Experimental and Molecular Pathology, Institute of Pathology, Faculty of Medicine, Ludwig-Maximilians-Universität München, Thalkirchner Str. 36, D-80337 Munich, Germany; 2 German Cancer Consortium (DKTK), Partner Site Munich, D-80336 Munich, Germany; 3 German Cancer Research Center (DKFZ), D-69120 Heidelberg, Germany

**Keywords:** Cancer genetics, Experimental models of disease

## Abstract

The *miR-34a* and *miR-34b/c* encoding genes represent direct targets of the p53 transcription factor, and presumably mediate part of the tumor suppressive effects of p53. Here, we sought to determine their functional relevance by inactivating *miR-34a* and/or *miR-34b/c* using a CRISPR/Cas9 approach in the colorectal cancer (CRC) cell line HCT116. Concomitant deletion of *miR-34a* and *miR-34b/c* resulted in significantly reduced suppression of proliferation after p53 activation, enhanced migration, invasion and EMT, as well as reduced sensitivity to chemotherapeutics, increased stress-induced autophagic flux, decreased apoptosis and upregulation of autophagy-related genes after 5-FU treatment. However, inactivation of singular *miR-34a* or *miR-34b/c* had little effects on the aforementioned processes. RNA-Seq analysis revealed that concomitant deletion of *miR-34a/b/c* caused EMT signature enrichment, impaired gene repression by the p53-DREAM pathway and elevated autophagy after 5-FU treatment. A gene signature comprised of mRNAs significantly upregulated after combined inactivation of *miR-34a* and *miR-34b/c* showed a significant association with the invasive colon cancer subtype CMS4 and poor overall survival in two CRC patient cohorts, and with 5-FU resistance in CRC cell lines. In *miR-34a/b/c-*deficient cells the upregulated miR-34 target *FOXM1* directly induced *p62* and *ATG9A*, which increased autophagy and consequently attenuated apoptosis and rendered the *miR-34a/b/c-KO* cells more resistant to 5-FU. Inhibition of autophagy by depletion of ATG9A or chloroquine re-sensitized *miR-34a/b/c*-deficient HCT116 cells to 5-FU. In summary, our findings show a complementary role of *miR-34a* and *miR-34b/c* in the regulation of EMT and autophagy which may be relevant for CRC therapy in the future.

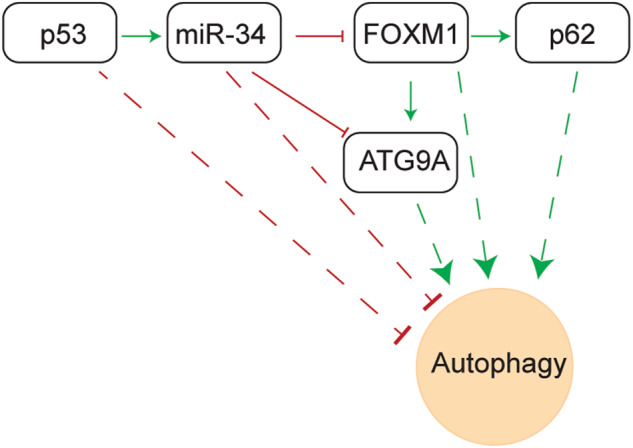

## Introduction

Colorectal cancer (CRC) represents the third most commonly diagnosed cancer type and the second leading cause of cancer death worldwide [1]. While surgery is curative and the first choice for early-stage CRC patients, an estimated 50% to 60% of CRC patients develop colorectal metastases [2], with 80% to 90% of these representing unresectable liver metastases [3]. Fluorouracil (5-FU)-based chemotherapy remains the standard of care for patients with metastatic colorectal cancer (mCRC). However, the overall response rate to 5-FU in mCRC patients is limited to about 50% [4] and resistance to 5-FU inevitably develops [5]. CRC is a complex and heterogeneous disease manifested by distinct epi-genetic and genetic characteristics. Patients with different biological CRC subtypes display a large variation in prognosis and therapy response [6]. Although promising, check-point inhibitor-based or targeted therapy only benefit a fraction of CRC patients [7, 8]. Therefore, there is an urgent need for more profound insights into the molecular mechanisms and gene regulations underlying the response of CRC cells to therapeutic drugs.

*MiR-34a* and *miR-34b/c* encoding genes represent direct targets of p53 [9], a tumor suppressive protein that is activated by cellular stresses including DNA damage, oncogene activation and hypoxia [10]. P53 presumably mediates its tumor suppression functions partially through *miR-34a/b/c* [11]. *MiR-34a* and *miR-34b/c* genes are frequently inactivated epigenetically by CpG methylation in CRC [12, 13]. Importantly, combined deletion of *Mir34a* and *Mir34b/c* promotes intestinal tumorigenesis and decreases survival in *Apc*^Min/+^ mice that inherit a mutant *Apc* (adenomatous polyposis coli) allele [14]. Furthermore, combined inactivation of *Mir34a* and *p53* promotes colorectal cancer development and progression in mice [15]. In addition, *miR-34a* suppresses EMT-mediated colorectal cancer invasion and metastasis by inhibiting an *IL6R*/*STAT3*/*miR-34a* feedback loop [16], and *miR-34a* silencing by DNA-methylation is significantly associated with increased lymph node and liver metastasis in CRC [17]. These findings indicate that *miR-34a/b/c* have tumor suppressive functions in CRC.

Here, we showed that concomitant inactivation of *miR-34a* and *miR-34b/c* in the CRC cell line HCT116 using a CRISPR/Cas9 approach significantly enhanced migration, invasion and EMT, reduced sensitivity to chemotherapeutics and increased stress-induced autophagic flux. RNA-Seq analysis revealed that combined deletion of *miR-34a/b/c* significantly induced the expression of EMT- and macroautophagy/autophagy-related mRNAs and impaired repression mediated by the DREAM complex. MiR-34a/b/c inhibited autophagy by directly repressing *FOXM1* and *ATG9A*. The downregulation of *FOXM1* subsequently repressed *p62* and *ATG9A*, as these represent FOXM1 target genes. In addition, inhibition of autophagy re-sensitized *miR-34a/b/c*-deficient CRC cells to 5-FU.

## Results

### Generation and characterization of *miR-34a/b/c*-deficient HCT116 cell lines

**Fig. 1:**
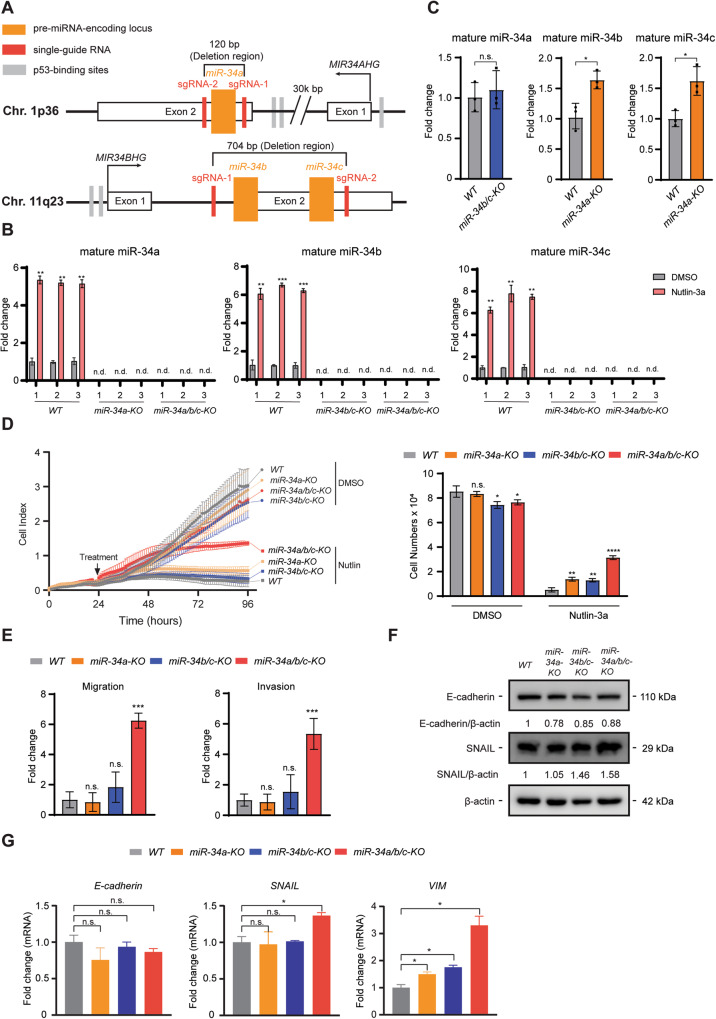
Deletion of *miR-34a/b/c* increases cell proliferation, migration and invasion, and promotes EMT in HCT116 cells. **A** Schematic illustrations of *miR-34a* and *miR-34b/c* genomic location and deletion of the mature miRNA coding regions using a CRISPR-Cas9 approach. MiRNA encoding loci are indicated as orange columns. Single-guide RNAs (sgRNAs) targeted regions are shown as red columns. Gray columns indicate p53-binding sites. **B** qPCR analysis of mature miR-34 expression after addition of DMSO or 10 μM of Nutlin-3a for 48 h. **C** qPCR analysis of mature miR-34 expression in *miR-34a-KO* and *miR-34b/c-KO* cells. **D** Proliferation of *miR-34*-deficient HCT116 cells was determined by real-time cellular impedance assay using an xCELLigence system. Cell numbers were determined at the end point. **E** Quantification of cellular migration or invasion using Boyden-chamber assays. **F** Western blot analysis and **G** qPCR analysis of EMT markers. Results are presented as the mean ± SD (*n* = 3) for **B–E** and **G** with *p < 0.05, **p < 0.01, ***p < 0.001, ****p < 0.0001, n.s. no significance.

In order to characterize the functions of the three *p53*-inducible miR-34 family members, the regions encoding the mature *miR-34a* and *miR-34b/c* within their host genes were deleted alone or in combination in the CRC cell line HCT116 cell using a CRISPR-Cas9 approach. Single-guide RNAs (sgRNAs) targeting the sequence regions flanking the genomic regions of precursor *miR-34a* or *miR-34b/c* are shown in Fig. 1A and Table S1. Three independent single cell derived clones for each genotype (i.e., *miR-34a-KO*, *miR-34b/c-KO* and *miR-34a/b/c-KO*) were obtained. As controls, three independent single cell derived clones for *wild-type* cells (*WT*) were also generated by transfecting HCT116 cells with pSpCas9 plasmids not harboring sgRNAs. The deletion of the *miR-34a* and/or *miR-34b/c* loci was confirmed by PCR (Fig. S1A–D and Table S2). In addition, the lack of *miR-34a* and/or *miR-34b/c* expression was confirmed by qPCR analysis after p53 activation by addition of Nutlin-3a, a highly selective MDM2 inhibitor [18] (Fig. 1B). Interestingly, mature miR-34b and miR-34c showed a significantly elevated expression after *miR-34a* deletion in HCT116 cells (Fig. 1C), suggesting that the loss of miR-34a functions may be partially compensated by upregulation of *miR-34b/c*. Deletion of *miR-34b/c* in HCT116 cells resulted in a marginal increase of mature miR-34a expression (Fig. 1C), presumably since the basal expression levels of miR-34b and miR-34c are relatively low in HCT116 cells when compared to miR-34a [19].

### *miR-34a/b/c* inhibits cellular proliferation in HCT116 cells

Next, we analyzed whether loss of miR-34a/b/c affects cellular proliferation by real-time impedance measurement. Deletion of *miR-34a/b/c* in HCT116 cells resulted in minor effects on proliferation when compared to *WT* cells, which however were not consistently statistically significant (Fig. 1D and Fig. S2A). However, when *p53* was activated by addition of Nutlin-3a, *miR-34*-deficient cells were partially refractory to Nutlin-3a when compared to *WT* cells, with *miR-34a/b/c-KO* cells displaying the highest rate of proliferation in the presence of Nutlin-3a (Fig. 1D). These results demonstrate that a substantial portion of p53-mediated repression of proliferation is mediated by the combined action of *miR-34a/b/c*. They also imply that inactivation of a single *miR-34* isoform is not sufficient to alleviate inhibition of proliferation by p53.

### Loss of *miR-34a/b/c* promotes EMT, migration and invasion in HCT116 cells

Next, we asked whether *miR-34*-deficiency affects migration and invasion of HCT116 cells. Indeed, *miR-34a/b/c-*deficient cells demonstrated significantly increased migration and invasion when compared to *WT* cells in Boyden-chamber assays (Fig. 1E and Fig. S2B). Increased migration of *miR-34a/b/c-KO* cells was further confirmed by a wound healing assay with or without treatment of Nutlin-3a (Fig. S2C, D). Since epithelial-mesenchymal transition (EMT) is an important mechanism underlying migration and invasion, EMT markers were tested to determine whether *miR-34*-deficiency affects EMT. Protein levels of E-cadherin, an epithelial marker, were decreased in *miR-34*-deficient cells when compared to *WT* cells, whereas the expression of SNAIL, a mesenchymal marker, was elevated in all *miR-34*-deficient cells (Fig. 1F). qPCR analysis indicated a significantly higher expression of *SNAIL* and *VIM* in *miR-34a/b/c-KO* cells when compared to *WT* cells, but for *SNAIL* not in *miR-34a-KO* or *miR-34b/c-KO* cells (Fig. 1G). In addition, mRNA expression of *E-cadherin* was downregulated in all *miR-34*-deficent cells, albeit not significantly (Fig. 1G). Therefore, the changes in EMT-related gene expression may at least in part explain the enhanced migration and invasion of *miR-34a/b/c-KO* cells.

### Loss of *miR-34a/b/c* mediates resistance to chemotherapeutic agents by enhancing autophagic flux

**Fig. 2:**
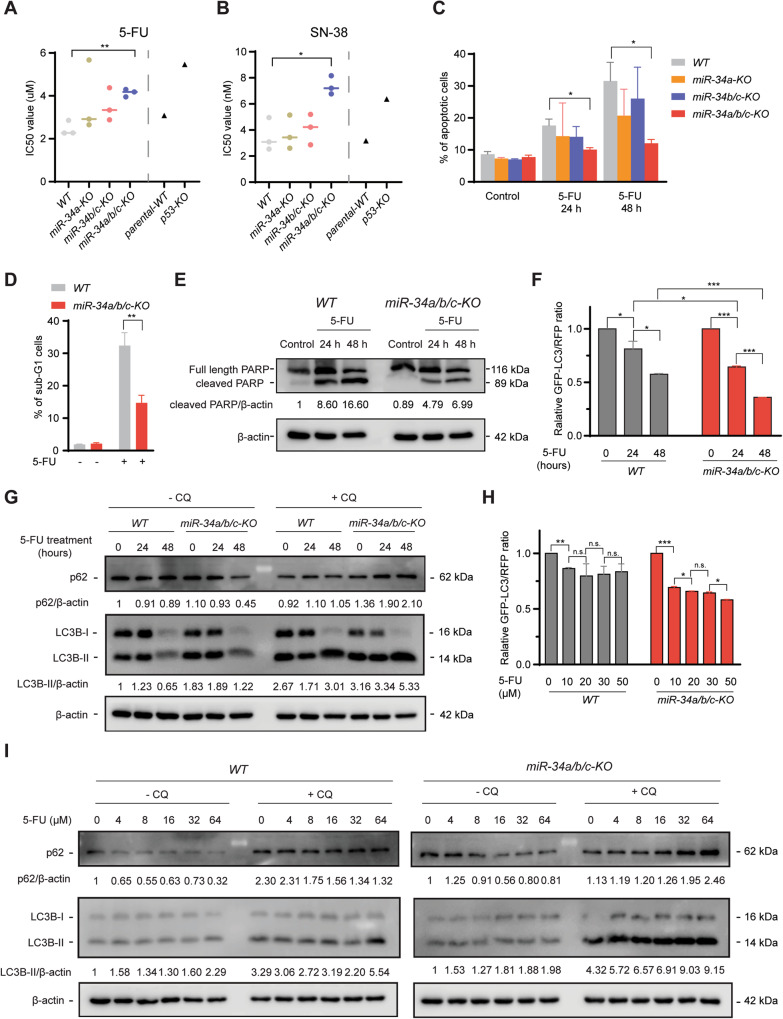
Loss of *miR-34a/b/c* de-sensitizes HCT116 cells to chemotherapeutic agents by enhancing autophagic flux. **A, B** IC50 value determination of HCT116 cells with different *miR-34* or *p53* genotypes in response to 5-FU or SN-38. The corresponding representative dose-response curves of which the IC50 values were calculated are shown in Fig. S3A, B. Cells were treated with a wide range of concentrations of the indicated therapeutic drugs for 72 h and then subjected to CCK-8 assay and IC50 value determination. **C** Cells were treated with 5-FU for 24 or 48 h and then apoptosis rates were determined by FITC Annexin V staining and FACS analysis. **D** Quantification of sub-G1 cell population by FACS analysis after treatment with DMSO or 5-FU for 48 h using PI staining. **E** Western blot analysis of cleaved-PARP after treatment with DMSO or 5-FU for the indicated periods. **F** Quantification of GFP-LC3/RFP ratio by FACS analysis of cells stably expressing GFP-LC3-RFP treated with DMSO or 5-FU. Lower GFP-LC3/RFP signal ratio due to increased GFP-LC3 degradation indicates higher autophagic flux. **G** *WT* and *miR-34a/b/c-KO* cells were treated with DMSO or 5-FU for the indicated durations and analyzed by immunoblotting. 20 μM of CQ (chloroquine) was added for 4 h before harvesting cells. **H** Quantification of GFP-LC3/RFP ratio by FACS analysis of cells stably expressing GFP-LC3-RFP probe treated with DMSO or 5-FU for the indicated concentrations. **I** Cells were treated with DMSO or 5-FU at the indicated concentrations. 20 μM of CQ (chloroquine) was added for 4 h before harvesting cells. Results are presented as the mean ± SD (n = 3) for **A**–**D** and **F** + **H** with *p < 0.05, **p < 0.01, ***p < 0.001, n.s. no significance.

Next, we analyzed whether loss of miR-34 function affects the cellular response to chemotherapeutic agents. 5-FU and SN-38 (the active metabolite of Irinotecan) are widely used chemotherapeutics for treatment of CRC. *miR-34-*deficient and *WT* HCT116 cells were treated with 5-FU or SN-38 at a wide range of concentrations, and then subjected to cell viability analysis and IC50 value determination. Intriguingly, only *miR-34a/b/c-KO* cells displayed a significant, ca. two-fold increase in IC50 values for both 5-FU and SN-38, whereas singular deletion of *miR-34a* or *miR-34b/c* resulted in a minor increase (Fig. 2A, B and Fig. S3A, B). *P53-KO* cells displayed a ca. two-fold increase in IC50 values for both 5-FU and SN-38 when compared to *p53-WT* cells (Fig. 2A, B and Fig. S3A, B), indicating that only the concomitant loss of *miR-34a* and *miR-34b/c* expression has a similar effect as *p53*-deficiency on the response to chemotherapeutics. As an alternative approach for testing sensitivity towards chemotherapeutic agents, apoptosis was evaluated by detection of Annexin V positive cells using flow cytometry. Only *miR-34a/b/c-KO* cells displayed a significant reduction in apoptosis when exposed to 5-FU when compared to *WT* cells (Fig. 2C and Fig. S3C), suggesting that inactivation of both *miR-34a* and *miR-34b/c* is required for increased chemo-resistance. The resistance of *miR-34a/b/c-KO* cells to 5-FU was further corroborated by DNA content analysis using flow cytometry, which showed a decrease of cells in the sub-G_1_ phase in *miR-34a/b/c-KO* cells after exposure to 5-FU for 48 h when compared to *WT* cells (Fig. 2D and Fig. S3D). In addition, *miR-34a/b/c-KO* cells showed significantly reduced cleavage of PARP, an apoptosis marker, after treatment with 5-FU when compared to *WT* cells as determined by immunoblotting analysis (Fig. 2E). Taken together, these results indicated the loss of *miR-34a/b/c* rendered HCT116 cells chemo-resistant by decreasing apoptosis.

Macroautophagy/autophagy, a process in which organelles termed autophagosomes deliver intracellular components to the lysosome for degradation, has been implicated in inhibiting apoptosis and thereby conferring therapy resistance [20]. Autophagy degrades and reduces the abundance of damaged mitochondria and pro-apoptotic proteins (e.g., active caspase 8) to attenuate apoptosis and promote cellular adaptation and survival [21]. Therefore, we asked whether increased autophagy contributes to the chemo-resistance observed in *miR-34a/b/c-KO* cells. To measure autophagic flux, a measure of autophagic degradation activity, we generated cells stably expressing GFP-LC3-RFP, an established fluorescent autophagic flux probe [22], and used them to estimate autophagy activity by calculating the GFP-LC3/RFP signal ratio. This probe is cleaved into equimolar amounts of GFP-LC3 and RFP by the ATG4 protease and subsequently GFP-LC3 is incorporated into autophagosomes and degraded, while RFP is not degraded by autophagy and remains in the cytosol, thereby serving as an internal control. Consequently, a lower GFP-LC3/RFP signal ratio represents a higher degree of autophagic flux. An example is shown in Fig. S4A, B, where GFP-LC3 signal intensity decreased after 5-FU treatment while RFP intensity remained unchanged. When *WT* and *miR-34a/b/c-KO* cells stably expressing a GFP-LC3-RFP probe were treated with 5-FU for 24 or 48 h, it caused a significantly higher level of autophagic flux in *miR-34a/b/c-KO* cells, since a significantly larger decrease in GFP-LC3/RFP ratio was detected in *miR-34a/b/c-KO* cells when compared to *WT* cells (Fig. 2F). As an alternative approach to measure autophagic flux, an immunoblotting analysis was performed to measure the lysosomal turnover of LC3-II, a widely used autophagosome marker, and SQSTM1/p62, a receptor protein for autophagy, in the presence and absence of chloroquine (CQ), a lysosomal inhibitor [23]. In agreement with the results obtained with the GFP-LC3-RFP probe, after treatment of 5-FU for 24 or 48 h, *miR-34a/b/c-KO* cells displayed a significantly increased turnover of endogenous LC3-II and p62 when compared to *WT* cells (Fig. 2G), indicating that *miR-34a/b/c-KO* cells display a higher level of autophagic flux after exposure to 5-FU. In addition, *miR-34a/b/c-KO* cells consistently displayed an enhanced autophagic flux in response to 5-FU in a dose-dependent manner, whereas *WT* cells showed less autophagy (Fig. 2H, I). Also higher concentration of 5-FU did not consistently result in increased autophagic flux in *WT* cells. Collectively, these results show that autophagy is significantly induced in *miR-34a/b/c-KO* cells after treatment with 5-FU when compared to *WT* cells. Therefore, autophagy is presumably responsible for the chemo-resistance of *miR-34a/b/c-KO* cells in response to 5-FU.

### Loss of *miR-34a/b/c* consistently elevates autophagic flux after stress

**Fig. 3:**
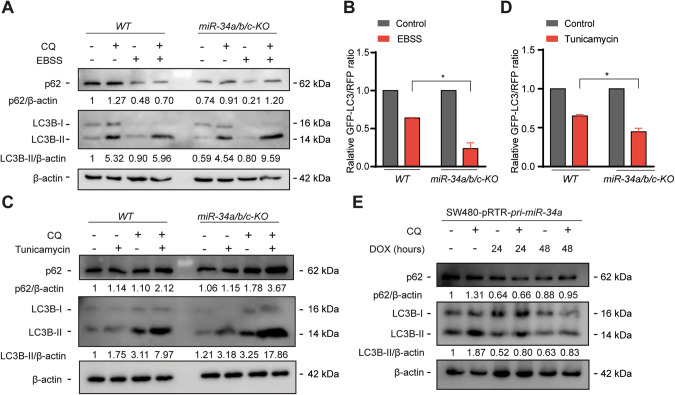
*miR-34a/b/c*-deficiency increases EBSS- and Tunicamycin-induced autophagic flux, while ectopic expression of *miR-34a* inhibits autophagy. **A** Cells were incubated in complete medium or EBSS for 24 h. 20 μM of chloroquine was added for the last 4 h before Western blot analyses of the indicated proteins. **B** Cells stably expressing GFP-LC3-RFP were incubated in complete medium or EBSS for 24 h and then subjected to FACS analysis. **C** *WT* and *miR-34a/b/c-KO* cells were treated with DMSO or Tunicamycin for 24 h. 20 μM of chloroquine was added for the last 4 h before Western blot analyses of the indicated proteins. **D** FACS analysis of cells stably expressing GFP-LC3-RFP treated with DMSO or Tunicamycin for 24 h. **E** Doxycycline was added as indicated to induce ectopic expression of *miR-34a* in SW480 cells. 20 μM of chloroquine was added for the last 4 h before Western blot analyses of the indicated proteins. Results are presented as the mean ± SD (n = 3) for **B** and **D** with *p < 0.05.

To investigate whether *miR-34a/b/c-KO* cells also display a higher autophagic flux when autophagy is induced by alternative means, cells were subjected to starvation of amino acids and serum by cultivation in Earle’s Balanced Salt Solution (EBSS), as well as to treatment of Tunicamycin, which induces ER stress. When cultured in EBSS, *miR-34a/b/c-KO* cells displayed significantly higher levels of autophagic flux when compared to *WT* cells, as indicated by the enhanced LC3-II and p62 turnover (Fig. 3A), as well as by the enhanced degradation of GFP-LC3 as shown by FACS analysis (Fig. 3B). Likewise, Tunicamycin treatment also induced significantly higher autophagic flux in *miR-34a/b/c-KO* cells (Fig. 3C, D). Unexpectedly, accumulation of p62 protein was observed (Fig. 3C). A possible explanation could be a transcriptional activation of p62, which we observed (Fig. S5A). This effect could be mediated by activation of NRF2, a transcription factor that is activated by tunicamycin and is known to regulate p62 [24, 25]. In addition, ectopic expression of *pri-miR-34a* from an episomal pRTR vector repressed basal autophagy in SW480 (Fig. 3E) and HCT15 cells (Fig. S5B). Since 5-FU also resulted in a significantly elevated autophagic flux in HCT116 *p53-KO* cells when compared to *WT* cells (Fig. S5C), the inactivation of miR-34a/b/c at least partially recapitulated the effects of p53 loss on autophagy. Taken together, these results confirmed that *miR-34a* and *miR-34b/c* negatively regulate autophagy in HCT116 cells.

### Analysis of mediators of miR-34 function by expression profiling

**Fig. 4:**
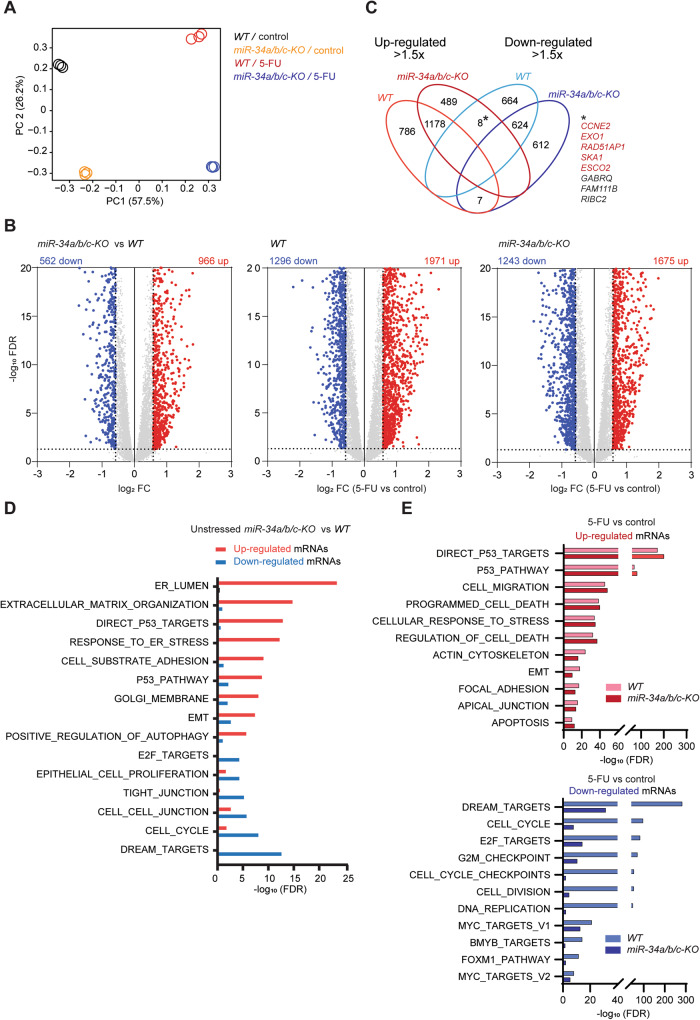
Comprehensive gene expression analysis of *miR-34a/b/c*-deficient cells after treatment with 5-FU. **A** Principal component analysis of mRNAs expression in *WT* and *miR-34a/b/c-KO* HCT116 cells treated with DMSO or 5-FU for 48 h. **B** Volcano plots showing differential RNA expression with FDR in −log_10_ scale and fold change in log_2_ scale. Significantly up- and downregulated genes (FDR < 0.05 & absolute fold change > 1.5) are highlighted in red and blue respectively as indicated. Non-significantly regulated genes are shown in gray. **C** Venn diagram displaying the number of differentially expressed mRNAs shared between *WT* and *miR-34a/b/c-KO* cells (5-FU vs control). **D** Overrepresentation analysis (ORA) of mRNAs significantly regulated in untreated *miR-34a/b/c-KO* vs. wild-type cells. **E** Overrepresentation analysis (ORA) of mRNAs significantly regulated in 5-FU-treated *miR-34a/b/c-KO* and wild-type cells.

To comprehensively identify mediators of *miR-34a* and *miR-34b/c* function by which these microRNAs regulate the aforementioned and other processes, we determined the mRNA expression profiles of HCT116 *miR-34a/b/c-KO* cells and corresponding *WT* cells after exposure to 5-FU (4 μM) for 48 h by RNA-Seq analysis. Libraries were generated from RNAs isolated from three biological replicates of 4 states: *WT* and *miR-34a/b/c-KO* HCT116 cells treated with 5-FU or, as a control, treated with DMSO. RNA-Seq analysis was performed with more than 30 million paired-end reads per library. Principal component analysis (PCA) revealed that the majority of variations between 5-FU-treated and untreated cells was captured by principal component (PC) 1 in both *WT* and *miR-34a/b/c-KO* HCT116 cells, while loss of *miR-34a/b/c* resulted in significantly altered expression profiles predominantly captured by PC2 (Fig. 4A). Differential RNA expression analysis was performed using DESeq2 and differentially expressed mRNAs (FDR < 0.05 & absolute fold change > 1.5) in unstressed cells or after 5-FU treatment are displayed in volcano plots (Fig. 4B) as well as summarized in Tables S7–9. Deletion of *miR-34a/b/c* in unstressed HCT116 cells resulted in a significant upregulation of 966 mRNAs and downregulation of 562 mRNAs when compared to *WT* cells (left panel, Fig. 4B and Table S7). In addition, treatment of 5-FU significantly upregulated 1971 mRNAs and downregulated 1296 mRNAs in *WT* cells when compared to DMSO control (middle panel, Fig. 4B and Table S8). Treatment of 5-FU significantly upregulated 1675 mRNAs and downregulated 1243 mRNAs in *miR-34a/b/c-KO* cells when compared to DMSO control (right panel, Fig. 4B and Table S9). Interestingly, the overlap between mRNAs either up- or downregulated (>1.5× fold change) in *miR-34a/b/c-KO* or wild-type cells after treatment with 5-FU was not complete (Fig. 4C). This suggested that the response to 5-FU treatment, while sharing substantial commonalities that were already indicated by PCA, showed differences that were dependent on *miR-34a/b/c*. We observed limited overlap between genes showing strong opposing regulation (>1.5x fold change up- or downregulation) (Fig. 4C). Interestingly, among the 8 mRNAs that were downregulated in *WT* cells and up-regulated in *miR-34a/b/c-KO* cells, 5 (*CCNE2, RAD51AP1, SKA1, ESCO2, EXO1*) were related to cell proliferation–related pathways such as DNA replication and repair (*EXO1, RAD51AP1*), mitosis (*ESCO2, SKA1*), and cell cycle regulation (*CCNE2*), suggesting that deletion of *miR-34a/b/c* may affect cell cycle progression after treatment with 5-FU.

Next, we employed pathway over-representation analysis (ORA) using gene sets from the MSigDB database [26] in order to identify molecular and cellular pathways, showing differential basal expression of their components in untreated *miR-34a/b/c-KO* vs. wild-type cells (Fig. 4D). The pathways enriched among the mRNAs upregulated in *miR-34a/b/c-KO* cells were represented by gene sets comprising genes involved in endoplasmic reticulum (ER) and Golgi organization, ER stress, positive regulation of autophagy, as well as extracellular matrix, EMT, and p53 pathway activation. Conversely, gene sets over-represented among the downregulated mRNAs included gene sets representing epithelial cell organization (TIGHT_JUNCTION, CELL_CELL_JUNCTION), as well as gene sets related to cell proliferation, such as E2F_TARGETS, DREAM_TARGETS, and CELL CYCLE. These results largely corroborated our initial observations that *miR-34a/b/c-KO* cells display a more mesenchymal phenotype, as well as increased autophagy.

Furthermore, we employed ORA to analyze which pathways were significantly altered after 5-FU treatment of *miR-34a/b/c-KO* and wild-type cells (Fig. 4E). Pathway over-representation among the upregulated mRNAs was similar between *miR-34a/b/c-KO* and wild-type cells, which included p53 activation, apoptosis, as well as EMT and additional gene sets representing pathways related to cell migration. Interestingly, we observed profound differences in pathway over-representation among the downregulated mRNAs between *miR-34a/b/c-KO* and wild-type cells after 5-FU treatment. Notably, the downregulation of cell proliferation associated pathways, though still significant, was severely diminished in *miR-34a/b/c-KO* cells. As noted before (Fig. 4C), these results strongly indicated that deletion of *miR-34a/b/c* may abrogate the cell cycle arrest observed after 5-FU treatment in *WT* cells.

**Fig. 5:**
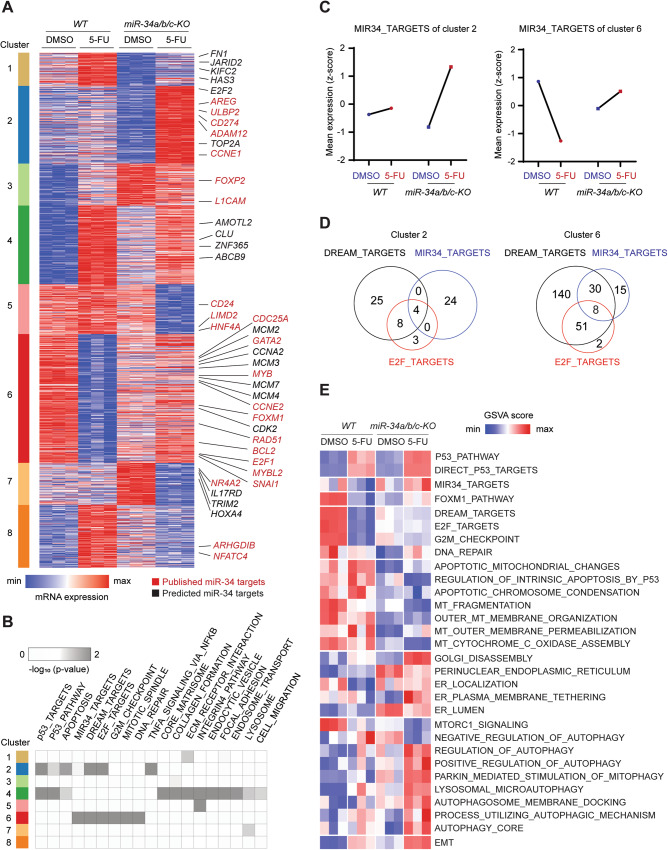
Comprehensive identification of differentially regulated miR-34 targets and their association with functional categories. **A** Heatmap of RNA expression of mRNAs with statistically significant, genotype-dependent differences (>1.5-fold) in regulation after 5-FU treatment grouped in the indicated transcriptional clusters. Selected miR-34 targets are indicated by color. Red: published; black: predicted. **B** Heatmap showing enrichment of the indicated pathways and functional categories in the expression clusters as shown in (**A**). Statistical significance was calculated by Fisher’s exact test. **C** Line plot representation of mean z-score of normalized RNA expression of all miR-34 targets with statistically significant, genotype-dependent differences in regulation after 5-FU treatment, grouped in the indicated transcriptional clusters. **D** Venn diagram displaying the number of shared DREAM, E2F and miR-34 targets in the indicated transcriptional clusters. **E** Heatmap of GSVA analysis of the indicated pathways activities changes caused by *miR-34a/b/c* inactivation after 5-FU treatment.

Next, we determined how the differential gene expression caused by the loss of *miR-34a/b/c* may cause the aforementioned alterations observed for specific cellular processes. Therefore, we first determined the set of mRNAs showing genotype-dependent differences (>1.5-fold) in regulation after 5-FU treatment. We performed K-means clustering with the resulting set of 1691 genes showing differential regulation (Fig. 5A and Table S10) and identified the published and predicted miR-34 targets in each cluster (Table S10). Individual predicted or published miR-34a targets did not follow a specific pattern of differential regulation between *miR-34a/b/c-KO* and wild-type cells and could be found in all of the expression clusters. However, we noted that miR-34 targets were strongly over-represented in cluster 6 (and to a lesser extent in cluster 2), which largely comprised genes involved in cell-cycle regulation (Fig. 5B). These mRNAs were characterized by strongly elevated upregulation in *miR-34a/b/c-KO* cells or severely diminished repression in *miR-34a/b/c-KO* cells (Fig. 5C). Although miR-34 targets did not show a uniform type of expression change after treatment with 5-FU, in sum they displayed either activation or de-repression in *miR-34a/b/c-KO* cells. Interestingly, among the 266 DREAM targets found in clusters 2 and 6, 42 (15.8%) were predicted and/or published *miR-34a/b/c* targets (Fig. 5D and Table S10), implying that miR-34a/b/c and the DREAM complex share a substantial proportion of targets and presumably cooperatively suppress these genes after p53 activation. Since the DREAM and E2F target gene signatures used here are highly overlapping, which is due to the binding to E2F sites by both E2F and DREAM complexes, we also detected shared targets of E2F and miR-34, such as Cyclin E1/CCNE1, which were upregulated in *miR-34a/b/c-KO* cells (Fig. 5D, Table S10). Collectively, these data suggest that miR-34 may contribute to the repression of mRNAs, which are also downregulated due to DREAM complex-mediated repression [27].

Next, we employed Gene Set Variation Analysis (GSVA) [28], a sample-wise pathway enrichment method, to analyze which pathways were significantly altered in *miR-34a/b/c-KO* vs. wild-type cells after 5-FU treatment, by estimating the variation of different pathway activities in an unsupervised manner (Fig. 5E). Activation of the p53 pathway and the upregulation of direct p53 targets was not significantly altered between *miR-34a/b/c-KO* and wild-type cells after 5-FU treatment. We observed significantly differential regulation of the *miR-34* target set between *miR-34a/b/c-KO* and wild-type cells, which was downregulated in *WT*, but upregulated in *miR-34a/b/c-KO* after 5-FU treatment (Fig. 5E). As already indicated by the ORA analysis, the differential regulation of mRNAs involved in cell proliferation (DREAM_TARGETS, E2F_TARGETS, etc.) was significantly changed in *miR-34a/b/c-KO* cells, which showed a severely diminished repression compared to *WT* cells (Fig. 5E). Furthermore, pathways involved in mitochondria and apoptosis were preferentially downregulated in *miR-34a/b/c-KO* cells after 5-FU treatment. We also observed a differential regulation of genes involved in ER and Golgi organization, as well as gene sets representing various autophagy pathways, and EMT, which either displayed elevated basal levels, and/or increased upregulation in *miR-34a/b/c-KO* cells.

### *miR-34a/b/c* inhibit multiple key autophagy-related genes

Next, we explored the role of *miR-34a* and *miR-34b/c* in the autophagic response to 5-FU treatment. As expected, the *pri-miR-34a* and *pri-miR-34b/c* transcripts were significantly induced in *WT* HCT116 cells when exposed to 5-FU (Fig. S6A). Also, mature miR-34a, miR-34b and miR-34c were significantly upregulated in *WT* HCT116 cells after 5-FU treatment, but not detectable in *miR-34a/b/c-KO* HCT116 cells (Fig. S6B). In line with the GSVA results shown in Fig. 5E, qPCR analysis of key autophagy-related mRNAs that are either predicted (*ATG13*, *ULK2*) or known (*ATG4B* [29], *ATG5* [30], *ATG9A* [31], *ULK1* [32], *XBP1* [33], *IRE1A* [34]) to be directly inhibited by *miR-34*, showed that they were either significantly upregulated in *miR-34a/b/c-KO* cells or downregulated in *WT* cells after 5-FU treatment (Fig. S6C). In addition, ectopic expression of *miR-34a* significantly repressed the expression of the aforementioned autophagy-related genes in SW480 cells (Fig. S6D). Therefore, *miR-34* presumably inhibits autophagy processes by targeting multiple key autophagy-related mRNAs in HCT116 CRC cells.

### *FOXM1* induces autophagy and transactivates *p62* and *ATG9A*

**Fig. 6:**
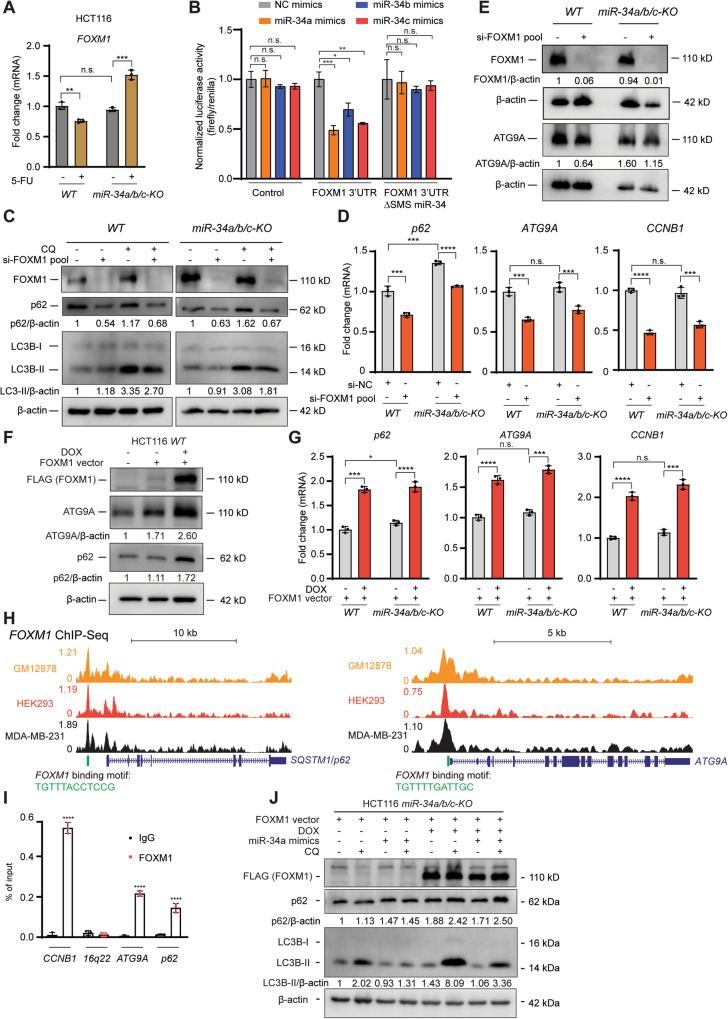
Upregulation of *FOXM1* mediates enhancing effects of *miR-34a/b/c* loss on autophagy by regulating *p62* and *ATG9A*. **A** qPCR analysis of *FOXM1* in *WT* or *miR-34a/b/c-KO* HCT116 cells after treatment with DMSO or 5-FU for 48 h. **B** Dual luciferase reporter assay was performed 48 h after *miR-34a/b/c-KO* HCT116 cells were transfected with indicated miRNA mimics and reporter plasmids. **C** Immunoblotting analysis of autophagic flux of cells transfected with 10 nM si-FOXM1 pools (siRNA pools specifically targeting *FOXM1*). *WT* and *miR-34a/b/c-KO* cells were transfected with si-NC or si-FOXM1 pool for 48 h. 20 μM of chloroquine was added for 4 h before harvesting cells for Western blot analysis. **D** qPCR analysis of indicated mRNAs in *WT* or *miR-34a/b/c-KO* HCT116 cells after transfection with si-NC or si-FOXM1 pool for 48 h. **E** Western blot analysis of ATG9A protein after transfection with si-NC or si-FOXM1 pool for 48 h. **F** Immunoblotting analysis of indicated proteins after HCT116 cells transfected with FOXM1 vector and addition of doxycycline (DOX) for 48 h. **G** qPCR analysis of indicated mRNAs in *WT* or *miR-34a/b/c-KO* HCT116 cells after transfection with FOXM1 vector and addition of DOX for 48 h. **H** Cistrome Data Browser representation of FOXM1 ChIP-Seq profiles at the genomic regions of *p62* and *ATG9A*. **I** ChIP-qPCR analysis of FOXM1 occupancy at the promoter regions of *p62* and *ATG9A*. Chromatin was enriched by anti-FOXM1 or anti-rabbit-IgG antibodies. *CCNB1* and *16q22* served as positive and negative control, respectively. **J** Immunoblotting analysis of autophagic flux of HCT116 *miR-34a/b/c-KO* cells co-transfected with *FOXM1* expression vector and NC mimics or miR-34a mimics. 20 μM of chloroquine (CQ) was added for 4 h before harvesting cells for Western blot analysis.

Among the known miR-34 targets that were differentially upregulated in *miR-34a/b/c-KO* cells after 5-FU treatment (Fig. 5A), *FOXM1* appeared to be a potential mediator of autophagy, since it had been previously linked to the regulation of autophagy [35, 36]. Since the upregulation of FOXM1 may cause the increased expression of the autophagy signatures observed in *miR-34a/b/c-KO* cells after 5-FU treatment (Fig. 5E), we explored whether *FOXM1* mediates the effects of *miR-34* on autophagy. First, the upregulation of FOXM1 in *miR-34a/b/c-KO* cells after 5-FU treatment was confirmed by qPCR (Fig. 6A). We further confirmed that *FOXM1* mRNA is a target of miR-34a/b/c by a dual luciferase reporter assay in HCT116 *miR-34a/b/c-KO* cells (Fig. 6B). The luciferase activity of a human *FOXM1* 3’-UTR reporter was repressed after co-transfection of miR-34a/b/c mimics, whereas that of a reporter with a mutant miR-34 seed-matching sequence (SMS) was refractory (Fig. 6B). The miR-34a/b/c-mediated repression of *FOXM1* mRNA was corroborated by querying the METAmiR34TARGET database [37], which shows that *FOXM1* mRNA was repressed after ectopic expression of miR-34a/b/c in various cell lines (Fig. S7A). Next, the *miR-34*-mediated repression of FOXM1 mRNA and protein was confirmed by ectopic expression of *pri-miR-34a* in SW480 cells (Fig. S7B, C). Furthermore, depletion of FOXM1 by a specific siRNA pool (Fig. 6C and Fig. S7D) significantly reduced autophagic flux in both *WT* and *miR-34a/b/c-KO* cells, which was indicated by the decreased turnover of endogenous LC3-II and p62 (Fig. 6C), supporting the assumption that *miR-34a/b/c* repress autophagic flux by downregulating *FOXM1*. Unexpectedly, depletion of FOXM1 also repressed p62 (Fig. 6C). p62 is a cargo receptor of autophagy and therefore should accumulate if autophagy is inhibited due to the decreased autophagy-mediated degradation of p62 [23, 38]. Therefore, the repression of p62 resulting from the depletion of FOXM1 cannot be attributed to autophagy inhibition but instead suggests that FOXM1 may transactivate p62 and potentially other autophagy-related genes. Indeed, depletion of FOXM1 repressed the p62 mRNA and also downregulated ATG9A at the mRNA and protein level (Fig. 6D, E). *CCNB1* (*Cyclin B1*), a bona fide FOXM1 target [39], was also significantly repressed after depletion of FOXM1 (Fig. 6D). In addition, ectopic expression of FOXM1 in HCT116 *WT* cells significantly increased the expression of p62 and ATG9A at protein and mRNA levels (Fig. 6F-G). Furthermore, FOXM1 occupancy at the promoter of *p62* and *ATG9A* was detected by publicly available FOXM1 ChIP-Seq data (Fig. 6H) from the Cistrome Data Browser [40]. Since a FOXM1 binding motif (Fig. S7E) was also identified under the corresponding FOXM1 ChIP-Seq peaks (Fig. 6H), FOXM1 presumably binds directly to the promoter of the *p62* and *ATG9A* genes and regulates their expression. The occupancy of FOXM1 at the promoter regions of *p62* and *ATG9A* was confirmed by qChIP (Fig. 6I). Importantly, ectopic expression of FOXM1 significantly induced autophagic flux in *miR-34a/b/c-KO* cells, and was sufficient to reverse the miR-34a mimics-mediated repression of autophagic flux (Fig. 6J), supporting that miR-34 represses autophagy by targeting *FOXM1*. Since *miR-34a/b/c* represent direct targets of p53, we propose that the p53-miR-34 axis negatively regulates autophagy by suppressing the expression of several autophagy-related genes via a coherent feed-forward regulation, in which miR-34 repress autophagy by directly targeting *FOXM1* and *ATG9A* mRNAs, as well as by indirectly repressing *p62* and *ATG9A* gene expression via targeting *FOXM1* (see Graphical abstract).

### Silencing of *ATG9A* in *miR-34a/b/c-KO* cells inhibits autophagic flux and re-sensitizes to 5-FU

**Fig. 7:**
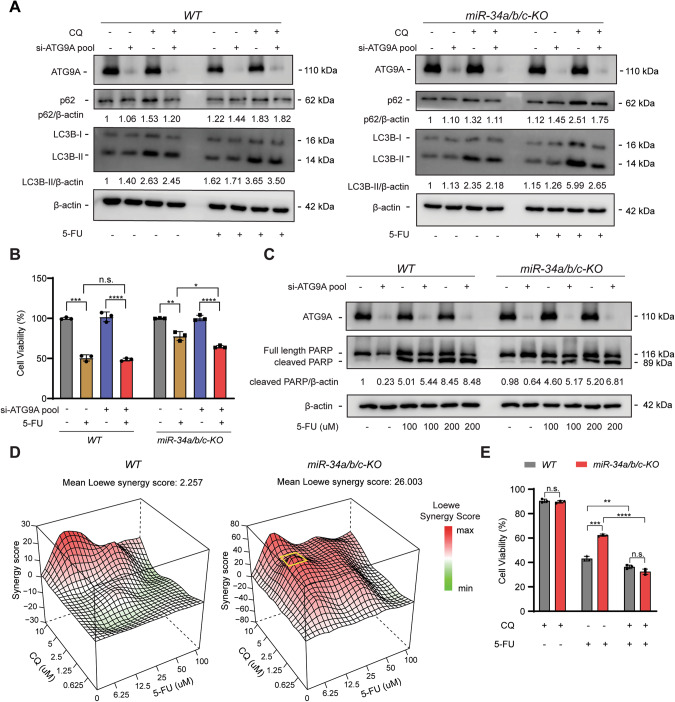
Inhibition of autophagy re-sensitized *miR-34a/b/c-KO* cells to 5-FU. **A** *WT* or *miR-34a/b/c-KO* cells were transfected with 10 nM si-NC or si-ATG9A pool for 24 h and then subjected to DMSO or 5-FU treatment for 48 h. 20 μM of chloroquine was added for the last 4 h before harvesting cells for Western blot analysis of the indicated proteins. **B** Cells were transfected with 10 nM si-NC or si-ATG9A for 48 h, and then re-seeded in 96-well plates and incubated for 24 h. After that, cells were treated with DMSO or 5-FU for 72 h before subjected to cell viability determination. **C** Western blot analysis of cleaved-PARP in DMSO or 5-FU treated cells transfected with si-NC or si-ATG9A for 24 h. **D** Analysis of synergistic effects of combined treatment of 5-FU and CQ in *WT* or *miR-34a/b/c-KO* cells. Cells were treated for 48 h with 5-FU and/or CQ as indicated and then subjected to cell viability analysis and Loewe synergy score estimation. The most synergistic concentrations are highlighted with a yellow square. **E** Cell viability assays showing cytotoxicity of 6.25 μM of 5-FU or 2.5 μM of CQ or their combination. Results are presented as the mean ± SD (n = 3) for **B** and **E** with *p < 0.05, **p < 0.01, ***p < 0.001, ****p < 0.0001, n.s. no significance.

We hypothesized that if increased autophagy accounts for the decreased 5-FU sensitivity in *miR-34a/b/c-KO* cells, then inhibition of autophagy should reverse this effect. To test this hypothesis, *ATG9A*, a key mediator of autophagy, was silenced using an siRNA pool specifically targeting *ATG9A* mRNA. ATG9A was downregulated by ca. 85% at the mRNA level and also effectively at the protein level (Fig. S8A, B). Furthermore, *ATG9A* protein levels were increased in *miR-34a/b/c-KO* cells but decreased in *WT* cells after 5-FU treatment, suggesting autophagy induction after 5-FU treatment may indeed be attenuated by the inhibitory effects of *miR-34* on autophagy-related genes (Fig. S8B). Surprisingly, depletion of *ATG9A* only marginally repressed 5-FU-induced autophagic flux in *WT* cells, but significantly repressed 5-FU-induced autophagic flux in *miR-34a/b/c-KO* cells (Fig. 7A), suggesting ATG9A may be a prominent mediator of autophagy in *miR-34a/b/c-KO* cells but not in *WT* cells, presumably since ATG9A was already repressed in *WT* cells after 5-FU treatment but significantly upregulated in *miR-34a/b/c-KO* cells after 5-FU treatment (Fig. S8B). In addition, cell viability assays showed that knockdown of *ATG9A* significantly re-sensitized *miR-34a/b/c-KO* cells to 5-FU, but had little effect in *WT* cells (Fig. 7B). Furthermore, depletion of *ATG9A* increased protein levels of cleaved-PARP, an apoptosis marker, in *miR-34a/b/c-KO* cells to a significantly greater degree when compared to *WT* cells after treatment with 5-FU (Fig. 7C). Taken together, these results suggest that ATG9A plays a pivotal role in the acquired resistance of *miR-34a/b/c-KO* cells to 5-FU presumably by enhancing autophagy and attenuating apoptosis.

### Synergistic effects of 5-FU and chloroquine in *miR-34a/b/c-KO* cells

We next used CQ (chloroquine) as an alternative autophagy inhibitor to determine whether it may also re-sensitize *miR-34a/b/c-*deficient cells towards 5-FU as observed for ATG9A inhibition. Indeed, we found that the combination of 5-FU and CQ resulted in a synergistic cytotoxicity in *miR-34a/b/c-KO* cells, whereas an additive cytotoxicity was observed in *WT* cells (Fig. 7D), as determined by the SynergyFinder 2.0 [41] algorithm. To validate the synergy map results we treated cells with a combination of two drugs at the concentration corresponding to the highest synergistic score area (highlighted by a yellow square in Fig. 7D). Indeed, by combined treatment with 2.5 μM of CQ and 6.25 μM of 5-FU, a significantly greater reduction in cell viability was achieved in *miR-34a/b/c-KO* cells than in *WT* cells (Fig. 7E). Therefore, the 5-FU-resistance of CRC cells with defects in the p53/miR-34a/b/c pathway may be alleviated by combining 5-FU-based treatment with CQ.

### Clinical relevance of *miR-34a/b/c-KO*-derived gene signatures

**Fig. 8:**
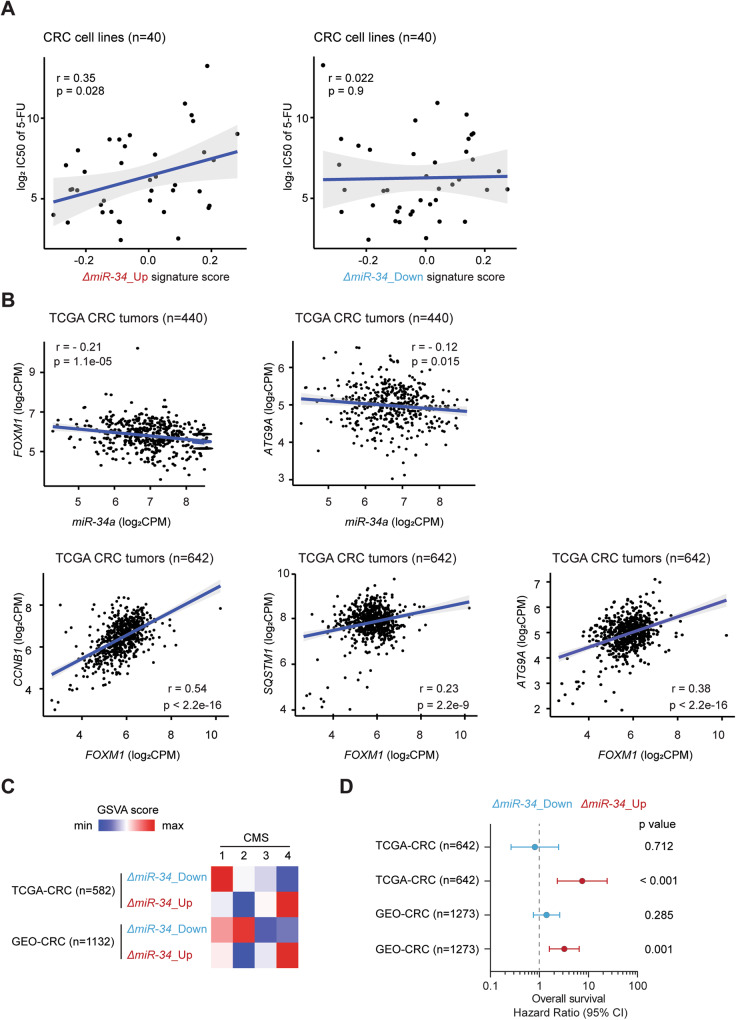
Clinical association analysis of selected genes and *miR-34a/b/c-KO*-derived gene signatures. **A** Scatter plots showing the correlations of the indicated *miR-34a/b/c-KO*-derived signatures scores with IC50 values of 5-FU in CRC cell lines. Two-sided Pearson correlation coefficient r and *p* values are indicated. **B** Scatter plots showing correlations between selected gene expressions in the TCGA-CRC patient cohort. Pearson correlation coefficient r and *p* values are indicated. **C** Associations between *miR-34a/b/c-KO*-derived signatures scores with CMS subtypes in the indicated CRC patient cohorts. **D** Cox regression model analysis of the associations between *miR-34a/b/c-KO*-derived signatures scores with overall survival in the indicated CRC patient cohorts.

Next, we explored the relation of *miR-34a/b/c-KO*-derived signatures with the chemotherapeutic response of CRC cells by interrogating the Genomics of Drug Sensitivity in Cancer (GDSC) datasets [42]. For this, we defined two *miR-34a/b/c-KO*-derived signatures (*ΔmiR-34_*Up and *ΔmiR-34_*Down), which comprise the significantly up- and downregulated genes in *miR-34a/b/c-KO* cells compared to *WT* cells (Table S7). Single sample *miR-34a/b/c-KO*-derived signature scores of CRC cell lines were computed by the GSVA algorithm in an unsupervised manner. Interestingly, the *ΔmiR-34_*Up signature score showed a significant, positive association with the IC50 values of 5-FU in CRC cell lines, whereas *ΔmiR-34_*Down signature score showed no significant association (Fig. 8A). Therefore, the observed resistance of *miR-34a/b/c*-deficient HCT116 cells to 5-FU may also be found in other CRC cell lines that exhibit a similar expression pattern.

Next, we analyzed whether the regulatory relationships between *miR-34*, *FOXM1*, *p62* and *ATG9A* identified above are conserved in primary CRCs. For this, we analyzed RNA expression data deposited in the TCGA database (TCGA-CRC) (n = 642) [43]. Expression of mature miR-34a displayed a significantly negative correlation with both *FOXM1* and *ATG9A*, whereas *FOXM1* showed a significantly positive correlation with *p62* and *ATG9A* as well as *CCNB1* (Fig. 8B), a bona fide FOXM1 target. Therefore, the FOXM1-mediated regulation of *p62* and *ATG9* is presumably conserved in primary CRCs. Taken together, these results suggest that the regulation of autophagy by miR-34a via repression of FOXM1 may be relevant in primary CRCs.

Finally, we sought to determine whether the *miR-34a/b/c-KO*-derived gene signatures are associated clinical parameters in primary CRC patient cohorts. Samples from the TCGA-CRC and a large, integrated GEO-CRC (n = 1273) patient cohort [44] were included in this analysis. *MiR-34a/b/c-KO*-derived signature scores of CRC patient samples were again computed by the GSVA algorithm. In line with the repression of EMT by miR-34 [45], *ΔmiR-34_*Up signature scores were highest in CMS4 (Fig. 8C), the consensus molecular subtype (CMS) characteristic for mesenchymal-like CRCs, which is associated with the worst overall patient survival [6]. Consistent with this finding, a Cox proportional-hazards model analysis showed that *ΔmiR-34_*Up signature scores were significantly associated with poor overall patient survival in both CRC cohorts, whereas *ΔmiR-34_*Down signature scores showed no significant association (Fig. 8D), supporting a tumor suppressive role of miR-34a/b/c. Furthermore, *ΔmiR-34_*Up signature scores were also significantly associated with poor overall patient survival in 17 out of 33 TCGA cancer types (Fig. S9), indicating that these findings may also be relevant to other tumor entities as *p53* and *miR-34a/b/c* inactivation are common in many types of tumors.

## Discussion

Here, we demonstrated a complementary role of *miR-34a* and *miR-34b/c* as only concomitant deletion of both isoforms resulted in significantly reduced suppression of proliferation after p53 activation, enhanced migration, invasion and EMT, as well as reduced sensitivity to chemotherapeutics. The latter was due to increased stress-induced autophagic flux and upregulation of autophagy-related genes after 5-FU treatment, which also resulted in a decreased rate of apoptosis. Furthermore, inhibition of autophagy re-sensitized *miR-34a/b/c-KO* cells to 5-FU. Genome-wide gene expression analysis revealed that deletion of *miR-34a/b/c* in the HCT116 cell line resulted in impaired functions mediated by the gene-repressive effect of the p53-DREAM axis and enhanced autophagy after exposure to 5-FU, which is presumably due to the upregulation of *FOXM1*, a miR-34 target and transcription factor that transactivates key factors involved in cell cycle and autophagy processes. Notably, FOXM1 was required for increased autophagy, presumably by transactivating *p62* and *ATG9A*. Since *ATG9A* is an established *miR-34* target, we propose a feed-forward loop in which *miR-34* represses autophagy by directly targeting *FOXM1* and *ATG9A* mRNAs, as well as by indirectly repressing *p62* and *ATG9A* gene expression via targeting *FOXM1*. Since a gene signature comprised of genes significantly upregulated as a result of the combined deletion of *miR-34a* and *miR-34b/c* was significantly associated with poor prognosis as well as 5-FU resistance, and the combination of chemotherapeutics with autophagy inhibition resulted in synergistic effects in a *miR-34*-deficient context, these findings are presumably of clinical relevance.

The combined inactivation of *miR-34a* and *miR-34b/c* in unstressed HCT116 cells resulted in enhanced migration and invasion, which was accompanied by elevated EMT. These observations were further corroborated by an RNA-Seq analysis, which showed enrichment of EMT pathways in *miR-34a/b/c-KO* cells, and by detection of a significant correlation between *ΔmiR-34_*Up signature score and poor survival of CRC patients. However, singular deletion of either *miR-34a* or *miR-34b/c* showed no significant effects on migration, invasion or EMT. Collectively, these data suggest a complementary role of *miR-34a* and *miR-34b/c* in repressing these processes.

By inactivating *miR-34a* and *miR-34b/c* alone or in combination using a genetic approach, we showed that the combined deletion of *miR-34a* and *miR-34b/c* has a significant effect on promoting stress-induced autophagic flux and reducing sensitivity to chemotherapeutics, whereas abrogation of endogenous expression of either of them was not able to render cells chemo-resistant.

In this study, we employed two approaches to estimate autophagic flux, which is a more reliable measure of autophagy activity than measuring the steady level of LC3 [46, 47]. On one hand, the turnover of LC3-II and p62 was determined to measure autophagy using immunoblotting assays. In addition, cells stably expressing a GFP-LC3-RFP autophagy probe were generated to measure the cumulative degradation of GFP-LC3 in a quantitative manner. By utilizing these two methods, we showed that *miR-34a/b/c*-deficient cells consistently displayed higher autophagic flux when stressed by chemotherapeutics, starvation or endoplasmic reticulum stress. Therefore, *miR-34a/b/c* presumably plays a pivotal role in suppressing stress-induced autophagy.

Genome-wide gene expression profiling analysis revealed an upregulation of autophagy-related pathways in *miR-34a/b/c*-deficient cells after 5-FU treatment, thus corroborating the enhanced autophagic flux observed in *miR-34a/b/c-KO* cells.

Interestingly, we also observed a strongly diminished repression of genes related to cell proliferation in *miR-34a/b/c-KO* cells after 5-FU treatment. A large number of these genes was previously identified as direct targets of the DREAM complex, which suggested a compromised DREAM complex activity in *miR-34a/b/c-KO* cells. Since the DREAM complex can regulate both E2F targets via binding to E2F binding sites, as well as FOXM1 targets via binding to CHR binding sites [48], the repression of both E2F and FOXM1 targets was also abrogated in *miR-34a/b/c-KO* cells. Interestingly, miR-34 regulates several upstream signaling components that affect DREAM activity, which showed either diminished repression or upregulation in *miR-34a/b/c-KO* cells after 5-FU treatment, such as CCNE1 (Cyclin E1) [49] and CCNE2 (Cyclin E2) [50]. Upregulation of CCNE1 and CCNE2 by the loss of *miR-34a/b/c* would presumably increase phosphorylation of p130 via CDK2 and result in disassembly of the DREAM complex [48].

Interestingly, we noted that miR-34a/b/c and the DREAM complex share a substantial proportion of targets and presumably cooperatively suppress these genes after p53 activation. Among these were several transcription factors that affect DREAM complex function, such as MYBL2 (B-MYB) [51], E2F1 [51], as well as FOXM1 [52], which are direct targets of miR-34 that showed upregulation in *miR-34a/b/c-KO* cells after 5-FU treatment. Upregulation of B-MYB by the loss of *miR-34a/b/c* presumably competes with DREAM complex for the MuvB complex and facilitate the formation of B-MYB-MuvB-FOXM1 complex since binding of B-MYB to MuvB is necessary for recruiting FOXM1 [53]. Moreover, the upregulation of E2F1 in *miR-34a/b/c-KO* cells likely counteracts the repression of E2F targets by DREAM.

FOXM1 represents a key factor for cell cycle progression [48], as well as a prognostic marker of CRC [54]. The *miR-34*-dependent upregulation of FOXM1 in *miR-34a/b/c-KO* cells after 5-FU treatment presumably competed with the DREAM complex for the MuvB core complex, thereby switching the MuvB-based complexes from DREAM repressor to B-MYB-MuvB-FOXM1 activators and exerting opposite functions [27, 48]. Taken together, the combinatorial effects of loss of *miR-34a/b/c* on these regulations are likely contribute to either abrogated repression or activation of DREAM, E2F and FOXM1 target genes after activation of p53.

Moreover, FOXM1 is a known inducer of autophagy [35, 36], a target of miR-34a [52] and presumably a prominent effector of *miR-34* in this context. Here, we showed that FOXM1 is also directly targeted by miR-34b and miR-34c. The elevated autophagy resulting from the deletion of *miR-34a/b/c* is presumably, at least in part, caused by the upregulation of FOXM1 as depletion of FOXM1 significantly repressed autophagic flux, whereas ectopic expression of FOXM1 significantly induced autophagic flux.

Here, we showed that FOXM1 induced autophagy, presumably through transactivating its target genes, *SQSTM1/p62* and *ATG9A*. P62 is one of the most prominent autophagy receptors [55]. Importantly, p62 is at the crossroads of autophagy and the ubiquitin-proteasome system, linking these two major quality control systems responsible for degradation of proteins and organelles in eukaryotic cells via its LC3-binding domain and ubiquitin-associated domain respectively [56]. The upregulation of *p62* by the de-regulation of *miR-34*/*FOXM1* axis presumably not only increases autophagy, but also affects the proteasome system, which could perturb cellular homeostasis. ATG9A is the only transmembrane protein of the core autophagy machinery [57]. ATG9A-containing vesicles form seeds that establish contact sites to facilitate the de novo formation of autophagosomes [58, 59]. Thus, the *miR-34*-dependent downregulation of ATG9A may presumably impair autophagosome formation and expansion, and thereby inhibit autophagy. Importantly, the expression of mature miR-34a showed a negative correlation with *FOXM1* and *ATG9A*, whereas *FOXM1* displayed a significant, positive correlation with *p62* and *ATG9A* expression in the TCGA-CRC patient cohort, indicating that such regulatory connections may also exist in primary CRCs.

CQ (chloroquine) and its derivative hydroxy-chloroquine are drugs widely used to treat malaria [60], amebiasis [61] and rheumatic diseases [62]. Importantly, CQ is a potent autophagy inhibitor that blocks autophagy at a late step by preventing autophagosome and lysosome fusion [63] and was therefore tested in clinical trials for use as an anti-tumor drug [64, 65]. Interestingly, CQ was shown to potentiate the cytotoxicity of 5-FU in colon cancer and pancreatic cancer cell lines [66, 67]. Here, we showed that a combination of 5-FU and CQ resulted in a synergistic effect specifically in a *miR-34*-deficient context whereas an additive effect was observed in *miR-34a/b/c*-proficient CRC cells. Therefore, the inactivation of *miR-34a/b/c* may sensitize tumor cells to autophagy inhibition. In the future, it should be tested whether combinations of other autophagy inhibitors and 5-FU-related drugs also elicit a synergistic toxicity towards tumor cells with defects in the p53/*miR-34a/b/c* pathway.

## Materials and methods

### Cell culture and treatments

The colorectal cancer cell lines HCT116, SW480 and HCT15 were cultured in McCoy’s 5A medium, with 10% fetal bovine serum and 1% penicillin/streptomycin, at 5% CO_2_ and 37 °C. For conditional *pri-miR-34a* expression from pRTR vectors, doxycycline (Sigma-Aldrich, St. Louis, MO) was dissolved in water and used at a final concentration of 100 ng/ml. To select for cells harboring pRTR vectors, cell pools were cultured at a final concentration of 4 μg/ml puromycin. pCW57.1-FOXM1c (obtained from Addgene, a gift from Adam Karpf; Plasmid #68810) was used to ectopically express FLAG-tagged human FOXM1c in cells in a doxycycline-inducible manner [68]. Hsa-miR-34a/b/c-5p mimics and corresponding negative control mimics were purchased from Qiagen (Hilden, Germany). FlexiTube GeneSolution GS2305 for FOXM1 (consisting of a pool of 4 different siRNAs for FOXM1) and control siRNAs were purchased from Qiagen (Hilden, Germany). Two siRNAs (#s35505 and #s35506) targeting ATG9A and the corresponding negative control siRNAs were purchased from ThermoFisher Scientific (Waltham, MA, USA).

### RNA isolation and real-time polymerase chain reaction (qPCR) analysis

Total RNA from cultured cells was isolated using High Pure RNA Isolation Kit (Roche) according to manufacturer’s protocol. One microgram total RNA was then used to generate cDNA using Verso cDNA Synthesis Kit (Thermo Scientific). qPCR analysis of mRNA was performed using LightCycler 480 (Roche) and the Fast SYBR Green Master Mix (Applied Biosystems). Mature miRNAs were isolated using miRNeasy Mini Kit (QIAGEN). Sequence information of the primers is provided in Table S3.

### Chromatin immunoprecipitation

Chromatin immunoprecipitation (ChIP) in HCT116 cells was performed according to protocol provided in the iDeal ChIP-qPCR kit (Diagenode, Belgium). The sequence information of the qChIP primers used here is provided in Table S4. 16q22 region was used as a negative control in qChIP assay, which is devoid of enriched FOXM1 signal (Fig. S7F) as determined by public FOXM1 ChIP-seq data from the Cistrome Data Browser [40].

### CRISPR-Cas9-mediated deletion of *miR-34*

Two single-guide RNAs (sgRNAs) targeting flanks of pre-miRNA encoding locus (Table S1) were designed using the CRISPR design tool at benchling.com. Each of them was cloned via two complementary DNA oligonucleotides into the *Bbs*I sites of pSpCas9(BB)-2A-GFP [69] to generate sgRNA expression plasmids, as described previously [70]. HCT116 cells were then transfected with 2.5 μg of each sgRNA-pSpCas9(BB)-2A-GFP plasmid, or transfected with “empty” pSpCas9(BB)-2A-GFP harboring no sgRNA. After transfection for 48 h, GFP-positive cells were sorted into 96-wells using a FACSARIA cell sorter (BD Biosystems) and expanded as single-cell clones for two weeks. Cells transfected with “empty” pSpCas9(BB)-2A-GFP vectors were treated in a similar manner to obtain wild-type single-cell clones. Subsequently, genomic DNA of individual single-cell clones were screened by genotyping PCR for appropriate deletions of pre-miRNA encoding regions using two pairs of genotyping screening primers listed in Table S2. Clones with deletion of both pre-miRNA encoding alleles were analyzed by qPCR to confirm the loss of mature miRNAs expression.

### Modified Boyden-chamber assay

Migration and invasion assays using modified Boyden-chambers were performed as described previously [16]. In brief, 1 × 10^5^ cells in serum-free medium were seeded in the upper chamber (8.0 μM pore size membrane; Corning), whereas medium containing 10% fetal bovine serum was placed in lower chamber as chemoattractant. For migration assay, cells were cultured for 24 h. For invasion assay, chamber membrane was first coated with 100 μl Matrigel matrix (Corning) at a concentration of 300 μg/ml in serum-free medium. Subsequently, cells were seeded and cultured for 48 h. Subsequently, non-motile cells at the top of the filter were removed and the cells in the bottom chamber were fixed with ice-cold methanol for 20 min at room temperature and stained with 0.5% crystal violet for 30 min. Fold change of migrated cells were calculated by normalizing to corresponding control groups.

### Wound healing assay

Cell-free gap was created by seeding and culturing cells in Culture-inserts (80241; IBIDI, Martinsried, Germany) until confluent cell monolayer was formed. Cells were treated with 10 μg/mL mitomycin C (M4287; Sigma-Aldrich, Germany) for 2 h before removing Culture-inserts to create cell-free gap. After washing twice with HBSS to remove mitomycin C and detached cells, cells were filled with medium. The cell-free gap was monitored immediately and after 36 h by phase contrast microscope and corresponding pictures were taken.

### Western blot analysis

Cells were lysed in RIPA lysis buffer with complete mini protease inhibitors (Roche, Basel, Switzerland) and PhosSTOP Phosphatase Inhibitor Cocktail Tablets (Roche). Lysates were sonicated and then centrifuged at 13,000 rpm for 20 min at 4 °C. Protein concentration was measured with BCA Protein Assay Kit (Thermo Fisher Scientific) according to manufacturer’s instructions. 30 μg protein per lane were separated by 12% SDS-PAGE gels and transferred to PVDF membranes (Millipore). ECL (Millipore) system was used and imaged through LI-COR Odyssey FC imaging system (Bad Homburg, Germany). Western blot signals were quantified using Image Studio (LI-COR). Antibodies are list in Table S5.

### Apoptosis detection with FITC Annexin V staining

Apoptosis analysis was carried out by flow cytometry with FITC Annexin V Apoptosis Detection Kit I (556547; BD Pharmingen™) according to manufacturer’ instructions. In brief, supernatant containing apoptotic cells was collected before harvesting cells by trypsinization (EDTA-free). Cells were then washed twice and resuspended in 1× binding buffer at a concentration of 1 × 10^6^ cells/ml. One hundred microloters of solution (1 × 10^5^ cells) were incubated with 5 µl of FITC Annexin V and 5 µl Propidium Iodide (PI) for 15 min at room temperature in the dark before adding 400 µl of 1× binding buffer. Samples were analyzed within 1 h by flow cytometry using an Accuri C6 flow cytometry instrument (BD Biosciences).

### Apoptosis evaluation with cell cycle analysis by Propidium Iodide staining

Cells were seeded in 6-well plates at a density of 2 × 10^5^ cells per well. After 24 h, cells were treated as indicated for 48 h. Both supernatant and attached cell fractions were collected and combined. Cells were washed twice with HBSS and fixed with ice-cold 70% ethanol overnight at −20°C. Fixed samples were washed once with HBSS and then resuspended by Propidium Iodide (PI) staining solution. Cell-cycle distribution was measured using an Accuri C6 flow cytometry instrument (BD Biosciences) and analyzed with the CFlow software. Sub-G1 cell population represents apoptotic cells.

### Cell viability assay

Cell viability was determined by Cell Counting Kit-8 (CCK-8) (Dojindo EU GmbH) according to manufacturer’s instructions. Briefly, 3000 cells per well were seeded into 96-well plates and treated with the indicated cytostatic agents for the indicated durations. 10% CCK-8 solution was added to each well at end point and incubated for 2 h. Absorbance was measured at 450 nm on a Berthold Orio II Microplate Luminometer (Berthold, Bad Wildbad, Germany). GraphPad Prism (v9.31; GraphPad Software, USA) was used to generate dose-response curves and estimated corresponding half-maximal inhibitory concentration (IC50) values of indicated drugs.

### Assessment of cell proliferation by real‑time impedance measurement

Cell proliferation was determined by real-time cellular impedance measurement using a xCELLigence Real-Time Cell Analyzer (RTCA) (Roche Diagnostics GmbH, Penzberg, Germany) as described previously [71]. Cells were seeded at a density of 3000 cells per well of the E-plate and treated as indicated after 24 h. Impedance was measured every 60 min for 96 h and reported as a dimensionless parameter called Cell Index by RTCA software . The magnitude of Cell Index is dependent on cell number, cell morphology and cell size and on the strength of cell adherence to the substrate coating the plate [72]. Because the Cell Index does not solely depend on cell number, the results of impedance measurements were validated by end-point cell number counting. Therefore, cells were simultaneously seeded into 96-well plates and treated in the same manner. Cells at the end point were counted using a Neubauer-chamber.

### Autophagic flux assay with GFP-LC3-RFP probe

Cells stably expressing GFP-LC3-RFP were generated by transfected of a GFP-LC3-RFP plasmid (obtained from Addgene, a gift from Noboru Mizushima; Plasmid #84573) with lipofectamine LTX (Invitrogen) followed by puromycin selection for two weeks. Cells were stressed as indicated and subjected to flow cytometry analysis using an Accuri C6 instrument (BD Biosciences) to assess GFP and RFP fluorescence intensities.

### RNA-Seq analysis

Total RNAs from HCT116 cells were isolated using a High Pure RNA Isolation Kit (Roche) with an on-column DNase digestion according to the manufacturer’ protocol. Random primed cDNA libraries were constructed and sequenced using the NovaSeq 6000 (Illumina, San Diego, CA, USA) platform by GATC (Konstanz, Germany). Each sample was covered by at least 30 million paired-end read pairs of 150 bp length. RNA-Seq FASTQ files were processed using the RNA-Seq module implemented in the CLC Genomics Workbench v20.0.2 software (Qiagen Bioinformatics, Dusseldorf, Germany) and mapped to the GRCh38/hg38 human reference genome and its associated gene and transcript annotation (ENSEMBL) using the settings mismatch cost = 2, insertion cost = 2, deletion cost = 3, length fraction = 0.8, and similarity fraction = 0.8. RNA-Seq data were filtered to exclude weakly expressed transcripts with less than 20 mapped exon reads in all samples from the analysis and subjected to upper quartile normalization using the R/Bioconductor RUVSeq (remove unwanted variation from RNA-Seq data, Version 1.18.0) package [73]. Differential gene expression analysis was performed with DESeq2 (Version 1.24.0) [74] after normalization using the RUVg approach to remove variation between RNA samples resulting from differences in library preparation. Principal component analysis (PCA) was performed using the PCA functionality of the EDASeq R package as implemented in RUVSeq. For the identification of miR-34 targets, we used recently published lists of miR-34 targets and of the top 1000 ranked miR-34a targets, which were generated using the METAmiR34TARGET website [37]. Pathway over-representation analysis (ORA) using a hypergeometric testing method was performed via the enricher function implemented in clusterProfiler 4.0 [75]. Gene sets were obtained from the Molecular Signatures database (MSigDB) [76]. AUTOPHAGY_CORE gene set was obtained from Bordi M et al. [77]. Sample-wise variations of different pathway activities were estimated in a non-parametric, unsupervised manner via the GSVA package [28]. EdgeR was used to test the interaction effects between genotype and treatment condition and determine the set of mRNAs showing genotype-dependent differences in regulation after 5-FU treatment [78].

### Analysis of gene expression and clinical data from public databases

For the analysis of human colorectal cancer (CRC) samples, we retrieved expression and clinical data of the TCGA-CRC samples from GDC portal [79] and a large integrated CRC samples from GEO repository [44]. CMS (consensus molecular subtypes) [6] classifications of CRC samples were determined using the CMScaller R package v.2.0.1 [80]. 5-FU sensitivity data of CRC cell lines were obtained from the Genomics of Drug Sensitivity in Cancer (GDSC) database [42], and corresponding gene expression data of CRC cell lines were obtained from the Cancer Cell Line Encyclopedia (CCLE) [81]. The Cox proportional-hazards regression model was applied to investigate the hazard ratio for assessing the association between patients’ overall survival time and *miR-34-a/b/c-KO*-derived signatures scores in CRC patient cohorts.

### 3′-UTR dual reporter assay

The full length human *FOXM1* 3′-UTR was PCR-amplified from cDNA obtained from HCT116 cells. The PCR product was cloned into pGL3-control-MCS [82]. To delete the miR-34a/b/c-5p seed-matching sequence (SMS) in the *FOXM1* 3′-UTR a QuikChange II XL Site-Directed Mutagenesis Kit (Stratagene, San Diego, CA, USA) was used according to the manufacturer’s instructions. All plasmids were verified by Sanger sequencing. The oligonucleotides used for cloning and mutagenesis were listed in Table S6. For the dual reporter assays, HCT116 *miR-34a/b/c-KO* cells were seeded into a 12-well plate at 3 × 10^4^ cells/well and cultivated for 24 h before transfection. Transfections were performed using HiPerFect Transfection Reagent (Qiagen) with 10 nM of indicated miRNA mimics and 100 ng of indicated reporter vectors and 20 ng Renilla plasmid as normalization control. After 48 h of incubation with the indicated treatments, luciferase activity was measured with a Dual Luciferase Reporter assay kit (Promega) according to manufacturer’s instructions using an Orion II Microplate Luminometer (Berthold, Germany) and the Simplicity software package.

### Drug combination synergy scores analysis

To analyze the synergistic effects of the combination between CQ and 5-FU, synergy scores were calculated by SynergyFinder 2.0 [41] with the Loewe model [83], using the dose-response matrix derived from cell viability assays.

### Statistical analysis

Statistical analyses were performed with Prism 9 (GraphPad Software, San Diego, CA, USA) or R (version 4.2.2). Each set of experiments was repeated at least three times. Student’s t test (unpaired, two-tailed) was used to determine statistical significance of differences between two groups of samples. *P* values less than 0.05 were considered statistically significant (*p < 0.05; **p < 0.01; ***p < 0.001; ****p < 0.0001; n.s. not significant). Benjamini–Hochberg method was used to adjust for multiple testing error and calculate false discovery rate (FDR).

## Supplementary information

Supplemental Material

## Data Availability

RNA expression profiling data obtained in this study were deposited in the Gene Expression Omnibus website (accession no. GSE227230). All data, analytic methods, and study materials will be made available to other researchers upon reasonable request.
