# Supplementary material for: CRISPR/Cas9-mediated inactivation of miR-34a and miR-34b/c in HCT116 colorectal cancer cells: comprehensive characterization after exposure to 5-FU reveals EMT and autophagy as key processes regulated by miR-34
Source: Cell Death Differ. 2023 Jul 24;30(8):2017–34. doi: 10.1038/s41418-023-01193-2 (PMC10406948; doi:10.1038/s41418-023-01193-2)
Supplement: Supplementary file 1 — Supplemental Material [file 41418_2023_1193_MOESM1_ESM.pdf]

Supplementary Material

CRISPR/Cas9-mediated inactivation of *miR-34a* and *miR-34b/c* in HCT116 colorectal cancer cells: comprehensive characterization after exposure to 5-FU reveals EMT and autophagy as key processes regulated by miR-34

Zekai Huang, Markus Kaller and Heiko Hermeking

Inventory of Supplementary Material:

Supplementary Figure 1
Related to Figure 1
Schematic illustrations of deleting *miR-34* using a CRISPR-Cas9 approach and genotyping screening results

Supplementary Figure 2
Related to Figure 1

Supplementary Figure 3
Related to Figure 2
Dose-response curves and flow cytometric analysis of FITC Annexin V and PI staining after chemotherapeutics treatment

Supplementary Figure 4
Related to Figure 2
Flow cytometric analysis of cells stably expressing GFP-LC3-RFP

Supplementary Figure 5
Related to Figure 3

Supplementary Figure 6
Differential expression of autophagy-related miR-34 targets after deletion of *miR-34a/b/c*

Supplementary Figure 7
Related to Figure 6

Supplementary Figure 8
Related to Figure 7
Analysis of si-ATG9A pools efficiency

Supplementary Figure 9
Related to Figure 8
Cox regression model analysis of the associations between *ΔmiR-34*_Up signature score with overall patient survival in the indicated TCGA pan-cancer patient cohorts

Supplementary Figure 10
Original Western blots (Uncropped membranes)

Supplementary Table 1
Sequence information for guide RNAs used for *miR-34a/b/c* deletion

Supplementary Table 2
Sequence information for genotyping primers used for selecting *miR-34a/b/c* homozygous deletion

Supplementary Table 3
Oligonucleotides used for qPCR

Supplementary Table 4
Oligonucleotides used for qChIP

Supplementary Table 5
List of antibodies

Supplementary Table 6
Oligonucleotides used for reporter plasmids

Supplementary Table 7
List of mRNAs significantly regulated in HCT116 *miR-34a/b/c-KO* cells when compared to *WT* cells

Supplementary Table 8
List of mRNAs significantly regulated in HCT116 *WT* cells when treated with 5-FU

Supplementary Table 9
List of mRNAs significantly regulated in HCT116 *miR-34a/b/c-KO* cells when treated with 5-FU

Supplementary Table 10
List of mRNAs to generate the K-means clustering heatmap

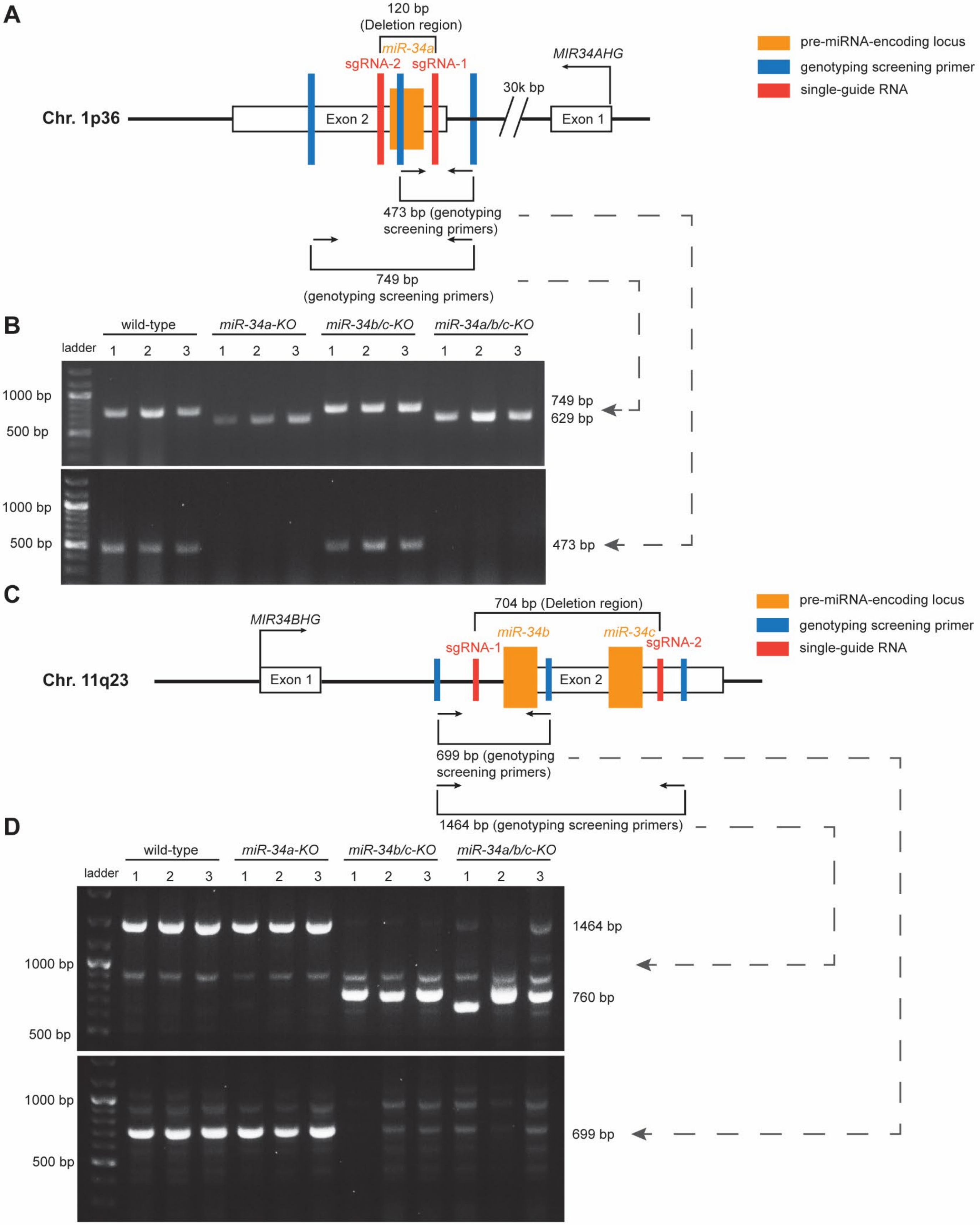

Figure S1. Schematic illustrations of deleting *miR-34* using a CRISPR-Cas9 approach and genotyping screening results. (**A**) Schematic of *miR-34a* genomic location and deletion of the mature miR-34a encoding region using a CRISPR-Cas9 approach. MiR-34a encoding locus is indicated by an orange column and two single-guide RNAs (sgRNAs) targeted regions are shown by red columns. Three genotyping screening primers indicated in blue were designed to determine cells with homozygous *miR-34a* deletion. (**B**) Genotyping results of deletion of the *miR-34a* locus. Intact *miR-34a* locus displayed 749 bp bands while deletion of it showed 629 bp bands (upper panel). The absence of *miR-34a* locus was confirmed by the failure to detect the 473 bp products (lower panel). (**C**) Schematic of *miR-34b* and *miR-34c* genomic locations and deletion of the mature miRNAs encoding regions using a CRISPR-Cas9 approach. MiR-34b and miR-34c encoding loci are indicated by orange columns and two single-guide RNAs (sgRNAs) targeted regions are shown by red columns. Three genotyping screening primers indicated in blue were designed to determine cells with homozygous *miR-34b/c* deletion. (**D**) Genotyping results of the deletion of *miR-34b/c* loci. Intact *miR-34b/c* loci displayed 1464 bp bands while deletion of them showed 760 bp bands (upper panel). The absence of *miR-34b/c* loci was confirmed by the failure to detect the 699 bp products (lower panel).

Figure S1

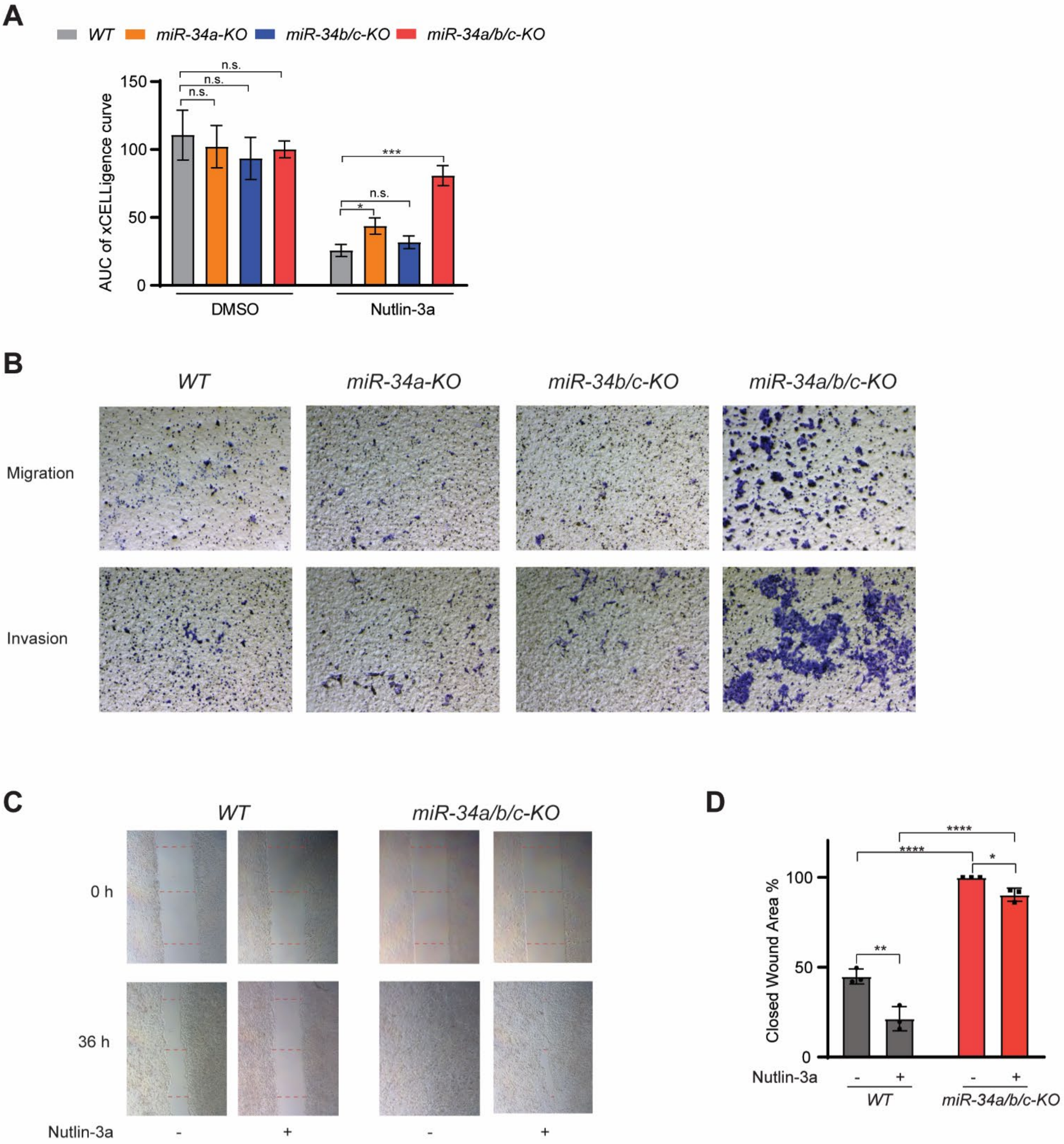

Figure S2. (**A**) Bar plot showing the AUC (area under the curve) of the xCELLigence growth curves shown in Fig. 1D. (**B**) Representative images of cellular migration and invasion capacity determination using Boyden-chamber assays. (**C**) Representative images of cellular migration capacity determination of indicated cells treated with DMSO or Nutlin-3a using wound healing assays. (**D**) Quantification of wound healing assay results in **C**. Results are presented as the mean +/− SD (n = 3) for **A** and **D** with *: $p < 0.05$, **: $p < 0.01$, ***: $p < 0.001$, ****: $p < 0.0001$, n.s.: no significance.

Figure S2

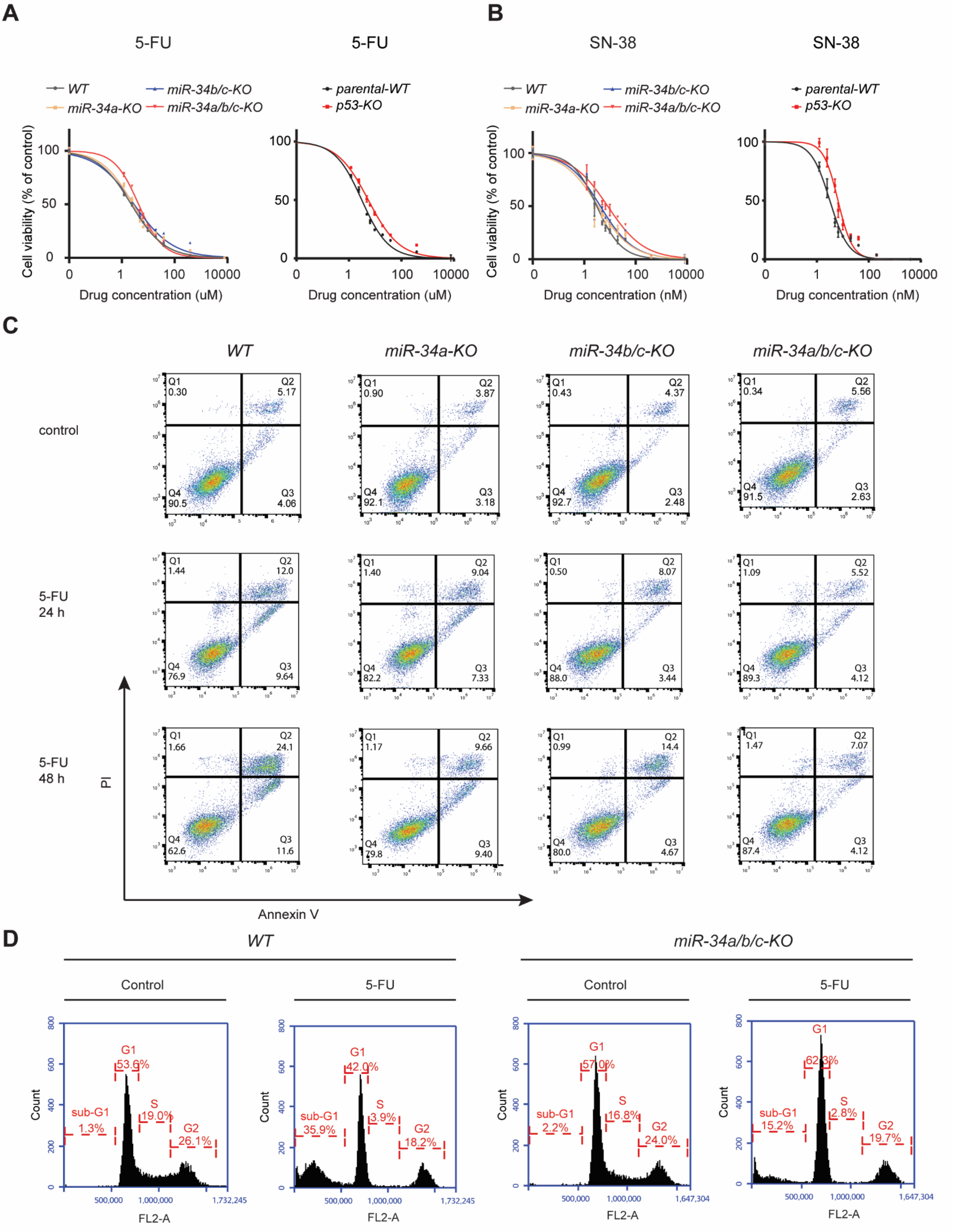

Figure S3. Dose-response curves and flow cytometric analysis of FITC Annexin V and PI staining after chemotherapeutics treatment. (A, B) Representative dose-response curves of HCT116 cells with various *miR-34* or *p53* genotypes in response to 5-FU or SN-38. Cells were treated with a wide range of concentration of the indicated therapeutic drugs for 72 hours and then subjected to CCK-8 assay. (**C**) Representative flow cytometry results. The indicated cells were treated with 5-FU for 24 or 48 hours and then subjected to FITC Annexin V staining and flow cytometric analysis to detect apoptotic cells. (**D**) Representative results of DNA content analysis by flow cytometry. The indicated cells were treated with DMSO or 5-FU for 48 hours and then subjected to PI staining and flow cytometric analysis to determine the sub-G_1 population.

Figure S3

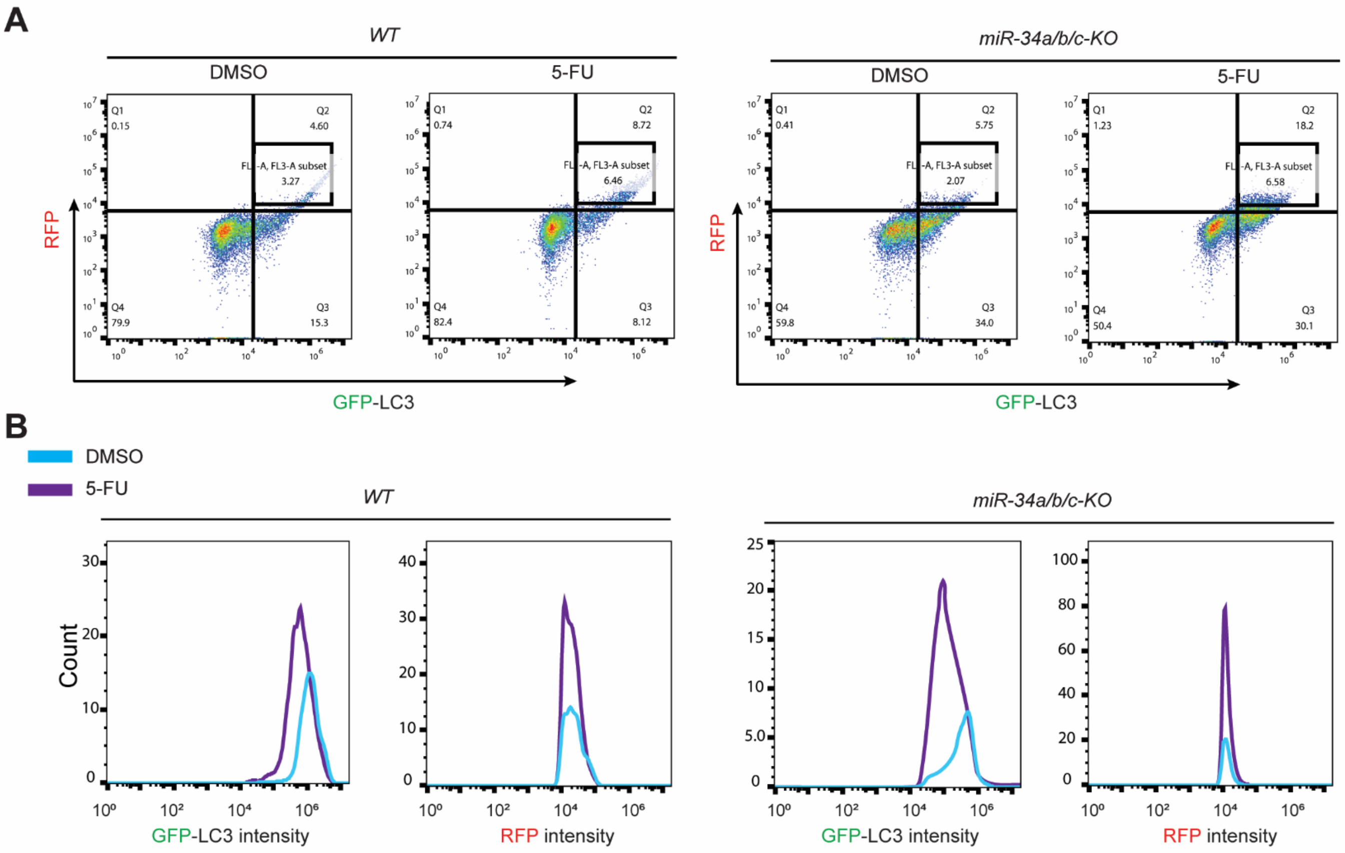

Figure S4. Flow cytometric analysis of cells stably expressing GFP-LC3-RFP. (**A**) An example of gating displaying cells stably expressing GFP-LC3-RFP after DMSO or 5-FU treatment. (**B**) An example of flow cytometry histograms displaying cell counts versus fluorescence intensity in cells stably expressing GFP-LC3-RFP treated with DMSO or 5-FU.

Figure S4

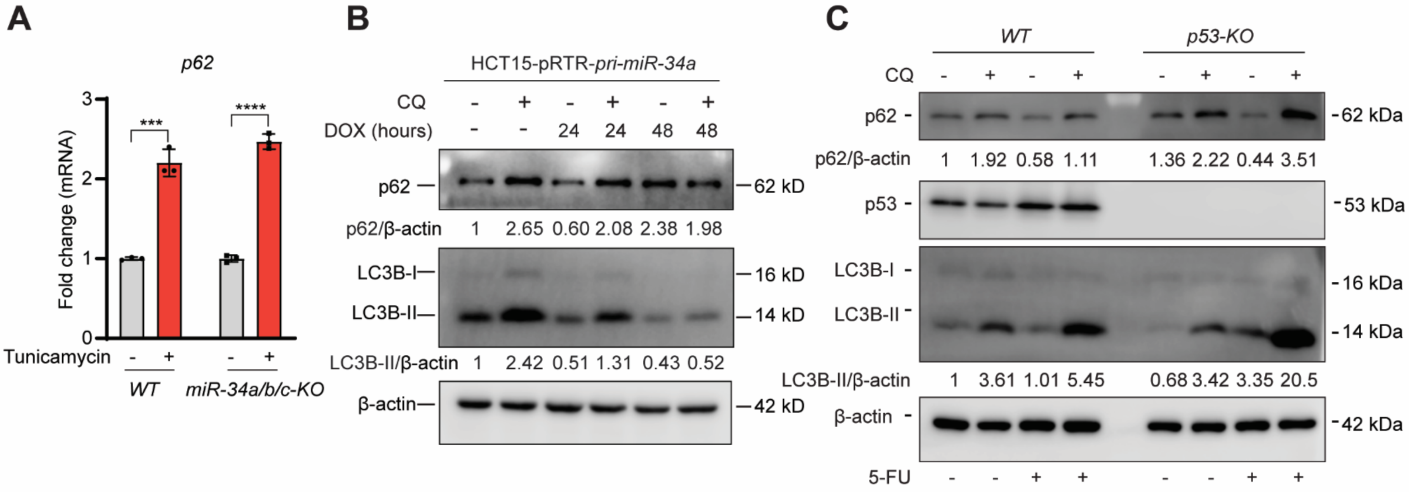

Figure S5. (**A**) qPCR analysis of p62 after treatment of HCT116 cells with 1 ug/ml Tunicamycin for 24 hours. (**B**) Doxycycline was added as indicated to induce ectopic expression of *pri-miR-34a* in HCT15 cells. 20 µM of CQ (chloroquine) was added for the last 4 hours before Western blot analyses of the indicated proteins. (**C**) *WT* and *p53-KO* HCT116 cells were treated with DMSO or 5-FU for the indicated durations and analyzed by immunoblotting. 20 µM of CQ (chloroquine) was added for 4 hours before harvesting cells. Results are presented as the mean +/− SD (n = 3) for **A** with ***: $p < 0.001$, ****: $p < 0.0001$.

Figure S5

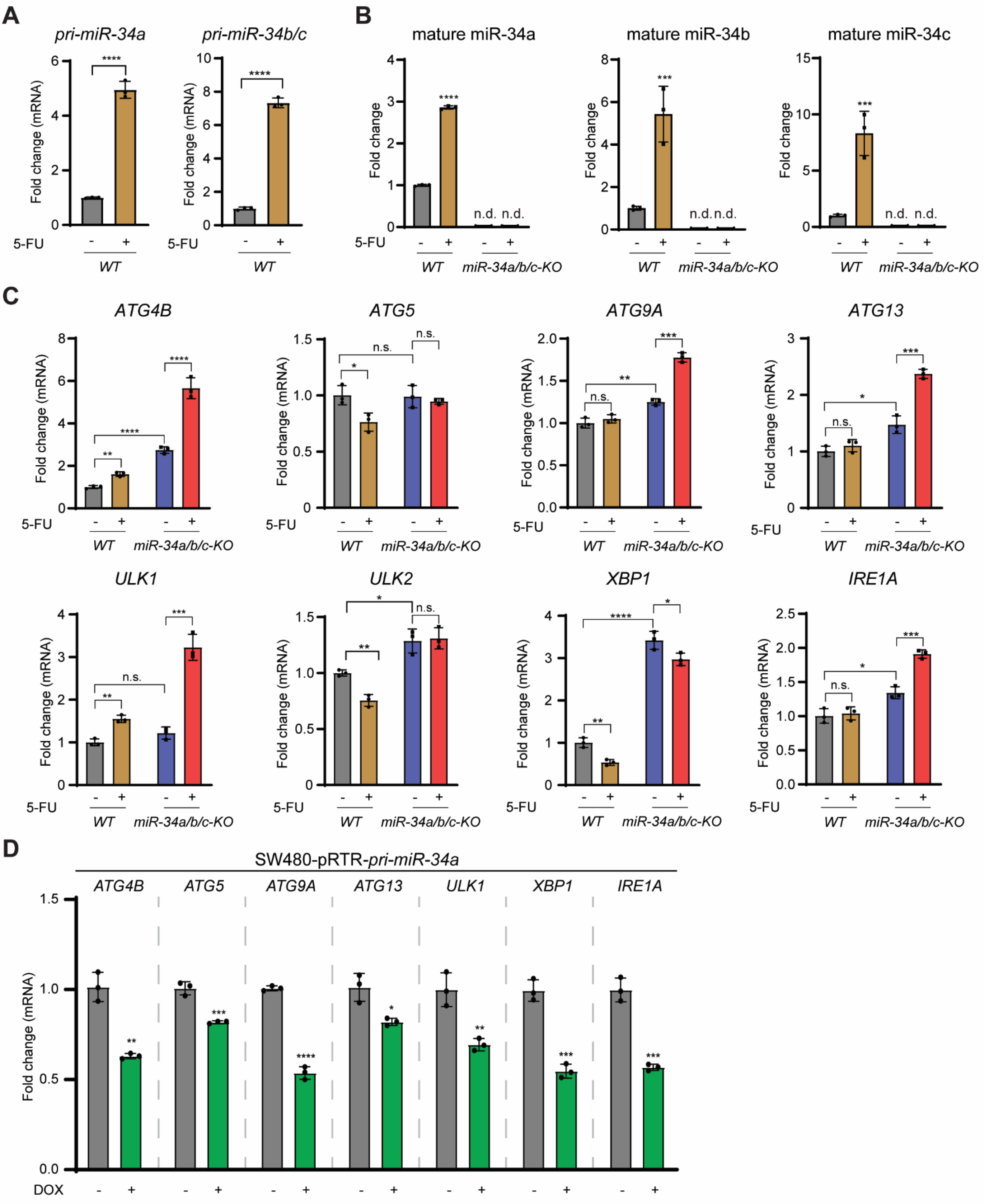

Figure S6. Differential expression of autophagy-related miR-34 targets after deletion of *miR-34a/b/c*. (**A**) qPCR analysis of *pri-miR-34a* and *pri-miR-34b/c* after DMSO or 5-FU treatment for 48 hours in *WT* HCT116 cells. (**B**) qPCR analysis of mature miR-34 in *WT* or *miR-34a/b/c-KO* HCT116 cells after treatment of DMSO or 5-FU for 48 hours. (**C**) qPCR analysis of selected autophagy-related miR-34 target mRNAs. (**D**) qPCR analysis of selected autophagy-related miR-34 target mRNAs after ectopic expression of *pri-miR-34a* from an episomal pRTR vector in SW480 cells by addition of doxycycline for 48 hours. n.d. indicates not detected. Results are presented as the mean +/− SD (n = 3) for **A-D** with *: $p < 0.05$, **: $p < 0.01$, ***: $p < 0.001$, ****: $p < 0.0001$, n.s.: no significance.

Figure S6

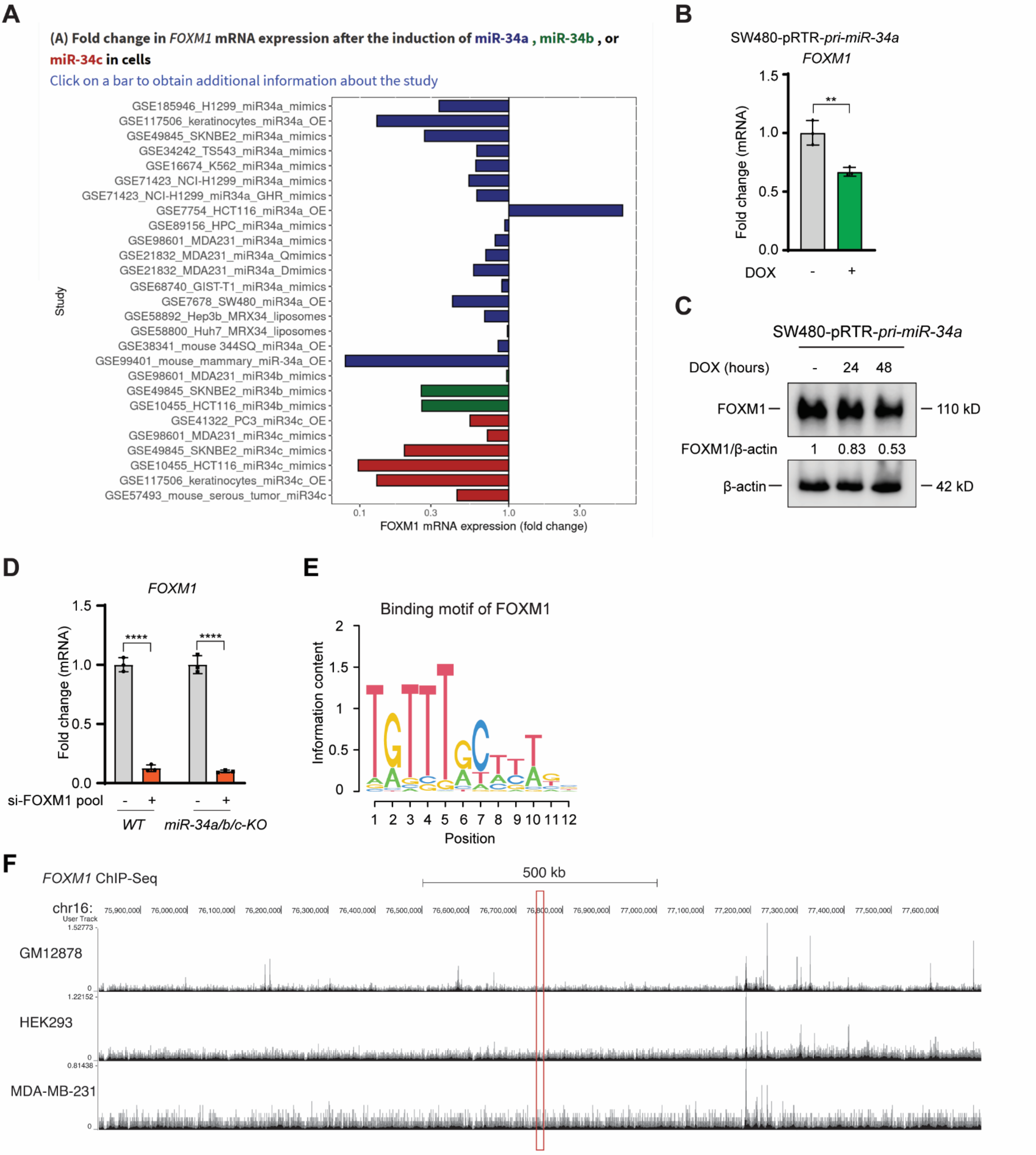

Figure S7. (**A**) Results from the METAmiR34TARGET website showing the fold change of *FOXM1* after ectopic expression of miR-34a/b/c in various cell lines. (**B**) qPCR analysis of *FOXM1* after ectopic expression of *pri-miR-34a* from an episomal pRTR vector in SW480 cells by addition of doxycycline for 48 hours. (**C**) Immunoblotting analysis of FOXM1 protein after ectopic expression of *pri-miR-34a* from an episomal pRTR vector in SW480 cells. (**D**) qPCR analysis of *FOXM1* after transfection of HCT116 cells with 10 nM si-FOXM1 pools for 48 hours. (**E**) Sequence logo showing the FOXM1 binding motif from the HOCOMOCO v11 database. (**F**) Cistrome Data Browser representation of *FOXM1* ChIP-Seq profiles at the genomic region of *16q22*. The region amplified by the qChIP primers used here (see Table S4) is indicated with a red rectangle. Results are presented as the mean +/− SD (n = 3) for **B** with **: $p < 0.01$ and for **D** with ****: $p < 0.0001$.

Figure S7

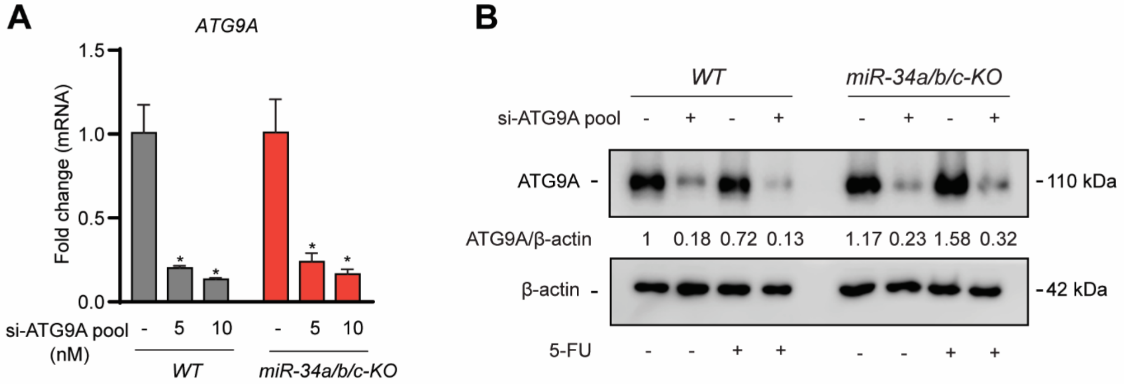

Figure S8. Analysis of si-ATG9A pools efficiency. (**A**) qPCR analysis of *ATG9A* after HCT116 cells were transfected with the indicated concentration of si-ATG9A pools for 48 hours. (**B**) Immunoblotting analysis of ATG9A protein after HCT116 cells were transfected with 10 nM si-ATG9A pools for 48 hours. Results are presented as the mean +/− SD (n = 3) for **A** with *: $p < 0.05$.

Figure S8

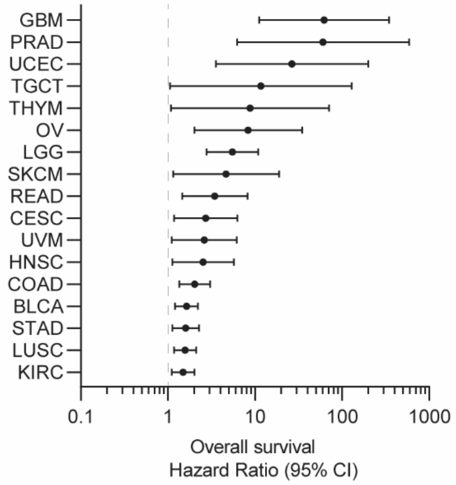

Fig. S9. Cox regression model analysis of the associations between *ΔmiR-34*_Up signature score with overall patient survival in the indicated TCGA pan-cancer patient cohorts.

Figure S9

Figure S10: Original blots.

Uncropped gel of Figure 1F

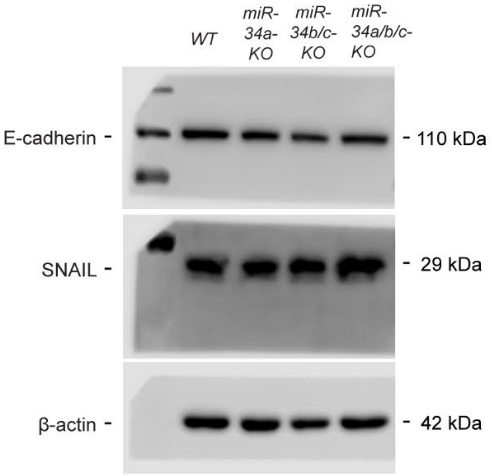

Uncropped gel of Figure 2E

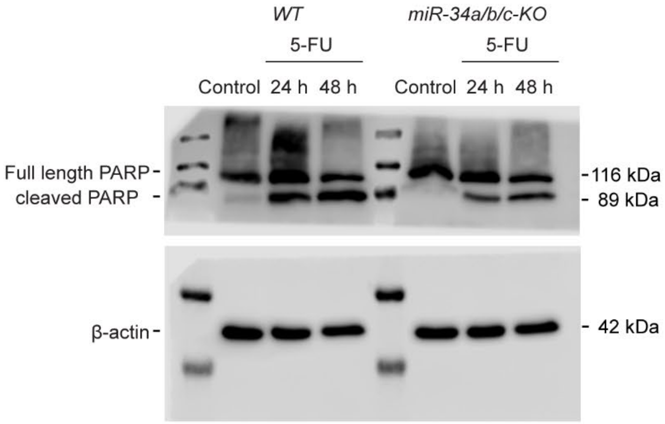

Uncropped gel of Figure 2G

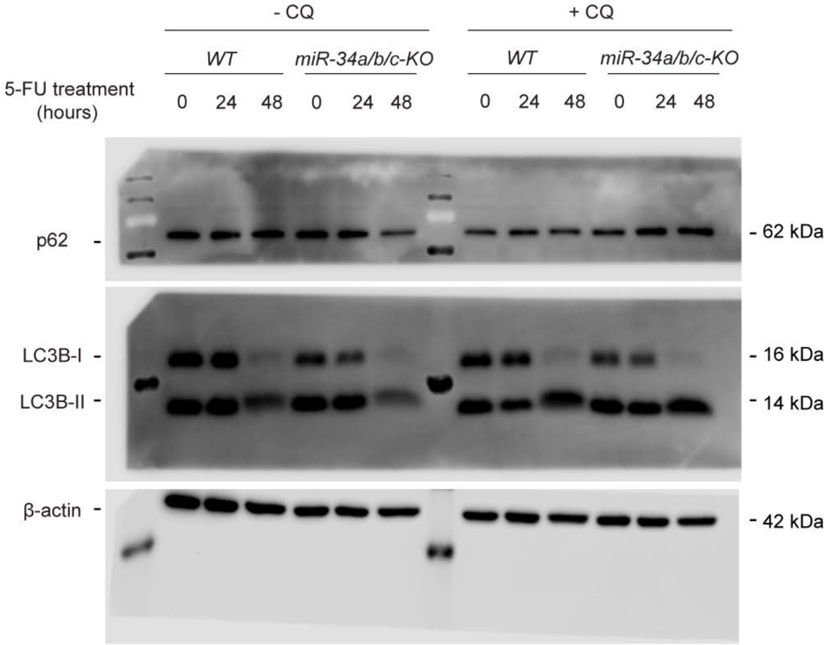

Uncropped gel of Figure 2I left panel

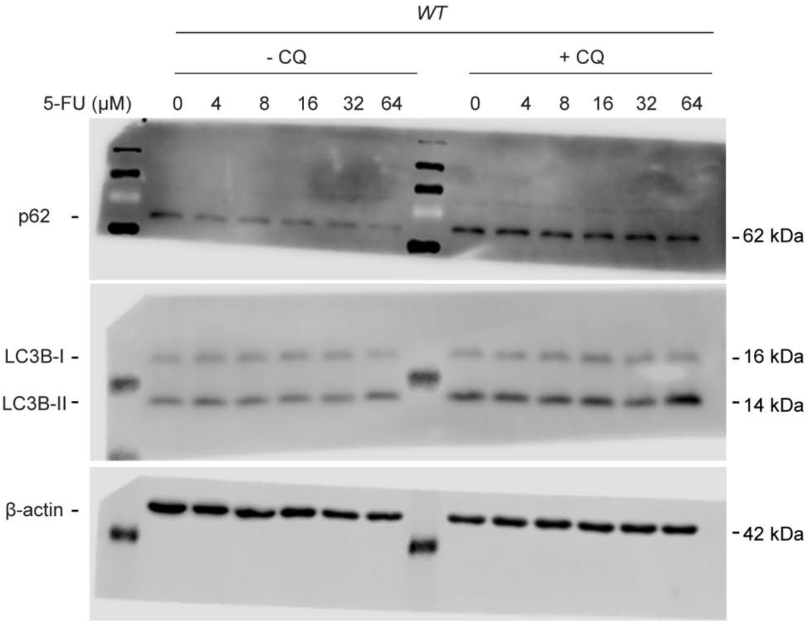

Figure S10

Uncropped gel of Figure 2I right panel

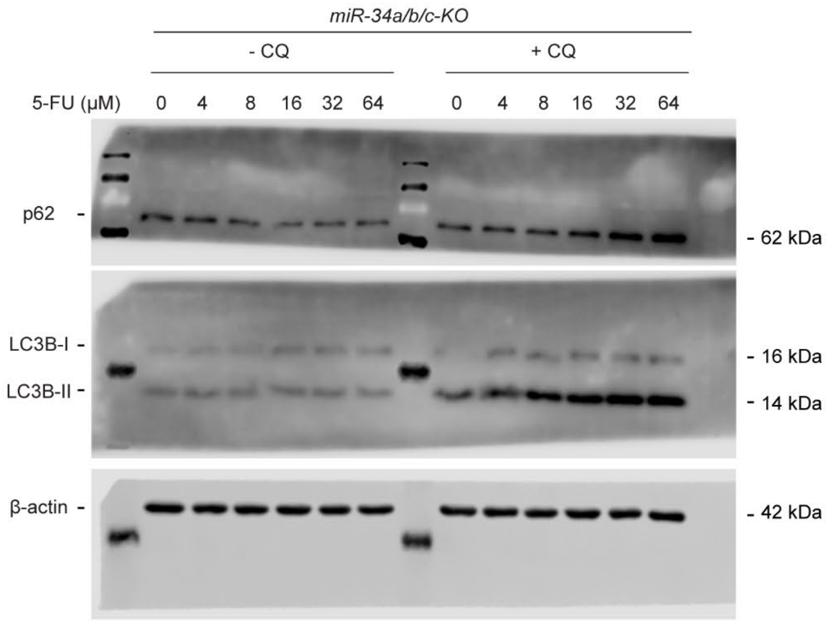

Uncropped gel of Figure 3A

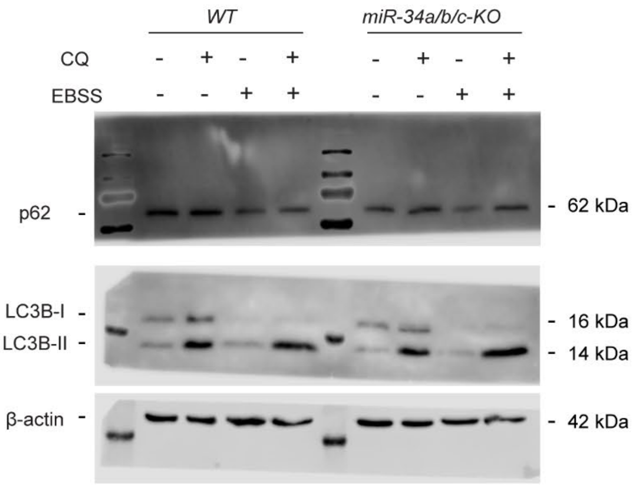

Uncropped gel of Figure 3C

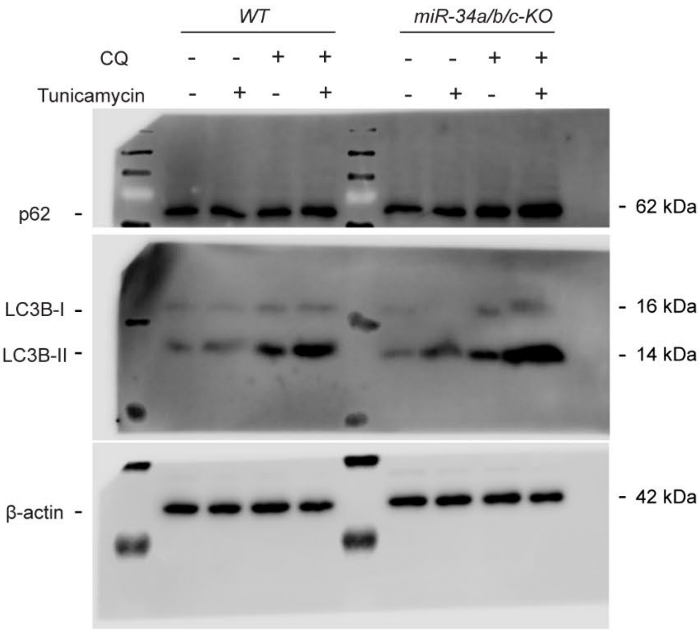

Uncropped gel of Figure 3E

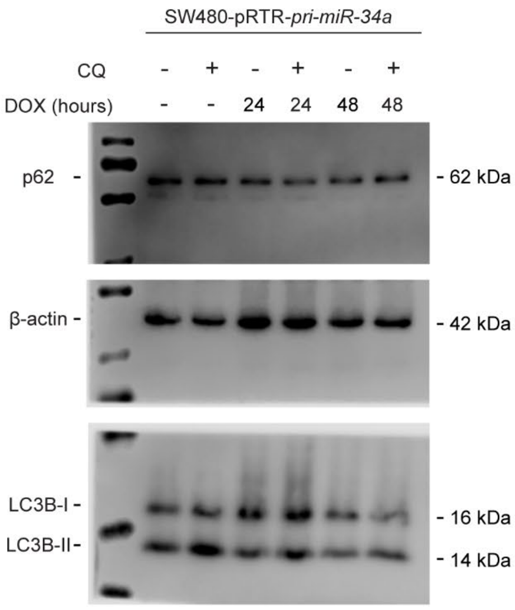

Figure S10

Uncropped gel of Figure 6C

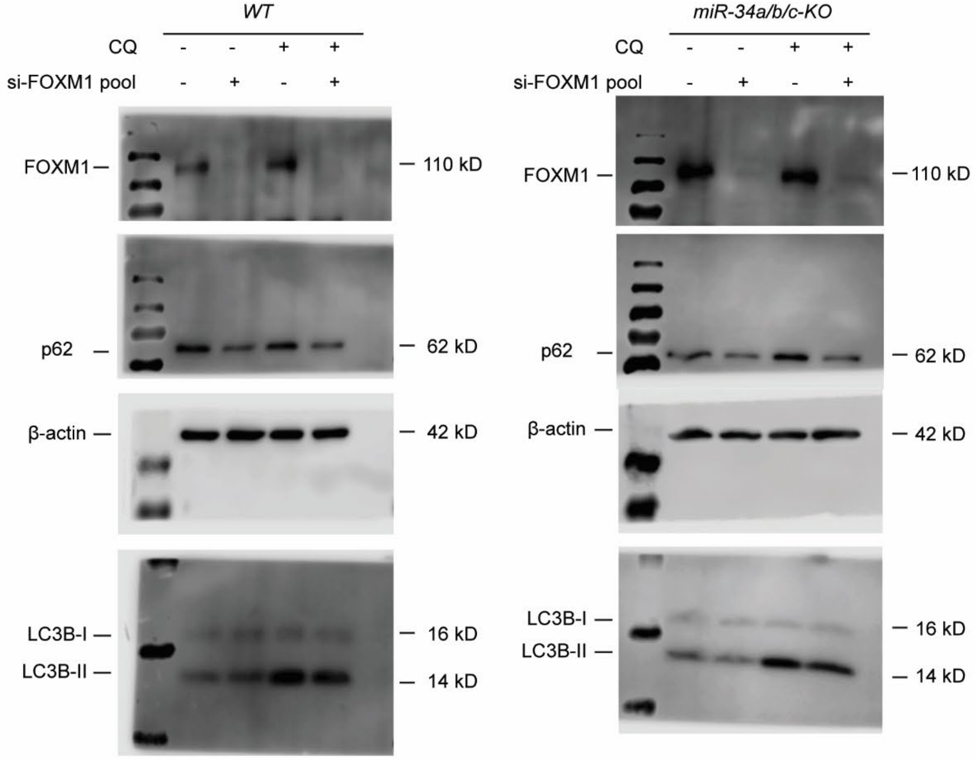

Uncropped gel of Figure 6E

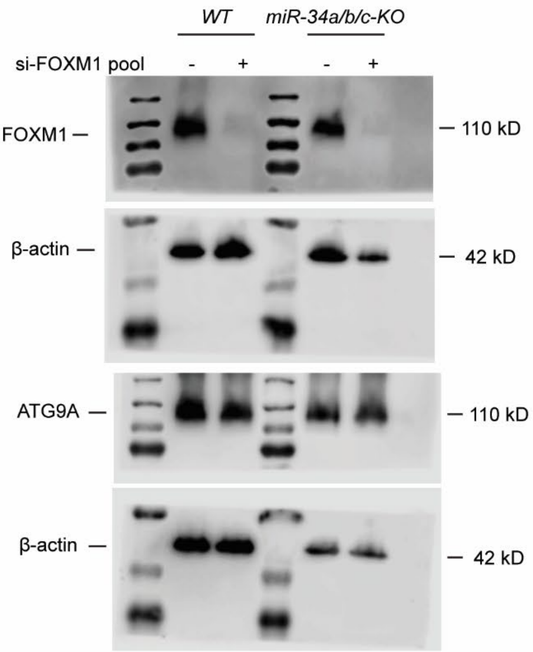

Uncropped gel of Figure 6J

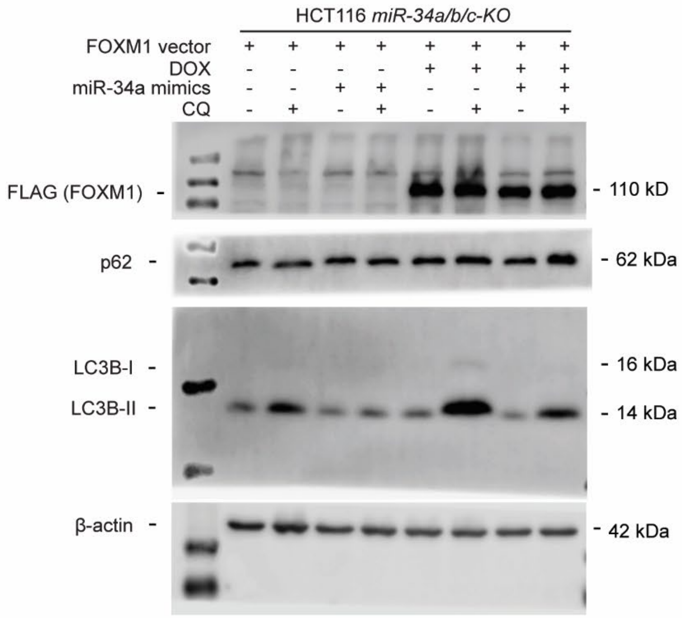

Figure S10

Uncropped gel of Figure 7A left panel

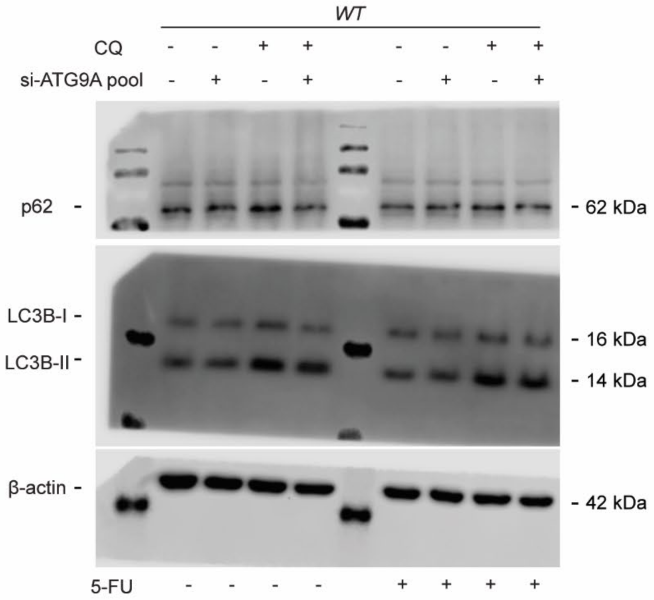

Uncropped gel of Figure 7A right panel

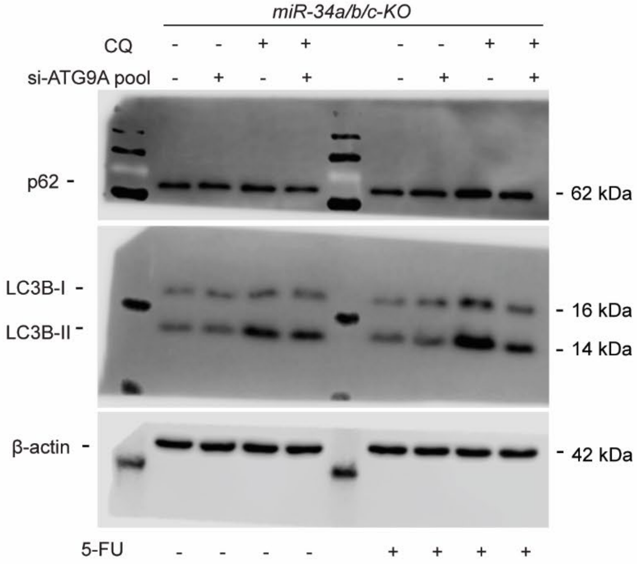

Uncropped gel of Figure 7C

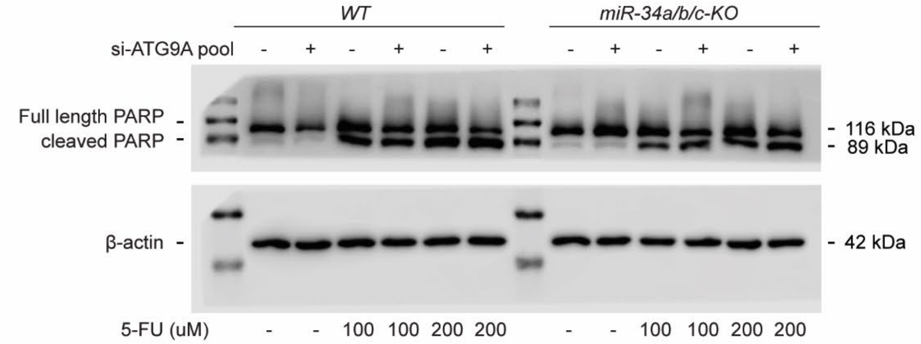

Uncropped gel of Figure S5B

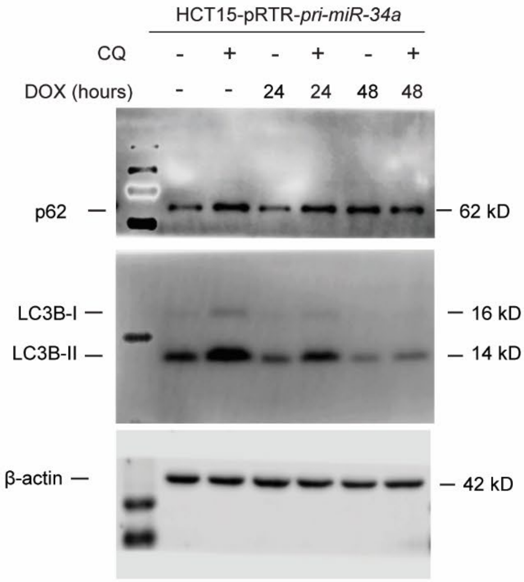

Uncropped gel of Figure S5C

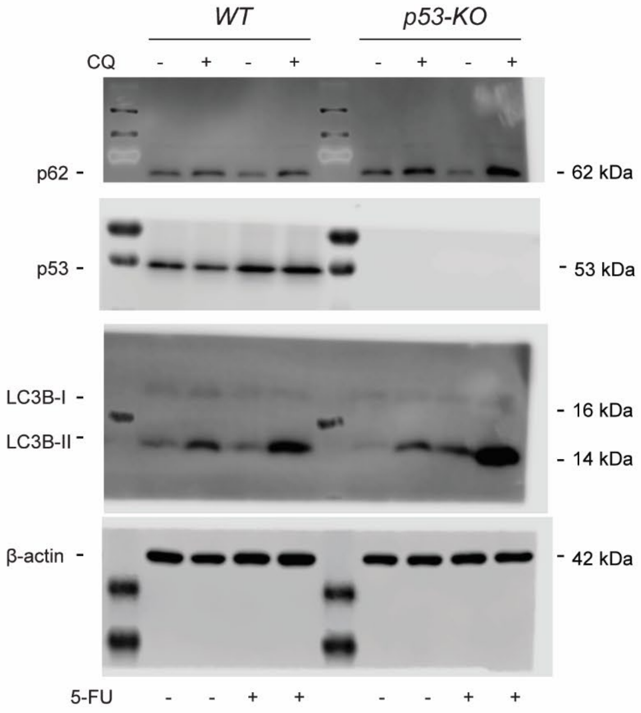

Figure S10

Uncropped gel of Figure S7C

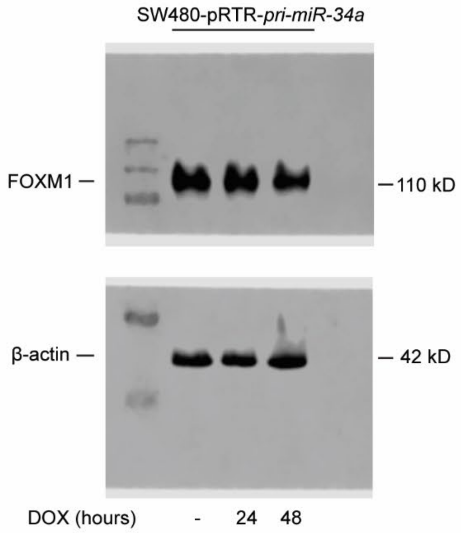

Uncropped gel of Figure S8B

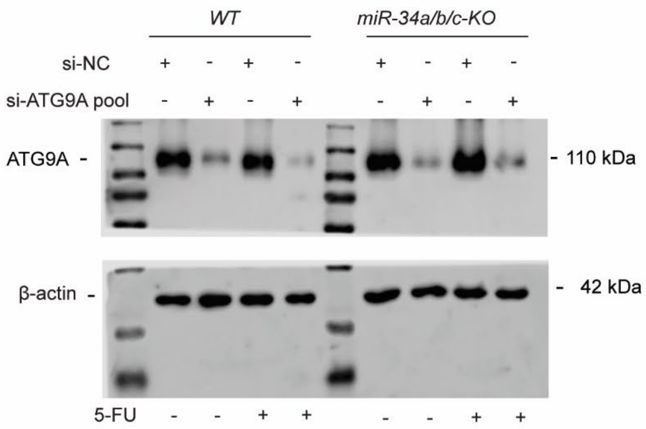

Figure S10

Table S1. Sequence information for guide RNAs used for *miR-34a/b/c* deletion

	Sequence information (5’-3’)
miR-34a guide RNA 1 forward	CACCGGCCGGTCCACGGCATCCGGA
miR-34a guide RNA 1 reverse	AAACTCCGGATGCCGTGGACCGGCC
miR-34a guide RNA 2 forward	CACCGCTAGAAGTGCTGCACGTTGT
miR-34a guide RNA 2 reverse	AAACACAACGTGCAGCACTTCTAGC
miR-34b/c guide RNA 1 forward	CACCGCCTCGGACCCCATTTCACCG
miR-34b/c guide RNA 1 reverse	AAACCGGTGAAATGGGGTCCGAGGC
miR-34b/c guide RNA 2 forward	CACCGGTGCATCATCAATGTGCGTG
miR-34b/c guide RNA 2 reverse	AAACCACGCACATTGATGATGCACC

Table S2. Sequence information for genotyping primers used for selecting *miR-34a/b/c* homozygous deletion

	Sequence information (5’-3’)
miR-34a genotyping primer forward	GTTTTGAACTTCTCGCCTCA
miR-34a genotyping primer reverse 1	CACAACAACCAGCTAAGACACT
miR-34a genotyping primer reverse 2	TACTATTCTCCCTACGTGCAA
miR-34b/c genotyping primer forward	GAACTGAAGCCTGGCGTGAAG
miR-34b/c genotyping primer reverse 1	TTTTGCTTTCCTGGCATGAGAC
miR-34b/c genotyping primer reverse 2	ATGGCAGGAACAACTTAACCAC

Table S3. Oligonucleotides used for qPCR

mRNA	Forward (5’-3’)	Reverse (5’-3’)
CCNB1	GACCTGTGTCAGGCTTTCTCTG	GGTATTTTGGTCTGACTGCTTGC
FOXM1	TCTGCCAATGGCAAGGTCTCCT	CTGGATTCGGTCGTTTCTGCTG
ATG5	GCAGATGGACAGTTGCACACAC	GAGGTGTTTCCAACATTGGCTCA
ULK2	TCCACGGAGTTCTGACTGGTTC	GACGAGTAACCAAGGCTAACAGG
pri-miR-34a	CGTCACCTCTTAGGCTTGGA	CATTGGTGTCGTTGTGCTCT
pri-miR-34b/c	GAGCTGCCTGTGCATCATC	GGATGAAATCAGCATTTTCCA
SQSTM1/p62	CCCTACAGATGCCAGAATCCG	GCCTTCATCAGAGAAGCCCAT
ATG13	AGATGACATTCTTCCGATGGAC	TCCACAAAGGCATCAAACTCG
E-cadherin	CCCGGGACAACGTTTATTAC	GCTGGCTCAAGTCAAAGTCC
VIM	TACAGGAAGCTGCTGGAAGG	ACCAGAGGGAGTGAATCCAG
SNAIL	GCACATCCGAAGCCACAC	GGAGAAGGTCCGAGCACAC
XBP1	GCTGAGTCCGCAGCAGGTG	GCTGGCAGGCTCTGGGGAAG
IRE1A	CGGGAGAACATCACTGTCCC	CCCGGTAGTGGTGCTTCTTA
ATG4B	ATGACTTCAATGATTGGTGCC	AGAAGAATCTGGACTTGGCAG
ATG9A	TCCTCTTTGTGGTTGCCTTC	AGTGACCTTGACGGGTTCAG
ULK1	TCATCTTCAGCCACGCTGT	CACGGTGCTGGAACATCTC

Table S4. Oligonucleotides used for qChIP

Gene	Forward (5’-3’)	Reverse (5’-3’)
CCNB1	CGCGATCGCCCTGGAAACGCA	CCCAGCAGAAACCAACAGCCGT
ATG9A	GTAACGGAGGAAGGGGCG	GCAGCTCCCAACAGCGGACAACC
16q22	CTACTCACTTATCCATCCAGGCTAC	ATTTCACACACTCAGACATCACAG
SQSTM1/p62	CTCAGGGGACTCACGGTGA	GTGAGTCAGCGTCCCATGAC

Table S5. List of antibodies

Epitope	Species	Catalog No.	Company	Use	Dilution	Source
Primary antibodies						
β-actin	Human	# A2066	Sigma-Aldrich	WB	1:1000	rabbit
FOXM1	Human	# 20459	Cell Signaling	WB	1:1000	rabbit
ATG9A	Human	# 13509	Cell Signaling	WB	1:1000	rabbit
PARP	Human	# 9542	Cell Signaling	WB	1:1000	rabbit
LC3B	Human	# 2775	Cell Signaling	WB	1:1000	rabbit
SQSTM1/p62	Human	# 88588	Cell Signaling	WB	1:1000	mouse
SNAIL	Human	# 3879	Cell Signaling	WB	1:1000	rabbit
E-cadherin	Human	# 33-4000	Invitrogen	WB	1:1000	mouse
P53	Human	# sc-126	Santa Cruz	WB	1:1000	mouse
Secondary antibodies						
Anti-mouse HRP	N.A.	# W4021	Promega	WB	1:10,000	goat
Anti-rabbit HRP	N.A.	# A0545	Sigma-Aldrich	WB	1:10,000	goat

Table S6. **Oligonucleotides used for reporter plasmids**

Oligo	Forward (5'-3')	Reverse (5'-3')
FOXM1 3'-UTR	AAAGAATTCAGCCCTGCCCTTGCCCCTGTGC	AAAACTAGTGCATGTCCACCTTCGCTTTTATTGAG
miR-34a/b/c-5p ΔSMS	CTTAGATCATTATCCAGAGGAAGGTGGGTAGGATG	CATCCTACCCACCTTCCTCTGGATAATGATCTAAG

Table S7. List of mRNAs significantly regulated in HCT116 *miR-34a/b/c-KO* cells when compared to *WT* cells.

Significantly upregulated mRNAs			Significantly downregulated mRNAs		
Gene symbol	Log_2 fold change	padj	Gene symbol	Log_2 fold change	padj
TNFSF18	4.339347454	3.6013E-250	*LINC01405*	-3.425416755	7.72945E-70
SPARC	3.228710284	1.8595E-54	*PRXL2A*	-2.838194736	6.40495E-47
ARL4C	3.227317679	0	*PROM2*	-2.719025795	2.2636E-99
DCLK1	3.157889129	1.25303E-74	*KRT20*	-2.29089276	4.2508E-28
GLIS3	3.065755484	5.99162E-48	*MAL2*	-2.288096133	0
DCBLD2	2.662825494	0	*GALNT3*	-2.206355282	1.75251E-85
ITPKB	2.384275358	3.10931E-44	*MACC1*	-2.138584388	8.9007E-108
VIM	2.359988802	4.53468E-48	*UCA1*	-2.101193192	2.0608E-242
AC159540.2	2.34676407	1.40232E-35	*SQLE*	-1.99992705	1.1569E-221
VCAN	2.328778639	7.8616E-235	*ACSL5*	-1.988307444	3.4415E-292
PELI2	2.23244312	3.28585E-33	*CPLX1*	-1.971139541	2.61176E-57
MIR34AHG	2.186496104	3.39822E-89	*MGLL*	-1.969537955	3.5061E-101
ADAM19	2.18214113	9.2184E-222	*PKIB*	-1.9381272	3.0759E-74
NECTIN3	2.129138114	1.7925E-221	*AC025419.1*	-1.913485746	8.28192E-24
EPB41L4A	2.119745107	4.33764E-42	*INAVA*	-1.897862972	9.86406E-50
FAM189A2	2.054754111	3.45259E-24	*ANXA10*	-1.893565725	4.67882E-44
GPRASP2	2.008988781	2.62536E-35	*LIPG*	-1.893384389	3.68344E-53
MAMDC2	1.988591768	2.48188E-30	*IFITM1*	-1.855880253	1.53495E-44
SLC16A6	1.968564187	4.27611E-64	*FGFBP1*	-1.812869965	1.63279E-39
ABCB1	1.930923623	1.07985E-27	*SEC63*	-1.771615157	4.2216E-111
THBS1	1.919675684	6.5602E-145	*AC016831.1*	-1.746735381	2.09265E-22
CYB5A	1.896202555	3.12931E-33	*AC016074.2*	-1.716349386	8.21974E-17
CDK6	1.875119472	7.4918E-129	*MAT1A*	-1.695543244	1.08417E-20
FBXO10	1.830023801	2.13162E-26	*RGS2*	-1.643914052	7.1141E-120
DNAJB9	1.829378486	1.9071E-146	*BST2*	-1.594567434	3.48061E-30
LIMS2	1.809630494	8.29734E-13	*CD33*	-1.578041646	7.42376E-14
CPEB2	1.799538637	3.73255E-20	*EPN3*	-1.546268271	2.94203E-65
SNTB1	1.787185823	9.32231E-47	*EGR3*	-1.527042216	3.69804E-07
PBX1	1.786330961	2.17635E-41	*ANKRD22*	-1.526440394	2.93886E-20
LINC00342	1.780223703	4.00811E-26	*EREG*	-1.519346141	1.3091E-100
NR2F1	1.778351791	5.0791E-161	*TMEM200A*	-1.502077668	5.90773E-83
SELENOP	1.759684301	4.41574E-29	*MATR3_2*	-1.501183652	0.002195474
SEC24D	1.743093773	9.9743E-149	*AC099489.1*	-1.490796943	2.10632E-16
ABCA1	1.740350067	4.69287E-26	*FAM122B*	-1.471222128	1.1464E-121
LAMA4	1.734279719	3.65897E-27	*USP2-AS1*	-1.451237	1.32183E-10
BHLHA15	1.724703491	1.78795E-10	*LRRC6*	-1.450015593	2.02362E-17
D2HGDH	1.701696036	2.02659E-81	*CKMT1B*	-1.449499776	2.69863E-60
DNER	1.695198009	8.66777E-18	*SOX8*	-1.422098046	2.66751E-27
ZEB1	1.693121566	1.94134E-18	*VSNL1*	-1.421364513	4.27088E-61
CTF1	1.682229192	3.4711E-11	*PADI3*	-1.414705066	2.56856E-19
TSC22D3	1.682129561	2.89747E-53	*ASAP1*	-1.395209536	6.7128E-108
SLC2A1	1.681457531	3.8444E-157	*DES*	-1.382963807	1.13103E-14
LOXL4	1.675004419	8.87237E-39	*RASD2*	-1.382741758	3.37273E-17
SH3BP4	1.663539621	5.3587E-159	*HAS3*	-1.369290052	1.23429E-98
ZNF30	1.650742373	2.45813E-10	*DUSP9*	-1.366558908	1.5783E-26
PLXNB3	1.649046958	1.01673E-29	*RASGRF1*	-1.359960319	2.00334E-13
CPA4	1.638334967	1.1798E-118	*NDRG1*	-1.356798933	2.5807E-49
SNORC	1.629227977	1.27252E-12	*RPS6KA5*	-1.349797183	1.99823E-61
ITGB8	1.619600045	1.15701E-59	*FAM156B*	-1.349124836	0.009554429
GFY	1.618258289	7.66398E-23	*HSD17B6*	-1.346379752	4.17064E-08
CEMIP2	1.603755017	4.7935E-166	*PLEKHA6*	-1.344431373	1.45137E-25
MIR22HG	1.600804888	2.24145E-18	*MED30*	-1.333495975	2.63376E-34
AJM1	1.598858162	1.90979E-35	*LRATD2*	-1.331562529	3.62457E-90
ARHGAP24	1.590609066	1.84899E-28	*LRG1*	-1.316461852	9.69307E-10
AF117829.1	1.585679751	1.42675E-24	*DTX4*	-1.309738135	4.68321E-31
RUNX1	1.569783298	8.4981E-142	*OR51B5*	-1.309595204	5.73233E-54

GPC1	1.566567386	7.6631E-109	*CYP4F2*	-1.288973638	3.97669E-06
AC092683.1	1.558952256	2.64195E-11	*MBOAT1*	-1.288082616	8.20711E-43
GDF15	1.536792101	4.91558E-85	*TERT*	-1.288078613	7.36642E-15
CYP4F3	1.533372269	1.30881E-09	*CR2*	-1.2876264	2.84882E-20
APLF	1.530922625	1.99074E-08	*HCN2*	-1.269343464	4.72229E-15
SLC25A21	1.525480397	8.1099E-09	*HSPH1*	-1.266340157	5.321E-109
CDKN1A	1.480251828	2.5163E-200	*LAMA2*	-1.265199934	2.56764E-16
NUCB2	1.467554575	2.2201E-113	*CLDN3*	-1.26432746	1.08079E-32
HDAC9	1.464697612	6.93157E-18	*ARL14EPL*	-1.260237815	3.91297E-28
EFNA5	1.456181739	1.22164E-14	*LINC00431*	-1.245976482	6.42281E-06
AC133644.3	1.454085911	2.55065E-13	*EGR1*	-1.243946725	3.46531E-42
ESRRB	1.444913425	2.59402E-13	*DSCC1*	-1.237127347	5.67434E-72
TP53I3	1.444783265	2.70212E-79	*ANXA9*	-1.227354952	5.07421E-07
CC2D1B	1.443924931	2.9983E-77	*SH3BGRL2*	-1.20854454	1.30912E-28
ABHD8	1.442709821	2.01198E-17	*PLCH2*	-1.199134856	1.01524E-06
EMC3	1.441814084	6.03933E-32	*KRTAP2-3*	-1.195182689	5.30696E-18
BTBD11	1.436358516	1.41781E-45	*IQANK1*	-1.194088906	8.54631E-48
AC118344.4	1.435515457	3.9257E-08	*FHAD1*	-1.193594334	1.65203E-30
RNF43	1.431512933	1.06338E-86	*PTK2*	-1.191499852	4.1736E-137
TP53INP1	1.427879101	2.72031E-81	*ANKRD37*	-1.178990132	3.80035E-07
PRSS23	1.427374843	1.22634E-52	*CU633906.2*	-1.171427627	0.004121707
CDKN2B	1.424088977	3.52188E-49	*TOP1MT*	-1.170864575	1.9799E-87
PNPLA7	1.413018304	6.52927E-11	*CASC8*	-1.169383611	2.16135E-06
SLC44A5	1.403448122	5.50055E-17	*CLMP*	-1.168231118	6.87979E-11
LPAR1	1.398034246	7.05586E-59	*PTGS1*	-1.168071003	2.93256E-13
CRELD2	1.393665723	2.6605E-107	*CRYBG2*	-1.157552734	1.39935E-11
RASSF8-AS1	1.388583342	3.22283E-10	*CPM*	-1.143750381	5.99166E-08
USP40	1.388548779	1.94571E-80	*ARRDC4*	-1.143128439	1.14622E-28
TSPAN7	1.385999575	1.54885E-09	*NR2F2*	-1.141173256	8.21958E-80
CREB3L2	1.385655326	1.3859E-102	*GNG4*	-1.138414013	1.6276E-14
SCART1	1.378883465	5.28402E-10	*PLAT*	-1.138213041	6.49968E-30
CCDC183	1.376063123	3.03309E-06	*DPP4*	-1.138065327	2.30451E-17
LCN12	1.369983679	1.53476E-07	*FDFT1*	-1.132643237	4.3053E-112
AC090114.2	1.3596514	6.23363E-06	*RNF32*	-1.130752198	7.62791E-08
PIP5KL1	1.358736235	1.06424E-30	*ZNF34*	-1.121467997	1.9525E-10
IGFBP6	1.356156259	4.73724E-66	*CKMT1A*	-1.121382903	2.10206E-27
XXYLT1	1.35371626	1.77183E-26	*PRSS3*	-1.118654965	6.56078E-26
EGF	1.353408249	2.42061E-10	*ID2*	-1.113649783	7.43941E-39
RAB17	1.352873058	1.18383E-51	*GABRE*	-1.109923761	1.60123E-06
TRIB2	1.3498719	4.477E-61	*SPNS2*	-1.104618397	1.42965E-17
CEACAM1	1.336159798	1.40298E-21	*ALPK3*	-1.101669723	8.83126E-12
HOMER2	1.328234878	5.33286E-06	*ARHGAP26*	-1.100477712	4.16489E-67
DUSP28	1.321075914	3.22776E-11	*WDYHV1*	-1.097465723	3.4928E-28
PER2	1.320934976	1.56131E-51	*HAUS7*	-1.096288246	8.00492E-15
ST6GALNAC4	1.318995512	3.64839E-56	*SLC5A5*	-1.094836651	4.05694E-05
COL4A3	1.318471098	4.61857E-24	*AC156455.1*	-1.094313561	2.18679E-09
MSX2	1.317089403	6.69996E-41	*WASF3*	-1.08272578	5.89947E-14
TINAGL1	1.316366639	3.06832E-85	*ZCCHC12*	-1.081937595	1.59313E-05
APOBEC3D	1.306956507	5.71456E-08	*AL135905.2*	-1.081594156	5.7176E-61
SERINC2	1.304739704	2.00687E-91	*KSR2*	-1.078692503	1.35176E-09
AL049840.2	1.302803174	3.44289E-06	*AC103718.1*	-1.077558878	8.05356E-06
KCNK13	1.298664436	1.49827E-11	*AC000120.4*	-1.074285796	0.005951078
ZSWIM5	1.298338423	1.67583E-06	*TMEM51-AS1*	-1.065819782	1.34151E-11
DENND5B	1.290499235	1.96519E-68	*GCOM1*	-1.064165437	2.20047E-12
MTND1P23	1.28921812	0.029839311	*PARP9*	-1.06097859	3.91987E-41
SMPD1	1.277660253	6.04976E-19	*PRKCQ*	-1.050641611	7.61062E-05
INPP4B	1.274322145	6.69551E-72	*ATAD2*	-1.045154444	3.91996E-46
SLC4A8	1.272138414	6.77673E-09	*GJB4*	-1.04487426	5.21686E-07
HSPG2	1.270918569	5.26513E-21	*TRIB1*	-1.043867759	1.65872E-93

RAMP1	1.269098832	3.52067E-25	*AC008771.1*	-1.043220805	2.54661E-08
TRIML2	1.268256149	5.84525E-15	*IL1RAP*	-1.040116729	1.02321E-09
AC022400.9	1.266997548	9.17697E-09	*GATA2*	-1.034845675	5.11436E-25
SUSD2	1.258584803	9.8875E-52	*WNK4*	-1.030222691	3.87053E-08
CPQ	1.254595673	1.07105E-07	*AOC3*	-1.029323326	1.81038E-09
PPIEL	1.251509796	2.18611E-16	*MOSPD1*	-1.026575916	1.70633E-53
RETREG1	1.248209291	1.85984E-27	*IQCD*	-1.006864356	1.40818E-08
SLC2A6	1.243920539	2.57842E-32	*PDE2A*	-1.00579607	6.02901E-18
ZNF738	1.243856176	3.34017E-08	*NUDCD1*	-1.00491147	9.83455E-41
TIGD1	1.241671234	8.51259E-08	*NSMCE2*	-1.000891015	5.46759E-22
CUEDC1	1.225305701	4.16791E-68	*GASK1B*	-0.999318354	3.67626E-07
MILR1	1.224093855	5.76648E-07	*MYH15*	-0.99728868	7.9069E-20
AL157935.3	1.222315457	1.48978E-23	*SLC25A32*	-0.995573406	7.21509E-60
GLT8D2	1.217384253	1.14132E-15	*CCDC122*	-0.99411676	3.20707E-06
COPS9	1.214488644	3.68273E-22	*MAFA*	-0.99176966	7.33894E-05
MAN1B1	1.213706327	6.6005E-121	*MYZAP*	-0.985521561	1.15528E-10
COL4A4	1.208986526	1.21985E-41	*NOS1AP*	-0.985445433	1.91435E-09
AC024909.1	1.208855389	1.50034E-05	*SLC8A2*	-0.985353644	0.000566824
CLIC3	1.205430986	1.9768E-15	*ATP8B1*	-0.982684872	1.44523E-43
PSMG3-AS1	1.203957232	6.47625E-07	*DTX3L*	-0.979991703	1.93658E-28
ZNF816	1.203527931	7.05517E-10	*AGO2*	-0.976873874	1.16166E-41
ZNF596	1.20235186	3.21804E-12	*IDI1*	-0.975334987	4.42302E-40
SNHG19	1.199267279	2.70052E-05	*LYNX1*	-0.972332651	3.94286E-10
PHPT1	1.197558785	3.51815E-49	*MAP3K8*	-0.971968211	5.05712E-05
HDLBP	1.197431032	1.952E-146	*RECQL4*	-0.96974489	5.72484E-37
FRMD3	1.194047107	4.91673E-07	*ID3*	-0.962929372	3.39358E-37
AL109976.1	1.193052345	1.10834E-06	*CABP1*	-0.959063219	0.000925878
KRT81	1.192186916	8.75568E-09	*OVOL1*	-0.958509771	2.90193E-21
AASS	1.190511076	1.71215E-17	*C8orf33*	-0.951527711	4.22645E-75
CCDC146	1.189350365	7.54102E-12	*MYEOV*	-0.945718358	1.8335E-50
P3H1	1.187085925	3.66232E-38	*AC005336.1*	-0.942406045	8.00422E-07
PDE5A	1.185059059	4.61328E-21	*MAPK15*	-0.937006225	2.34471E-17
SURF4	1.183365314	3.368E-131	*UTP23*	-0.934716106	4.53805E-36
CEL	1.182805964	1.47253E-13	*LNCAROD*	-0.933811013	2.23637E-18
P4HB	1.181806019	2.6483E-102	*SYT3*	-0.933625858	1.459E-07
AL359715.1	1.178806755	6.58731E-07	*SLC37A2*	-0.932149303	1.3518E-15
SATB1	1.172619999	6.22686E-11	*CASC19*	-0.932072979	2.72438E-22
MIR1915HG	1.170466928	2.54479E-20	*PPARG*	-0.930803337	9.2222E-34
GRIN2C	1.169831995	3.79338E-11	*ZNF827*	-0.930114234	1.00895E-33
SOBP	1.1639309	2.73609E-14	*FSD1*	-0.928066852	9.72296E-16
ZDHHC12	1.159321711	1.98292E-30	*CTSH*	-0.923814429	1.76855E-22
NTNG2	1.157761339	3.17634E-09	*FRMD4B*	-0.923746015	1.33116E-05
IQCN	1.157305367	2.64325E-06	*LINC02495*	-0.923665467	2.81623E-05
INSYN1	1.156880073	2.53965E-05	*MYB*	-0.92334152	1.56358E-12
ANKMY1	1.156781722	3.44615E-19	*GRHL2*	-0.921763162	2.68767E-25
TTLL11	1.156510679	0.000288186	*NDUFC2*	-0.920837961	2.88892E-34
SEC11C	1.156227489	2.23672E-56	*MAPK8IP2*	-0.920251918	9.20692E-11
GLDN	1.146167084	8.14391E-10	*MTBP*	-0.916855329	1.09788E-18
SLC35F3	1.142941334	1.54926E-30	*ZNF695*	-0.916652753	4.22723E-09
TCTA	1.142308298	5.58162E-29	*TRABD2A*	-0.914835973	1.21197E-07
INPP5D	1.14021015	8.26169E-12	*PARP10*	-0.911928137	1.51929E-06
COL12A1	1.139934809	8.48455E-70	*SLC39A4*	-0.910931356	7.85383E-22
APH1B	1.137802487	1.27084E-16	*TATDN1*	-0.909230113	9.39589E-27
ILKAP	1.131157803	1.08083E-46	*C1orf226*	-0.908914076	1.47058E-26
CACNB2	1.130988335	0.000273304	*EIF3E*	-0.908085081	2.36652E-52
MRC2	1.130193746	5.66026E-33	*P2RX5*	-0.908028166	2.87876E-09
PDIA4	1.130090637	1.1696E-154	*MEF2C*	-0.907268858	4.17331E-14
FICD	1.126327354	4.97296E-17	*GOLT1A*	-0.906238447	6.77885E-05
GIGYF2	1.124462075	4.98728E-67	*CCDC153*	-0.901874018	9.04855E-05

CERCAM	1.124220989	1.05202E-61	*AC083843.3*	-0.899484599	2.56429E-07
ANKRD44	1.122017037	1.70709E-07	*CFAP57*	-0.897669804	0.001561467
STK25	1.121800836	9.67545E-82	*ATG12*	-0.897253801	1.20605E-48
ING5	1.118412709	4.91711E-51	*FA2H*	-0.896308175	4.72975E-15
CYS1	1.11783723	1.09699E-14	*DPYSL3*	-0.892483802	4.62548E-27
SHC2	1.115186965	1.60419E-06	*TMOD1*	-0.892392921	3.69005E-24
ADAMTS13	1.113793086	3.53604E-26	*ZBED2*	-0.892035373	7.30007E-11
PLXNA2	1.112900454	1.90873E-05	*S100A14*	-0.890885818	6.0451E-54
PLK2	1.109091757	2.74263E-85	*PIM1*	-0.889232162	6.62179E-20
NACC2	1.106061653	2.32114E-52	*FOS*	-0.887394583	1.19765E-09
ATG4B	1.104533874	1.12407E-54	*AC011912.1*	-0.886856565	0.001002011
TIAM1	1.103952653	6.09788E-62	*KLK6*	-0.885562836	4.89918E-30
PBXIP1	1.103807006	1.2038E-39	*BOP1*	-0.884573887	7.32456E-32
CDK14	1.100635024	8.05045E-24	*SHANK1*	-0.884497675	0.001255551
AC108860.2	1.097701692	9.33926E-05	*ZNF567*	-0.883084234	2.74858E-08
SEMA3A	1.096014036	3.45991E-42	*ACAA2*	-0.881916584	6.5813E-23
SLC30A4	1.094560485	6.49425E-13	*PLTP*	-0.879851645	2.65236E-05
ADAMTS17	1.092749277	3.69542E-13	*KLF4*	-0.878968664	3.16715E-22
PLD1	1.091786813	8.40056E-33	*LINC-PINT*	-0.876105454	7.2042E-07
SEMA3C	1.091597046	1.46423E-22	*SECTM1*	-0.873755915	3.53632E-15
KIAA1324L	1.089512613	1.23344E-48	*HNF4G*	-0.871830237	5.18546E-05
PPP1R26-AS1	1.086963635	1.76185E-06	*TRIM34*	-0.871200874	0.010825678
DOK7	1.086336875	1.4005E-06	*WASHC5*	-0.870536806	1.04916E-43
TBX19	1.085220149	1.36819E-06	*DCAF13*	-0.870103721	2.19141E-37
ARL4D	1.083829239	2.64875E-29	*GDA*	-0.870079804	1.61961E-35
PXDN	1.08238246	2.22854E-56	*IRF7*	-0.866790802	4.08326E-14
VWCE	1.079429873	0.000217301	*AC005747.1*	-0.866414281	4.24344E-11
PDGFC	1.078845215	2.65739E-30	*CEROX1*	-0.865640791	3.46271E-12
KRT8P33	1.076543619	0.000228814	*AKAP12*	-0.863760678	1.47542E-71
ATP8B3	1.076301141	1.25874E-26	*LNX1*	-0.861119609	1.09307E-07
ARSG	1.073252951	7.58788E-26	*EML1*	-0.860541467	3.19797E-15
IL6R	1.071354505	1.68012E-20	*BDNF*	-0.859194282	3.51076E-41
CICP14	1.065924851	5.09585E-14	*KLK10*	-0.857423797	2.24662E-19
WWC3	1.061552049	1.49746E-26	*LAMB1*	-0.856858182	5.5497E-56
TTC17	1.056926382	6.89379E-59	*CACNA1G*	-0.856202408	1.39106E-09
MYH3	1.055673994	1.62648E-05	*ATP6V1C2*	-0.852254051	1.80285E-06
ZNF211	1.055472585	2.47734E-10	*KIF24*	-0.851408749	3.21484E-11
SLC22A31	1.055008493	2.10741E-29	*DENND3*	-0.849470562	2.91425E-21
AC112220.2	1.052023203	2.63235E-06	*BNIP3*	-0.849207848	9.67778E-29
SLC3A2	1.05124855	1.80337E-92	*SPHK1*	-0.848988324	2.68961E-08
AF127577.4	1.051084342	0.000880922	*ATP6V1C1*	-0.845995015	8.79339E-40
LINC01271	1.043925122	1.28485E-06	*EFEMP2*	-0.845658441	7.60576E-06
CRAT	1.04326714	1.83574E-36	*MSMO1*	-0.84527714	1.26178E-31
C22orf34	1.042968559	2.63398E-09	*PCLAF*	-0.840325711	1.83874E-44
XYLT1	1.040888061	5.77182E-14	*IFITM3*	-0.837743208	2.23052E-30
TUBBP5	1.040462985	1.60356E-10	*TCERG1*	-0.83734595	1.23076E-42
AC009022.1	1.039208397	3.61E-15	*SYT12*	-0.835987909	2.0203E-09
FOXP2	1.036455418	0.000815801	*PLEKHA4*	-0.83429193	3.01996E-16
TXNDC15	1.030557469	1.21423E-35	*EPB41L4B*	-0.832121772	6.10997E-26
KCNB1	1.029944794	5.51168E-11	*AC092821.2*	-0.831332438	0.001507499
APOBEC3C	1.02908737	8.9118E-98	*AGPAT5*	-0.830608541	1.53614E-31
AC009120.2	1.02763779	0.001370669	*EIF3H*	-0.826340351	1.43162E-50
MTERF4	1.026722864	2.85656E-27	*AC015712.6*	-0.824432642	0.001215112
DNAJC3	1.026516551	4.65535E-62	*UBE2H*	-0.823296884	2.7464E-51
EXD3	1.025648091	1.50667E-17	*FAM49B*	-0.823268149	1.14402E-37
ARHGAP42	1.025267204	9.30476E-37	*RAB27B*	-0.823224482	1.36472E-12
LRIG1	1.022715193	1.24035E-13	*DDX60*	-0.82152783	1.89788E-08
GPM6A	1.02185431	2.2867E-11	*DHCR24*	-0.821011386	5.43459E-50
UNC5A	1.018688336	4.14884E-18	*CYP4F11*	-0.820969871	5.19439E-28

ZNF608	1.016412879	2.44978E-18	*E2F8*	-0.818987884	6.27007E-23
KLRK1	1.015768925	0.000534611	*BMP6*	-0.818745907	0.00051951
AL441992.2	1.014767262	1.07394E-07	*ZNF675*	-0.818366225	0.002897905
LRRN2	1.014687835	9.91453E-15	*INTS13*	-0.817137541	1.80809E-31
MLPH	1.014376975	3.58418E-47	*WDR66*	-0.814402283	0.001215112
CD82	1.014239779	5.9671E-38	*TMEM158*	-0.812977915	1.43303E-08
EMP3	1.013214287	6.1857E-23	*FHL1*	-0.812820739	3.22103E-19
AC018665.1	1.010102851	5.06452E-05	*ZFPM2-AS1*	-0.810966726	1.31695E-06
EIF4E2	1.009101445	8.96876E-56	*RGS17*	-0.810493366	5.45341E-07
FAM102A	1.008834619	9.1238E-51	*KLK8*	-0.809783461	4.14943E-10
MZF1-AS1	1.008007036	2.79503E-05	*ANKRD29*	-0.809190781	1.42056E-08
SERPINE2	1.006721238	2.99647E-50	*AFAP1*	-0.808105584	9.42957E-29
AGAP1	1.006467178	1.12058E-50	*RAD21*	-0.807631395	7.44547E-36
HSPA13	1.005351799	3.17823E-35	*ZMYND15*	-0.806105763	5.37228E-05
SEC61A1	1.004810815	1.8531E-118	*GADD45B*	-0.806084651	1.61626E-19
PLPP5	1.004618611	1.8647E-35	*MNX1*	-0.805970493	8.23163E-08
ENPEP	1.00309285	1.05532E-10	*HMGCR*	-0.804823646	6.46083E-45
MTUS1	1.002234958	6.34485E-71	*C1R*	-0.804768234	2.36781E-05
MYDGF	1.002181337	1.40502E-73	*EIF4EBP1*	-0.803390758	1.28064E-27
FAM78B	1.001751235	0.001307767	*PADI2*	-0.803389333	0.000316073
MIGA2	1.00079235	5.61869E-27	*LINP1*	-0.802731462	1.33103E-10
PNMA2	1.000065971	0.002079476	*F2RL1*	-0.801150519	6.36316E-44
RTCA-AS1	0.999541784	0.00075136	*VWA5A*	-0.800412429	4.54369E-09
RNPEPL1	0.999364547	4.53387E-47	*SYDE2*	-0.799920943	1.97215E-06
HYOU1	0.999347707	6.4368E-120	*AZIN1*	-0.799440265	2.14805E-36
TMED9	0.997636912	1.14088E-62	*MALL*	-0.799135156	3.27831E-68
LINC00963	0.99708919	4.26522E-56	*CYP1B1*	-0.799076328	0.000379746
MRPL41	0.996506857	4.25656E-27	*SLC4A7*	-0.798593341	3.37881E-17
CSTA	0.99626431	9.9286E-08	*WWOX*	-0.796810009	0.000167418
UBE2F	0.995879318	8.83555E-33	*CLIC4*	-0.795312513	5.62001E-33
CYTH4	0.994966182	8.73872E-07	*SUMO3*	-0.795260544	4.73261E-32
VAMP1	0.994394978	1.24547E-14	*AC108488.1*	-0.793032331	0.005856171
SERPINE1	0.994341781	6.57339E-52	*SPAG4*	-0.792045538	0.009721604
AL157392.3	0.993526282	4.89373E-06	*SPRY4*	-0.7916007	8.29625E-43
ASB1	0.992071044	5.47715E-54	*ZFP41*	-0.789900035	3.9013E-09
KIAA1614	0.991799304	2.93238E-05	*KCNC4*	-0.789614096	1.3982E-16
PLCL2	0.991037986	5.12768E-12	*PTP4A1*	-0.789464001	2.98947E-19
EPM2AIP1	0.990078931	1.38328E-23	*RIBC2*	-0.787539206	0.000123436
CCPG1	0.989634791	2.05413E-17	*IGSF9*	-0.786752328	1.17694E-08
ST20-AS1	0.988739712	0.000120922	*AC024940.2*	-0.785236716	0.00067381
CAPN10	0.987959963	1.77326E-22	*NRP2*	-0.785143373	0.000294133
AC004980.1	0.98551536	3.87724E-05	*MYOM3*	-0.784579001	9.30256E-17
CALR	0.983007605	2.04015E-97	*SPTBN2*	-0.7845577	4.82426E-20
ZDHHC14	0.982504114	3.54041E-14	*ADM*	-0.784016415	7.53882E-16
CACNA2D2	0.98139106	6.4224E-23	*AC108488.3*	-0.783519503	0.006309651
MATN2	0.981127562	2.85972E-17	*MRPL13*	-0.781073776	3.78174E-23
SAMD4A	0.980179362	7.44827E-21	*RAB11FIP4*	-0.778888574	4.13575E-17
PHACTR2	0.980135699	2.38471E-25	*ZNF83*	-0.778767072	6.80871E-11
IQGAP2	0.978690609	6.54273E-05	*PPP1R1B*	-0.778652392	0.000218747
SSR2	0.978442118	3.18183E-63	*NARS2*	-0.778086013	2.59912E-28
RASSF8	0.971935835	5.01015E-26	*ID2-AS1*	-0.775042697	0.000709167
AL355987.4	0.967740636	2.04774E-10	*ATP2C2*	-0.774723774	6.76011E-13
WNT9A	0.96766801	3.64739E-18	*AL928654.4*	-0.774158154	3.23305E-06
CYSRT1	0.967332788	1.69536E-05	*RFC5*	-0.773461419	4.54339E-39
SLC33A1	0.966075098	8.77777E-58	*GPAT2*	-0.771013574	2.20434E-24
AL590399.1	0.9653909	0.002236941	*ELK3*	-0.769006628	5.61083E-39
AP002026.1	0.963316172	9.23951E-08	*MGAT4A*	-0.768756935	1.79754E-16
PABPC1L	0.962080585	7.02101E-40	*RAB29*	-0.768249211	1.97385E-25
RGS6	0.96203315	8.15144E-09	*ABHD11*	-0.766887435	3.06824E-26

COL4A6	0.962019437	4.0215E-06	*SMARCD2*	-0.766597636	5.8681E-58
WNT16	0.961654196	3.39056E-28	*OASL*	-0.765834808	3.00659E-12
SSR3	0.961224697	6.46334E-79	*HERC3*	-0.765540301	4.79458E-10
SEPTIN2	0.961155548	9.38623E-84	*ZC3H13*	-0.763074011	1.58124E-35
TPST1	0.961064965	4.93584E-25	*RNF213*	-0.762990941	1.32573E-28
ZBED6	0.96084836	3.02206E-05	*AP3S1*	-0.762794924	2.39751E-33
AL662795.2	0.958372767	1.49502E-07	*DDN*	-0.762338541	1.15026E-12
GOLGA6L4	0.957991603	3.81622E-06	*CDK5R1*	-0.761229901	4.83097E-10
MAN1B1-DT	0.956946405	0.000340575	*CHD3*	-0.76120917	6.27705E-38
BCL11A	0.955770075	0.002200607	*EEF1D*	-0.759660463	1.31822E-34
TGFB1I1	0.955425924	9.31573E-15	*WDR53*	-0.757840047	1.96257E-09
POMT1	0.954979108	3.17448E-36	*NR5A2*	-0.757439484	8.00504E-09
PI4KAP1	0.953635509	3.28467E-18	*TBC1D31*	-0.756645811	6.93773E-12
HIBCH	0.953204766	3.09048E-28	*FAM122C*	-0.754900855	1.96571E-05
VPS13B	0.953146279	8.45398E-30	*AC016831.6*	-0.753859842	4.6048E-06
AC004623.1	0.952257604	0.001822697	*MYC*	-0.753510694	2.50938E-42
SLCO1B3	0.951317418	3.2863E-10	*IL4R*	-0.752774452	3.17672E-26
ACER2	0.951311036	1.2363E-11	*AC113189.4*	-0.750449311	0.000425061
U47924.2	0.950870007	0.001186132	*CD3EAP*	-0.750358899	1.66764E-19
SDF2L1	0.947350819	4.69495E-24	*HMGCS1*	-0.750356239	3.7585E-27
HECW2	0.942088581	0.001178864	*KIF18A*	-0.747110541	2.57387E-17
DGCR5	0.941540736	3.06365E-06	*INSIG1*	-0.746205223	3.16297E-33
HID1	0.940128212	3.9161E-29	*MFSD3*	-0.745646167	6.82847E-14
ROCK2	0.940023235	7.5617E-43	*GPAT2P1*	-0.745150741	0.000271886
CCDC9B	0.939973475	2.32379E-31	*GSR*	-0.74464982	2.14402E-64
ISG20	0.939743216	1.64658E-08	*GJB3*	-0.742126761	3.39263E-18
GTF2IRD2	0.9397394	2.53626E-06	*TMEM92*	-0.741546126	2.45036E-08
FADS2	0.939709312	1.02108E-26	*CLEC4O*	-0.741251162	0.006177859
TVP23C-CDRT4	0.939356518	0.010143263	*ALDOC*	-0.741236073	1.88815E-22
SESN3	0.937890996	4.66842E-20	*CYC1*	-0.741080902	1.17848E-33
SH2D3C	0.937400762	0.000104015	*TAF1A*	-0.740688098	2.93696E-12
TOR2A	0.936370851	9.68032E-23	*HGH1*	-0.73839972	4.72087E-12
SRRM2-AS1	0.936102532	0.001937031	*OCEL1*	-0.737905342	3.75776E-15
PRICKLE1	0.935760608	1.76469E-08	*TMEM65*	-0.737036376	1.9551E-17
L1CAM	0.934750379	1.56277E-34	*PMAIP1*	-0.736892079	4.07504E-18
KRT23	0.933190171	2.45319E-07	*MTAP*	-0.736562566	2.18331E-35
GPR135	0.931537682	0.000288834	*BHMG1*	-0.73625134	1.59715E-11
COPS8	0.930705813	1.25235E-27	*PINX1_2*	-0.733693451	1.2687E-16
STRA6LP	0.929492548	8.74151E-07	*TTC26*	-0.733418795	1.28279E-11
COL1A1	0.928313804	1.7583E-26	*DYNC2H1*	-0.731046334	0.000626945
CDRT4	0.927242074	0.006054709	*C6orf132*	-0.726361262	1.01092E-20
MSLN	0.927236161	7.97565E-35	*TINCR*	-0.725743326	2.2248E-10
FARP2	0.92498114	4.43951E-30	*PDK1*	-0.72280554	6.60227E-17
ALCAM	0.924830518	6.37003E-46	*SLCO4A1*	-0.722544998	1.39733E-14
NR4A2	0.92316843	4.75277E-17	*SLC27A5*	-0.722384402	1.76779E-05
DHRS3	0.923113269	2.68847E-31	*PPP1R16A*	-0.721123254	3.23405E-20
HLA-DRA	0.922004683	6.52028E-07	*PARP16*	-0.720536635	1.51632E-07
AC109460.2	0.921430236	0.000401554	*ZC3H3*	-0.720473835	4.57917E-22
FAM229A	0.921273867	3.92513E-09	*BACH2*	-0.719464211	0.000152362
CTSO	0.919341321	3.42425E-05	*KLK1*	-0.719425713	0.009544798
ENG	0.919221679	0.002980334	*EMC2*	-0.718859256	2.8654E-14
PCDHGA10	0.918560946	1.11551E-10	*BRCA1*	-0.718124567	2.89913E-27
KIF26B	0.918478977	0.000177786	*C6orf223*	-0.717728872	3.89254E-22
CD37	0.9169036	6.58632E-05	*FAM91A1*	-0.717675266	5.32934E-26
PI4KAP2	0.91579978	1.77932E-26	*NYAP1*	-0.717307608	4.34179E-06
AL158206.1	0.91380131	4.58066E-09	*ZFP36L2*	-0.716383229	3.87209E-21
MYH16	0.913335247	2.79471E-09	*HSD17B14*	-0.714229877	0.0139362
BMP4	0.912882871	4.42768E-50	*ARL17B*	-0.713116338	1.28676E-05
AL096870.2	0.912237241	0.005157061	*OXR1*	-0.712553565	8.21431E-14

KHDC1	0.911909807	5.20146E-08	*PTGR2*	-0.710429419	3.24562E-13
KLHL13	0.90953382	1.04414E-09	*ST6GAL1*	-0.709390368	1.65014E-18
AC132219.2	0.908837078	0.001186255	*PITPNM3*	-0.708509986	1.37259E-05
MAGT1	0.908610529	7.34071E-73	*CXADR*	-0.708306392	1.04744E-12
DAPK1	0.902816452	8.82065E-12	*MFSD13A*	-0.70760586	2.95413E-14
CCDC92	0.900191468	1.46669E-09	*PRIM1*	-0.706864797	3.83119E-16
NDUFA10	0.900045383	7.57717E-62	*FASN*	-0.705256901	1.27622E-20
PRSS30P	0.900028648	6.30712E-12	*GOLGA7B*	-0.704656342	3.11975E-05
NT5DC1	0.899742347	1.29196E-38	*GRIN2B*	-0.704135419	1.97777E-12
C9orf16	0.897838584	1.54036E-25	*MYO1E*	-0.704087727	1.03668E-28
SCLY	0.894616481	1.02108E-26	*AC092279.1*	-0.703827902	0.000416675
FGF19	0.894079159	5.52608E-43	*LRRC46*	-0.703540077	0.025714417
SELENOM	0.892874157	6.25589E-22	*SCG2*	-0.701944697	7.6516E-14
AC007686.3	0.892242578	0.000422432	*DGAT1*	-0.701921556	6.65384E-18
HPSE	0.891399303	4.30302E-29	*F3*	-0.700683186	1.31989E-39
GPAT3	0.890500175	2.12629E-32	*E2F2*	-0.700663571	3.20594E-17
TRIM73	0.890488042	0.000586555	*MAD2L1*	-0.700020671	2.26851E-21
CSPG4	0.890284517	8.83555E-33	*FN1*	-0.699058703	4.69658E-10
TRAF3IP1	0.888852907	3.39235E-30	*CAPNS1*	-0.699037198	5.54084E-31
DPP7	0.886444851	7.2349E-22	*ELOA-AS1*	-0.698048961	0.005666826
LIPE-AS1	0.885062363	0.003581298	*TRAF1*	-0.695541488	0.005494694
PPIB	0.884722287	9.06279E-75	*SLC2A3*	-0.694365985	0.000126064
KIF9-AS1	0.8830051	0.000271938	*PLSCR1*	-0.69292968	3.25669E-22
CDH15	0.882962026	3.12759E-08	*NAPRT*	-0.692327476	6.38344E-17
MCF2L2	0.882946957	0.000665017	*MMAB*	-0.691272547	1.93553E-11
DOLPP1	0.882709074	1.97908E-28	*IFITM2*	-0.691146178	1.69212E-17
TMEM189-UBE2V1	0.881633975	2.00562E-06	*RAD51*	-0.690332293	1.01007E-17
CARD9	0.880824806	0.000496129	*FER1L4*	-0.688121189	6.45372E-09
AC010422.8	0.880694412	1.74015E-14	*ABCC6*	-0.686741203	0.005803213
GALNT9	0.880546675	8.30935E-07	*JPH1*	-0.686670125	3.78981E-15
LTK	0.880487327	3.20469E-21	*UBR5-AS1*	-0.686569678	0.008468696
TNS2	0.880425323	4.91229E-41	*AP1S1*	-0.686095491	2.97692E-35
MUC20	0.879798469	0.003039412	*LYAR*	-0.685957543	4.80997E-32
AL138756.1	0.879215353	2.07213E-06	*FXYD3*	-0.68537436	9.39071E-08
COL13A1	0.874236384	1.0602E-26	*SAT1*	-0.68536751	2.19523E-25
SUSD6	0.873408973	9.13907E-28	*ACVR1C*	-0.685366187	6.6108E-05
HECW1	0.873189111	1.76979E-08	*C4B*	-0.68518447	0.00472829
TENT5B	0.872898264	1.24407E-15	*GCNT3*	-0.684761969	6.62405E-06
LINC00482	0.872745881	1.25802E-05	*B3GNT5*	-0.684600722	2.25897E-16
NTSR1	0.871951151	7.13796E-34	*PUF60*	-0.683817993	3.86779E-27
SSR4	0.870940731	1.98292E-30	*STON2*	-0.683635134	0.008816442
PDIA3	0.870739174	2.1497E-112	*LDLRAP1*	-0.682341077	4.89595E-20
GSTT2	0.869448046	0.000372833	*DNAAF4*	-0.682053146	7.67502E-05
AC132872.5	0.869377309	7.6443E-05	*ITGA2B*	-0.680976037	0.000425061
DYSF	0.869164353	4.19757E-06	*C16orf95*	-0.680548341	1.51373E-06
GIPR	0.868693134	2.192E-13	*EFR3A*	-0.679976598	1.50866E-22
SEC61B	0.867327847	1.60078E-32	*LINC01719*	-0.67952714	3.58259E-05
MANF	0.867068225	2.9143E-56	*MRPS6*	-0.679418022	7.2847E-16
BHLHE41	0.865410242	1.99459E-08	*AKNA*	-0.678948951	1.09285E-08
MIR31HG	0.865078411	0.004202074	*CAMK1D*	-0.678317775	3.33443E-14
ZFYVE9	0.863635447	9.67507E-29	*LAD1*	-0.677229322	4.29308E-22
ZNF888	0.862946862	2.12374E-09	*LYPD5*	-0.677089509	0.000249394
NAGLU	0.862899086	2.53677E-19	*FOXL1*	-0.676735964	4.83587E-05
ACP2	0.862696861	3.78533E-16	*CHRAC1*	-0.675342989	3.34199E-25
APOBEC3F	0.862382791	5.19439E-28	*AC234917.3*	-0.674870561	0.00189823
AL035530.2	0.861316918	2.24999E-06	*ANKLE1*	-0.673600487	1.33486E-11
FUCA1	0.860216686	6.37568E-48	*MINDY4*	-0.673217002	2.67135E-06
ALOX12P2	0.859536094	3.93815E-06	*RNF125*	-0.673197419	3.61441E-12
ARHGAP31	0.857804965	2.85324E-07	*ZGLP1*	-0.673152986	0.017601904

KCNQ1OT1	0.856796609	0.000275303	*SPTSSB*	-0.671741647	0.008749355
ZNF469	0.856216997	3.44039E-10	*SH3PXD2A*	-0.671389288	1.08283E-11
TNFRSF13C	0.855347803	0.000328189	*SCARB1*	-0.670534887	6.36593E-23
GAS6-AS1	0.854893997	5.28996E-15	*RBP7*	-0.669793233	4.35126E-08
DOLK	0.85478129	5.70427E-21	*PRR15*	-0.668125713	0.002756013
DUSP4	0.853676749	2.79168E-69	*WLS*	-0.667173968	2.35444E-23
ZNF77	0.853337265	6.01678E-13	*NOXO1*	-0.667141859	0.002437698
KCTD12	0.852047366	1.37131E-08	*EPAS1*	-0.666052215	5.71178E-33
ERICH5	0.85022635	2.89894E-19	*VRK1*	-0.665988071	2.53279E-18
ANXA6	0.850104394	1.42084E-54	*DOC2B*	-0.665748912	4.45399E-14
FNDC3B	0.84957742	4.85442E-38	*TRMT10C*	-0.665042249	9.16882E-19
FOXA1	0.849096259	1.75055E-05	*EPPK1*	-0.664539908	3.10551E-11
PTGER4	0.84845711	2.03371E-07	*MIPEP*	-0.664245264	2.25627E-14
SCARF2	0.847388199	0.000233971	*NFIA*	-0.662905903	1.74214E-12
CASTOR1	0.845145311	5.79208E-05	*TMPO-AS1*	-0.66181506	0.00085038
TMEM229B	0.844822867	9.8216E-05	*ENY2*	-0.661807046	7.37527E-23
CCDC163	0.844784991	4.00004E-06	*MYCBP*	-0.661478052	1.53647E-17
AL731571.1	0.844546515	0.000148817	*ZNF649*	-0.659867	0.006001437
ARRDC1-AS1	0.840506984	1.66518E-13	*PRRT4*	-0.659360413	9.9608E-05
RFTN1	0.839661467	2.25112E-07	*AL606834.1*	-0.658753579	0.011530788
AC015813.2	0.839607407	3.45467E-24	*RAD51B*	-0.658096741	0.000154296
LINC02331	0.839155718	0.000161935	*DDX58*	-0.657567426	1.50448E-12
MAML2	0.838087483	5.52462E-17	*FAM166C*	-0.657252872	0.001838214
CAVIN3	0.837816207	3.46585E-15	*PAG1*	-0.656018748	0.000350888
NIPAL2	0.83717805	1.72447E-16	*ZNF704*	-0.654668537	3.24206E-22
DGKD	0.836568155	8.61678E-32	*SORD2P*	-0.654494705	0.001549538
LINC01521	0.835656576	0.002995729	*PET117*	-0.654272626	2.2038E-07
CADPS2	0.835473681	1.07066E-20	*EPHB3*	-0.65418871	8.139E-08
XRCC4	0.834944652	8.07701E-14	*MALT1*	-0.652679483	1.47116E-26
AC139887.2	0.833539481	8.33695E-05	*MCM6*	-0.652285261	6.38812E-32
DUOX1	0.833513516	8.21293E-17	*SCRIB*	-0.652038295	4.12956E-17
TMEM39A	0.833401001	4.95644E-33	*EIF5A2*	-0.651270256	5.93337E-11
FAM3C	0.829291472	4.55315E-26	*APIP*	-0.650039535	1.08E-19
PCDHAC1	0.829075132	0.003703848	*TAF4B*	-0.650000269	1.2582E-18
SYTL1	0.828769895	6.47082E-25	*CEP72*	-0.649614779	1.92601E-10
MAP3K12	0.828711612	8.28012E-29	*ETV7*	-0.649349449	0.013035968
ZNF767P	0.828428778	3.87025E-09	*DUS2*	-0.649250131	1.73254E-13
ZNF814	0.828048566	1.40245E-05	*NAA20*	-0.648719757	1.06106E-25
STAG3L1	0.828027349	0.000248511	*OSCP1*	-0.64862631	0.009380935
WIPI1	0.827845351	7.4956E-22	*RGL2*	-0.648322683	6.11402E-17
NPAS2	0.826431791	7.93488E-31	*SCML2*	-0.646606505	1.38475E-06
DNAJC3-DT	0.826279117	0.00336285	*ARHGEF25*	-0.646028203	4.28467E-05
SLC7A11	0.82617704	2.36367E-11	*THEM6*	-0.645017619	4.73226E-14
AC253572.1	0.82591172	0.017889073	*STK17B*	-0.644431429	5.3965E-09
AC084018.2	0.825807771	0.000177124	*DIXDC1*	-0.644124112	1.63413E-08
DTNA	0.825606607	7.05014E-10	*PEX5*	-0.643088456	1.24521E-25
C1QL4	0.825246581	4.79371E-11	*VOPP1*	-0.643033399	1.95881E-24
DIS3L2	0.824526021	1.67281E-19	*AC097448.1*	-0.642774924	1.12875E-06
ZBTB38	0.821531329	4.01782E-29	*RAET1E*	-0.64271873	0.000965171
SERPINA5	0.821442444	2.59191E-08	*LNCOC1*	-0.64214514	0.010559845
RNF207	0.820955596	4.03213E-27	*DIRAS1*	-0.641298346	3.27066E-26
HSPA5	0.819806149	9.5039E-133	*S100A3*	-0.641062842	0.001334294
TOM1L2	0.819564365	5.09351E-32	*AL357075.3*	-0.640776004	0.011258826
COL17A1	0.819425103	1.78133E-08	*LINC01694*	-0.640345386	0.011098647
STAG3L5P-PVRIG2P-PILRB	0.819349846	2.11136E-19	*UBL7-AS1*	-0.638720033	0.001593726
PPIC	0.814150116	1.26908E-29	*METTL15*	-0.638566204	1.10303E-16
MAMDC4	0.814100204	9.09347E-10	*ABLIM2*	-0.638509712	3.0228E-05
EDEM2	0.813396735	5.25424E-31	*TIGD5*	-0.638102393	3.97543E-07
LRRC8A	0.812408356	8.90747E-24	*ARID5B*	-0.636847038	2.6923E-08

SLC36A1	0.812300983	1.34435E-19	*FMNL2*	-0.636340049	3.78032E-16
SNED1	0.81206051	0.000452273	*DPCD*	-0.63604891	8.08087E-14
TSC1	0.811809132	2.786E-36	*DGAT2*	-0.635236164	6.56884E-05
COL9A2	0.810244709	1.18625E-07	*LMBR1*	-0.635087995	1.98582E-27
TOR4A	0.809474789	1.02895E-20	*AC084125.2*	-0.634875269	0.000132977
AC010547.4	0.806072532	0.001805932	*C21orf59-TCP10L*	-0.633059543	2.09088E-07
TMEM184A	0.803911096	3.78301E-22	*SYK*	-0.63265766	2.02689E-08
AGAP2	0.803477754	9.79798E-25	*ITGA2*	-0.631876339	1.43778E-16
RALGDS	0.803443235	3.30613E-34	*TRIM6*	-0.631708804	9.29463E-05
GPR107	0.802883028	9.38706E-47	*MND1*	-0.631027703	1.19021E-06
TPRN	0.801710388	1.39501E-19	*TIMM9*	-0.631021099	1.04939E-07
LINC01089	0.800281095	1.56929E-06	*ACAT2*	-0.630018488	3.8771E-16
DTYMK	0.79983295	3.07245E-25	*FANCI*	-0.629605678	3.88275E-22
ACBD7	0.798755904	5.39972E-10	*RPL8*	-0.628005105	9.23324E-22
COLQ	0.797348874	0.006850841	*UGCG*	-0.625657494	2.72044E-14
H2AW	0.797078875	6.19649E-07	*HOMER1*	-0.625608962	9.67034E-14
PLCD4	0.796788258	0.000118588	*ASB13*	-0.625258364	4.02279E-15
FBXW5	0.796534327	2.89757E-19	*LDHA*	-0.624965826	2.17692E-25
ABCA3	0.79650855	2.26369E-19	*TONSL*	-0.624317769	2.88731E-12
ACHE	0.796330769	1.62167E-05	*HSPD1*	-0.623785409	1.60424E-30
STAG3L5P	0.796100038	1.16002E-10	*EGR2*	-0.621228012	0.016418798
FKBP2	0.79546972	9.39626E-18	*NOVA2*	-0.620080636	3.2314E-08
PPP1R32	0.794668065	0.004841043	*RBMS1*	-0.619999854	1.39699E-17
AHNAK2	0.794603857	4.31542E-26	*MLLT3*	-0.619808158	1.55484E-06
IER3	0.794470141	3.29122E-57	*ZNF7*	-0.619801058	2.30027E-15
THAP8	0.794032887	0.000855026	*C1orf116*	-0.619645006	4.22276E-10
APOLD1	0.793441404	0.000573059	*NET1*	-0.619409617	1.10891E-17
MFSD4A	0.793138524	0.001285176	*ASIC3*	-0.618737422	0.023998734
ARMH4	0.79298786	0.000361173	*LBR*	-0.61803011	1.13309E-21
ADGRG6	0.792121917	3.35014E-36	*AC004803.1*	-0.617945553	0.034544195
FAR2	0.791444715	1.0023E-10	*HMGA1*	-0.617926221	9.67511E-20
AQP3	0.790714613	6.36532E-06	*HSH2D*	-0.617920305	2.22091E-12
HGSNAT	0.790450186	2.54532E-29	*DEPTOR*	-0.616475543	5.69698E-06
USP20	0.790342063	7.84718E-24	*GABRD*	-0.616126251	6.38705E-09
AC068946.1	0.788925589	4.08745E-06	*DUSP7*	-0.61572588	4.60425E-15
NOTCH1	0.786853535	5.45171E-22	*ARV1*	-0.615559397	0.006955951
TRIM22	0.786428865	0.002874781	*FERMT1*	-0.615155462	1.34722E-28
CLN8	0.785279869	8.68082E-29	*AL359922.2*	-0.614553959	0.045284512
AXIN2	0.784191409	1.18669E-10	*CCDC68*	-0.613784084	2.54427E-06
AGPAT2	0.784122454	8.73062E-21	*OCLN*	-0.613742136	2.70748E-17
TMEM45A	0.78371705	6.29084E-11	*FKBP5*	-0.61371744	4.89776E-24
SLC27A4	0.783605654	1.2449E-24	*KHDRBS3*	-0.612908819	5.71187E-10
ATP6AP1	0.783295551	6.31379E-45	*KLF10*	-0.612521457	2.26534E-21
C3orf35	0.782674662	0.017778265	*PITPNC1*	-0.611970267	1.28766E-20
ATG16L1	0.782313858	1.19974E-28	*SMS*	-0.611348208	3.72199E-29
CNTNAP3P2	0.782196369	1.57064E-05	*AC108174.1*	-0.611159366	0.017815092
AL358113.1	0.781422518	0.009629585	*POLA2*	-0.611116179	1.60578E-09
DNAH10	0.781230182	0.002350859	*PAQR5*	-0.610824912	1.31341E-15
STAT4	0.781133771	2.03472E-06	*VDAC1*	-0.610096671	6.79339E-52
AL360181.3	0.781106652	0.009368708	*PHGDH*	-0.610036472	8.78369E-24
BDNF-AS	0.780945426	0.000387073	*FANCF*	-0.609401503	4.42868E-05
SH3GLB2	0.780524556	9.56963E-34	*PRKAG2-AS1*	-0.608903585	0.006950347
AC027290.3	0.77966779	0.000556286	*ZNF485*	-0.608844413	0.002226094
PTOV1-AS2	0.778775565	0.025795694	*OAS3*	-0.608114982	3.95332E-20
PTPRJ	0.778701312	2.73003E-30	*WNT7B*	-0.608037261	3.21753E-10
THAP4	0.778217032	7.48877E-27	*PXK*	-0.606852702	4.94195E-08
AC010761.1	0.778072519	0.033125283	*LINC01806*	-0.606267363	4.48713E-05
FKBP14	0.77583052	2.90162E-16	*SH3BP1*	-0.606105324	8.66136E-10
SERPINB9P1	0.775558208	0.001947407	*PRKCQ-AS1*	-0.606003683	0.001842939

SIK2	0.775557411	1.83616E-30
AL512625.1	0.775133129	0.001866543
RGS5	0.774421296	0.000966649
SPCS2	0.77248046	9.79309E-51
AC004922.1	0.772313397	0.001211371
NEBL	0.771358839	2.45992E-17
FAM95B1	0.771082534	0.000199211
SCAF1	0.770500157	4.51703E-24
CNTD2	0.769785803	3.73443E-08
HOXC6	0.769436127	1.73754E-07
ENDOG	0.769337113	1.35901E-12
UAP1L1	0.769288993	4.29522E-20
SLC22A18AS	0.768750495	0.011903231
AC006254.1	0.768624597	0.007638903
AC046134.2	0.768380734	0.001568735
MSL3P1	0.768296539	1.16474E-08
ANKRD6	0.766678985	5.77964E-12
AL590560.3	0.766516794	2.76925E-05
FNDC11	0.765236155	0.001803403
NCSTN	0.764014956	2.76385E-47
PTGES2	0.763693259	1.72058E-27
TOR1A	0.762681573	1.75205E-36
ICA1	0.762323249	3.26194E-13
L3MBTL1	0.762055457	8.48382E-14
EIF2AK3	0.761894682	1.29497E-19
SH3BGRL	0.761328165	5.19697E-26
RNF19A	0.761270935	7.51043E-26
DGKA	0.760678919	1.11091E-41
ZNF594	0.76062272	2.29436E-05
NPDC1	0.760358514	5.17023E-17
SUGT1P4-STRA6LP	0.759859365	0.025281074
VPS9D1	0.758237204	9.78008E-07
NHS	0.758234622	2.37352E-15
MOB3B	0.758131458	3.5038E-05
INKA2	0.758100127	2.75564E-12
ZSCAN31	0.757331843	1.32683E-07
HOXA3	0.75725282	1.15941E-09
TM9SF1	0.756399894	4.87219E-23
RCBTB2	0.75637456	0.000747149
ECM1	0.754637328	9.80433E-05
MMP14	0.754158497	2.22219E-28
PPP1R7	0.754119167	3.24641E-27
PTGES	0.753286529	1.90315E-26
URM1	0.752553913	6.6488E-26
PSKH1	0.752191112	1.48576E-16
PTPA	0.752134714	1.53751E-41
HES2	0.751683255	1.46843E-17
AC023509.1	0.751202268	4.67976E-06
VAMP2	0.751030967	7.5135E-25
AP001453.5	0.750407936	0.001134946
TMED10	0.750211042	6.08794E-55
COG3	0.749440202	1.31214E-22
TMEM214	0.749136061	1.22889E-47
SCNN1D	0.7486118	9.86537E-06
ABCC3	0.747685366	6.45486E-24
COQ4	0.747566585	2.18205E-24
CCSER1	0.746881725	0.003936663
TP53INP2	0.746699291	3.59943E-11
STARD5	0.745983154	3.57592E-05
FAM135A	-0.605909088	1.24541E-09
THEMIS2	-0.603839463	1.37781E-07
MAF1	-0.603313673	6.32423E-16
ITGB3BP	-0.603229221	7.22937E-12
COQ2	-0.602907672	1.84794E-09
LSM6	-0.602771376	5.36251E-11
AUNIP	-0.602169379	8.31897E-08
PRKG1-AS1	-0.601131251	0.009320957
KIAA0040	-0.601078168	1.36193E-23
CCNB1IP1	-0.600968963	4.6994E-12
PDLIM3	-0.599753366	8.90024E-06
GREB1L	-0.599746847	2.9849E-06
SNRPA1	-0.59925001	2.89913E-27
RPP25	-0.596862262	0.000107133
GRPEL1	-0.596707657	9.22444E-25
FGF11	-0.596477683	0.010143263
NOLC1	-0.595977961	3.39368E-52
C15orf41	-0.594203595	1.17466E-07
SLC39A8	-0.593901787	1.15332E-14
BTG3	-0.593247733	4.50095E-23
RALB	-0.592151625	5.86301E-25
NUP155	-0.591584866	4.61927E-19
AP1S3	-0.591042875	1.31845E-20
AACSP1	-0.590405755	0.036414296
PRPS2	-0.590180942	2.27969E-20
BCL2L12	-0.589944156	8.7513E-09
GMNN	-0.589542683	2.69017E-15
RCL1	-0.588929946	1.45066E-18
SYTL3	-0.588574299	1.25008E-17
HHEX	-0.58738418	1.20839E-07
EFHD2	-0.587282351	6.37363E-19
CRABP2	-0.586328601	6.3487E-19
ARMC9	-0.585664475	1.04612E-09
GTF2E2	-0.585045784	1.92244E-20

AK1	0.745235791	8.02118E-19
STXBP1	0.744267085	2.50682E-45
SLC49A3	0.744113286	0.00470993
SLC20A1	0.743739642	4.76911E-60
SLC22A15	0.743197021	2.30795E-06
C12orf57	0.7431002	1.96014E-06
AC006128.1	0.74286968	7.31039E-05
LINC02035	0.742746189	5.14369E-08
OSER1-DT	0.741772053	0.000387812
NALT1	0.74156349	0.003171623
ARHGEF6	0.741472413	1.20747E-06
FPGS	0.740775638	2.32402E-25
DHRS7	0.740534508	1.01508E-37
GOLGA2	0.740044984	4.12753E-45
OS9	0.739558288	3.74729E-56
CAMTA2	0.73899792	6.27728E-22
LRRFIP1	0.738959568	3.189E-38
CST6	0.737527587	9.04252E-11
LINC00326	0.736748536	2.05565E-05
SLC39A7	0.736526257	2.32058E-54
CADM4	0.735771301	1.02869E-29
IDUA	0.735673806	9.41932E-10
PRAF2	0.735545457	4.77664E-17
ZNF579	0.733479281	2.66678E-14
AC104452.1	0.733307573	1.85408E-06
AC026471.1	0.732548227	7.89937E-05
HERPUD1	0.732341049	9.17589E-33
ALG2	0.73173627	3.84972E-29
RPN2	0.731187328	2.25955E-51
ATF7IP2	0.730411997	1.37994E-07
CSGALNACT2	0.729943055	2.95332E-15
BVES	0.729612864	9.56006E-07
RNF182	0.729386105	3.17231E-13
PGAP3	0.729127253	3.33464E-10
OR51B4	0.728996875	3.8406E-07
MSANTD3-TMEFF1	0.728932969	0.004217115
ZSCAN5A	0.728748101	5.81084E-05
AL590822.3	0.728434083	0.002280917
PDGFB	0.728351787	5.74613E-24
CSAD	0.72810548	5.88025E-08
HPX	0.727780514	0.011295691
RPN1	0.72757406	2.42775E-54
ATXN1	0.727406821	1.14431E-11
RNF215	0.727361366	1.40902E-21
VMAC	0.726475778	0.008601967
ARHGAP40	0.725821566	0.005367773
LAMC3	0.725761154	1.20414E-10
HERC2P3	0.725646543	5.82548E-10
ACTA2	0.725609903	8.11197E-10
NEIL1	0.725056277	1.90029E-20
AC018865.3	0.724713678	0.008466924
TTLL7	0.724620073	1.15441E-12
CRIP1	0.723954705	0.005201934
NR2F1-AS1	0.72340284	5.30527E-16
PKD2	0.723032825	3.85892E-20
UGGT1	0.722361059	5.65763E-36
SPRY1	0.721085403	2.86577E-09
CHAC1	0.720395157	0.00047693
AL022322.1	0.719546023	0.01660063

NAIPP2	0.719124172	0.000547856
DDOST	0.717053292	1.12714E-52
PTCHD4	0.716924385	9.64345E-05
AC139795.2	0.716759516	0.012575877
SETX	0.716408809	3.75606E-32
ORAI3	0.716343451	9.34012E-11
C1QTNF6	0.716325534	1.3012E-12
AL590560.2	0.715874373	1.88799E-05
FRMD4A	0.715393188	0.002921846
SLC25A25	0.714993755	8.43349E-26
ZER1	0.714947597	6.40499E-27
SPATA20	0.713830224	3.71534E-29
FBLN1	0.71335293	4.69953E-22
FAM83E	0.712817605	0.008466924
ERFE	0.712814345	4.22328E-09
INPP5E	0.712675193	7.66462E-15
AC078993.1	0.712673355	0.001123439
EIF3FP3	0.712465548	4.12501E-06
ATF6B	0.711719255	1.0518E-28
RPS6KA2	0.711499438	3.05575E-11
BTG2	0.711326291	5.22258E-32
NXPE3	0.710352306	8.4945E-12
CALB2	0.710284049	3.28462E-12
SPRY2	0.709127845	1.2204E-09
STT3A	0.708695231	3.40316E-70
PSD4	0.708033219	6.32958E-15
LINC01270	0.707750897	2.69261E-09
RPL32P3	0.707462535	9.36556E-06
RNF223	0.705509163	0.000510289
ADM2	0.704817868	2.1507E-11
CLGN	0.704396708	8.59459E-05
DRAXIN	0.70427756	1.51461E-05
ICAM5	0.703374868	1.93311E-16
MINAR1	0.703247941	0.007400599
SGMS1-AS1	0.702018088	0.000616196
SIL1	0.701087192	8.76416E-33
ENO3	0.700830995	7.86246E-08
TMED2	0.700383393	1.95869E-41
ARHGAP11B_2	0.700004293	0.022311305
H3C6	0.699728206	0.000433117
COL4A5	0.699112882	3.57619E-22
HSP90B1	0.698010519	2.67709E-43
CD99	0.697807926	1.46136E-27
EEF1A1P6	0.697730251	0.044462131
BX255925.3	0.696796662	4.78936E-10
YBX1	0.69679301	2.33415E-42
PMS2P3	0.696467436	0.000607202
TTC13	0.696401669	4.48614E-15
PEAR1	0.696197644	3.196E-15
TVP23C	0.69608039	0.000249038
C14orf28	0.696050624	1.58423E-05
DNASE1L1	0.695850387	2.82637E-17
LINC02163	0.694997718	0.046036945
CARMIL1	0.69341354	2.92452E-21
SRPRB	0.691957961	1.45835E-41
RARA-AS1	0.69146521	0.021933518
CYP1A1	0.691414158	0.008880831
AC009690.2	0.691137802	0.020685007
H2AC11	0.690387887	0.034306789

KIF5A	0.689750333	1.54703E-09
NPTXR	0.689313841	2.7825E-22
ROGDI	0.688940446	3.00053E-15
TMEM132A	0.688612821	1.35137E-19
LAMA3	0.687807931	1.18347E-13
HIVEP3	0.686967965	9.43467E-16
FTX	0.686802134	6.89761E-08
ARSA	0.685911399	1.77561E-11
AGAP5	0.685514978	0.008654358
NOXA1	0.685049866	0.000241985
TMC3-AS1	0.684777058	0.038151958
TES	0.684763995	2.21786E-38
FGD4	0.684196245	1.45199E-11
PLCD1	0.683787518	6.69094E-08
LINC00476	0.683191354	0.004975052
SERPINF2	0.682773808	6.92094E-06
BOK	0.682699385	2.8861E-21
AP001273.1	0.682451674	0.022924295
MFAP2	0.682347179	1.22445E-21
MCOLN3	0.682099228	1.67668E-16
ABCC10	0.681795914	1.26538E-23
LINC00174	0.681427303	9.77546E-07
WDR45	0.68121511	5.87785E-16
TRAM1	0.680949027	4.48508E-33
SRGAP3	0.680397865	5.33977E-09
H1-2	0.679931422	0.00495398
AC073957.3	0.679338891	2.92105E-06
SRD5A3	0.678943914	9.55403E-15
PMFBP1	0.67876076	0.008103263
HM13	0.677225014	6.02033E-31
ABCG4	0.677158549	1.12083E-05
TMED2-DT	0.677011717	0.01329541
LAMB2	0.675521936	2.75705E-27
HSD17B7P2	0.674294718	0.008684925
ENTPD2	0.674016765	1.82111E-12
AL021707.2	0.673694434	0.025799232
EPHA10	0.673616506	1.37918E-11
SLC27A6	0.673153418	2.3397E-12
CHST6	0.672992414	5.28297E-24
LINC00863	0.672678658	0.000305573
HTR7P1	0.671603677	0.001415487
ZNF75D	0.67151837	1.32806E-07
MSRB3	0.671101183	1.3886E-11
ABAT	0.670108706	4.85314E-07
THSD4	0.669526661	6.95919E-14
RXRA	0.668759193	1.43734E-26
KCNIP3	0.668736939	0.00207988
ABALON	0.66856231	3.53205E-12
FKBP11	0.668488432	0.004258816
ATM	0.668152338	2.51851E-11
AL121845.3	0.668033464	2.73785E-07
PINK1-AS	0.6676993	5.20731E-13
SLX1A	0.667611399	0.013753223
SLC41A2	0.66753003	2.46485E-09
AC006230.1	0.667444753	0.000962111
HSF4	0.667347148	2.18505E-08
BICD1	0.666728493	5.80158E-15
PRPF40B	0.66641566	6.04563E-18
SLFNL1-AS1	0.666314583	0.01876048

ITFG1	0.666288667	9.08908E-25
WDR72	0.665533556	7.40681E-09
SGSH	0.664801247	1.03824E-12
AC105233.5	0.66454288	0.003377181
ST3GAL3	0.664041467	0.00727407
DISP1	0.663953297	8.30515E-06
FAM124A	0.663489832	0.000351055
FBXL8	0.663216207	8.44371E-07
AC015813.6	0.662913655	4.55404E-07
AC241952.2	0.661905354	0.025351739
UFSP1	0.661705265	0.029953669
MYO10	0.659521989	7.88273E-27
YIF1B	0.659404442	4.96331E-16
RAPGEF1	0.658589017	6.07394E-22
ABCA5	0.658584898	5.22098E-11
ANO1	0.658512204	5.71206E-37
MARCHF1	0.657677424	0.000388158
SLC23A3	0.656789301	0.002006684
POMK	0.656675541	0.000346499
QRICH2	0.656582781	0.001947407
AC093157.1	0.656348201	8.67617E-05
ZNF467	0.65633245	1.43689E-09
CIDECP1	0.653781249	0.00122736
AC080112.2	0.653465389	0.013123814
ADAMTS16	0.653007537	0.014537318
DLG5	0.652633968	2.48096E-27
ERVK13-1	0.652339868	2.58397E-05
METTL7A	0.651617202	4.88755E-07
C1orf50	0.651446893	4.21266E-05
MXD1	0.651391191	3.0149E-06
SNHG7	0.651112939	3.43764E-23
GPC2	0.650946239	0.00016129
CREBRF	0.649933937	4.04469E-08
WBP1	0.649854753	1.5987E-22
ATG4A	0.649466702	2.81768E-07
GMPPB	0.649372827	2.27617E-19
POGLUT2	0.649275537	1.28082E-05
ADAM22	0.648933988	4.06432E-12
JMJD7-PLA2G4B	0.648735226	5.01873E-09
AC007906.2	0.64810899	0.009993933
GOLGA6L5P	0.647180369	3.4788E-05
LINC01963	0.646508839	0.000185802
ARMCX3	0.644882453	1.87365E-22
NAV3	0.644754262	1.95063E-05
EML2-AS1	0.644008316	0.036919494
MINDY1	0.643956704	0.047796357
IFI27L1	0.643713176	9.00995E-07
GMPPA	0.643703356	2.48059E-17
RAB37	0.641696786	1.05E-11
TMEM255B	0.641468563	1.91785E-05
TLE6	0.641418551	1.60255E-05
FAS	0.641073751	1.43443E-15
BMP1	0.640788159	1.29706E-27
SPACA6	0.640575204	0.001155814
LTBP3	0.639864185	3.28765E-22
RHGAP27P1-BPTFP1-KPNA2F	0.639386063	5.89385E-05
CD109	0.63932316	1.6015E-21
KRBA2	0.639271575	0.01751146
CDC42BPA	0.63920548	1.87798E-20

ASB6	0.639106634	5.01269E-22
SMIM14	0.636769171	1.72965E-15
KRCC1	0.636710813	4.37046E-07
ANO8	0.636087228	1.07775E-12
AC012513.3	0.634789026	0.019340243
EFHC1	0.634714214	1.06386E-07
LINC00923	0.633971855	0.00398602
SDSL	0.633850913	5.58216E-10
FDXR	0.633811175	2.31205E-22
PRRG2	0.632938624	7.07245E-05
MTDH	0.632933848	1.7175E-29
CORIN	0.631766275	0.005499556
ACCS	0.63149603	1.90437E-06
MIR4435-2HG	0.631062627	1.17447E-13
FAM184A	0.629539609	0.024500525
SGK1	0.629483933	0.003444071
CCDC142	0.629400267	1.32534E-08
FNBP1	0.628841995	1.9169E-26
ADAMTSL4	0.628410982	0.000608446
KBTBD7	0.628166231	6.27672E-11
SPCS3	0.628053829	1.78611E-20
RHOU	0.627599723	0.000627944
TTYH3	0.627051835	8.54934E-14
LMAN2L	0.626884849	2.50977E-13
PLEKHG1	0.625529973	2.30186E-11
SERPINI1	0.624923814	0.000448204
CACNA1A	0.624854854	0.000106484
AC106782.1	0.624536946	0.016645011
GBA	0.624507582	1.71668E-13
MSANTD2	0.624329417	6.87709E-06
CPLANE1	0.624079592	1.61276E-07
KIAA1211L	0.623659731	0.02677653
BTD	0.623505779	2.73183E-07
OPTN	0.623162057	4.74632E-22
HS3ST3B1	0.622814241	0.004533657
NUDT16	0.622215584	1.0578E-13
FOXJ1	0.622169691	0.048912337
SH3BP5-AS1	0.621276668	0.002523351
SLC9A2	0.621212866	0.000462581
PSD2	0.620931935	0.006945239
THUMPD3-AS1	0.620798189	7.15559E-06
CAPS2	0.620664643	1.63687E-05
OSTC	0.619707054	2.15525E-19
SPACA9	0.619301925	1.33073E-05
PRRT2	0.618992286	0.001222316
GAB2	0.618529521	0.000383748
EVI5L	0.617814391	4.49883E-13
AUP1	0.617236057	2.06989E-29
NCMAP	0.616841102	0.016715202
AC068946.2	0.616246682	0.006849656
DPH7	0.616164072	5.35986E-19
KIF16B	0.616081171	5.73168E-08
IL11	0.615409785	0.007478158
CYP4F12	0.615402409	0.003508493
ABHD4	0.615302698	1.161E-09
PCLO	0.61514573	6.49023E-05
AC027601.6	0.614978494	0.006029417
TNFRSF10A-AS1	0.614944091	0.013778282
CNKSR1	0.614833658	1.10476E-13

PHLDA3	0.614723683	2.7271E-11
ADGRV1	0.614653446	0.000641871
CPB2-AS1	0.614102687	0.036171357
TMED5	0.613538172	7.27892E-20
TOB1-AS1	0.612713625	0.017575486
LCAT	0.612322925	0.001049342
AC245452.1	0.611509786	0.006963461
CACFD1	0.61130898	2.70558E-06
FKBP10	0.611271076	1.39129E-32
AC073508.2	0.610035913	0.004214538
ANKDD1A	0.609651265	7.8303E-06
GPSM1	0.609584705	1.60352E-11
LAMB3	0.608393328	3.1083E-17
ANAPC2	0.607300052	5.57825E-18
AC078820.3	0.60713973	0.040481083
RSRP1	0.607112548	3.0962E-16
TPT1-AS1	0.606929891	1.94528E-09
LPXN	0.606032693	0.011697753
UNC93B1	0.606025324	4.93807E-17
BBC3	0.605372294	1.15963E-09
WSB1	0.604281438	2.99735E-12
IL10RA	0.604195375	0.048781695
TMEM117	0.604028798	0.000369574
PIGCP1	0.603954972	0.036416644
SDCBP2	0.603661209	0.041531932
TYMP	0.603184941	5.46913E-14
CORO1A	0.602156591	0.003094812
ZNF425	0.601700211	8.68052E-05
DHRS13	0.601646367	8.94775E-09
ANK3	0.601436421	9.51746E-08
PER3	0.601312613	6.87211E-07
SHANK2	0.60122615	3.76185E-10
OLFM1	0.601221509	0.000219892
DNAH1	0.600622164	0.000299775
LEMD1	0.600508124	0.000155244
NOP14-AS1	0.600444763	5.03603E-09
MOGS	0.600221143	1.35158E-25
CDK2AP2	0.600108621	9.51435E-15
HDAC4	0.599764629	7.91393E-11
GALNT4	0.598742302	0.001060714
TMPPE	0.598278755	0.029026154
PRSS53	0.59803223	7.55663E-08
AADACP1	0.597900001	0.01095173
TMC4	0.597586217	5.23557E-13
ATXN7L1	0.597227067	1.09385E-07
LIME1	0.596967865	9.22683E-08
TMEM98	0.596778611	6.02846E-13
ALDH3B1	0.596648256	2.46008E-16
ZNF276	0.595722111	1.71733E-19
TERF2	0.594877194	2.0933E-20
SEC31B	0.59456338	1.9109E-15
EPHX1	0.594348011	2.10393E-17
PAX2	0.593615188	3.61022E-05
ZNF561-AS1	0.593226756	0.003908665
KRTCAP2	0.593140429	3.6856E-10
MGAT5B	0.592243853	9.47174E-06
RABL6	0.592210362	8.37386E-18
CIZ1	0.592130027	2.05413E-17
NPIPB4	0.591764751	0.001702082

LERFS	0.591552922	0.03900725
KIAA0895L	0.591551254	3.24123E-11
GOLGA8B	0.590874494	2.22898E-11
AC008124.1	0.590621275	0.001015774
UBAC1	0.59052923	6.94761E-25
PDIA3P1	0.590484888	0.025899342
FBXL19-AS1	0.590253885	0.007294044
MYLIP	0.590023461	9.28079E-10
OTUD6B-AS1	0.589812153	1.66954E-10
SUCO	0.589784847	3.99534E-16
CYP2D7	0.589663574	0.00872428
LYG1	0.589446542	0.047116946
FAM210B	0.589278993	9.28491E-16
STIMATE-MUSTN1	0.589274644	0.007700198
BMP8A	0.589177122	3.07844E-06
MAP3K14-AS1	0.58904381	0.001240202
SUSD1	0.589000583	2.9811E-07
LIX1L	0.588772172	1.64869E-17
GPNMB	0.588536511	0.021591138
LMAN2	0.588462077	1.92723E-21
NPIPB5	0.588020965	1.161E-09
NTN1	0.586860765	2.33198E-07
CRIM1	0.586300818	5.19439E-28
TRPM6	0.586197932	0.042430765
KLRC2	0.585892951	0.000370504

Table S8. List of mRNAs significantly regulated in HCT116 *WT* cells when treated with 5-FU.

Significantly upregulated mRNAs			Significantly downregulated mRNAs		
Gene symbol	Log_2 fold change	padj	Gene symbol	Log_2 fold change	padj
COL17A1	5.093205151	0	*RPL22L1*	-2.947739468	1.078E-234
KRT81	4.541001833	2.4813E-142	*PPP1R1B*	-2.425424705	1.17263E-27
NECTIN4	4.364619028	8.243E-151	*SPARC*	-2.208205224	9.38991E-19
MYH16	4.343242754	2.9845E-253	*PRKACB*	-2.155454499	2.8823E-266
GALNT5	4.238747906	0	*DLEU2*	-2.119668913	4.85432E-68
CEACAM1	4.223837471	3.4426E-263	*INSIG1*	-2.042148672	3.2473E-212
TP53I3	4.149871832	0	*RGS2*	-1.970613188	9.7965E-167
PTAFR	4.099904536	0	*PLK4*	-1.951654467	2.4334E-110
HMCN2	4.098342255	4.27724E-90	*LINC00326*	-1.930782623	1.97236E-19
KLHDC7A	4.079223742	6.06374E-89	*SESTD1*	-1.869828138	5.04359E-73
WNT7A	3.888622884	2.0242E-278	*NR2F1*	-1.821058792	1.0275E-151
LAMA3	3.888309981	0	*L3MBTL3*	-1.804762153	1.4078E-120
GPR87	3.85115528	8.5186E-196	*ARMC4*	-1.741043482	5.88331E-75
CDKN1A	3.676709945	0	*EREG*	-1.719528074	4.2862E-129
PLXNB3	3.595207534	9.8538E-157	*PRIMA1*	-1.677871839	1.72041E-12
AC007906.2	3.462150575	1.71506E-67	*DDIT4L*	-1.659484037	7.30389E-46
CEL	3.396282562	7.94E-128	*CLYBL*	-1.658771111	3.82705E-38
CYSRT1	3.386425103	3.91037E-69	*LRRCC1*	-1.650219229	7.59485E-55
TRIML2	3.373690427	7.6982E-121	*CUTC*	-1.648737056	1.38604E-96
APOBEC3D	3.365295873	5.58333E-55	*OR2A7*	-1.643719823	3.52987E-14
C1orf116	3.332180219	0	*SLF1*	-1.638247559	8.58063E-58
SERPINE1	3.302111157	0	*ANXA10*	-1.636378187	3.37042E-35
CHAC1	3.292446232	1.5068E-80	*ZNF704*	-1.63546185	1.4859E-122
KRTAP2-3	3.288805903	2.1911E-240	*FBXO4*	-1.621460974	4.0332E-22
CD82	3.23997658	0	*TEKT4P2*	-1.610078315	1.51312E-14
CPA4	3.227501746	0	*DENND5B*	-1.606498616	6.55475E-75
NRP1	3.195716765	1.2278E-141	*ZNF367*	-1.603154848	3.0279E-114
TNFRSF10C	3.164356285	1.7382E-146	*MAN1A1*	-1.602962566	8.9261E-103
BTBD19	3.142015702	1.2705E-105	*TMSB15A*	-1.597521364	1.03717E-18
PRODH	3.123777772	1.797E-182	*KITLG*	-1.58478276	6.9154E-130
TP53INP1	3.111066614	0	*FOXC1*	-1.576301382	2.5447E-127
TRIM22	3.082868097	3.13541E-45	*KLRK1*	-1.574624308	8.10024E-07
WDR63	3.079215439	9.5945E-116	*C2CD4C*	-1.567121843	2.41601E-31
LAMP3	3.020087011	0	*ST3GAL6*	-1.541946331	7.10318E-32
CYP4F3	3.019026509	2.53829E-36	*FAM156B*	-1.539955641	0.001989351
MDM2	3.012217042	0	*PCDH7*	-1.529493801	4.8578E-112
ABCA1	2.99075455	2.77621E-83	*DDN*	-1.50724733	5.90795E-43
DRAXIN	2.978238432	1.9383E-110	*RIMKLA*	-1.501509418	2.72162E-34
TMEM249	2.970781543	4.56636E-80	*SCD*	-1.500323375	7.9749E-208
ZBED2	2.949943667	3.3256E-180	*DEPDC1B*	-1.48237817	2.25114E-47
SMIM10L2A	2.91647807	2.24887E-93	*MANF*	-1.472372169	3.788E-134
SERPINB5	2.905030656	0	*C22orf34*	-1.471866514	9.22651E-12
COL4A6	2.904651218	1.69872E-58	*TMX4*	-1.470953569	1.18535E-45
HES2	2.900921215	1.1306E-282	*DEPDC1*	-1.462022665	5.06402E-21
THBS1	2.878935224	0	*OR51B5*	-1.461144691	8.92852E-67
GJB4	2.854783065	1.54446E-83	*ENPP1*	-1.45640596	2.21624E-35
KRT80	2.805436423	8.5317E-249	*HMGB2*	-1.44634792	6.23053E-90
TLR3	2.801889287	1.1506E-166	*TNFSF18*	-1.438946968	2.79623E-11
LTBP2	2.761933124	7.8437E-138	*RASSF8*	-1.433847017	2.47783E-45
GDF15	2.759766024	6.2152E-277	*SMAD9*	-1.42709926	7.31675E-10
ACHE	2.733006722	4.547E-66	*ZC3H6*	-1.424355812	1.68347E-25
PRDM1	2.731234785	8.10767E-50	*C1orf21*	-1.417198841	1.72298E-32
COL16A1	2.690025512	1.946E-106	*MYB*	-1.41681652	3.95663E-26
SULF2	2.66452505	0	*EFNA2*	-1.415373727	3.53671E-36

ECM1	2.660244891	1.37263E-56	*TMEFF1*	-1.415322732	9.27448E-07
ATG9B	2.648858494	2.88244E-47	*NR2F1-AS1*	-1.410456544	5.93172E-43
CCN1	2.643804011	2.663E-241	*ARHGAP11A*	-1.404409297	2.11015E-73
UNC5B-AS1	2.641932156	1.52047E-59	*ZDHHC2*	-1.395906598	8.36023E-81
AL109976.1	2.618076893	4.33117E-33	*KCNQ1*	-1.393748256	4.24031E-23
NANOS1	2.617844164	4.08674E-78	*SNRNP48*	-1.393574701	1.06539E-67
H19	2.6167637	8.0004E-105	*MCM6*	-1.385851261	1.1248E-138
AC159540.2	2.605474397	9.47012E-45	*POLE2*	-1.381028187	9.15093E-30
GRIN2C	2.593482469	3.74551E-57	*ETV1*	-1.380567611	1.16719E-31
INPP5D	2.590495319	1.01291E-66	*MALL*	-1.377732534	3.7765E-198
LOXL4	2.579025642	4.75051E-98	*FKBP5*	-1.373701897	7.6222E-114
PHLDA3	2.578561168	1.394E-196	*GINS2*	-1.367730763	6.81672E-76
KRT15	2.57244915	0	*ARL6IP6*	-1.361573772	3.54911E-78
ALOX5	2.5698056	2.35319E-71	*CENPH*	-1.354741068	1.76389E-35
INKA2	2.547217534	8.85E-156	*PIR*	-1.353284384	3.1968E-46
AL158206.1	2.530460493	2.8359E-74	*EGR1*	-1.351023191	8.00487E-50
HPX	2.517676387	2.01549E-25	*SEMA3D*	-1.339320502	3.07063E-10
BCL2L15	2.477656979	1.6746E-24	*PDK1*	-1.33666268	1.55652E-51
ACER2	2.47458404	6.0177E-86	*MCM2*	-1.331725174	6.04647E-93
WNT9A	2.473256941	2.384E-131	*LONRF1*	-1.326967362	8.61026E-54
SUSD6	2.445301555	4.482E-244	*HSD17B8*	-1.324321131	2.40303E-24
AC093866.1	2.437765966	7.3397E-101	*ARMH4*	-1.323712992	1.47372E-07
ATF3	2.436045116	6.4003E-249	*SGO2*	-1.318869163	4.40867E-37
AL135905.1	2.424932843	7.72392E-41	*DTL*	-1.316905793	2.21056E-64
PROM2	2.423852465	4.6399E-151	*SMIM19*	-1.312455551	9.93165E-31
ALS2CL	2.414253224	1.5193E-231	*NECTIN3*	-1.311021106	4.53678E-65
SFN	2.411155189	1.2901E-239	*MANEA*	-1.309298112	3.8191E-26
FXYD3	2.405029353	7.6923E-115	*LINC02609*	-1.308747671	4.08243E-06
MIR1915HG	2.390329804	2.04307E-90	*DBF4*	-1.307182581	4.02754E-55
BTG2	2.374321019	0	*ARHGAP18*	-1.304223161	2.28391E-66
EPS8L2	2.32869544	5.1059E-283	*OLMALINC*	-1.301635943	1.05061E-58
SEMA7A	2.328529829	7.7414E-300	*KIF15*	-1.301162027	1.37475E-53
PLK3	2.312083761	1.3765E-222	*HMMR*	-1.292370654	2.41709E-19
KIAA1549L	2.309909747	1.61927E-18	*AK7*	-1.288259991	1.16492E-08
ZMAT3	2.309166777	1.9161E-288	*ACAA2*	-1.282306089	1.1382E-45
FAS	2.306371311	3.2612E-216	*TFPI*	-1.281383009	2.15098E-38
FDXR	2.302837417	2.0127E-298	*PEG10*	-1.280568612	4.54235E-44
CCN2	2.282507787	9.48247E-61	*MKX*	-1.279826514	7.88379E-40
DCLK1	2.271806014	3.72173E-37	*WDR76*	-1.279451589	5.26346E-58
TBC1D2	2.258560098	5.8041E-160	*AC092279.1*	-1.279290139	1.77672E-10
KIAA1211L	2.240070721	2.01785E-21	*CLSPN*	-1.278039803	2.43039E-68
TCEA3	2.225309433	5.24074E-37	*WEE1*	-1.277936185	1.86519E-68
FHL2	2.219893527	0	*CHEK1*	-1.277719196	3.61765E-79
PTCHD4	2.215474437	6.46356E-44	*ANP32E*	-1.268750799	5.69904E-72
SLC26A1	2.202663636	9.16924E-36	*LMF1*	-1.263041303	8.56142E-21
SERPINA1	2.183079856	9.4873E-124	*ARHGAP19*	-1.258404111	1.96534E-84
TMEM229B	2.181000101	1.17011E-29	*HSPA14_2*	-1.255036601	5.81251E-38
LAMB3	2.173966274	9.4263E-227	*PTPRN2*	-1.252924713	8.02759E-10
VWF	2.173934555	9.97607E-24	*LONRF2*	-1.252736212	1.14676E-11
ABCG4	2.17198965	2.2164E-59	*MSH6*	-1.248883291	6.44221E-95
DUOX1	2.16481998	1.4846E-118	*EGR3*	-1.248882534	1.3284E-05
STAT4	2.164490422	1.19127E-50	*ATP2B1*	-1.248826472	3.28082E-79
SESN2	2.163454188	1.9012E-195	*BMP8B*	-1.248286168	1.98172E-42
LRP1	2.156688416	1.3804E-110	*CDCA7*	-1.246479148	3.15564E-63
YPEL3	2.155005711	5.31364E-44	*ACSL3*	-1.243720118	4.6017E-103
KRT7	2.152646212	1.38758E-43	*ZFPM2-AS1*	-1.24269347	1.26725E-13
PLK2	2.149945206	0	*DPY19L1*	-1.240563574	7.58532E-65

CYFIP2	2.135120979	0	*GIHCG*	-1.233987308	6.96718E-07
IQCN	2.126297456	3.72136E-21	*IMPA2*	-1.233298973	8.62935E-51
LAMA4	2.122939148	3.98466E-42	*SRR*	-1.226880606	2.06261E-15
AMOTL2	2.119312579	0	*TAF5*	-1.226594439	8.34033E-31
CDIP1	2.104149587	1.93028E-73	*UNG*	-1.2260953	6.4685E-110
DGKA	2.103438003	0	*FBXO5*	-1.218647954	7.70892E-44
ZNF561-AS1	2.096843285	1.99858E-33	*ST8SIA6*	-1.214964791	2.06226E-14
SPNS2	2.094364619	4.82101E-91	*PSIP1*	-1.214238149	3.57292E-67
TIGAR	2.087388451	1.6016E-147	*ABHD10*	-1.212597353	2.86693E-67
NDRG4	2.079300485	8.9672E-120	*GAS2L3*	-1.211154007	5.93495E-26
SUSD2	2.073665967	9.4391E-143	*AC068831.8*	-1.210107246	4.01022E-18
ABCB9	2.072255155	1.2816E-108	*NASP*	-1.20876599	1.78549E-88
ESRRB	2.067574815	1.37258E-28	*POGLUT3*	-1.208526442	4.12641E-60
AC092683.1	2.06752541	3.29185E-20	*LMNB1*	-1.207802139	7.77543E-84
FAM210B	2.057736503	2.4137E-203	*CCNE2*	-1.207268457	9.94763E-40
NWD1	2.05504782	3.14752E-26	*MDH1B*	-1.205230677	2.97064E-06
ALOXE3	2.054843442	1.03894E-36	*CDC20*	-1.203677081	2.1571E-60
RINL	2.051405811	5.578E-210	*LSM6*	-1.203014045	3.79523E-39
DOK7	2.049311987	2.71053E-23	*ACSM3*	-1.202155841	1.15601E-33
PPM1D	2.044495644	0	*PLK1*	-1.201305199	2.83529E-68
RND3	2.038048081	2.6043E-121	*HBG2*	-1.200153077	1.88905E-06
TMEM40	2.035942634	3.0731E-88	*RHOBTB3*	-1.198530477	6.96288E-75
S100A2	2.005257607	2.31505E-55	*AC125257.1*	-1.197205363	0.006105558
LAT2	1.993974878	1.91078E-66	*OXCT1*	-1.197062834	5.89173E-53
SEMA3B	1.984753035	1.8053E-152	*STS*	-1.195548981	7.44598E-05
AL731571.1	1.97872562	2.06253E-23	*ERCC6L*	-1.192287323	7.84255E-28
PSTPIP2	1.977978342	1.5547E-257	*HSPA8*	-1.188690135	1.1796E-131
AK1	1.977116932	4.8107E-136	*CHRNA5*	-1.188216284	2.69894E-33
BLCAP	1.976040891	7.2596E-216	*KLF12*	-1.183194466	5.79726E-12
ALPK3	1.976037551	5.30843E-58	*HTR7*	-1.182423397	7.43386E-44
SLC44A5	1.974890167	1.84208E-34	*CCNA2*	-1.181850618	2.1773E-57
ZNF219	1.974376363	1.34302E-92	*MCEE*	-1.181136719	1.03349E-09
SDCBP2	1.969736363	1.47701E-15	*IFITM1*	-1.176016695	6.31857E-21
SRGAP3	1.969429761	1.79237E-78	*HAUS8*	-1.174072084	3.06356E-30
ACTA2	1.966461254	1.06252E-75	*HNF4G*	-1.17261093	3.03115E-08
EPPK1	1.95748498	1.7687E-103	*TTC39B*	-1.172505917	1.26379E-19
APOBEC3B	1.954671681	5.7469E-175	*ABRAXAS1*	-1.17110051	5.52925E-29
MIR22HG	1.950420398	4.11517E-28	*HMGB1P5*	-1.170851702	1.74223E-21
GJB5	1.943623512	3.79289E-65	*MCM4*	-1.169743741	1.1242E-114
SRRM2-AS1	1.940371497	8.6533E-13	*LRG1*	-1.16966573	2.63825E-08
KRT8P33	1.939380333	2.78577E-13	*PRKAG1*	-1.168998799	2.19281E-67
OASL	1.936590392	2.3672E-103	*AC083799.1*	-1.168978986	1.91592E-08
AC004263.2	1.935767577	7.56051E-16	*MCM7*	-1.16479894	1.9E-105
MAPKBP1	1.935572448	6.1735E-169	*FZD4*	-1.163540887	3.60303E-30
SESN1	1.935319589	3.2788E-164	*LBR*	-1.162610461	4.18368E-74
CCDC9B	1.935053319	6.8592E-138	*SPRY4*	-1.15805429	4.78905E-89
DDB2	1.931285824	4.2389E-229	*MAN2A1*	-1.154000388	1.87737E-54
RRAD	1.930680487	4.11614E-33	*KIFC1*	-1.153052428	8.69171E-51
SH2D2A	1.920798643	1.88566E-28	*MHENCR*	-1.152292419	9.61782E-06
SYNE1	1.917722282	1.86232E-50	*AURKA*	-1.151358836	1.51297E-38
AL031587.5	1.91647536	7.1035E-55	*ASF1B*	-1.151065413	6.79877E-53
HSPG2	1.912660476	2.57884E-47	*NT5M*	-1.151028556	1.37709E-11
AVPI1	1.910301234	6.6749E-113	*EFNB2*	-1.149992371	6.66556E-61
AC016831.6	1.892011025	1.97896E-41	*IGF2BP3*	-1.14599541	4.09012E-81
AF117829.1	1.878670058	3.34496E-35	*TRDMT1*	-1.145417199	1.80948E-17
COL9A2	1.876340467	6.01792E-41	*HSP90B1*	-1.143502753	5.0517E-115
ASTN2	1.875943678	1.23012E-95	*CHAF1A*	-1.142307004	2.29483E-53

LAMC3	1.873973394	1.91012E-71	*CKS2*	-1.138574683	2.62967E-65
SEMA3C	1.868841441	2.13052E-69	*DEK*	-1.134066418	6.2409E-51
DUOX2	1.867203359	8.01173E-13	*BEND3*	-1.128986771	2.28876E-23
MIR34AHG	1.864545932	7.01992E-64	*FOS*	-1.128314674	7.46081E-15
REEP2	1.863757173	9.44722E-50	*SRSF1*	-1.127523006	4.8821E-117
APOBEC3H	1.862949402	1.39557E-15	*GFY*	-1.125247303	1.99099E-08
ARVCF	1.859106001	4.119E-123	*CYB5B*	-1.122639863	1.7362E-134
ZNF425	1.848164178	5.64956E-43	*AC022400.8*	-1.121890141	1.58003E-14
AC103718.1	1.842460465	2.15616E-22	*GPR180*	-1.119895756	6.6361E-42
NLRP1	1.834240044	5.9786E-114	*SEPTIN10*	-1.118492594	9.29488E-69
ETV7	1.832390348	2.6379E-21	*MCM3*	-1.118419721	1.84491E-83
MFSD4A	1.825680844	1.1102E-16	*PTMA*	-1.117394559	2.10679E-88
LINC02086	1.824202234	2.53459E-34	*ID1*	-1.116053118	1.01679E-82
SERINC2	1.821752652	1.9267E-181	*TMEM143*	-1.11569644	3.24774E-15
CRACR2B	1.820366828	3.07461E-61	*GFPT2*	-1.114657619	6.31946E-32
RASSF6	1.81914574	5.98045E-28	*PSRC1*	-1.113576188	1.17197E-23
MAGEB17	1.817015753	5.09501E-11	*VASH2*	-1.10985886	4.58654E-09
EFNB1	1.811041733	5.7489E-142	*KIF18A*	-1.109413642	5.32819E-37
MYO7A	1.808762775	1.998E-47	*ORC1*	-1.108852326	4.18157E-30
NADSYN1	1.807416828	1.7376E-170	*LINC02458*	-1.108505706	1.49861E-08
SLFNL1-AS1	1.805652142	1.89709E-13	*KIF14*	-1.108398565	1.79518E-36
AC021066.1	1.80406635	8.3522E-12	*WDR4*	-1.107800204	1.10564E-28
VSIR	1.80401096	9.76302E-96	*MCM5*	-1.107373836	1.53491E-80
MACC1	1.800048527	3.9193E-109	*FZD3*	-1.107101098	6.19827E-24
PTPRE	1.799912124	8.7619E-147	*GXYLT2*	-1.105241962	3.77674E-09
PLXDC1	1.793656417	2.99997E-13	*CDC7*	-1.104830864	1.40736E-32
PLEKHF1	1.793528744	2.72393E-56	*IER5L*	-1.104228682	9.44322E-18
TINAGL1	1.789859321	2.1594E-159	*KNTC1*	-1.103269269	1.72131E-60
AC027290.3	1.789161252	1.86971E-18	*HMGB1*	-1.102666161	3.16052E-71
CASTOR3	1.788137192	1.04242E-55	*TXNRD3*	-1.100201224	4.82019E-31
HSD17B7P2	1.784896887	1.30603E-15	*H2AZ1*	-1.096578691	2.82148E-83
ZNF79	1.784009411	2.63941E-52	*AC126474.2*	-1.096498229	0.000206733
ADAM19	1.782403239	4.4107E-147	*MINDY2*	-1.095137256	6.50997E-35
PIDD1	1.77660075	1.0643E-116	*NSMCE2*	-1.094502794	3.91871E-26
RPS27L	1.773392904	4.6391E-113	*RPL10A*	-1.093757417	1.8948E-101
EFR3B	1.772879749	5.81535E-19	*PARVB*	-1.093477	1.37114E-37
TSPAN10	1.771159628	3.11581E-18	*FAM20C*	-1.093396305	4.9896E-27
MAP3K12	1.767338477	3.0445E-138	*TMEM37*	-1.092548557	2.91671E-07
IGDCC4	1.765796756	6.07534E-21	*ABCB10*	-1.092289154	2.30741E-50
ANKRD33B	1.765543221	2.3777E-132	*AGA*	-1.091244732	1.2103E-08
PGF	1.764761805	2.2253E-52	*IL1R1*	-1.09124327	4.3514E-18
LYNX1	1.763097668	3.2183E-48	*CKMT1B*	-1.088626678	2.82049E-36
IGFBP6	1.759669025	1.1136E-112	*PRKAR2B*	-1.087342056	2.4982E-41
KLK7	1.755172044	6.8267E-156	*CDC25A*	-1.08602979	2.25988E-47
TNFAIP3	1.754923035	3.49738E-53	*POLA1*	-1.084817018	1.05938E-56
NR2F2	1.753915132	2.2628E-228	*MAD2L1*	-1.084419078	5.19184E-50
MXRA8	1.752787615	7.7599E-15	*KIF11*	-1.082842067	5.14773E-32
ABHD4	1.7448456	1.13116E-79	*LYPD6*	-1.081367114	8.10395E-13
LINC00680	1.743973186	3.48381E-14	*ARRDC3*	-1.081044855	1.42939E-10
CASZ1	1.742418144	4.34466E-67	*MCM10*	-1.080837838	1.50523E-41
SLC31A2	1.741841518	3.97115E-24	*B3GLCT*	-1.077986627	4.70645E-22
LCAT	1.735320972	1.0196E-26	*CLMP*	-1.077748276	7.921E-10
PLCL2	1.734706881	2.90221E-38	*UGT8*	-1.076567192	3.06547E-40
AC093827.4	1.732294412	6.06135E-11	*NUP35*	-1.07519846	8.55854E-35
TRPM6	1.72676107	2.29061E-12	*STARD8*	-1.075066267	2.69918E-11
LACC1	1.725186631	4.02276E-63	*HASPIN*	-1.074676141	5.22061E-22
CHRNB1	1.714400556	3.9213E-146	*RHBDL3*	-1.07439732	8.74472E-16

TMEM139	1.708941173	2.59051E-21	*MBOAT1*	-1.073707477	2.10148E-31
NUAK2	1.705104518	2.23696E-54	*ZBED8*	-1.073071668	2.47482E-13
NRG1	1.703292002	5.41653E-20	*AC012146.1*	-1.072658748	1.48752E-05
ARX	1.699312611	5.31161E-13	*MAGOHB*	-1.06976307	2.3113E-22
MR1	1.69926209	9.74909E-93	*NEDD4*	-1.069690469	4.29426E-26
COBLL1	1.695072491	1.77097E-70	*KLRC2*	-1.06675	2.88252E-09
BAX	1.695047474	2.70411E-95	*CASC2*	-1.06453968	4.33004E-07
TGM1	1.694770918	1.83863E-16	*SPDL1*	-1.060447725	7.57775E-34
INAVA	1.690349885	7.81518E-65	*CKLF*	-1.060216796	5.28437E-20
EIF3CL	1.690110514	0.017952025	*TRA2B*	-1.059644462	2.5859E-109
GADD45A	1.687957727	2.0248E-254	*MIR210HG*	-1.059135392	2.57777E-06
DUSP14	1.687274686	7.3374E-148	*ZDHHC6*	-1.058649925	1.04291E-64
RHOD	1.687221457	1.74103E-95	*TDP1*	-1.058319752	5.98563E-50
BBC3	1.684451593	1.05902E-72	*MND1*	-1.058152077	1.25957E-16
MAMDC2	1.682047452	7.23875E-22	*KCNS3*	-1.056458669	2.36403E-21
HYAL1	1.68081436	3.76396E-48	*SAMD1*	-1.055568232	8.2759E-35
ADGRG1	1.677549588	2.8023E-140	*HSPE1*	-1.053903147	5.16917E-15
ABHD8	1.673895111	4.49756E-24	*TEX30*	-1.052663399	6.13875E-28
MDGA1	1.673634234	3.78798E-66	*HMGB1P6*	-1.050803781	2.61736E-06
SLC2A1	1.667097401	4.6631E-155	*ZNF775*	-1.04966529	9.05755E-09
AL138756.1	1.666953218	1.22847E-22	*GINS3*	-1.048479725	5.27568E-28
KIF26B	1.660551172	1.21942E-13	*RP9P*	-1.048397591	4.64233E-12
AP002761.4	1.657429596	1.09605E-24	*CASP8AP2*	-1.046658508	1.35905E-28
RPL23AP53	1.65478432	1.2023E-12	*MEX3A*	-1.044309013	1.03546E-26
SLC2A12	1.651146098	1.33834E-72	*BUB1*	-1.04395937	5.83192E-39
VWCE	1.648115176	1.27719E-09	*DPYD*	-1.043166621	1.1157E-05
HHAT	1.64560143	8.92997E-45	*SRSF2*	-1.041894012	6.4927E-128
NOP14-AS1	1.643147943	7.30105E-69	*VWA5A*	-1.041296126	2.08185E-14
MICALL1	1.642094093	6.45009E-97	*MTAP*	-1.040499648	3.45813E-70
FBLIM1	1.637297368	1.51339E-54	*UBE2T*	-1.039931537	3.86039E-39
LIMS2	1.636488519	7.67557E-11	*OCEL1*	-1.039827892	2.23952E-28
NINJ1	1.635194513	1.26482E-68	*WWC3*	-1.038157383	2.74352E-19
GREB1	1.634841498	5.36531E-31	*VSNL1*	-1.036305261	2.78501E-34
PORCN	1.633473533	9.48406E-67	*ORC6*	-1.034569109	3.05117E-47
SRPX2	1.632957969	2.24791E-32	*PPP1R3E*	-1.033893009	6.59346E-07
MTND1P23	1.631935559	0.003581427	*CDC45*	-1.033249429	1.19594E-33
CACNA1A	1.631108367	8.50829E-29	*ALMS1*	-1.031276217	6.46469E-33
LGALS9	1.63078931	1.53686E-14	*RASSF8-AS1*	-1.030918421	0.000100323
ISG15	1.629850815	2.3096E-128	*PDS5B*	-1.029374294	1.21376E-29
IFI6	1.629349845	7.55688E-59	*PTPRG*	-1.029001898	1.56905E-39
PHYHIP	1.628269381	2.4707E-27	*AC127502.2*	-1.028701544	0.000171344
PRF1	1.625704257	2.86844E-28	*BUB1B*	-1.028609419	4.39107E-40
SLC37A2	1.625032076	3.61511E-63	*CHDH*	-1.027806417	6.23672E-34
TRAF4	1.624642854	6.0987E-157	*TMED7-TICAM2*	-1.026905839	5.03721E-22
FMN1	1.621426868	3.13706E-14	*MIS18BP1*	-1.026117712	2.32573E-27
UNC13D	1.619743278	6.15765E-61	*TCFL5*	-1.02593444	1.24748E-33
STAG3	1.617522863	6.48828E-14	*PHLDB2*	-1.02529635	3.51535E-97
BTBD11	1.617494359	5.55772E-59	*TMBIM4*	-1.024089108	2.3634E-22
ADAMTS7	1.616583191	1.75938E-79	*STRADB*	-1.022862935	8.76599E-24
ITGAX	1.615457674	3.19663E-24	*TMPO*	-1.021498431	1.52512E-91
NABP1	1.614152904	5.32857E-67	*ATAD2*	-1.018486781	2.27427E-44
AC016588.2	1.613459896	3.36921E-19	*HS3ST1*	-1.018056783	1.62501E-56
PLCXD2	1.613343662	4.5136E-136	*CCDC88A*	-1.017703287	2.72282E-26
AADAC	1.612548665	1.45357E-15	*CU633906.2*	-1.015494978	0.010370234
GM2A	1.611094838	3.1219E-208	*EGLN3*	-1.014487756	1.07307E-14
AJUBA	1.610935996	9.8615E-157	*LPCAT2*	-1.014213068	1.11187E-27
RRM2B	1.607256709	5.9913E-102	*FAM81A*	-1.010203689	5.63555E-13

HBEGF	1.606875558	5.39042E-55	*PELI2*	-1.008414539	1.1451E-05
WDR66	1.60617571	1.70798E-16	*EBF2*	-1.007918845	6.72004E-06
AC016831.1	1.602803955	4.03229E-31	*NDC1*	-1.007314698	3.78914E-65
RGS20	1.602653035	2.84155E-31	*PDE3B*	-1.007065765	7.57665E-21
AC148477.2	1.602191351	4.72716E-18	*BARD1*	-1.00646745	3.4842E-27
PARP10	1.600197596	8.38724E-26	*MIR100HG*	-1.004249199	1.40186E-53
IDUA	1.599054528	1.26357E-45	*C3orf80*	-1.003804808	5.6509E-06
PEAR1	1.598088885	1.81095E-83	*CCNB2*	-1.002854566	9.88649E-33
ANKRA2	1.595673932	3.20526E-81	*NRM*	-1.002580111	1.54108E-12
LINC02591	1.594750555	3.97778E-12	*G2E3*	-1.001821874	6.41572E-30
FRMD4A	1.594745952	1.22893E-13	*IDI1*	-1.001532885	1.25843E-42
STON2	1.588827157	8.38339E-15	*MARC1*	-1.000538968	1.61264E-23
PKD1L2	1.585086583	4.69954E-28	*ITGB3BP*	-0.999524846	4.30302E-30
MAMDC4	1.582833662	4.26566E-37	*DUT*	-0.999212757	2.42998E-47
FADS3	1.582358123	1.4649E-119	*CU634019.6*	-0.99640058	0.000389062
SAXO2	1.580352543	8.56663E-10	*RORC*	-0.995582562	3.20745E-05
INKA1	1.579767973	1.27416E-10	*HSPD1*	-0.994671461	3.46461E-77
SDC4	1.574445714	2.5349E-254	*IPO5*	-0.993631214	4.8494E-69
CRYBG2	1.574210756	6.79454E-30	*SLC16A9*	-0.991472512	1.6616E-17
LHX5	1.571757869	2.66911E-35	*HFE*	-0.990991818	1.24907E-21
CARNS1	1.564395092	2.58884E-10	*SRSF7*	-0.990406598	2.85046E-57
GAB2	1.556630079	3.83794E-23	*ZRANB3*	-0.988573533	4.01416E-14
ATG16L2	1.556118357	3.80005E-82	*ZBTB14*	-0.987910812	9.42864E-20
SSPO	1.552684422	5.31043E-24	*PALM3*	-0.987448399	2.32128E-18
KCTD11	1.551381682	2.89965E-52	*JPH1*	-0.987377062	6.05096E-30
KIF17	1.550591468	1.36511E-15	*NIPSNAP2*	-0.987068912	8.88715E-63
RIN1	1.549552581	3.15134E-78	*ACYP1*	-0.986292575	4.1384E-19
CES2	1.549402911	6.4261E-153	*ZNF165*	-0.985631911	5.91899E-14
TTLL6	1.547716399	2.25646E-15	*FGD5-AS1*	-0.985362047	1.46343E-72
MYEOV	1.547441539	5.2289E-150	*KIF20B*	-0.982817142	8.30668E-25
LINC02323	1.545842982	5.31834E-08	*USP28*	-0.982385734	3.816E-36
PCDH1	1.543807748	8.7351E-103	*GALNT3*	-0.981766697	1.63723E-21
AC080112.2	1.542668509	7.47277E-11	*ARL13B*	-0.980827388	4.17218E-15
ARHGAP40	1.537601129	4.47879E-11	*ARHGEF39*	-0.980790795	1.05001E-25
CDC42BPG	1.534743073	4.56636E-80	*METTL25*	-0.980595089	9.22414E-06
DUSP10	1.534239096	3.42619E-18	*AC092718.4*	-0.980321011	3.34643E-07
DPYSL4	1.520811419	5.21626E-23	*HOOK1*	-0.979987799	1.30308E-32
TRIM54	1.520416584	4.32881E-13	*SSX2IP*	-0.979951913	4.82539E-40
ITGB4	1.519634906	2.7035E-141	*ZBTB2*	-0.979425208	2.71868E-22
SLC12A4	1.51961249	1.2257E-100	*ASPM*	-0.979102444	6.34125E-32
HLA-DOA	1.515267901	2.08084E-13	*PASK*	-0.979021791	3.70368E-29
AC055811.4	1.512759784	8.56791E-16	*PRKCQ*	-0.977559647	0.000148397
VMAC	1.508813908	9.26805E-10	*LFNG*	-0.97710841	6.42018E-12
KLLN	1.507908677	4.32498E-21	*KIAA0319*	-0.976922436	0.000153505
GSDME	1.506608099	1.04342E-82	*BORA*	-0.976736702	1.4848E-22
CRIP2	1.50522435	2.08314E-59	*TMEM209*	-0.976220346	4.20993E-43
IKBIP	1.504807748	2.7454E-108	*POLD3*	-0.975414559	2.33936E-22
TSPAN14	1.496112205	1.3198E-226	*SLC1A1*	-0.974892254	1.25937E-18
EPHA2	1.495995232	1.6667E-167	*HAUS6*	-0.974587943	3.1968E-46
FAM214A	1.49554805	4.40059E-36	*GCNT1*	-0.973471535	1.60057E-13
LMO7	1.494326143	6.43887E-52	*RHEBL1*	-0.973248501	0.000861386
TRIM3	1.493787043	8.92557E-69	*ATAD5*	-0.973211028	3.70673E-18
RGS16	1.490627564	1.70717E-32	*DCK*	-0.973188895	3.08647E-34
TTYH3	1.488376355	4.52382E-76	*FEN1*	-0.972902034	1.02726E-50
VCAN	1.488330683	1.26195E-93	*TMCO3*	-0.972700218	2.52382E-54
AHNAK2	1.487296154	1.23647E-91	*ZNF700*	-0.972080056	4.493E-17
ALOX12P2	1.48527848	1.46779E-17	*CENPE*	-0.971809042	5.86798E-07

PRAG1	1.48338766	2.35399E-77	*P4HA1*	-0.970764257	3.46569E-42
TSPAN1	1.482130274	3.5689E-109	*RNASE4*	-0.966709994	1.64494E-06
EPN3	1.481167872	1.55504E-78	*BIRC5*	-0.965361266	1.58634E-66
SYTL1	1.47758201	6.50652E-80	*STIL*	-0.965054045	5.79369E-36
HOGA1	1.477084865	3.16129E-27	*C6orf141*	-0.965017674	4.88313E-11
TMEM51-AS1	1.475580963	6.61476E-32	*STAMBPL1*	-0.964352142	1.81329E-14
RAB27B	1.475130433	7.76642E-48	*PMP22*	-0.960045731	7.1629E-39
FER1L4	1.469041377	1.73634E-49	*CHRAC1*	-0.95967671	1.01072E-48
MEAK7	1.469012153	5.922E-164	*SLC7A2*	-0.959115959	6.51346E-16
AL117339.5	1.465525339	2.45377E-09	*ZNF443*	-0.958755614	4.26487E-06
PI4K2A	1.463259425	4.9956E-126	*BLM*	-0.95679635	5.78794E-26
LINC02405	1.462398209	1.50845E-08	*HADH*	-0.956582865	2.35809E-41
COL13A1	1.460967005	1.67681E-76	*NAT16*	-0.955248057	0.000338542
TTN	1.456965269	1.05215E-07	*DPYSL3*	-0.95468969	4.69385E-31
UBR5-AS1	1.456810227	1.69845E-11	*PLEKHO1*	-0.953913783	1.40831E-07
AL589743.1	1.45651886	6.62151E-33	*CDC25B*	-0.953835792	1.27425E-53
C2orf88	1.456394356	4.4573E-17	*SNRPD1*	-0.950695056	6.4005E-52
OSBPL7	1.456217711	1.41047E-79	*KCNK5*	-0.950244404	2.45589E-18
AC067930.5	1.45567669	6.34007E-19	*RPL3*	-0.949155194	6.0479E-131
ANKRD19P	1.454882952	1.60801E-49	*HOTAIRM1*	-0.949106331	0.00035811
SDSL	1.452535425	2.98973E-52	*CCNB1*	-0.949068838	4.82539E-40
PLCD3	1.451406565	1.7129E-113	*GNG7*	-0.948083863	7.27092E-22
COL7A1	1.451139472	2.70743E-42	*PBK*	-0.946845093	3.58778E-38
ANKRD24	1.448995673	5.3555E-27	*TSHZ1*	-0.945102697	1.41513E-17
AC132219.2	1.448754854	2.1422E-08	*HOXA13*	-0.945085597	1.26244E-15
TMEM63B	1.446341654	6.2962E-165	*SCMH1*	-0.944955756	1.00983E-21
KIAA1614	1.446067808	8.99073E-11	*RFC5*	-0.94375036	2.14414E-57
AC245452.1	1.444159858	1.24431E-12	*LCORL*	-0.943591903	7.28709E-25
C6orf52	1.441620911	5.63293E-06	*STOX1*	-0.942739612	2.96898E-11
FGFBP3	1.438510867	4.29881E-10	*NADK2*	-0.942651936	3.06763E-23
ZNF600	1.438081042	3.84698E-64	*FZD1*	-0.942612813	2.21476E-16
FOXD1	1.436707545	2.98795E-47	*ANKRD36C*	-0.94247832	1.44356E-08
TRIM8	1.431458952	2.5238E-116	*JADE1*	-0.942167715	3.43108E-33
ZNF843	1.431312776	1.87803E-11	*RBBP8*	-0.94195448	5.02397E-62
PSORS1C1	1.431275801	1.06801E-11	*CNPY4*	-0.941740896	0.000954845
PLXNA2	1.431120017	6.68016E-09	*ENOX2*	-0.941668307	2.86486E-15
TP53INP2	1.429815605	1.82038E-41	*LEPR*	-0.940595105	7.24781E-19
MORN4	1.422292978	6.60742E-31	*POLR3G*	-0.939253028	1.51741E-26
F3	1.42213708	2.3686E-171	*CADPS2*	-0.938934479	7.88468E-22
PABPC1L	1.420340098	3.16484E-89	*AUNIP*	-0.938887628	5.04189E-17
COL9A3	1.420206779	4.2566E-63	*REXO5*	-0.938757378	3.91343E-19
AC138150.2	1.417808416	7.08372E-08	*CYP3A5*	-0.93866045	4.54115E-09
CORO6	1.413880373	3.05318E-53	*E2F1*	-0.93736222	1.27886E-23
CSF1	1.412687519	7.54305E-34	*KLHL15*	-0.93573204	1.80958E-24
SEMA3G	1.412451973	1.33669E-30	*LIFR*	-0.935511438	2.14828E-20
MMP28	1.41110957	7.5293E-10	*CALML4*	-0.935323385	0.000524495
MATN1-AS1	1.408721383	6.36455E-12	*FNIP2*	-0.934727899	2.53162E-09
ANO9	1.407531562	1.39256E-76	*XRCC2*	-0.934601442	1.49789E-25
NCEH1	1.407409841	1.52966E-82	*NUBPL*	-0.933354917	1.46782E-14
SLX1A	1.405456266	2.09965E-08	*DGKE*	-0.931532935	1.30625E-10
IQANK1	1.403799405	8.73149E-81	*HOXC8*	-0.930937536	0.00508269
PHPT1	1.403394829	3.17211E-68	*NR5A2*	-0.930205683	7.48449E-13
TNFRSF10B	1.402471667	8.0942E-264	*PIK3R1*	-0.930093093	1.21877E-21
TCN2	1.400292655	5.47424E-18	*TUBD1*	-0.92963218	1.54055E-19
ST6GALNAC5	1.394235992	1.06956E-21	*COQ2*	-0.929179935	7.20237E-21
MIRLET7BHG	1.393700984	3.44257E-15	*LINC00997*	-0.926497133	0.001495122
PCNX2	1.392521678	1.26985E-65	*GNE*	-0.926208897	2.43714E-33

PRSS27	1.391795013	2.38291E-08	*SEC23A*	-0.925356083	1.13419E-60
FXYD1	1.391126891	1.68547E-05	*CNOT6L*	-0.924019988	2.16834E-24
LINP1	1.389236898	2.62247E-41	*FZD7*	-0.923085683	7.74608E-22
LCN2	1.388593273	3.00738E-63	*SLC44A1*	-0.922629573	1.19499E-50
SAMD9	1.387269628	1.42449E-31	*FKBP4*	-0.922629016	2.17334E-65
ARAP1	1.387204411	2.6317E-111	*H2AW*	-0.92255624	1.2285E-07
PPP1R14C	1.385740949	1.06971E-81	*AGPAT5*	-0.922335531	4.46859E-39
SNAI3	1.385396215	1.74095E-17	*SUV39H1*	-0.922209237	8.10434E-20
UCN	1.381919568	3.13309E-06	*AC027228.2*	-0.922165011	8.97806E-06
P2RY2	1.379178403	5.72319E-26	*CENPA*	-0.92031487	2.83528E-20
ADAMTS16	1.378946705	7.34955E-09	*NEIL3*	-0.9201983	3.20364E-20
RNF182	1.378893322	4.03258E-48	*ZNF92*	-0.920193385	4.64639E-16
APLP1	1.377572004	1.61797E-74	*SUCLG2*	-0.919894867	1.40903E-50
LINC01521	1.375425688	1.17028E-07	*NRTN*	-0.919137018	3.796E-06
C6orf132	1.365462505	7.90253E-85	*USP37*	-0.918401951	4.55231E-40
ADM2	1.364300279	4.35676E-43	*TRIM59*	-0.918062059	4.1387E-21
RAPGEF3	1.363622431	1.47875E-13	*PRIM1*	-0.916773345	1.13298E-26
PGAP1	1.363440832	3.52654E-34	*NFIB*	-0.915809834	1.04935E-26
ABALON	1.361934764	1.02726E-50	*ING2*	-0.915780556	2.70104E-08
AP002026.1	1.36182932	1.66443E-15	*RFC4*	-0.914804813	7.05035E-43
CPE	1.359696482	9.2365E-107	*CACYBP*	-0.913496156	2.06352E-60
AC026471.1	1.359182635	1.66738E-15	*HSPA5*	-0.910647728	1.7896E-159
HSPA4L	1.358218716	2.4283E-93	*CBX3*	-0.908757724	4.17232E-40
NTPCR	1.357301958	2.2024E-100	*OARD1*	-0.908062516	5.60137E-25
MANCR	1.355047477	2.28408E-09	*EAF2*	-0.905664518	2.00007E-05
PIFO	1.352877568	6.11511E-07	*ZBTB12*	-0.905037998	3.67267E-11
CASC8	1.35253878	6.77295E-11	*ZNF280D*	-0.904250347	1.00371E-26
RBMS2	1.350413149	1.156E-108	*HLF*	-0.903485922	6.76014E-06
ZFYVE1	1.350361775	3.82741E-56	*KIF23*	-0.903466928	5.27755E-29
RTL5	1.350193363	5.5174E-37	*HPF1*	-0.903074146	4.92293E-23
AC018629.1	1.350037781	3.9124E-10	*BUD13*	-0.903007836	9.85719E-23
SMAD7	1.3462419	2.08146E-16	*NEDD1*	-0.902556692	3.52229E-28
PLAT	1.344352113	1.77193E-56	*FERMT2*	-0.901359596	2.48517E-32
NPR2	1.342408812	5.43317E-24	*SRSF3*	-0.900228889	8.98731E-74
CEMIP2	1.340374993	4.9039E-115	*SKP2*	-0.900071399	5.5701E-37
NEURL1	1.340224205	1.31583E-11	*YPEL1*	-0.899615369	0.002087101
AC015712.2	1.339948183	3.69998E-63	*DEPTOR*	-0.89956964	1.62209E-11
PPP1R3F	1.339685926	3.09148E-09	*CBX2*	-0.899099633	4.69175E-42
ABAT	1.338972787	5.19975E-27	*AC021092.2*	-0.898929438	2.42483E-06
AC112220.2	1.338236872	3.83081E-10	*GLCE*	-0.898847149	2.27075E-32
ISG20	1.337715769	2.55933E-17	*PAXIP1*	-0.897537542	1.29505E-30
TGFA	1.336270956	8.6816E-116	*NSD2*	-0.89717157	1.28547E-58
LINC02298	1.335982194	4.11299E-12	*ING1*	-0.896888189	6.02011E-32
PLEKHM1P1	1.334845101	5.09586E-34	*RPUSD4*	-0.89646764	1.69605E-42
ST20-AS1	1.334689831	3.74327E-08	*SLC25A24*	-0.895306915	3.31294E-30
KLK6	1.33451772	8.64147E-81	*SMC4*	-0.895302157	8.07471E-38
SPRED3	1.327690158	5.20658E-11	*PDE9A*	-0.89477555	2.05954E-35
LPIN3	1.326395259	4.58818E-64	*TTC21B*	-0.894017546	1.87547E-14
FCGBP	1.325835814	1.61993E-23	*IMPA1*	-0.893976991	2.10579E-34
DRAM1	1.320689537	3.12082E-78	*GINS1*	-0.893869325	5.26442E-41
BLOC1S2	1.320191772	1.60945E-87	*TAGLN2*	-0.89326409	1.17453E-62
AC005674.2	1.319797781	3.42392E-16	*SAAL1*	-0.889683513	1.65656E-27
PXDN	1.319522629	3.78172E-85	*HSD17B11*	-0.889263444	3.54715E-38
IFRD1	1.317594538	1.26682E-63	*MPP7*	-0.88841639	2.79561E-26
NALT1	1.313056134	1.66794E-08	*PSMC3IP*	-0.887592618	1.47564E-16
SCNN1D	1.31257074	7.42878E-17	*NAP1L1*	-0.886968906	2.84604E-60
COL12A1	1.312004961	2.02772E-93	*TMEM135*	-0.886630221	7.38437E-26

PI4KAP2	1.311237216	2.59228E-55	*NCAPG*	-0.886312366	1.67015E-27
AGRN	1.310961609	3.85262E-80	*CFL2*	-0.885756066	9.09984E-35
MEGF11	1.309382811	7.61836E-14	*C5orf30*	-0.885720987	7.83489E-19
RNF144B	1.308977754	1.02008E-14	*EPM2A*	-0.885212598	1.10028E-08
CCDC88B	1.30854635	8.35175E-36	*EPHX2*	-0.885029983	3.92981E-23
KRT8	1.304177733	9.1595E-159	*CTDSP1*	-0.884807736	2.41473E-43
LINC00174	1.303504117	7.11639E-24	*PMS1*	-0.884333559	3.95527E-31
SYK	1.303375808	2.30544E-42	*AC097448.1*	-0.88432753	1.18017E-11
RNF19B	1.302906617	5.09894E-83	*LRRC8C*	-0.883967855	4.69047E-24
PI4KAP1	1.301119631	1.4251E-34	*FAM72C*	-0.883011968	3.48003E-11
DEGS2	1.300521862	5.32712E-07	*LARP1B*	-0.882543944	2.64821E-28
AQP3	1.299271547	8.46844E-15	*SMC3*	-0.882414747	3.72863E-41
KDM4B	1.298215224	3.06558E-77	*PPA2*	-0.88198405	3.42593E-28
PDGFB	1.295587573	3.52911E-78	*SMCO4*	-0.880472711	3.29647E-13
RFNG	1.294920889	5.523E-66	*AC010733.2*	-0.880412135	3.74782E-05
LINC01405	1.292186702	8.28685E-25	*SASS6*	-0.87959313	6.99005E-18
FLNC	1.287682447	2.2283E-100	*EXO1*	-0.879509341	7.13283E-22
AC037459.2	1.287181975	5.13418E-09	*RUNDC3B*	-0.878717093	1.63491E-06
RTCA-AS1	1.285570097	4.48324E-06	*OGDHL*	-0.878627421	4.69421E-13
RBP1	1.284029173	4.17316E-66	*FOXRED1*	-0.878539024	3.34562E-30
RPL23AP82	1.283913439	1.21058E-69	*RGS17*	-0.877287464	3.32491E-08
AC122688.3	1.281494508	4.54857E-09	*RAB27A*	-0.876439998	5.51226E-14
POLH	1.276854249	6.1246E-100	*POPDC3*	-0.876305068	4.21014E-08
ZNF707	1.276838736	4.90745E-34	*XXYLT1*	-0.876088598	5.05478E-09
ID2-AS1	1.276259639	1.15475E-11	*HAT1*	-0.875937348	1.40239E-40
DNAJB5	1.273780537	2.02433E-26	*YEATS4*	-0.87477872	4.61246E-23
DOCK3	1.272339461	8.64283E-48	*BCL2L12*	-0.873128481	3.25182E-18
NDUFAF8	1.271310917	8.98276E-42	*CNTLN*	-0.871863495	1.13988E-11
EMX1	1.270898481	1.5465E-11	*PARP2*	-0.871664921	1.06518E-39
AEN	1.269675633	3.8868E-102	*HNRNPA1*	-0.871424698	2.62337E-72
MST1P2	1.268403772	1.14351E-10	*KANK2*	-0.870708243	1.41289E-27
CDK5R2	1.267592586	8.57717E-09	*H1-10*	-0.870403134	1.06808E-43
CHST6	1.265503908	3.18725E-87	*UGDH*	-0.869624547	7.20608E-39
IRF7	1.265481285	1.46462E-36	*DPYSL5*	-0.869225579	3.58047E-17
ARHGAP23	1.26526859	6.75609E-68	*TXNDC5*	-0.868127822	1.7639E-104
AC108676.1	1.264711605	1.86133E-10	*C1orf112*	-0.868096118	3.79257E-12
GJB3	1.261332592	5.96289E-58	*RPS6KA3*	-0.866984923	3.72465E-42
MARCHF2	1.260548513	4.93567E-39	*HNRNPH3*	-0.866575508	6.77094E-40
CICP14	1.25669988	7.2871E-20	*DRAM2*	-0.866573596	4.12332E-30
CGB7	1.256293308	7.07529E-11	*C6orf136*	-0.866506416	2.47054E-15
GPRC5A	1.254062906	1.6851E-133	*IFT88*	-0.866345017	1.475E-15
KSR1	1.253094207	4.97908E-52	*USP1*	-0.865478882	3.77111E-34
COL4A5	1.253076463	3.51647E-73	*ANK3*	-0.864799919	4.65087E-13
NIPAL3	1.252968802	3.33375E-92	*MSANTD3-TMEFF1*	-0.863719369	0.001085618
FYN	1.250631408	1.61793E-41	*PRC1*	-0.862357347	2.52466E-49
NOTCH1	1.250539523	1.80311E-55	*C10orf95*	-0.862079211	0.00346651
SUGCT	1.249304706	6.01383E-20	*FAM72B*	-0.861934088	3.15439E-09
CLN8	1.246861471	7.24268E-75	*DSCC1*	-0.86158086	4.39721E-37
INPP1	1.246440118	6.31283E-77	*NRARP*	-0.861329027	2.51407E-24
ARSA	1.24643696	1.02599E-37	*KLHL23*	-0.861077535	7.30189E-39
PLXNB1	1.24542943	8.23689E-89	*NCAPD3*	-0.860105582	1.81428E-43
CALCOCO1	1.245252504	1.13432E-64	*SLC35G1*	-0.860000099	2.6283E-18
MAP3K14	1.245202591	2.23466E-45	*PDLIM5*	-0.859303448	9.29257E-52
PRKAB1	1.243411946	7.81653E-50	*EXTL2*	-0.857617257	6.10621E-23
COL11A2	1.242826966	9.23572E-41	*SP4*	-0.857453927	7.10681E-15
HDAC9	1.241567776	3.3565E-13	*PIGBOS1*	-0.856249926	3.34526E-08
DAGLA	1.240696126	2.44201E-28	*ISOC1*	-0.856037967	4.20554E-35

LMNTD2-AS1	1.240587061	2.5794E-16	ATG10	-0.855621758	1.20397E-08
SMTNL2	1.23986983	9.5423E-19	PTBP1	-0.855339907	2.88581E-49
PRSS30P	1.239550958	9.12222E-23	TTK	-0.854550461	4.65814E-26
PHLDB3	1.238208083	4.44492E-23	RHOBTB1	-0.854013103	8.46576E-07
SAMD4A	1.237885098	6.84759E-34	SGO1	-0.853649083	9.79734E-15
GAL3ST4	1.237800638	2.28464E-10	RMI1	-0.853464272	3.42058E-27
SLC4A11	1.236462338	1.00496E-43	TESC	-0.853339102	8.50936E-20
TAP1	1.236390051	3.3074E-133	TOPBP1	-0.852095432	2.02214E-44
SPRY1	1.235896818	2.86609E-27	PTGES3	-0.851495231	4.59333E-42
RAB3B	1.233125162	3.3497E-69	STX2	-0.85100044	1.68696E-25
LAMC2	1.231735832	7.8145E-110	NKAIN1	-0.850923943	0.001245249
CEP85L	1.231104476	4.00387E-20	RAB31	-0.850578887	1.47286E-35
PGPEP1	1.227587285	2.65893E-95	MALSU1	-0.849104877	2.71013E-26
GPX1	1.226762798	5.45144E-88	ANP32B	-0.847806497	3.56955E-49
SOCS1	1.222229909	9.60587E-10	AC073857.1	-0.84504431	0.000854389
HSPB8	1.22142858	3.24134E-12	PI4K2B	-0.843838872	2.87752E-23
CSTA	1.220154137	1.13922E-11	CKMT1A	-0.843394326	8.98867E-17
DGCR11	1.219177092	1.30751E-06	SQLE	-0.842710431	7.95887E-44
CASTOR2	1.219112723	5.60764E-34	ZNF799	-0.842682034	1.40237E-06
MRVI1	1.218936754	8.3311E-53	USP13	-0.84219896	1.13042E-34
ADGRB1	1.217365412	8.2664E-15	CENPJ	-0.841896972	9.79313E-26
CCDC84	1.216776371	8.6752E-35	ALDOC	-0.8417024	5.62081E-29
SERTAD1	1.216080474	8.5966E-34	FH	-0.841587247	6.84356E-43
MAP2	1.21567141	2.4707E-27	RAD21	-0.841376948	2.28578E-39
GPR135	1.215407496	6.06476E-07	PCSK9	-0.841337196	5.66259E-39
NEURL1B	1.214894765	1.12806E-57	PARP16	-0.840768964	4.37591E-10
AP003419.1	1.214310377	0.029823577	USE1	-0.840584305	3.62805E-13
HAP1	1.214103087	1.81451E-29	SENP1	-0.840172405	4.97285E-20
AC022167.2	1.213803899	6.15765E-07	CCDC61	-0.84007573	0.000123619
LRP2BP	1.213433675	8.78094E-12	PIF1	-0.837212596	1.48715E-10
SEMA3F	1.212876454	2.87822E-39	SPATA33	-0.836783856	8.13883E-13
AC078993.1	1.212507583	1.87721E-09	NFIL3	-0.836494468	7.01395E-26
STK17A	1.212367082	1.11402E-80	CDCA7L	-0.835775806	5.81666E-48
HERC2P3	1.211925707	2.19732E-27	GPR160	-0.835368836	9.97987E-11
CA2	1.211685217	9.42681E-98	SOX4	-0.834858499	3.45534E-61
TEP1	1.21159975	9.54749E-97	C19orf81	-0.833945052	0.00557394
IZUMO4	1.211400249	5.66917E-11	EMP3	-0.833333097	5.92538E-15
SLC48A1	1.211334232	7.20439E-70	PTPN18	-0.832617084	2.39724E-20
TOB1-AS1	1.210068155	2.01721E-07	SCML2	-0.832337125	3.10674E-10
AP003068.2	1.209334128	2.73556E-15	TCAF1	-0.832195812	7.11019E-45
SNTB1	1.208928804	4.27367E-21	HELLS	-0.832092727	1.41709E-36
MSX1	1.207117325	2.68509E-73	REEP1	-0.831941124	1.98279E-16
GDPGP1	1.207036097	3.13338E-24	NUCKS1	-0.831121667	1.08109E-44
SMTN	1.206030274	3.96487E-69	SULT1C2	-0.830698672	5.42701E-06
PTPRU	1.205417813	5.14752E-71	CDCA8	-0.830185727	1.26672E-23
DUBR	1.203133488	2.82835E-10	MIPEP	-0.828396142	6.18673E-22
AL606834.1	1.202502182	2.8664E-08	CCDC18	-0.828372352	2.75607E-11
RIOK3	1.201835916	2.42892E-64	HJURP	-0.82759319	2.35795E-24
AP002478.1	1.200793368	4.58654E-09	RBM3	-0.827449326	1.82226E-54
SERF1A	1.199219473	0.011511478	ERI1	-0.827139529	1.09815E-33
FN1	1.198389791	1.06354E-32	ATP11C	-0.826637297	3.23993E-28
MGLL	1.197566338	1.55287E-55	RESF1	-0.826467437	8.24072E-35
SLC25A45	1.196934777	7.02862E-11	AC083843.3	-0.825549512	1.32215E-06
TUSC2	1.196876263	2.18582E-54	TEAD2	-0.825179284	1.36797E-23
ELF3-AS1	1.195141233	2.39138E-16	PEX11A	-0.824698509	4.29367E-10
MMP14	1.194781069	5.15857E-73	SLC38A2	-0.82429937	2.04995E-34
TRIP6	1.194532833	4.16719E-69	FAM222A	-0.823336213	2.46854E-07

PSD2	1.19346967	8.69562E-09	*RBL1*	-0.822976766	3.51078E-16
TEX9	1.191837599	5.89989E-20	*LMAN1*	-0.82140743	6.24564E-38
AC018665.1	1.191337919	6.23689E-07	*NAT14*	-0.821183237	4.28016E-17
NATD1	1.191210208	1.35893E-30	*IGIP*	-0.819984594	2.03608E-09
HLA-DRB1	1.188483333	4.55549E-23	*PDIA3*	-0.819713924	1.23064E-96
PHOSPHO1	1.188391987	2.56245E-09	*DIPK2A*	-0.819292811	4.63938E-26
NRP2	1.187252704	1.40521E-11	*ASMTL*	-0.819212028	1.32757E-35
PRKAB2	1.186415612	6.1971E-62	*GALNT12*	-0.819153254	6.86156E-40
LINC02604	1.184819962	1.23737E-09	*ZW10*	-0.818486644	9.97917E-28
SLC27A2	1.184756697	8.70047E-70	*CAMK4*	-0.81696075	8.49452E-14
BTG1	1.181659199	7.9547E-101	*NUF2*	-0.815920535	8.78597E-21
CYP4F12	1.180280896	9.25832E-10	*TNIK*	-0.813364277	6.2747E-41
FLYWCH1	1.18026803	6.14319E-63	*SRSF10*	-0.81323672	1.15875E-44
AL118506.1	1.177378983	5.56493E-11	*HOXD8*	-0.813075778	7.25313E-07
PIGCP1	1.176647752	5.70849E-06	*CREB3L4*	-0.812657471	1.26508E-09
S100A3	1.175702726	2.0376E-12	*OBI1*	-0.81240518	1.37573E-23
POLD4	1.17533493	2.75153E-43	*METTL15*	-0.812395675	1.15241E-26
PLEKHG1	1.174834405	1.86524E-40	*CDCA4*	-0.810599872	2.66971E-37
CCDC187	1.174708552	4.85317E-11	*MFSD13A*	-0.808942249	1.60301E-18
PDLIM2	1.17461133	1.08173E-39	*MIS18A*	-0.808369074	6.69594E-27
DYSF	1.17379116	6.83914E-11	*MCMBP*	-0.807818588	3.78386E-67
GBE1	1.170666746	4.92108E-64	*GABPB2*	-0.807666497	2.71054E-14
ZNF385A	1.168088087	8.15715E-43	*STT3B*	-0.807285752	1.79822E-47
ARHGAP24	1.167191497	1.70726E-15	*ZNF680*	-0.806386708	1.66106E-12
PROCR	1.165395912	1.43694E-94	*CWF19L1*	-0.8055749	5.21983E-32
ENHO	1.165119398	1.09855E-10	*GON7*	-0.805055311	1.13985E-09
LTB4R	1.164986533	1.15635E-16	*ZNF827*	-0.805008669	3.15665E-26
CLCF1	1.164758419	1.46779E-17	*PAGR1*	-0.804974359	9.74297E-28
MAGEA2B	1.164698822	4.2189E-06	*TUBGCP4*	-0.804926674	1.02627E-34
HOXC10	1.161726827	6.79865E-17	*RPA2*	-0.804808742	5.21369E-39
AC016074.2	1.161485728	7.5667E-13	*PPIH*	-0.801580697	5.84853E-28
FAM98C	1.161020391	9.6456E-43	*HAUS4*	-0.801215668	1.85096E-31
CRPPA	1.159498536	1.34166E-05	*AGO4*	-0.80092868	7.42678E-13
RIMBP3B	1.158677042	1.32981E-05	*HSPA2*	-0.80076257	9.60705E-05
CDH3	1.158400935	1.95E-171	*DIMT1*	-0.800420707	5.69818E-30
PIP5KL1	1.158111273	1.3602E-22	*RNF141*	-0.800347499	2.23668E-30
DNASE1	1.157806605	1.78212E-23	*TCERG1*	-0.799913532	1.67885E-39
HMOX1	1.154293361	4.67127E-31	*MPHOSPH9*	-0.799358463	2.49614E-28
CCND1	1.152456349	2.1609E-158	*CAMK2D*	-0.798865966	1.33076E-29
ULBP2	1.151876759	2.00134E-31	*AHI1*	-0.798248584	5.45846E-23
AC007128.1	1.150957297	3.63947E-05	*RNF43*	-0.797056471	4.53436E-22
TMOD3	1.150867496	3.83994E-55	*UQCC3*	-0.796107929	9.18346E-14
TGFB3	1.150711953	2.62436E-05	*DTWD2*	-0.796066222	2.62483E-10
PMAIP1	1.150336179	9.54256E-46	*TIFA*	-0.795387086	8.49822E-26
HYAL3	1.149756556	1.04156E-21	*TCTEX1D2*	-0.794445322	0.000607164
PLEC	1.149286403	2.40482E-55	*RIBC2*	-0.794271441	6.97434E-05
ROM1	1.148905931	5.33095E-05	*NEK2*	-0.79424187	7.19973E-22
COQ8A	1.147742582	1.73355E-87	*CDCA5*	-0.793223241	3.94549E-32
TNFRSF10A-AS1	1.147591189	3.82263E-07	*KNL1*	-0.791155799	3.08103E-20
KRT19	1.146446779	7.70845E-81	*WDHD1*	-0.791001019	2.02488E-29
TRIAP1	1.143600851	3.18747E-61	*MRPS6*	-0.790816769	2.25347E-21
GLIS3	1.142323436	6.7078E-07	*H2BC20P*	-0.789406508	1.18376E-08
AC019069.1	1.142005211	6.4938E-09	*ATP8B3*	-0.788648649	4.05276E-12
KIF3C	1.14142419	2.85969E-31	*GEMIN2*	-0.788621786	3.40516E-15
MLPH	1.140316879	2.39418E-60	*CENPV*	-0.788240788	5.11247E-16
TMEM175	1.1400867	1.3439E-30	*ARHGAP32*	-0.785627128	5.78665E-15
TMPPE	1.138832183	3.92322E-06	*GCSH*	-0.785514859	4.37135E-31

AXL	1.138096528	2.6461E-118	*CRYL1*	-0.785117577	7.82039E-22
SMPD1	1.137475368	2.26439E-15	*MSH2*	-0.784207172	3.00367E-44
IL4R	1.136481513	3.19059E-70	*SUV39H2*	-0.784134528	1.0949E-18
HLA-DRA	1.136448869	1.62159E-10	*SPC25*	-0.784097875	2.19553E-17
CPEB4	1.136206193	1.37023E-38	*KAT5*	-0.783821634	6.28361E-23
GTF2IP20	1.135942701	2.54274E-14	*PHF19*	-0.783429397	1.17585E-14
AC008915.3	1.135659655	2.09977E-05	*HMGCS1*	-0.782934025	7.27386E-30
SCARF1	1.135571581	4.761E-09	*ICK*	-0.782754424	6.34292E-24
COL27A1	1.134666189	3.75709E-38	*CA8*	-0.782716762	0.000102601
BTBD10	1.134290932	1.12182E-64	*ITPRID2*	-0.781916351	8.9984E-41
CECR7	1.133856019	1.14005E-15	*NAGA*	-0.781879312	2.79692E-24
COL1A1	1.132944856	6.75856E-40	*SLBP*	-0.781779322	3.22121E-57
ASB16-AS1	1.132020373	4.31124E-20	*GRIN2B*	-0.781352061	2.35695E-15
KIFC2	1.131974732	1.78998E-54	*PPP3CA*	-0.780742084	1.31799E-52
MIR31HG	1.130206516	6.70328E-05	*ST6GALNAC6*	-0.780221617	1.44386E-11
INHBB	1.127395554	2.33865E-05	*TBC1D15*	-0.780200367	3.56533E-18
SFXN3	1.126452214	1.22528E-90	*UHRF1BP1L*	-0.779961292	5.49335E-21
TNXB	1.126423897	1.95424E-28	*NUDT21*	-0.779836919	1.08607E-49
LINC00638	1.12596447	5.89092E-06	*CAMKK1*	-0.779341754	2.41425E-15
ZNF365	1.125177011	6.67541E-13	*OSGEPL1*	-0.779153013	4.09631E-13
PRDM16-DT	1.12243377	6.69321E-05	*SLC19A2*	-0.778558096	1.12269E-14
WNT7B	1.121826687	8.84427E-36	*RFX2*	-0.778315815	3.23769E-11
KRT18	1.121658392	1.2207E-167	*GBP2*	-0.778201045	0.000545426
MOK	1.121511112	6.40821E-24	*SLC30A9*	-0.777618429	2.94856E-34
PVT1	1.120818182	6.40075E-53	*BBS10*	-0.777508979	1.08499E-18
TMEM255B	1.120267532	1.11081E-15	*C17orf49*	-0.776618335	8.60647E-10
LRRC37A3	1.119942148	4.53068E-11	*AK6*	-0.776389409	3.53602E-11
PDZD7	1.119363538	5.72319E-26	*STAG1*	-0.776280551	5.23687E-28
DEPP1	1.118213984	7.9626E-19	*DSN1*	-0.775984953	8.02663E-29
RASSF4	1.117351797	1.99847E-09	*SAP30*	-0.775916022	2.94541E-09
IL6R	1.117338671	1.10479E-22	*KIF20A*	-0.775532872	9.87083E-19
BMP1	1.115863135	7.43452E-85	*NHEJ1*	-0.775506689	6.45075E-15
XPC	1.115184588	3.2381E-114	*CDPF1*	-0.775234325	1.58011E-09
SLC25A25-AS1	1.115174224	4.7536E-23	*KLF6*	-0.774543737	1.14959E-32
CRIP1	1.114627539	5.33479E-06	*MEIS2*	-0.774439831	5.41423E-14
AP003486.1	1.112940667	5.49025E-06	*CANX*	-0.774370748	2.45804E-52
WNK4	1.112534792	5.85457E-13	*NPM1*	-0.773977182	4.57498E-45
FBXO22	1.111816616	1.58791E-87	*AL391988.1*	-0.773583093	0.025728464
SLC6A9	1.111126693	3.49399E-14	*MTFP1*	-0.773055023	8.16623E-19
AC079922.2	1.109267788	7.69659E-06	*LEMD3*	-0.771294488	3.73746E-20
CLEC16A	1.108779317	9.73394E-48	*XBP1*	-0.771258175	1.98513E-26
FAM83H	1.108583805	7.71452E-53	*GNAZ*	-0.771218549	1.34376E-11
AC120114.3	1.107992733	0.000230979	*ZNRF2*	-0.77006453	6.65766E-14
LAMA5	1.106148997	3.9585E-60	*TRIQK*	-0.768171891	3.18178E-21
CEP170B	1.105088912	1.71481E-58	*ERLIN1*	-0.767988894	2.41308E-42
MGRN1	1.104679605	3.40856E-41	*ZNF22*	-0.767528955	6.09456E-15
AC073957.3	1.104104181	1.00275E-15	*RSRC1*	-0.766952942	9.71529E-22
ZNF767P	1.103171249	3.42711E-16	*GPSM2*	-0.766698384	3.3674E-19
IL11	1.103139953	1.49695E-07	*MRPL48*	-0.766522549	1.02123E-20
LATS2	1.102613454	4.32221E-45	*CDCA2*	-0.766371493	2.14654E-17
ATXN3	1.102006212	8.97752E-39	*BRCA1*	-0.766329539	3.45802E-31
H2BC5	1.101804606	1.10037E-12	*UBA2*	-0.766148108	3.43217E-43
AC068946.2	1.100848058	1.89682E-07	*MINAR1*	-0.766055397	0.007948713
RBPMS2	1.099544178	1.5748E-33	*HLCS*	-0.765993648	4.34166E-19
NTN1	1.098284235	1.71466E-24	*TUBB*	-0.765934037	7.13632E-28
F8	1.098234801	6.78689E-17	*AC107027.3*	-0.765205527	0.000157419
JUP	1.096897127	4.66506E-78	*ZWILCH*	-0.765145603	2.05455E-18

PNPO	1.096134474	6.4007E-113	*CTDSPL2*	-0.764951897	7.32054E-24
ALDH1A3	1.095788582	3.5117E-109	*ANKRD22*	-0.763574583	1.15112E-06
ZNF747	1.094227614	3.12987E-12	*GABPB1*	-0.763369027	1.13857E-16
CLUHP3	1.092663437	3.68907E-30	*BPHL*	-0.763044232	7.59803E-15
LINC02709	1.09233746	2.1187E-05	*CHPT1*	-0.76275001	2.07871E-10
KIAA1217	1.09227107	3.20097E-39	*AC027601.6*	-0.762359033	0.001346331
MYO1E	1.090124088	3.76235E-73	*LRR1*	-0.762047227	1.97402E-18
PTGES	1.089304537	6.24171E-56	*CENPF*	-0.761536527	3.58562E-19
GOLGA8B	1.089085215	1.75778E-37	*ALG14*	-0.761403912	7.10152E-06
RAET1E	1.088574009	2.47725E-11	*PHF13*	-0.761347913	2.08084E-35
BRICD5	1.088370468	4.85175E-10	*CSE1L*	-0.76082394	2.42988E-39
NOCT	1.088340499	1.84598E-34	*CDKN3*	-0.760595476	6.71976E-19
AC012676.1	1.087076997	3.58131E-06	*PTBP2*	-0.760501998	5.77535E-10
KIFC3	1.086480611	2.28684E-63	*CORIN*	-0.760428335	0.001738327
NCR3LG1	1.085972655	1.4448E-07	*SMCHD1*	-0.760411868	5.60922E-27
RALGDS	1.085696964	3.2404E-63	*PROS1*	-0.759315587	9.7638E-16
SMPD3	1.084864581	3.22489E-10	*ATG4C*	-0.759129796	1.20287E-09
ERVK13-1	1.083757504	7.14518E-14	*ZSWIM5*	-0.758677424	0.018004503
WWC1	1.083339971	5.7024E-127	*NEK3*	-0.758621073	2.83473E-11
TRIM73	1.082580794	1.14487E-05	*HYLS1*	-0.758465222	4.29811E-14
MYOM2	1.082174973	2.55564E-08	*TBC1D30*	-0.758172615	5.54043E-22
MIR4435-2HG	1.081678937	3.31654E-40	*GAL*	-0.758092178	1.97735E-15
AC027601.2	1.081483615	0.000118622	*PPIB*	-0.757829383	9.01147E-54
IER5	1.081113115	4.76561E-78	*TBCD*	-0.75676635	7.62341E-44
ELL3	1.079821563	1.19293E-32	*FAM111B*	-0.756210708	3.98821E-09
AC137932.3	1.079119694	0.000261265	*SORD*	-0.755898554	1.30111E-61
SHC4	1.078929562	0.00024169	*PAQR3*	-0.755556631	9.59871E-23
ISCU	1.078682183	4.98797E-83	*SGK3*	-0.755212154	1.5068E-21
TNNT2	1.078167553	8.82434E-06	*PBX1*	-0.754277906	1.71399E-06
AC008966.1	1.077840336	4.81323E-17	*AC004803.1*	-0.753878818	0.007306116
AC253572.1	1.077376039	0.00098216	*CPS1*	-0.753566831	8.84031E-29
NOTCH2NLC	1.076787949	3.66044E-10	*ANO5*	-0.753031476	1.61292E-05
TAF3	1.075936797	3.90723E-53	*CPQ*	-0.752897124	0.006419609
EPHB3	1.075724633	7.31923E-24	*GGPS1*	-0.752267856	1.76426E-22
B3GALT5-AS1	1.075670898	1.31059E-05	*TBC1D16*	-0.751988039	3.22884E-25
PSPH	1.075632995	6.50005E-61	*CEP78*	-0.751735664	5.06638E-27
APOBEC3C	1.075180151	2.2164E-107	*ZFP36*	-0.75127764	2.08773E-21
CST6	1.074857643	1.62678E-22	*NT5C*	-0.750944884	3.6526E-21
CAPN1	1.074843656	9.14743E-82	*CENPI*	-0.750596211	2.10803E-12
AC008267.5	1.074199118	1.3783E-05	*TUBGCP3*	-0.750509659	5.63078E-33
C19orf33	1.07274215	9.09349E-08	*LINC01355*	-0.750312506	0.00990019
TMEM189-UBE2V1	1.072627633	2.131E-09	*BZW1*	-0.750253477	2.31797E-37
PLEKHN1	1.071119829	1.14685E-25	*TWSG1*	-0.749270473	4.81702E-16
RHBDL1	1.071059883	2.69935E-13	*RNF145*	-0.748572451	1.12357E-40
LINC00205	1.070934335	1.54286E-41	*NXT2*	-0.748512578	1.31783E-13
AC092299.1	1.070081638	3.29844E-08	*MACROD1*	-0.748381524	2.61789E-13
DSC3	1.069152188	6.52051E-36	*SMC2*	-0.748286445	4.56103E-18
KIF9-AS1	1.068450908	3.95731E-06	*OSTC*	-0.748100396	1.1035E-26
TFEB	1.068423828	2.60938E-14	*PGM3*	-0.746912012	3.44078E-28
AC009090.6	1.067560264	4.4304E-08	*ACOX3*	-0.746716247	7.33144E-14
S100A10	1.065830919	2.17726E-49	*SCG2*	-0.746408515	8.80166E-16
GPC1	1.06538855	1.86205E-50	*ITGAE*	-0.746406502	1.42596E-12
ZNF561	1.064157108	1.53748E-44	*MIS12*	-0.746368954	1.02099E-19
AL022069.3	1.063515194	0.000113767	*RFXAP*	-0.746049311	8.80767E-06
AZGP1	1.061781623	0.00011821	*ADAM22*	-0.745817195	4.17795E-14
SRA1	1.059376667	6.57817E-57	*SPACA6*	-0.744964288	0.000293023
SMPDL3B	1.058755536	9.28219E-27	*GRB14*	-0.74460642	1.078E-05

ELK3	1.058622831	4.56636E-80	*ANXA6*	-0.744301241	2.34843E-39
AC006453.3	1.058395883	2.65816E-15	*TUBGCP5*	-0.744117175	2.80574E-20
TCP11L1	1.056425216	1.38558E-26	*MAPK4*	-0.744104394	0.01911864
DAPK1	1.05630354	2.14412E-16	*KCNC3*	-0.744085475	2.81515E-06
BIRC3	1.056260555	5.42177E-25	*PRKD3*	-0.743978913	7.51894E-20
TMCC3	1.055176605	1.32E-40	*EXOSC9*	-0.743898595	8.30686E-18
MSX2	1.05462043	3.94012E-26	*SAMD11*	-0.743191986	0.005304581
AC026748.3	1.054522781	3.60048E-14	*HAUS1*	-0.742820695	5.26416E-22
PCCA-DT	1.052604855	2.71411E-11	*NARS2*	-0.742625815	2.51545E-26
ITGA3	1.0488729	1.48137E-96	*AC024940.2*	-0.742429339	0.00091264
MST1	1.047879697	2.51437E-15	*MTSS1*	-0.742146604	0.00029348
ITGA2B	1.047655548	1.30917E-10	*ECPAS*	-0.741797582	2.15914E-31
AL669830.2	1.047499341	0.005764699	*LINC01089*	-0.741716866	5.60648E-05
NT5E	1.047184525	7.78653E-56	*MED16*	-0.741713006	3.43941E-22
LTB4R2	1.046234621	1.49975E-08	*B3GNT2*	-0.741704908	6.83203E-26
MAN2B1	1.045397794	3.8119E-44	*NCAPH*	-0.740631851	1.48441E-26
LPXN	1.045386865	2.1325E-06	*RMND1*	-0.73992851	4.80841E-19
SLX1A-SULT1A3	1.045242091	7.86474E-47	*DLGAP5*	-0.739644561	5.45726E-16
NMB	1.044246175	3.02389E-11	*SEC24D*	-0.739549966	7.54915E-23
MERTK	1.042318244	1.43012E-20	*CENPQ*	-0.739153257	1.24431E-12
TRAK1	1.041984471	6.86978E-87	*MLF1*	-0.738038673	6.55141E-21
TRIM35	1.040255431	4.53758E-29	*MARCKSL1*	-0.737956909	4.57256E-27
FAM189A2	1.039129985	1.48363E-06	*VGF*	-0.737805784	4.50281E-15
CLMN	1.039118708	4.56047E-66	*HPS3*	-0.737467492	2.5602E-14
SLC1A4	1.038803674	3.98332E-17	*GABRQ*	-0.737071251	2.81129E-09
GARS-DT	1.038225711	2.65817E-19	*GSTZ1*	-0.736492907	6.99208E-14
FBXO32	1.03820149	2.66362E-10	*SAYSD1*	-0.735735588	7.07312E-08
GSTT2B	1.038027427	1.21511E-33	*HNRNPL*	-0.734842222	1.55872E-59
CLIP4	1.037611819	1.16348E-42	*AC121761.1*	-0.734666202	0.042544247
HOXB4	1.036667864	2.76457E-36	*OBSL1*	-0.733914573	3.53541E-12
FAM83F	1.036333455	3.3132E-57	*NREP*	-0.733658928	1.16229E-05
GOLGA6L4	1.035444734	2.67342E-07	*FGF19*	-0.733311362	2.75042E-25
CRYZL2P-SEC16B	1.035415403	5.19709E-05	*HNRNPA2B1*	-0.732270483	1.74756E-29
AP001062.1	1.034810381	4.47905E-05	*DENND6A*	-0.732037185	1.60432E-19
CD109	1.034514775	3.99082E-56	*ACTG1*	-0.731909476	9.08012E-39
TOM1L2	1.034281071	7.78663E-52	*NQO1*	-0.731140295	1.60545E-43
AC108134.1	1.033211959	6.36578E-06	*ZDHHC20*	-0.730816735	1.11262E-34
CHST11	1.033042272	1.32126E-34	*C2orf69*	-0.730796066	5.1023E-19
BAG3	1.032794347	1.0174E-67	*KDELR3*	-0.730674884	2.62855E-27
CASKIN1	1.031488434	1.26259E-16	*SEMA3A*	-0.73022646	3.07904E-17
BX842570.1	1.030621339	2.68111E-07	*ZFC3H1*	-0.729867984	2.54309E-21
CGN	1.029664417	3.38742E-58	*AL118516.1*	-0.729691347	0.005322115
S100A6	1.028664736	9.34535E-18	*AC132872.1*	-0.729640716	0.014375103
DOK3	1.027952083	9.6553E-06	*GMNN*	-0.729081797	4.72385E-23
SNHG26	1.027885764	6.81772E-11	*DEGS1*	-0.729056841	1.83404E-24
AC129492.1	1.027178541	1.0912E-13	*DNAJC6*	-0.727880983	7.48439E-16
LINC02454	1.026327983	0.000152937	*C9orf40*	-0.727872109	2.66635E-14
FAM229A	1.026219598	1.58025E-11	*CDKL3*	-0.727313636	0.030825086
APBB2	1.025176784	1.01714E-68	*EXOSC8*	-0.726831567	1.85844E-23
MYO15A	1.02509094	3.98013E-07	*JAM3*	-0.726790341	0.019770646
LMNA	1.024586849	1.09293E-96	*MKI67*	-0.726762412	3.3772E-18
CLU	1.023925707	1.3954E-80	*MTERF3*	-0.726681593	1.74001E-18
AC005336.1	1.023570305	6.20186E-11	*ZNF273*	-0.726509648	3.00922E-08
FAM50A	1.023252504	6.34515E-66	*BCL3*	-0.726429511	1.49106E-13
AC005329.1	1.022201494	1.77348E-10	*GNL3*	-0.725539749	1.06352E-41
SDC1	1.021498076	1.89883E-48	*UHRF1*	-0.72530758	2.11199E-25
KANK3	1.02140935	2.03681E-28	*PDE4B*	-0.725222105	1.27865E-25

ZDHHC9	1.02139118	6.60825E-48	*OSBPL8*	-0.724925009	2.09867E-23
PADI3	1.020981138	4.75133E-15	*TMX3*	-0.724880376	2.85447E-15
PARD6G	1.020411827	4.16176E-48	*PSMG1*	-0.723076182	1.16914E-18
CLTB	1.019295445	1.37294E-39	*ECSIT*	-0.722786882	2.19429E-14
AL732372.3	1.01791779	0.005101796	*PAICS*	-0.722274753	2.05298E-52
PDE2A	1.017557549	6.09759E-23	*SMIM8*	-0.721700544	1.33679E-06
PRR34-AS1	1.017304343	1.42676E-08	*PHLPP2*	-0.72165188	3.65177E-16
OPHN1	1.017048381	1.09075E-21	*COMMD10*	-0.72119507	2.09286E-14
HYI	1.016889907	1.62151E-27	*MIR924HG*	-0.720872977	0.002860711
TTC9	1.016401515	1.23864E-19	*STK39*	-0.720639879	2.03739E-56
ABCB6	1.015295002	5.06985E-46	*PLOD2*	-0.720140125	1.70483E-23
GSTT2	1.015062534	1.41566E-05	*PCF11*	-0.719865614	2.22205E-28
HOXB6	1.014652566	4.92046E-63	*MYO5C*	-0.718679215	6.57243E-22
PRSS22	1.014628946	8.77642E-14	*EGFR*	-0.717912784	4.11533E-29
LINC01719	1.014609018	4.50931E-13	*TFAM*	-0.715928022	8.5885E-25
PLIN5	1.014360841	9.30602E-10	*ZNF581*	-0.715450114	1.05862E-14
SLC44A3-AS1	1.013647424	4.15116E-07	*FANCA*	-0.715329124	2.90675E-24
DMPK	1.01353475	5.0477E-42	*PRXL2C*	-0.714767388	2.96648E-14
TAB3	1.013274923	2.6302E-52	*GCA*	-0.714099461	2.58565E-12
SH3BP5-AS1	1.013170524	1.20414E-07	*FUT4*	-0.713564598	2.10253E-08
MYO6	1.011596416	9.72115E-47	*ETFDH*	-0.713362291	4.6936E-13
PSAT1	1.010748455	5.3693E-65	*EIF3E*	-0.713345453	4.12109E-33
CYTOR	1.01026725	1.54336E-14	*SATB2*	-0.712887424	1.3077E-13
LRP10	1.009381155	2.6302E-52	*POC1B-GALNT4*	-0.712246565	0.035461184
STK36	1.008465289	1.22778E-42	*SLC29A2*	-0.711667924	3.29741E-20
NPM2	1.007696695	1.24257E-11	*TPRKB*	-0.711648318	3.75037E-12
CMBL	1.006323504	1.6787E-78	*TRMT10A*	-0.711487114	4.36942E-07
SLC39A4	1.006146087	2.02488E-29	*MRPL24*	-0.711333599	8.97324E-19
ABCC10	1.005907272	1.98345E-52	*RPA3*	-0.711203165	7.49274E-18
DUSP11	1.005550186	9.09213E-40	*C2CD5*	-0.710614941	3.24454E-18
AC099489.1	1.002089033	2.38223E-11	*H2AZ2*	-0.710337033	1.28227E-34
TTC7A	1.00178282	8.81028E-59	*BOLA1*	-0.710246456	4.98186E-10
CUEDC1	0.999347398	1.03921E-45	*IQCH*	-0.709871278	8.31511E-06
FBXW7	0.999057063	3.97518E-33	*PLA2G4A*	-0.709766793	1.04615E-11
ERV3-1	0.998964815	1.01295E-09	*LOXL2*	-0.70976674	2.95541E-11
SIDT2	0.997376149	1.03707E-43	*MANEAL*	-0.709736123	1.34404E-20
GRAMD2A	0.996812029	3.15937E-28	*RAP2A*	-0.709359919	1.07876E-18
FUCA1	0.996584829	2.83108E-65	*FUT11*	-0.709276438	5.37437E-15
LIMA1	0.995751579	8.33546E-72	*SPIN4*	-0.708993765	1.12452E-17
PRR5-ARHGAP8	0.995013831	5.66315E-07	*RAB23*	-0.708217214	1.44335E-16
CFLAR	0.994682662	5.76409E-45	*CHAF1B*	-0.708095287	6.43611E-26
ANKRD29	0.994655017	6.67267E-16	*PHF20*	-0.707717739	1.4654E-28
GASK1B	0.994214072	9.76905E-10	*ELOVL5*	-0.707245531	1.39469E-27
FOSB	0.991230135	1.71107E-09	*TIGD3*	-0.707029866	0.004467645
MAPK11	0.990885356	2.00188E-38	*SUZ12*	-0.706878857	5.31031E-27
PLAU	0.99027748	6.8282E-110	*DZIP1L*	-0.706755661	4.65819E-05
PML	0.989188224	1.19458E-45	*P2RY1*	-0.706593172	0.000165084
BCL2L1	0.987137541	2.34532E-84	*C19orf48*	-0.706086369	9.25227E-23
BAK1	0.986485005	4.71043E-32	*LPAR3*	-0.705682757	4.49458E-30
WDR81	0.985720335	1.83931E-28	*DYRK2*	-0.705592006	5.82062E-21
PRRG2	0.985465182	5.22835E-11	*RET*	-0.705566233	2.71602E-09
LINC01588	0.98543413	6.85957E-07	*PMF1-BGLAP*	-0.704917326	0.010906955
AC022107.1	0.98252	1.0071E-06	*PDIA5*	-0.704909359	2.41332E-31
DENND3	0.98250557	1.35523E-33	*HNRNPF*	-0.704411902	8.36558E-77
C2CD2L	0.981797459	2.2818E-33	*BRI3BP*	-0.703836728	5.79441E-51
WDR78	0.981362066	2.41648E-06	*SHPK*	-0.703372774	2.3886E-20
ORAI3	0.980211938	3.66932E-20	*KTN1*	-0.703304122	7.20853E-28

DNAJB2	0.979853171	8.48577E-39	*SNRNP40*	-0.70322702	8.22882E-27
EGR2	0.979685586	2.29719E-06	*GATA2*	-0.70308163	6.44425E-13
MON2	0.979028401	1.38927E-36	*DONSON*	-0.702991324	1.58338E-16
CYP2S1	0.978441353	1.35037E-34	*HACD1*	-0.702966924	1.32719E-08
RCBTB2	0.978303595	4.14985E-06	*SETMAR*	-0.702160809	1.84766E-07
RIMBP3	0.977430946	1.16056E-06	*ESCO2*	-0.702126886	1.13273E-17
AC027020.2	0.977244201	0.000466899	*LRRC8B*	-0.701600625	6.42018E-12
INAFM2	0.977170821	1.77587E-26	*B3GNT5*	-0.701505984	1.8114E-17
TLL2	0.976824028	2.62623E-07	*AC125807.2*	-0.701223932	1.08492E-06
AGAP3	0.976623932	1.2378E-44	*NAMPT*	-0.70120787	3.96886E-21
ZNF468	0.975772441	7.60064E-20	*ARMC10*	-0.701187629	7.5632E-30
BATF2	0.974150345	3.46802E-07	*ATP23*	-0.701115208	8.08453E-07
NOTCH2NLA	0.973561926	1.68407E-12	*HSPH1*	-0.700494985	6.49438E-35
HSH2D	0.973101334	1.51761E-34	*DENND5A*	-0.700379436	3.65937E-28
AL590560.3	0.972680947	2.54759E-08	*CHD1*	-0.699901307	1.13928E-33
RAB15	0.971813919	1.29893E-16	*GKAP1*	-0.699355792	6.42273E-06
ARRDC1	0.971644483	1.61602E-42	*TCTN2*	-0.699343387	6.39599E-16
NINJ2-AS1	0.971556583	7.98656E-06	*MGAT2*	-0.699035263	8.96204E-25
SH2D3A	0.970625705	2.49824E-45	*POT1*	-0.698455968	4.75226E-15
ACSL6	0.970523174	6.54946E-08	*CPOX*	-0.697989111	5.30833E-39
SIN3B	0.969326363	4.17675E-47	*HNRNPR*	-0.697696049	6.66644E-54
ASS1	0.969158527	1.2788E-59	*GFPT1*	-0.69763006	6.48671E-24
USP35	0.96870562	2.23212E-15	*SNAI1*	-0.696638721	7.95481E-07
LINC00857	0.967763072	2.07579E-24	*LETMD1*	-0.696598265	8.69979E-20
SGK1	0.967602558	1.31151E-06	*ATP1B3*	-0.696532667	1.22753E-39
STARD5	0.967582432	1.91128E-08	*KIAA1958*	-0.696478974	1.43688E-10
AC004967.2	0.965927443	6.0412E-06	*GLG1*	-0.696223396	7.36394E-37
TNFRSF10A	0.965515558	6.22463E-70	*ABHD14B*	-0.69514155	3.62334E-28
ZNF701	0.965473418	3.62547E-06	*BDNF*	-0.694726869	7.18279E-28
ADAMTS17	0.963724157	1.25664E-10	*NUCB2*	-0.694039166	8.38043E-23
AC016026.1	0.963572066	3.90537E-08	*KIAA0895*	-0.693556806	4.54399E-09
RBKS	0.962927788	1.23507E-08	*MYDGF*	-0.69341065	1.49955E-33
VPS37D	0.962448782	6.91936E-19	*RBMX*	-0.692961831	2.19012E-49
CCDC18-AS1	0.961983867	1.37484E-16	*ABCB7*	-0.691736854	1.32321E-10
AC104024.2	0.961494469	7.82485E-13	*ZNF100*	-0.691459253	7.71602E-05
ASCC3	0.960748645	1.29082E-47	*SYT3*	-0.690710968	6.13563E-05
PPP1R13L	0.960740177	2.96185E-45	*SLC25A40*	-0.690331771	1.44923E-13
AL157392.3	0.960686814	6.5339E-06	*PCGF6*	-0.689643013	2.9173E-17
SIRT2	0.960215364	4.06833E-29	*PGBD1*	-0.689246515	1.63775E-05
YBX3	0.9598117	9.3304E-100	*CHD7*	-0.689018144	7.36067E-18
JMJD7-PLA2G4B	0.958923023	2.12778E-19	*KIF22*	-0.688942293	6.51409E-32
SUSD1	0.95817232	1.66071E-18	*CCDC117*	-0.68864609	1.77042E-22
SPSB3	0.957637062	1.1244E-34	*MMD*	-0.687428897	3.73797E-15
CTNNBIP1	0.957003735	4.34177E-32	*SMC1A*	-0.68691875	3.40635E-34
GOLGA6L10	0.956849068	4.07796E-05	*NSMCE4A*	-0.68689666	5.73846E-23
MINDY1	0.956748536	0.001375502	*USF3*	-0.686800471	4.69074E-15
BCAR3	0.956207467	8.74578E-77	*BCOR*	-0.686686365	7.38402E-14
TRIM16	0.955726138	2.66789E-44	*MTMR2*	-0.686610902	1.23492E-22
AC104452.1	0.954778014	1.08945E-10	*TTBK2*	-0.685724608	1.90487E-08
HIC2	0.953849909	2.32111E-24	*UBE3D*	-0.685641466	6.361E-06
MOSPD1	0.953750525	5.38664E-55	*DLAT*	-0.685419429	4.83925E-36
PRKAR2A-AS1	0.953262913	0.000490803	*FAM20B*	-0.685134184	1.19235E-20
SPOCD1	0.951389097	9.71697E-07	*FAM241A*	-0.684900674	8.92938E-10
MEGF6	0.950667244	2.03345E-25	*GANAB*	-0.684552512	3.28966E-55
AC068946.1	0.950614435	8.09585E-09	*NUP54*	-0.684194714	4.4789E-29
VAMP8	0.950477977	1.19261E-53	*HSP90AB1*	-0.683343734	1.65157E-67
CROCCP3	0.949481505	2.71594E-06	*THG1L*	-0.682876915	6.52959E-23

RFTN1	0.949312829	1.56578E-09	ACADS	-0.682346801	1.0816E-12
AADACP1	0.948277	1.26339E-05	APIP	-0.682328413	6.8912E-22
GRAMD2B	0.948260367	3.22562E-17	PPP3R1	-0.68226409	1.23256E-23
CD274	0.946915182	3.0257E-06	H6PD	-0.681790479	2.06724E-16
MICALL2	0.946603225	2.49817E-33	RFC2	-0.681759024	2.63911E-23
NCMAP	0.946250619	7.16875E-05	ZNF138	-0.681751102	5.41755E-07
FOXJ1	0.946033926	0.001076215	SMC5	-0.681374857	2.81324E-19
FOSL2	0.945153801	7.12856E-38	VPS54	-0.681337666	7.86881E-17
LBX2-AS1	0.94496032	0.000229684	HNRNPD	-0.680943587	2.04991E-36
HR	0.944952096	4.68502E-28	AC091057.1	-0.680771171	1.70942E-09
TIAF1	0.944370951	6.47125E-05	SNED1	-0.680603353	0.005440969
CCDC90B	0.944245435	3.41577E-44	ATP6V0A2	-0.680450947	2.18202E-16
RIN2	0.9432294	0.001224648	SREBF1	-0.680004874	4.509E-38
ZNF211	0.942355205	1.28529E-08	STT3A	-0.679621798	3.59003E-61
AC087289.3	0.942354807	7.48155E-05	LRRC26	-0.679396365	0.000699172
AL391422.4	0.94215541	0.000118149	MAPK9	-0.678918878	9.35725E-29
ERAP2	0.941367414	7.16279E-07	KLRA1P	-0.678855538	0.001933916
NRBP2	0.940739815	4.83243E-36	GPR63	-0.678737341	2.19175E-08
CFAP74	0.940328183	5.3535E-07	MAP3K5	-0.678660107	5.95457E-18
MYH9	0.939549854	8.70067E-66	MTR	-0.67749731	2.16307E-24
AL022311.1	0.938565606	2.82675E-05	MZT1	-0.677290236	1.61021E-09
CD68	0.938402798	1.49104E-20	XYLT1	-0.677284098	1.79005E-05
GOLGA8A	0.93838878	6.03828E-31	RACGAP1	-0.677113514	6.60785E-22
CIDECP1	0.938120929	7.46203E-07	DCLRE1A	-0.677031089	6.91174E-20
ECHDC2	0.937662745	1.97468E-20	TAF12	-0.676903683	7.66952E-21
TCTA	0.937404167	8.04703E-20	LIG3	-0.676473069	7.08923E-27
MXD1	0.936376714	1.45022E-12	RNF130	-0.676461648	2.73952E-23
EFEMP2	0.935854459	5.22778E-09	ORC3	-0.676249085	1.7146E-16
NF2	0.934525961	9.27704E-79	CD58	-0.675222665	6.24281E-08
FGD6	0.933947217	1.05403E-31	TAF1A	-0.674600008	1.02816E-10
TMCC2	0.932699853	1.97244E-12	CISD2	-0.674593816	6.85451E-25
LPAR5	0.932473078	1.13477E-13	FADS1	-0.674457477	3.60402E-38
SVIL	0.931751286	9.31136E-42	MBNL3	-0.674374529	0.001031016
IP6K2	0.931367072	1.31138E-79	FAM72A	-0.674170789	1.48098E-08
SPTSSB	0.930940589	1.95974E-05	CCDC171	-0.673850819	0.000779608
PXN	0.930806505	1.08959E-79	HMGN5	-0.6738002	5.5388E-11
DNAH10	0.930407344	0.000138209	METTL9	-0.673405249	7.10183E-28
AL008721.2	0.929675931	3.04508E-05	MMS22L	-0.673281261	8.07366E-21
GAD1	0.929039229	1.49122E-20	UBE2S	-0.672005763	6.509E-18
NFATC4	0.928907909	1.23026E-13	TRIM37	-0.67132589	1.18715E-34
MIR3936HG	0.928736204	0.001158694	SLC25A10	-0.671315437	1.68002E-17
TENT5B	0.928660393	5.09335E-18	MCUB	-0.671166251	1.27225E-12
CHST14	0.928555212	1.28477E-30	STIMATE-MUSTN1	-0.670826963	0.002727431
FBXL18	0.928548652	5.61347E-36	PRMT3	-0.670586404	2.61882E-13
NR4A1	0.928423698	1.33603E-22	AL513534.3	-0.670585095	0.043523849
MELTF-AS1	0.926581676	0.001110372	LRRN2	-0.670362198	6.3792E-06
MARVELD1	0.926362169	1.33465E-50	FAM217B	-0.670325254	2.26073E-12
PIK3IP1	0.926222121	6.1068E-05	TMEM123	-0.669981955	2.25266E-25
SYTL3	0.925602812	4.81968E-46	STK26	-0.669929124	1.07213E-19
AC009113.1	0.925572706	1.50843E-05	DDHD2	-0.669804033	6.46635E-31
AC009120.2	0.925302152	0.003212456	UBE2J1	-0.669258286	9.80228E-24
ZNF441	0.925202106	1.72626E-05	CNKSR3	-0.668461124	7.70109E-12
NCKAP5L	0.924363029	1.0715E-27	RNF144A	-0.668092516	9.23707E-16
CAPN2	0.922797908	2.58E-123	SLC25A13	-0.66654363	1.11334E-10
ARHGAP27P1-BPTFP1-KPNA2P	0.922351067	1.07339E-09	SNHG8	-0.665895044	2.08146E-16
S100A11	0.922209632	3.49187E-37	ARL4A	-0.66525648	2.22415E-12
PPARD	0.921791498	2.22988E-47	DPY19L3	-0.664587052	8.04423E-12

MIEF2	0.921553564	3.78064E-24	*IFT52*	-0.66442143	4.20808E-17
UBXN11	0.92077495	9.68513E-23	*LIN52*	-0.66418261	3.68286E-10
LYG1	0.920749242	0.000720926	*ALG6*	-0.664114155	2.84129E-12
BORCS8	0.92055782	7.85707E-11	*TMED10*	-0.66382701	4.56196E-42
VEPH1	0.920429962	0.000153813	*FABP5*	-0.663671107	2.5671E-19
ZNFX1	0.919849621	5.90405E-56	*LINC01515*	-0.663452639	4.56632E-06
DTX2P1-UPK3BP1-PMS2P11	0.919515302	1.22362E-07	*SNX5*	-0.663359062	9.87159E-35
FOXN3-AS1	0.919462483	0.002340265	*HS2ST1*	-0.662464187	8.20284E-27
NPAS1	0.918853355	4.72074E-05	*CDK5R1*	-0.662449702	3.23728E-08
AC068587.4	0.918841301	3.96225E-13	*MRPL11*	-0.662439233	2.0724E-18
PERP	0.91878659	1.19099E-92	*ONECUT2*	-0.661996312	2.15573E-11
TNFRSF10D	0.918282762	6.73315E-88	*TRIM36*	-0.661466084	6.44407E-09
TUFT1	0.917286922	3.01781E-23	*GALNT6*	-0.660834598	5.68333E-12
AC022400.9	0.917014417	3.90662E-05	*EBP*	-0.660809557	1.0095E-18
SIPA1L3	0.916668175	2.7037E-22	*PPIG*	-0.660389574	1.83421E-22
PINK1	0.91659817	2.05519E-29	*ANKRD18EP*	-0.659877815	0.001046339
ZNF814	0.916492293	6.69081E-07	*EML1*	-0.65972809	6.95813E-10
ZNF654	0.916368543	3.30822E-22	*ADGRF1*	-0.658682383	6.35201E-29
MPZ	0.916140281	1.08827E-08	*CDK2*	-0.658619317	1.1521E-21
HRAS	0.915613054	1.92686E-27	*PPP2R5A*	-0.658536912	9.54625E-33
GPR153	0.915207116	4.69488E-15	*FANCB*	-0.658312	4.78332E-05
RFX5	0.914925539	2.04783E-59	*LENG9*	-0.658212385	1.95217E-06
ZNF185	0.914082815	5.99518E-46	*SOWAHC*	-0.65788628	1.90626E-13
TMC8	0.913991585	1.65535E-14	*EME1*	-0.657881232	4.45555E-10
DACT3	0.913981773	1.02769E-06	*RAD54B*	-0.657814681	1.73655E-18
OPTN	0.913865774	7.22919E-48	*PBX3*	-0.657262733	3.21548E-08
HSF4	0.913624291	1.5634E-15	*AP000295.1*	-0.656999079	0.001945723
LTK	0.913451715	3.33394E-23	*ZNF232*	-0.656918706	6.14419E-06
RHBDF1	0.913344887	5.25796E-20	*CNTRL*	-0.656843859	2.92825E-10
SNORC	0.912965435	0.000149906	*ZNF487*	-0.656816016	0.011514099
TMEM44-AS1	0.912687783	5.42054E-05	*TC2N*	-0.656811648	8.84387E-11
SPDYE3	0.912090734	5.34216E-06	*COQ3*	-0.656516304	1.05847E-08
AC093525.7	0.911711982	0.000274545	*SFR1*	-0.656407342	3.51237E-14
AL627309.6	0.910519231	0.000294807	*ELK4*	-0.656156802	2.61147E-17
LINC00265	0.910519065	1.48525E-07	*LRTOMT*	-0.656083637	8.88179E-05
RUNX1	0.910183903	1.11573E-46	*FAM13A*	-0.656052276	4.0066E-10
KREMEN2	0.910182803	2.52046E-22	*ZNF627*	-0.655839122	1.71899E-09
KCTD13	0.909706978	1.86971E-18	*INSR*	-0.655805443	7.2307E-19
EME2	0.908937037	1.70952E-33	*IL17RB*	-0.654839327	9.49471E-14
AC018638.4	0.908321967	5.11391E-05	*PARPBP*	-0.654736555	6.45317E-12
DNAH17-AS1	0.907768865	2.10786E-07	*TNPO3*	-0.654721923	3.71337E-39
SOWAHB	0.907667041	1.1773E-16	*LDHB*	-0.654701152	1.11831E-44
ZNF117	0.907655284	1.32957E-10	*WDR62*	-0.654589179	9.04395E-12
AL662795.2	0.907596877	4.33732E-07	*CCNQ*	-0.654420615	2.02559E-09
FRMD8	0.907353758	1.10156E-32	*LDHA*	-0.654053954	3.55842E-28
MSLN	0.907275654	7.95472E-34	*ANP32A*	-0.653752587	1.64004E-48
BX890604.2	0.907211833	1.04314E-42	*UNC13A*	-0.652864089	6.0103E-33
FGD3	0.907013945	3.40356E-14	*MCM8*	-0.65283798	1.90898E-16
ASIC4	0.906730631	0.00055075	*SACS*	-0.652753811	2.11055E-16
AC100810.3	0.906479139	0.003663871	*ARL5A*	-0.652477402	7.96623E-14
AC138028.4	0.905487265	6.0429E-10	*DIRAS1*	-0.652464922	1.97529E-27
SMAD3	0.905358119	9.45772E-72	*CHD1L*	-0.652290287	1.37271E-26
ZDHHC8	0.905310018	3.26527E-28	*CDC6*	-0.65219248	3.91762E-21
WDR45	0.904957083	2.31034E-28	*MARC2*	-0.652142438	1.37929E-10
MYLK	0.904933842	3.9446E-17	*MED30*	-0.652096889	4.88576E-10
FBXO2	0.904761803	3.16031E-28	*TMED1*	-0.651653346	3.0899E-11
TINCR	0.904036163	4.37303E-20	*KIF2C*	-0.651467079	1.02175E-18

SLC37A1	0.903863196	7.40521E-24	*CETN3*	-0.651420536	3.27213E-15
RAP2B	0.903858143	8.41398E-79	*TANK*	-0.651233254	6.46122E-18
CACNB4	0.903768188	5.03597E-07	*TAF4B*	-0.650989545	5.02896E-19
TMEM63C	0.902858021	9.08721E-20	*AP1S3*	-0.650973229	3.36226E-25
AL591806.4	0.902670656	0.027816143	*TTLL1*	-0.650671522	0.000331168
AL732372.2	0.902466534	0.000983183	*ZBTB41*	-0.650382146	5.77297E-18
PISD	0.902456946	1.10737E-36	*GNAL*	-0.650142806	1.03193E-09
MDFI	0.902408161	1.54319E-22	*AP006621.3*	-0.650074244	0.003613698
AC025419.1	0.902256039	2.90134E-09	*S1PR5*	-0.64997004	1.99316E-07
AL133352.1	0.902070603	9.49838E-12	*XK*	-0.649360118	2.41598E-07
ANKRD23	0.901727229	8.18875E-05	*ERO1A*	-0.649344703	3.6003E-25
ITPKC	0.900665862	1.60702E-21	*HSD11B2*	-0.648078619	1.31851E-07
AC138932.1	0.899565077	4.4482E-17	*TIPIN*	-0.647863464	9.25024E-12
UAP1L1	0.899268538	7.42146E-28	*RGMA*	-0.647516897	5.94218E-06
TRIM26	0.899256326	3.86136E-57	*RHOT1*	-0.647038037	4.14641E-23
NBPF14	0.898578063	7.62043E-34	*RSBN1L*	-0.647033333	2.38818E-21
LY6G5B	0.898063906	2.69865E-17	*SYNE2*	-0.646832369	1.17665E-11
RBM24	0.897613264	6.37596E-17	*ZIC2*	-0.646572452	1.2224E-18
MFGE8	0.897086068	2.00585E-52	*MELK*	-0.646564625	1.57248E-21
RAB17	0.896703328	1.38691E-22	*ACTL6A*	-0.646551972	1.02562E-28
AC109322.1	0.896462957	0.003779046	*CCDC77*	-0.646447888	5.98931E-09
SNX19	0.896426504	2.80697E-31	*IGSF3*	-0.646349223	1.0252E-20
MCAM	0.896203839	3.76938E-54	*MBNL1*	-0.646160498	8.74664E-23
APOLD1	0.895675694	5.11521E-05	*ITPRIPL1*	-0.645829501	3.50351E-13
BDNF-AS	0.895550914	2.22781E-05	*FAM98B*	-0.645296293	4.6834E-13
CEACAM19	0.895022384	0.000118239	*RIDA*	-0.645123452	2.37513E-10
ZNF488	0.894818819	4.2886E-16	*ZFP30*	-0.645069657	3.45628E-05
IL10RA	0.893629174	0.001502014	*PCLAF*	-0.644862149	2.59994E-27
CSNK1G1	0.893501526	7.89955E-43	*SNRPF*	-0.644760221	3.81385E-15
LINC02495	0.893491847	1.63491E-06	*FAM72D*	-0.644699294	1.19998E-06
E2F7	0.893425895	1.72929E-28	*IMPDH2*	-0.644653602	3.12997E-61
RABGGTA	0.893007764	2.61607E-21	*HEIH*	-0.644603284	0.006132984
CASC9	0.892851477	1.13601E-06	*PGM2L1*	-0.644389028	9.21579E-09
ACTN1	0.891817568	7.25471E-63	*SNRNP35*	-0.644090566	1.24504E-07
ZDHHC11B	0.890109933	3.36007E-07	*SEC11A*	-0.644042607	2.12956E-28
CLCN1	0.889898244	0.000779063	*DNAJC9*	-0.643615874	2.42976E-21
MST1L	0.889009374	1.33335E-10	*FAM135A*	-0.643304519	5.30481E-11
UPP1	0.888063438	1.47348E-13	*BRCA2*	-0.643241903	3.54962E-09
ALDH4A1	0.887274996	2.70367E-35	*PWWP2A*	-0.643187844	8.00495E-10
ARTN	0.887076094	3.49967E-11	*ANKRD12*	-0.64314724	4.42525E-10
PTCH1	0.886714612	2.43514E-17	*CKAP4*	-0.642863932	2.14404E-33
NKX1-2	0.88551567	1.35543E-19	*DTD1*	-0.642859829	1.23362E-24
CFAP157	0.885151626	0.000879495	*CDKN1C*	-0.642375537	1.23097E-07
NUDT16L1	0.885005597	2.27473E-18	*SVBP*	-0.641700318	8.51382E-06
ATG2B	0.884862935	1.71534E-12	*KIF18B*	-0.641489406	2.81054E-17
CORO1A	0.884794273	3.48662E-06	*ZNF136*	-0.640958767	0.013904845
EPB41L4B	0.884572161	6.47405E-34	*UBE2V2*	-0.640905802	4.09728E-20
LRRC37A4P	0.884349351	2.39049E-28	*CCNC*	-0.640581542	7.3383E-20
ISL2	0.883668257	2.01297E-08	*FRMD4B*	-0.640564691	0.001726191
PRKCG	0.883455336	3.5601E-10	*SKA3*	-0.64047391	4.38519E-16
SP2-AS1	0.883391071	0.000174989	*ZIC5*	-0.640408115	2.73141E-12
CTSD	0.883182486	5.49349E-33	*KLF7*	-0.640052919	2.57103E-11
RHOF	0.883039893	2.70797E-34	*PRSS12*	-0.639776384	2.29492E-23
LAMB2	0.882925133	6.83244E-47	*EFNA4*	-0.638959151	3.05939E-06
AHRR_2	0.882521615	4.31608E-06	*BMP6*	-0.638525386	0.005204764
VPS13C	0.882458714	1.98678E-36	*CSRP1*	-0.6384679	8.7958E-24
WRAP73	0.881773473	1.07548E-46	*CEP63*	-0.638311338	9.7657E-14

TMCC1-AS1	0.881434763	0.000288513	*PAM*	-0.637246074	4.44978E-29
BICDL2	0.881292542	2.24261E-18	*PAXIP1-AS1*	-0.636552608	9.46305E-06
AL133325.3	0.880829677	5.63759E-12	*ATRIP*	-0.63622822	7.06776E-13
PRKG2	0.880382714	3.36412E-06	*KHDRBS3*	-0.635963676	6.26993E-11
NBR2	0.879786989	6.04545E-07	*GNB1L*	-0.635960923	1.89872E-06
AC022210.2	0.879779561	0.005634894	*PXN-AS1*	-0.635931526	0.002106313
SPRYD4	0.879676919	2.1403E-13	*IL27RA*	-0.635922017	4.29216E-13
RPS6KL1	0.878024399	6.06412E-09	*SELENOT*	-0.635895178	2.7305E-17
SLC35D1	0.87768472	1.91736E-27	*AC107871.1*	-0.635788092	1.00753E-05
LINC00294	0.876720773	1.25732E-16	*NRF1*	-0.635691205	3.23647E-13
LINC01145	0.876190313	6.69331E-07	*AP1S2*	-0.635491052	5.4907E-10
CPEB2	0.874473747	2.88293E-05	*AAAS*	-0.635446993	3.01382E-27
TOB1	0.873869514	1.15369E-55	*ERRFI1*	-0.635127884	1.40396E-34
FBXO44	0.872147334	2.8663E-26	*MYT1*	-0.634421941	3.5256E-06
CRISPLD2	0.871724416	1.08017E-18	*SPRY2*	-0.634177643	4.86771E-07
ZFP41	0.871656384	1.7069E-12	*LPGAT1*	-0.633698748	4.6648E-22
SYNJ2	0.870807269	1.01509E-47	*SPCS3*	-0.633540844	1.1012E-20
CPLANE2	0.870721321	6.64429E-09	*AC006230.1*	-0.633418814	0.003481271
SIRT4	0.869656408	0.001466116	*HHEX*	-0.632810678	6.54191E-09
C5AR1	0.869535847	2.18328E-08	*HGH1*	-0.632646612	1.7602E-09
SLC29A3	0.868920112	7.27907E-16	*THAP12*	-0.632600469	6.66794E-15
LBHD1	0.866631963	3.65887E-12	*MCTP2*	-0.632083336	1.24008E-09
DNAJC18	0.866074867	5.10146E-13	*CNIH1*	-0.631969849	6.65527E-20
CYHR1	0.865924519	1.80906E-32	*DHX9*	-0.631659651	7.50345E-34
C11orf68	0.865001983	1.12144E-24	*CCNJ*	-0.631650511	1.93119E-10
SNX33	0.864928983	5.63247E-20	*EED*	-0.631107344	2.7624E-16
AL035530.2	0.864623604	1.20352E-06	*TTC28-AS1*	-0.631079073	9.80853E-06
ZBTB45P2	0.864194879	8.44938E-10	*DENND11*	-0.630663291	7.68271E-21
AJM1	0.86380807	9.24832E-11	*HMGN2*	-0.630325636	1.13822E-21
FBXL8	0.863380321	2.54583E-11	*C18orf54*	-0.630138032	3.68255E-08
AL928654.4	0.863279809	1.34315E-09	*SND1*	-0.630137904	8.23779E-44
SERPINB8	0.863189094	8.22384E-09	*HNRNPDL*	-0.630063702	8.07531E-21
AIFM2	0.863126772	1.17312E-36	*SPTLC2*	-0.629947871	2.40493E-21
ENC1	0.863124009	1.19592E-47	*KNDC1*	-0.629390725	0.000244534
AC012676.5	0.862620214	0.000543207	*MAP3K4*	-0.628746417	2.7215E-17
MYL9	0.862522209	3.71356E-26	*MPHOSPH10*	-0.62837546	1.1562E-18
TNNI1	0.862511452	1.21965E-05	*TWIST1*	-0.6281812	0.001373101
ATOX1	0.862081894	3.29112E-16	*STEAP1*	-0.628063734	9.82517E-09
DUSP5	0.861587075	6.5994E-104	*IER3IP1*	-0.62799734	4.88159E-17
ARL4D	0.861519125	8.3348E-19	*TSPAN3*	-0.627784957	2.68337E-33
MFSD4B	0.861400576	7.14495E-18	*LRRC46*	-0.627638041	0.038322147
LDB1	0.861392356	1.79172E-61	*NUP85*	-0.626825805	7.6055E-34
ZNF512	0.860943213	4.24225E-35	*ZBTB8B*	-0.626747562	0.036235237
PPIEL	0.860732227	3.14842E-08	*EEF1AKMT2*	-0.626558521	6.27621E-14
VPS37B	0.860169018	3.90212E-56	*RTN4R*	-0.626545483	3.21809E-08
ZBTB5	0.859670563	4.99684E-44	*ARSB*	-0.626497615	5.00525E-07
TRHDE-AS1	0.859548656	1.65671E-10	*EBPL*	-0.625809394	3.1703E-20
SIRT7	0.859466572	8.22546E-33	*C12orf60*	-0.624340803	0.000855128
ZBTB11-AS1	0.858536355	2.76471E-05	*ATP9B*	-0.624093413	1.63747E-07
GPR173	0.858344125	4.00573E-13	*NRSN2-AS1*	-0.62325927	2.01041E-05
EFNA5	0.857859323	1.20768E-05	*SLC39A8*	-0.622917226	2.37434E-16
RELL2	0.85595561	2.62302E-11	*SET*	-0.622190765	1.45884E-43
RHPN1	0.855487135	1.16708E-18	*KLF10*	-0.622112268	2.17261E-22
STARD10	0.855309239	1.36301E-20	*LYRM4*	-0.622020442	1.43971E-12
TMEM256-PLSCR3	0.855066288	0.011961812	*DCLRE1B*	-0.621480884	3.43706E-11
NPIPA1	0.854607516	5.35762E-30	*CEP41*	-0.621430381	3.51424E-11
SH2B3	0.854490493	1.61161E-29	*BCKDHA*	-0.620700354	1.59989E-15

SMAD6	0.853767847	2.11905E-14	*RAD51*	-0.620603339	5.97047E-15
ARNT2	0.853710666	1.99336E-16	*MIR4453HG*	-0.619723671	0.024704856
IRGQ	0.853573686	3.27327E-23	*P3H3*	-0.61946957	4.93849E-21
DNER	0.852879086	4.9032E-05	*FGFR4*	-0.619333545	4.29281E-17
NLRX1	0.851399987	1.36879E-24	*GLB1L2*	-0.618919981	7.06403E-17
TOP3B	0.850052085	1.4938E-28	*AC068888.1*	-0.618824539	0.000124533
GARNL3	0.849455471	1.38661E-08	*SPA17*	-0.618822173	6.20313E-07
STX1A	0.849429562	1.30739E-20	*ALDH9A1*	-0.61861736	4.2263E-21
ARG2	0.848401294	1.40159E-13	*BOD1*	-0.618579103	1.30365E-21
PTOV1-AS2	0.848314076	0.010818981	*SNRPG*	-0.618577074	0.00012774
AC022137.3	0.847564975	0.000525111	*CABLES2*	-0.618285696	9.04327E-11
NPIPB11	0.847563718	2.58744E-05	*PLEKHJ1*	-0.618071036	2.57425E-21
ULK1	0.847454583	3.33056E-29	*DYM*	-0.617390822	4.50837E-18
AHDC1	0.847342613	4.14207E-25	*METRN*	-0.617308256	1.34415E-10
AC092338.3	0.847211195	0.007983005	*FANCM*	-0.617046054	5.51766E-10
ACP7	0.846565833	2.30487E-06	*SKA1*	-0.616822896	2.13241E-08
PNPLA2	0.846012497	8.72022E-28	*GMCL1*	-0.616608396	6.29279E-19
ELF1	0.844841142	4.73715E-31	*COMMD8*	-0.61655898	3.37188E-08
DHRS7	0.844804903	1.08423E-49	*TXNDC11*	-0.616235299	6.44181E-18
TRIM32	0.843979792	6.54859E-37	*BX088651.4*	-0.615809913	0.014324934
MTG1	0.843554425	9.05379E-19	*MASTL*	-0.615348628	1.65061E-12
TCAF2	0.843219455	1.95273E-05	*CDON*	-0.615029556	9.6374E-11
CRIM1	0.842788945	2.11125E-58	*HPRT1*	-0.614920768	1.36228E-19
CSRNP1	0.842345523	1.97536E-30	*EML5*	-0.614750804	0.000368895
MGAT5B	0.841619939	3.90849E-11	*LPAR1*	-0.614713738	2.84134E-10
OSBPL3	0.841471719	9.59853E-40	*KLF11*	-0.614108386	1.90523E-07
AL096870.2	0.841103553	0.007893469	*PIGX*	-0.613776443	1.04972E-14
AC138969.3	0.838860859	2.58492E-07	*AL357075.3*	-0.613459186	0.011791829
RUSC2	0.837688427	1.90999E-23	*ANAPC15*	-0.61334511	6.67911E-10
LURAP1L	0.837185365	2.93658E-05	*FBXO45*	-0.613054459	3.87694E-15
BEST1	0.836552995	0.001856523	*LHX2*	-0.612992526	2.01154E-05
AAK1	0.836496632	4.73361E-24	*SNHG3*	-0.612564894	5.97149E-13
HERC2P2	0.835554251	1.49861E-34	*GALK2*	-0.612436414	1.57491E-06
ZNF383	0.835346888	4.71941E-10	*SFPQ*	-0.61231405	5.0646E-46
MROH6	0.834625569	2.60499E-25	*SDHD*	-0.611672611	1.79022E-27
STK10	0.834591934	2.31061E-35	*TMEM18*	-0.611260904	5.70205E-12
SH3BGRL3	0.834481606	9.91594E-26	*MIOS*	-0.611252881	1.92184E-12
SLC15A1	0.834281061	1.95277E-11	*CHN1*	-0.611224156	0.007931042
STOM	0.834280664	1.48022E-31	*SRSF6*	-0.611125593	4.19978E-30
AC018638.5	0.834269549	0.005588236	*NSRP1*	-0.610691412	1.66834E-16
ZNF337	0.83394062	2.32752E-22	*UBE3B*	-0.609793867	5.19947E-20
AC110079.1	0.833369253	4.81145E-10	*LIX1L*	-0.609609095	8.10739E-17
CCDC30	0.832982785	0.002407312	*TOE1*	-0.609573183	7.50882E-07
PLSCR3	0.832953093	3.93034E-22	*NCOA7*	-0.609541151	3.03326E-11
FTX	0.832926514	1.50953E-11	*H2AX*	-0.609521278	4.01205E-12
IFFO2	0.832709966	1.22971E-55	*GMEB1*	-0.609408106	1.96631E-11
AL049840.2	0.832673689	0.003818933	*SS18L2*	-0.608751659	2.34567E-08
PCBP4	0.832110127	2.6811E-39	*AZI2*	-0.608491161	1.53106E-11
GOLGA8N	0.83145576	0.000339704	*METTL7A*	-0.608477052	1.13342E-05
HOXA1	0.831255878	4.94895E-05	*RPARP-AS1*	-0.608365598	2.83671E-08
BOLA2B	0.830494801	0.000815813	*SPRYD3*	-0.608280276	6.8912E-22
PPP3CB-AS1	0.829870468	5.98226E-05	*CHORDC1*	-0.60803045	8.01648E-20
AC023509.1	0.829639137	1.8462E-07	*CDK2AP2*	-0.607910418	1.9521E-14
SH3TC2	0.829442705	5.31816E-30	*SERPINB9*	-0.607316162	1.079E-17
RABL2A	0.828899928	2.14638E-08	*FARP2*	-0.607033431	1.15364E-11
NTN4	0.828672908	3.93426E-15	*DIXDC1*	-0.606975845	5.74092E-08
BAIAP2	0.828667924	3.67025E-29	*LMNB2*	-0.606918655	1.95976E-25

ESRP2	0.828642348	1.22676E-38	*CYP24A1*	-0.606568073	1.67176E-30
APAF1	0.828596433	5.07281E-25	*FAM133B*	-0.606562788	8.74654E-11
AP5Z1	0.827873214	2.86929E-13	*MRGBP*	-0.606502847	1.41045E-26
AC106782.1	0.827484214	0.000718057	*INPP5A*	-0.606112441	1.5299E-08
LINC00471	0.827459708	0.006622711	*FAM149B1*	-0.605866228	4.02188E-09
FAM157A	0.827135734	6.06726E-14	*SELENOP*	-0.605723561	0.000863383
PLEKHM1	0.826364484	5.144E-28	*KIAA1841*	-0.604835504	0.001292768
B3GNT7	0.826330796	9.58623E-21	*RARS2*	-0.604775273	3.896E-14
EPS8L1	0.826147605	3.51295E-20	*UACA*	-0.604060646	1.71734E-14
NEK8	0.826128346	1.14762E-07	*CRYZ*	-0.603690938	4.61173E-14
SLC12A9	0.825977573	3.73266E-36	*SMAD1*	-0.603536576	9.56925E-15
LINC00513	0.825239816	0.003258062	*RFC3*	-0.603511566	1.3698E-10
TRIM62	0.82520956	1.00156E-12	*TMTC4*	-0.603495044	2.02627E-10
RHOC	0.825175148	1.31084E-39	*PPIL3*	-0.603447474	3.50142E-13
AL627309.5	0.824731774	2.80293E-06	*AURKB*	-0.602625712	1.17236E-12
ZCCHC12	0.823410358	0.000110976	*ACVR2B*	-0.602582478	2.0031E-10
MAP3K14-AS1	0.823177248	1.75417E-06	*ADAM10*	-0.60257116	6.16134E-21
MLXIPL	0.823037362	3.40734E-26	*SEC61G*	-0.602119912	2.31352E-09
PNMA2	0.822486461	0.010253257	*FBXL3*	-0.601976484	5.40962E-16
SLC43A2	0.82238355	9.66759E-16	*KPNB1*	-0.601834932	2.45256E-40
ANXA4	0.822374636	1.27235E-40	*AC012467.2*	-0.60173936	0.001109981
CITED2	0.821959218	1.45435E-24	*TMA16*	-0.601732031	2.75253E-15
RAB3A	0.821577546	2.58152E-18	*ELOA-AS1*	-0.601703827	0.013493142
SOGA1	0.821222938	6.23669E-22	*GTSE1*	-0.601258842	1.40792E-11
FAM214B	0.821162466	4.37315E-19	*TMED7*	-0.601181794	6.75339E-21
GTF2IP13	0.821131451	6.7002E-08	*DHRS2*	-0.601178761	0.000745647
IFT27	0.820460176	8.75441E-27	*CCDC85B*	-0.601159431	3.67285E-14
SLC17A9	0.820396442	2.40422E-19	*TMX1*	-0.601118595	3.5677E-16
ACAP3	0.819281975	9.56513E-27	*SNHG30*	-0.601110286	0.003913794
STX18-AS1	0.818496549	1.26314E-08	*PHTF2*	-0.601065654	2.6101E-13
C6orf99	0.818407725	0.003279706	*CPSF6*	-0.600749462	6.40068E-38
ZDHHC8P1	0.818177715	0.000121228	*NR4A2*	-0.600581968	4.92473E-07
DCUN1D3	0.818138272	1.74979E-41	*DNAAF5*	-0.600294732	2.8783E-21
STX16	0.818020953	5.63835E-30	*ULK2*	-0.599748605	1.13852E-12
UBTD1	0.817529803	4.19865E-14	*BEND6*	-0.599541417	0.001150524
CES3	0.816577375	1.00254E-12	*BMPR1A*	-0.599472909	2.38258E-15
METRNL	0.81636153	5.91577E-27	*SLC41A2*	-0.599463629	7.21987E-07
ZGLP1	0.815667331	0.000632283	*ZNF292*	-0.599156021	3.75448E-17
DES	0.815549317	1.14158E-07	*TDRD3*	-0.599148856	1.68493E-10
DAP	0.81537974	3.151E-47	*G6PC3*	-0.599045932	6.28095E-12
COQ6	0.81504801	1.78215E-20	*NUP37*	-0.599039582	2.61736E-14
AC004706.3	0.814353876	0.000173841	*ATP5MC2*	-0.598747568	3.69376E-11
TNK2	0.81412653	3.3946E-30	*MRPL54*	-0.598540865	3.51054E-09
ZNF513	0.814078156	6.05382E-15	*SOCS6*	-0.598412028	1.38865E-13
AL132800.1	0.813571699	0.000569881	*ZNF438*	-0.598398474	0.000867283
EHD1	0.813315226	5.88672E-33	*CAT*	-0.597787743	4.24087E-23
BRMS1	0.813102379	3.87466E-30	*NPAT*	-0.597717324	6.08503E-13
ZNF213-AS1	0.812855859	1.52916E-07	*ERVMER34-1*	-0.597376668	6.57034E-13
NXN	0.81266629	1.21606E-30	*ZBTB8A*	-0.59732825	3.26415E-08
BHLHA15	0.812372767	0.004537002	*NUP188*	-0.597198637	1.89401E-26
FP236383.3	0.812327769	1.5208E-05	*FUBP1*	-0.597059175	5.35729E-26
SLC27A4	0.81225038	7.51889E-27	*TMEM39B*	-0.596447061	2.30896E-09
S100A1	0.81139613	0.000546408	*MPP6*	-0.595732211	1.19751E-10
SOX9	0.811061455	3.1458E-56	*SAMD13*	-0.594914139	0.000443358
HERC2P9	0.810213997	2.46023E-15	*TMEM258*	-0.594801092	0.000373101
MYL5	0.809500071	8.02754E-09	*CCNF*	-0.59455688	6.62882E-17
TJAP1	0.809459585	2.83228E-30	*ANLN*	-0.594142558	9.09262E-14

DGAT1	0.809002406	3.59344E-25
IQCJ-SCHIP1	0.808551088	1.44427E-14
ARC	0.807412828	2.78417E-15
TMEM86A	0.807125592	0.000601678
MVP	0.807076502	2.3014E-55
B3GALT5	0.806615092	6.20017E-07
KDM4A	0.806353189	1.54886E-45
NEIL1	0.806200475	1.20577E-25
RUNDC3A-AS1	0.806181084	1.69566E-06
ATP6V0E2	0.806118598	1.05826E-20
NDST1	0.805926757	2.24306E-38
SYBU	0.805639188	3.08753E-16
ZMIZ2	0.805370283	5.90149E-38
BCL11A	0.8047242	0.008866205
CABP1	0.804225936	0.000793051
MXD4	0.803883773	1.01144E-19
ATXN7L1	0.803584563	1.05651E-13
PTK2B	0.803547951	1.37946E-19
AC012640.4	0.803396867	0.00150813
AMOTL1	0.803251229	2.24773E-48
C6orf226	0.802497645	0.000824397
CMIP	0.8020713	1.80079E-43
PICK1	0.801791154	2.08907E-20
NAV2	0.800419489	7.39788E-09
MAPK13	0.800319263	6.0152E-40
PTPN14	0.800143125	4.63842E-36
C2orf16	0.799180473	0.001280175
TMEM158	0.799108011	8.55878E-10
CPLANE1	0.798981192	3.28171E-12
DOCK5	0.798774427	5.04674E-35
OVOL1	0.798488248	5.65707E-20
STX6	0.798441881	5.48865E-53
TTLL3	0.798200027	3.90077E-25
PLEKHA6	0.79802809	3.62665E-13
SCNN1A	0.797549447	2.30384E-52
CCDC69	0.796700715	5.63416E-11
PDLIM1	0.796672144	9.61904E-50
WDR25	0.796555697	3.7441E-07
SLC23A3	0.795667711	8.35989E-05
UBASH3B	0.795271776	3.83965E-68
TMEM59L	0.794319162	4.421E-17
ZBTB4	0.79428503	3.49478E-48
UBAP1L	0.793739956	4.89441E-05
INO80B	0.792577118	4.74351E-14
H2AC6	0.792515789	1.11168E-07
MTMR7	0.791767675	0.01504143
KCNIP2	0.791755225	3.79821E-06
ERFE	0.791740723	2.09411E-11
GRIK5	0.791074774	0.001269006
FTH1	0.790056903	1.97896E-41
PPP1R1C	0.789261483	4.23372E-08
PANK1	0.788981184	1.79397E-15
CDHR3	0.788759417	8.72175E-05
GLTP	0.788711945	1.44005E-37
BX255925.3	0.788292274	5.24584E-13
IL12A	0.787824083	0.000235854
HIP1R	0.787429074	5.30928E-37

STBD1	-0.594043166	4.19252E-08
SUMO2	-0.593967867	3.86472E-33
TRAF2	-0.593512114	2.77382E-13
TBCA	-0.59344171	9.51249E-17
DNMT1	-0.593319787	6.06038E-19
C1orf61	-0.59291038	0.019257442
C17orf113	-0.592584133	0.011847262
RABEP1	-0.5923675	7.99313E-22
FKBP9	-0.592300612	7.23872E-31
PPP1CC	-0.592064929	7.88867E-33
NIF3L1	-0.592019637	3.84042E-14
DNAH14	-0.591744777	4.74414E-08
ASXL2	-0.591526414	2.45046E-18
TRA2A	-0.591038525	3.36624E-20
TCEAL8	-0.591021059	1.7026E-18
EXOC6	-0.590888112	1.14857E-09
RAD51AP1	-0.590831133	6.60203E-12
STRBP	-0.590721401	1.2465E-15
USO1	-0.590688629	9.63874E-17
FANCI	-0.590419564	5.41653E-20
TMEM38B	-0.589839131	5.0327E-10
CTHRC1	-0.589724841	7.65608E-05
CDKN2A	-0.58969986	1.96318E-10
FAIM	-0.589423636	6.96822E-07
TOMM6	-0.589127417	8.20873E-11
MEX3D	-0.589007672	1.9015E-10
SLC16A7	-0.588819231	1.07198E-06
KANK1	-0.588510919	4.05368E-14
DIAPH2	-0.58832231	1.76971E-11
NDUFB6	-0.587666209	1.78587E-11
METTL18	-0.587602252	0.000199376
SIAH1	-0.586983215	4.47241E-10
TMEM147-AS1	-0.586584013	9.40186E-10
CENPL	-0.586564617	5.10427E-06
NLRP2	-0.586553654	2.50227E-24
CHAC2	-0.586529335	2.55128E-06
BUD23	-0.586518504	3.54872E-24
SLC25A19	-0.586197915	3.58821E-13
LARS1	-0.586145724	3.2496E-21
FAR2	-0.586080545	9.30759E-06
MPHOSPH6	-0.585782361	1.17931E-13
ZNF181	-0.585411563	0.0001419
KDM3A	-0.585323183	1.89191E-16
IARS2	-0.585084763	6.15839E-23
EIF1AX	-0.584971471	9.37627E-17

TRNP1	0.785973536	3.13359E-29
AC109460.3	0.785815334	0.005442553
ZNF875	0.785570475	1.65087E-07
MINK1	0.785367949	5.90149E-38
INSYN1	0.784444495	0.004788537
AC145098.2	0.78345097	0.004185211
SEPTIN9	0.78289049	4.45177E-40
NBPF10	0.782609475	9.76753E-19
KAT2B	0.782301626	6.23091E-12
NAPSA	0.781725354	0.000659118
IMPG2	0.781259962	0.004608858
AC015802.6	0.78121538	0.000613579
BLACAT1	0.781190854	7.64548E-12
COX19	0.779624726	3.25117E-19
AC104162.2	0.779340644	0.011042122
SLC30A1	0.778466793	1.38371E-27
GSE1	0.777727913	6.82283E-43
SLC12A6	0.777620282	2.71856E-27
GDPD5	0.777490123	1.38603E-21
ZNF586	0.777459733	0.000336177
ZNF70	0.777390099	1.93321E-11
BMP7	0.776677356	2.47542E-19
FAM120C	0.775940256	2.7157E-05
SARM1	0.775293123	0.00091148
ATP6V0C	0.774955667	6.85674E-27
C1orf122	0.774944262	3.47343E-12
S1PR2	0.774875319	2.00703E-13
MMP25	0.774266442	0.009558712
PMS2P3	0.774135565	7.3305E-05
SEC31B	0.774006932	2.67843E-26
SCRIB	0.773906225	1.28941E-24
TNIP1	0.773834365	1.04212E-38
HERC6	0.773481352	1.19944E-16
NDRG1	0.773348619	2.8462E-17
DNAH17	0.772724098	0.007931042
TNFAIP1	0.772602826	1.80184E-45
HMOX2	0.772130975	2.0965E-35
PHLDB1	0.771387292	6.97711E-41
SZT2	0.771272592	6.42002E-24
BLVRB	0.771022291	4.35129E-26
KCNN4	0.770405376	6.46113E-28
ELL	0.769839391	5.45749E-27
AC132872.5	0.769697206	0.000369258
LHX4	0.769295255	0.001786244
PLD1	0.769036234	1.11158E-16
ITGA7	0.768983259	3.07166E-20
AC021218.1	0.768821322	0.000148877
ARFGAP1	0.768778679	5.31012E-27
TDRD6	0.768727779	0.009103004
HSD17B14	0.767970734	0.002943155
AC004816.1	0.767285524	0.00275736
LIME1	0.766895128	1.19461E-12
DDIT4	0.766891468	1.69852E-30
MS4A15	0.76687697	0.00022652
LINC01001	0.766352473	5.69006E-06
RPL32P3	0.766206599	7.53619E-07
NBPF19	0.766025532	3.53742E-07

CERS5	0.765816873	2.87537E-28
SPEG	0.765597928	1.34623E-26
PLCB4	0.764410721	4.78668E-09
MID1	0.764134575	7.64815E-22
C17orf100	0.763819369	0.001233175
WDR1	0.762515078	3.89026E-56
TICAM1	0.762311327	5.44621E-18
MRC2	0.762234118	1.65336E-15
ATG2A	0.762014033	3.08245E-13
KCNJ12	0.761247702	2.89867E-18
ISYNA1	0.760779997	1.53698E-25
DNAJC30	0.76065476	1.03587E-11
AC078820.3	0.760603829	0.006308524
DISP2	0.760505149	2.03856E-10
AC132812.1	0.759625388	0.001044939
C12orf45	0.759073925	1.27913E-16
AL135999.1	0.75893164	1.3184E-06
COL5A2	0.758729562	0.00015452
ABCA7	0.758577602	1.43734E-29
GAS6	0.758268624	1.48582E-32
CTF1	0.757858798	0.005091895
PLD6	0.757308044	1.18013E-20
ZNF783	0.757287916	1.07417E-11
ARHGAP31	0.757190796	4.74081E-06
POLN	0.756883987	0.013602628
SHANK2	0.756866897	3.75292E-16
CLDN23	0.756165555	7.41822E-07
SAV1	0.755078315	2.53708E-40
PRKX	0.755074462	1.31868E-40
RECQL5	0.755040013	2.55978E-34
SPAG1	0.754803573	1.69303E-14
PARD6B	0.754239408	1.60237E-13
ATP6AP2	0.754059105	3.07633E-35
AC008105.1	0.753984863	0.014897754
CYP2D7	0.753781901	0.000372394
SLC5A5	0.753706613	0.001170503
SLC49A3	0.753084757	0.003013649
TPM1	0.752399022	5.04262E-25
CCDC3	0.752345378	1.47333E-13
PLEKHG5	0.751679501	3.8132E-19
SLC20A2	0.751116221	1.66162E-24
SUPT7L	0.750427146	3.06658E-34
AC078923.1	0.750125404	0.003643914
LRRC24	0.750110051	0.000496387
AC009237.14	0.750079374	0.000813828
RASSF5	0.749538889	2.73294E-10
AC008105.3	0.749030889	0.016517932
PKD1P1	0.748893942	0.002250464
ITGA2	0.748892583	3.59264E-24
PDIA3P1	0.748571384	0.002651501
WASH5P	0.748216803	6.74999E-08
SRSF8	0.74812552	1.03529E-33
VPS18	0.74753333	1.86361E-16
ABCD1	0.747220918	2.79046E-13
LINC01002	0.747140696	2.03346E-05
PBLD	0.747046702	0.00114129
YPEL5	0.746829272	2.81426E-22

SKIDA1	0.746817339	0.013018837
MAPK8IP3	0.746589767	7.86265E-32
JDP2	0.746582569	2.96162E-13
DHDH	0.746197105	4.6776E-07
MLH1	0.745774993	9.23687E-09
ZNF324	0.745610804	7.90572E-12
ZDHHC24	0.745431536	1.08723E-13
PLEKHB1	0.745406625	1.14475E-27
RSRP1	0.745166047	1.281E-24
AC126755.1	0.744930187	5.19429E-14
AATK	0.744722033	2.29833E-08
PTK6	0.743581179	1.80215E-09
ROBO3	0.743472732	4.92607E-18
HOXB5	0.743457963	1.74001E-18
FHL1	0.742065567	4.84959E-20
DLK2	0.741840114	5.55569E-05
PLXNB2	0.741786508	1.21664E-26
BBS2	0.741462563	8.69583E-32
DNAJC3-DT	0.741319994	0.00701981
AC010973.2	0.740486408	0.017165374
AUXG01000058.1	0.740397915	0.006069374
IFIT3	0.740314172	5.00296E-08
MYO15B	0.740050859	1.04355E-10
RIOX1	0.739861125	1.95644E-05
CCNK	0.739694428	4.77145E-47
CIAO2B	0.739452714	5.0246E-18
TNS4	0.738061697	7.33301E-28
ZNF516	0.737732988	1.90577E-24
ASPSCR1	0.737395989	6.46453E-31
PLA2G4B	0.736885341	0.000130802
SYNE3	0.736817938	2.35645E-14
ARHGAP8	0.736473788	2.47458E-25
RBM4	0.736411549	4.27546E-35
IFIT1	0.736191942	1.04307E-12
AC040160.2	0.736180232	0.005972901
MOB3C	0.736121956	3.07799E-16
SORBS1	0.735041862	2.61031E-10
SDCBP2-AS1	0.734652076	0.000184324
RNF114	0.734466933	5.3999E-54
SGSH	0.733864331	9.7238E-16
CAMTA2	0.733777769	5.15999E-22
FAM95B1	0.733251491	0.000289316
FAAP20	0.732055982	8.1592E-14
GOLT1A	0.731941949	0.000173137
LMNTD2	0.731741952	6.86699E-05
BCL9L	0.731561741	1.71227E-28
KCTD21	0.73140714	8.20032E-08
RNF207	0.731389748	5.5689E-22
UBALD1	0.731225181	1.52513E-12
ANKRD11	0.730354504	6.61518E-48
POLR2A	0.730008894	1.79231E-30
ANK2	0.729891689	0.002499657
C3orf67	0.729682756	1.44682E-07
ZNF276	0.729503799	1.79519E-29
MRNIP	0.729419627	6.56434E-17
GUSBP11	0.72857482	1.58307E-15
PPP2R5B	0.728404603	1.8936E-15

ZNF37A	0.728398142	1.14993E-24
CASTOR1	0.728017485	0.00043267
SLC9A1	0.727994306	1.83676E-12
THEMIS2	0.727233559	2.9253E-12
RNF39	0.727174883	0.004886029
NICN1	0.727162344	2.2664E-05
AC245060.4	0.727129486	7.08962E-08
ABL2	0.726710213	4.56065E-21
IGF2BP1	0.726380171	0.002970681
TBC1D10A	0.726271484	5.96849E-15
EOMES	0.725903742	1.15747E-05
TET2	0.725724307	4.05456E-14
ACTN4	0.725533668	4.90728E-46
PARP3	0.725295892	2.16608E-15
SNX21	0.72520402	8.55992E-13
HMGA2-AS1	0.724533594	0.004999732
TNRC18	0.724510324	7.10745E-20
CXorf40B	0.724135084	7.21246E-13
RAMP1	0.724114634	6.8662E-09
FP671120.5	0.724070509	1.21276E-05
DLG5	0.72387507	5.63665E-34
CLK4	0.723850514	5.27762E-14
ZNF816	0.723533587	0.000311874
AC040162.1	0.723440943	9.78542E-05
LINC01123	0.72340945	8.38889E-13
B3GALT6	0.723399358	2.3597E-14
ANXA9	0.723353473	0.000495291
CLP1	0.72291053	4.26444E-26
RPL23AP7	0.721766535	2.81131E-07
H3C6	0.721480584	0.000177527
MAFF	0.721156368	1.18533E-21
CROCCP2	0.720919869	1.20254E-17
MAP2K3	0.720774376	6.45089E-34
SLC4A3	0.720269227	1.159E-18
MATN2	0.719648152	8.92098E-10
TAGLN	0.719486705	0.000195221
RRN3P3	0.719442358	0.000635026
CYTH4	0.719299055	0.000423014
NKD2	0.719012318	2.26627E-13
SHB	0.71821931	1.2572E-19
GRIPAP1	0.718151533	6.41094E-38
SAMD12	0.717844283	6.36385E-14
SH2D5	0.717556519	1.45008E-10
LNCAROD	0.717195421	4.19112E-13
MAP3K9	0.717150471	2.76954E-21
MAFK	0.717034726	1.60805E-20
CHD5	0.71654583	4.04986E-16
GFOD1	0.716093875	1.37145E-08
GOLGA6L5P	0.715490579	2.2552E-06
FLNA	0.714885083	8.77008E-32
SHANK1	0.714618551	0.003506695
KLK10	0.714181397	2.65891E-16
LINC01876	0.714068356	0.021491388
PADI2	0.713763579	0.000213349
ADRM1	0.713481254	5.05352E-21
FCHSD1	0.712947383	7.77987E-20
LYPD5	0.711851789	1.65437E-05

LCP1	0.711453101	5.215E-05
SARS1	0.711137153	5.53952E-38
TMEM92	0.710991831	3.40307E-09
NIBAN1	0.710487105	1.69845E-13
INTU	0.70983568	9.79734E-15
C12orf4	0.709000496	1.93935E-14
SLC46A1	0.708995316	5.0631E-19
ZHX3	0.70894581	3.24331E-21
NEDD4L	0.708190741	1.62854E-34
PCSK7	0.707962344	2.067E-24
DMTN	0.70779085	1.82855E-22
RHOBTB2	0.706953985	2.95763E-18
DEF6	0.706945441	7.02517E-23
PARVA	0.705962634	1.84603E-28
MED20	0.705902497	1.50386E-25
ASNS	0.705857445	7.40242E-15
ELFN2	0.70578695	1.23523E-35
PPP1R15A	0.705690849	2.94772E-25
SLC26A11	0.705164877	1.1622E-12
FAM102A	0.704647998	3.21742E-25
ABCG2	0.704285254	1.47022E-05
RBM14	0.704167787	4.15884E-25
AC245060.7	0.70353016	2.26743E-05
C1R	0.702638891	3.0656E-05
SHANK3	0.702523215	1.83999E-15
SPATA20	0.702502485	1.06998E-28
AC009690.2	0.702253221	0.014234281
GANC	0.702148644	3.80202E-10
INTS6L	0.701686973	1.14498E-08
IL17RE	0.700513498	8.67705E-10
KDM5B	0.700510448	1.63094E-24
SHROOM3	0.70035114	8.93098E-33
TUBGCP6	0.70019981	2.01355E-28
NIBAN2	0.699779404	1.67804E-33
PRKAG2	0.699692532	1.91941E-24
AL137782.1	0.699625832	0.01358132
ESPN	0.698722028	1.43099E-16
SOX9-AS1	0.698443606	7.00918E-05
ACBD4	0.698023453	3.32951E-09
CLBA1	0.697549361	2.50509E-16
SYNC	0.697392736	2.53525E-08
AL136295.5	0.697339247	0.024659737
AL359715.1	0.696723827	0.00438225
USP2	0.696466169	9.76017E-07
ZNF746	0.696459606	2.0912E-26
CDH1	0.696214111	4.20554E-35
CST3	0.695545299	4.7008E-16
LINC01410	0.695538018	0.033849473
POLR3GL	0.694646446	3.54452E-18
ZFP90	0.694155336	1.55612E-20
TSNARE1	0.694044771	1.88868E-10
MICAL1	0.693847358	4.7875E-25
AC135048.4	0.693736685	0.008312382
TBC1D3L	0.692867009	1.66976E-17
LINC02163	0.69268947	0.037800946
CYP4F2	0.692562928	0.002816559
VOPP1	0.692288025	1.0369E-31

RNF185	0.691805322	1.46713E-19
C18orf25	0.691728591	9.92716E-24
GABPB1-AS1	0.69100687	4.19215E-06
NBPF26	0.690985936	2.17895E-16
CARS1	0.690509535	6.96348E-34
AC020916.1	0.690490585	9.66128E-06
SLX1B-SULT1A4	0.689771759	6.02445E-09
TM7SF3	0.689517191	2.40054E-33
L3MBTL1	0.688773528	1.07138E-11
UTRN	0.688661978	1.04524E-14
LAMC1	0.688468991	1.47145E-52
DMAP1	0.688156804	4.38862E-22
S100A16	0.687094598	6.19544E-29
ANXA2	0.68694978	2.09103E-62
PMS2P4	0.686847317	0.018065504
LINC00910	0.686600506	0.005361465
AC008038.1	0.686463282	0.038419215
AC074143.1	0.686284921	0.000161247
AC207130.1	0.686246032	0.000292394
PCYT1A	0.685132755	6.52044E-35
ZFHX2	0.684984067	0.001904464
NUDT16P1	0.684750888	2.38765E-05
STAT2	0.68421305	3.71886E-26
IFI27L2	0.684212777	2.70877E-07
AL133216.2	0.683175546	0.000571636
C11orf45	0.683150253	1.20691E-05
SLC35G2	0.682806058	0.000570917
MBD6	0.682666087	2.08297E-12
MAP3K10	0.682510599	4.81371E-13
MAPK8IP2	0.682053181	6.37753E-08
SCD5	0.681243728	1.34246E-24
GRN	0.681053712	1.10019E-19
TGFBRAP1	0.680806297	6.006E-30
MIR193BHG	0.680687527	0.019045033
ZBTB17	0.680058329	3.06331E-18
NGFR	0.680006864	9.02123E-18
DDA1	0.679096051	1.91687E-23
STAT6	0.679066116	4.47621E-34
SLC9A3R2	0.678628772	5.79756E-15
TAX1BP3	0.67801706	1.01171E-16
RABEP2	0.676512295	5.65852E-13
ADAT3	0.676256021	6.73902E-06
AC090114.2	0.675952878	0.032391429
MCC	0.675858885	2.39359E-25
OXTR	0.675806581	0.018079084
AMIGO2	0.675642644	0.001284794
MLYCD	0.67540562	1.42279E-13
CAPG	0.675304192	8.42819E-24
MTO1	0.674849019	1.81663E-24
CCDC120	0.674735916	5.22187E-10
MCF2L2	0.674671189	0.008932998
SLC39A13	0.674629514	7.19967E-18
KCNB1	0.674241373	2.72772E-05
KDM6B	0.674020432	5.05014E-15
AGAP2	0.673561082	8.7663E-18
LRATD2	0.67346883	2.25811E-27
ATXN7_2	0.673435165	8.10896E-05

CXXC4	0.673265079	0.00150813
SHC1	0.673239734	2.59445E-46
CLIC3	0.672873049	1.59036E-05
ZNF585B	0.672609046	1.57293E-07
RPL13P5	0.67258397	0.000375299
LIMK2	0.672228977	2.41672E-19
ZNF469	0.672132419	7.41107E-07
DVL3	0.671792629	8.14897E-27
AL022328.4	0.671773311	0.006158394
BIK	0.671722323	1.06866E-11
TRADD	0.67161671	6.26279E-14
ABCC5	0.671493141	6.64286E-19
BCL10	0.671249372	4.96171E-19
LTBP3	0.671212091	8.42432E-25
USP18	0.671125774	1.19327E-10
CDCP1	0.670306201	2.21624E-35
FAM27C	0.670058034	0.026947579
PNPLA7	0.669363756	0.003079185
ZBTB7B	0.669179588	5.81218E-18
MED15	0.668971141	7.27814E-22
CCND3	0.668219701	7.62464E-21
RGS14	0.66768636	2.7383E-08
NPEPL1	0.667666933	1.32937E-14
AC004980.1	0.66747534	0.005925131
ZNNT1	0.667159965	0.000174278
TMBIM1	0.667071407	5.22031E-45
AC138969.1	0.66660381	5.12475E-05
CLGN	0.666535116	0.00014612
FAM184A	0.666071356	0.012786843
ZNF28	0.665953505	5.00672E-14
LYPLA2	0.66585563	4.12439E-25
POMK	0.665776528	0.000183165
GSN	0.665191959	1.73266E-21
AC002066.1	0.664986146	0.028228129
C16orf70	0.664723175	2.68554E-16
SNX18P3	0.664672951	0.003759659
AC093724.1	0.66456168	0.04577093
AKIP1	0.663961358	1.01289E-17
NPIPB12	0.663802128	1.36548E-07
COMMD5	0.66307291	1.49355E-11
ADGRB2	0.662729665	2.35685E-14
AL358472.6	0.662575688	0.000910996
GTF2IP1	0.662485142	1.92437E-12
C1QTNF1	0.662458754	3.27019E-06
TEPSIN	0.661255646	1.57061E-11
AC242376.2	0.661121431	0.005071483
TERF2IP	0.660370433	1.30397E-35
CCDC142	0.66035709	1.0754E-09
ADAMTS13	0.658680144	1.03587E-09
AC108488.3	0.658199828	0.00796611
BVES	0.658190727	7.44027E-06
TMEM234	0.658178694	4.4781E-07
AC026362.1	0.65807873	2.65318E-06
SAMHD1	0.657895871	2.42862E-20
AC018647.2	0.657817472	5.67496E-07
PRRT4	0.657668603	1.43964E-05
LINC02700	0.657440568	0.000120091

TNFRSF25	0.657294426	1.26865E-11
AMZ2	0.65682295	3.95592E-34
DCTN5	0.656678768	5.19953E-37
HSD11B1L	0.656447224	0.001167028
AC012313.1	0.656238766	0.046551854
ADORA2B	0.655342471	1.15425E-22
WHRN	0.654961188	1.61829E-12
GIT1	0.654793561	3.99367E-24
MRI1	0.65448228	1.65031E-14
NUDT14	0.653603591	6.91727E-12
GRAMD4	0.653224695	1.12288E-15
CLDN15	0.652849197	1.96629E-07
TUBBP5	0.652618081	9.24505E-05
PRSS8	0.651981015	1.02461E-19
VIPR1	0.651962713	2.68781E-06
AC112128.1	0.650832601	0.00497593
PLA2G7	0.650809203	7.57136E-07
SLFN5	0.650561588	1.55726E-15
HOXB-AS3	0.650488758	0.024961651
NBPF20	0.650468185	4.07807E-10
NFATC2	0.649931295	2.66369E-12
RBM47	0.649372462	9.20311E-22
ALDH2	0.649141272	9.03725E-22
CTTN	0.649079459	1.58894E-38
CTDSPL	0.648901472	5.67559E-31
DOP1A	0.64873605	8.01201E-08
CRABP2	0.648446047	7.74967E-24
KCND1	0.648420592	6.97341E-05
NUTM2A	0.648009482	0.030898089
RRP12	0.647847916	4.33494E-32
AC108010.1	0.647543274	0.000917577
RRBP1	0.64727617	4.27222E-34
SEC61A1	0.646651441	1.53452E-49
MT2A	0.646215731	0.003562948
UPK3BL1	0.645999805	0.000465234
BLOC1S3	0.645612371	4.4877E-09
SOCS4	0.644681124	7.95687E-15
TANGO2	0.644312266	1.31636E-10
SNHG20	0.643704023	6.05398E-07
PIK3R3	0.64359024	6.60417E-06
AC006128.1	0.643471608	0.000494837
NECTIN1	0.643458726	4.4224E-15
CYB561D1	0.64343192	1.14694E-09
CASP10	0.64343126	4.19146E-08
INPP5J	0.64263004	5.51978E-08
RAB19	0.642527997	0.001421277
AOC2	0.642360491	3.49962E-05
CARD9	0.642058045	0.011195117
TMEM131	0.641603124	6.06301E-37
AREG	0.6411174	7.33622E-27
HOXB13	0.640956729	7.88717E-14
MARVELD2	0.640783954	1.44906E-19
UBE2J2	0.640524508	1.87169E-18
GPC2	0.640098938	0.000139763
AL365205.1	0.639899198	1.66456E-14
AC012313.8	0.639791591	0.023159968
ERCC6	0.639787389	5.25796E-20

RILP	0.639528007	1.11788E-06
APTX	0.639369934	4.41258E-22
PLIN4	0.639014463	0.000187712
ZNF503-AS1	0.638218208	3.99751E-06
CDK18	0.638195729	1.1669E-20
JAG2	0.638023589	2.23199E-19
MORC4	0.637718475	1.70427E-28
LIPH	0.63768351	7.65233E-13
CXorf40A	0.637514733	1.27246E-06
ATF7IP2	0.637224485	3.40919E-06
ZNF205	0.636592111	4.71065E-13
FBXL2	0.636252157	2.35191E-12
FBLN1	0.636230203	6.27445E-18
INE1	0.636195442	0.017977554
AL669831.1	0.635919429	1.51437E-09
SKIV2L	0.6355831	6.3289E-26
ELOB	0.63555224	2.08333E-05
PHRF1	0.635191737	5.95358E-19
EI24	0.63496914	2.96598E-55
ENPEP	0.634729735	6.39763E-05
YJEFN3	0.634695241	1.66829E-10
ZNF594	0.63452869	0.000359297
EXOC7	0.634469269	1.5704E-39
WDR73	0.634440254	4.64186E-14
PIGG	0.634130844	1.47351E-22
ATP6V0D1	0.633797206	3.31228E-21
LINC00342	0.633223566	0.000374247
ARHGEF6	0.632361106	2.92229E-05
SLC2A11	0.632255703	2.40455E-10
GAS6-AS1	0.632050579	7.6036E-09
SH3BP4	0.631790579	1.27782E-22
BAIAP2L1	0.631404155	5.69724E-43
KLHL31	0.631379489	0.014686564
RHPN2	0.6305808	2.78231E-30
RBM38	0.630283569	1.57524E-23
RGL1	0.630130395	0.000174774
ZNF517	0.629867572	1.12737E-05
ENTPD2	0.629217585	2.64705E-11
MAN2C1	0.629184197	2.70487E-19
FSCN2	0.628831922	0.043932253
LINC01271	0.628749126	0.004362593
ZFAND2A	0.62863072	1.30054E-08
LMTK3	0.628569658	3.37386E-10
LENG8	0.628558032	1.37013E-16
BSCL2	0.628228099	9.00702E-06
NPDC1	0.627816718	3.22227E-12
HDAC10	0.627798842	4.75267E-13
COMT	0.627257548	1.99237E-17
CDK5	0.627163221	8.1098E-10
HSPA12A	0.627107963	2.26182E-27
MTURN	0.626418551	5.72645E-13
S100A14	0.62631636	4.06818E-29
BX537318.1	0.625300279	0.014584517
TMEM127	0.624767181	4.06697E-24
SEC14L2	0.624525295	8.95088E-18
ALOX12B	0.624335097	0.032549196
TNFAIP2	0.62426486	1.91119E-22

FAM222B	0.624053806	1.78701E-18
HSD17B7	0.623929825	2.01162E-11
SEPTIN4	0.62392692	4.13684E-06
DTX4	0.623792776	4.0414E-10
SLC17A7	0.623620841	0.002865941
URB1	0.623303126	4.06406E-25
FAM13A-AS1	0.623288738	0.039117941
DNAH6	0.623143503	0.010178452
NACC2	0.622323857	2.78218E-17
EMC3-AS1	0.622008478	0.011375372
EIF6	0.621740832	6.13535E-22
AC119673.3	0.621518799	0.000650738
PRICKLE3	0.621396581	2.26924E-13
FLII	0.621181155	5.02747E-33
DNMBP	0.620883904	5.33745E-33
ZNF319	0.620552124	5.92215E-10
RCL1	0.620518655	4.53431E-24
YY2	0.620291332	0.007931643
FCHSD2	0.619957105	6.95418E-18
CRLF1	0.619608238	2.62563E-15
DLX4	0.619460342	5.96244E-09
OTUB2	0.619278463	3.75202E-11
HOXA6	0.61926148	0.005809681
ZNF596	0.619006005	0.00060295
AL161891.1	0.618709872	0.013923162
AC100861.1	0.618644953	1.01663E-11
PBXIP1	0.61850087	4.98198E-13
REXO2	0.618481699	1.33076E-29
ABCC3	0.618415354	6.53974E-17
TRIM46	0.617876661	3.88925E-05
CDA	0.617216287	2.83289E-12
TMEM171	0.616848553	7.78613E-05
GPR176	0.616275334	0.017308873
MAP11	0.615857707	1.10186E-08
AP003071.5	0.615601431	0.002430433
RAB37	0.615457494	3.81587E-11
GPR155	0.614630835	1.47166E-07
RSKR	0.614271427	0.000336417
CCDC159	0.613119821	0.029122994
ZNF343	0.612971094	4.31664E-12
OBSCN	0.612888051	8.80005E-11
ZBTB38	0.612492583	8.25289E-17
TMEM45A	0.611848205	3.35075E-07
ZBED6	0.611750424	0.007455353
ABTB1	0.611313587	3.86794E-11
ZNRD1ASP	0.610219194	0.000128184
FXYD5	0.609942113	4.08734E-16
AC132872.4	0.60991885	0.011386447
AC005747.1	0.609815417	2.48921E-07
TRIM15	0.609241038	0.000151784
ZER1	0.608722979	5.99727E-20
DGLUCY	0.608620313	2.59319E-24
KCNH2	0.608402313	4.6499E-10
AIFM3	0.608027105	0.000709611
ZSWIM8	0.607944195	3.27221E-22
GLCCI1	0.607737667	7.76797E-06
CD59	0.607380129	3.40005E-32

ANKRD44	0.60735332	0.006403923
ZNF195	0.606924927	1.98933E-15
PARM1	0.606814652	1.83137E-16
SYNGAP1	0.606613639	4.75513E-13
AL157935.3	0.60638153	1.16087E-06
ADPRHL1	0.605310104	7.75849E-11
ZNF75D	0.605033658	1.48568E-06
STK11	0.604881331	2.68167E-21
TANC1	0.604863884	2.01716E-18
BRSK1	0.60448633	5.01097E-10
MINDY3	0.604189566	1.32186E-13
MICAL2	0.603561653	6.38595E-15
SPG7	0.60312634	7.93574E-30
SLC4A8	0.602946974	0.009587688
AC040160.1	0.601792299	9.94788E-18
ZRSR2	0.601617532	3.14379E-07
ZNF692	0.601580652	6.70546E-14
PODXL	0.601563308	3.59479E-19
CORO2A	0.601430888	2.9764E-15
RGCC	0.601292774	0.002655067
GSTM2	0.601158625	0.00142772
FRMD5	0.601118087	4.61545E-18
GUCA1B	0.601059488	0.003973204
ZNF416	0.600725113	0.011048937
ZMYND10	0.600655845	0.020991021
AP001453.4	0.600422804	0.001085869
CDC42SE1	0.600140466	1.04838E-25
AC118344.4	0.599624082	0.031266959
EPB41L4A	0.599280211	0.000620075
ELMOD3	0.598967278	5.81143E-07
AC018628.2	0.598797471	0.038792288
OVGP1	0.598728034	0.002230632
TSC2	0.598426802	6.08839E-19
SP100	0.598394179	4.96824E-20
CREBRF	0.598176487	2.88604E-07
ZNF774	0.598014802	0.002486505
CEP164	0.597850095	2.61872E-13
DUXAP8_1	0.597605407	3.82108E-07
ZNF408	0.597470037	1.77622E-09
SNAPC4	0.596912052	3.17853E-17
DMWD	0.596179124	3.52309E-09
CAVIN1	0.596089396	1.01821E-21
SHFL	0.59606684	1.48219E-11
PYROXD2	0.595831827	3.36676E-05
PIK3R2	0.595332688	3.68588E-13
SLC2A6	0.595074658	5.0087E-08
TPST1	0.594954784	3.94054E-10
ASB6	0.594879929	1.64401E-19
UBALD2	0.594460183	1.60512E-14
HTR1D	0.594003077	1.31284E-05
DGCR6	0.593434021	3.79266E-07
FOXD2-AS1	0.593094764	0.000551733
LINC00346	0.592959467	1.18141E-07
HEMK1	0.59291873	3.92367E-14
CBR3	0.592623006	0.010491322
EML2	0.592119555	1.42294E-22
BSG	0.592094897	3.93559E-21

CA12	0.59191219	1.05247E-10
HAS3	0.591287101	6.76065E-22
L1CAM	0.59099469	2.20762E-14
FERMT1	0.590980772	5.17714E-29
NME1-NME2	0.590870353	3.53602E-11
ALKBH6	0.590766239	2.31927E-09
CEP76	0.590723975	4.35585E-12
TNRC6C-AS1	0.590552999	5.58211E-05
ZP3	0.590402412	2.74208E-05
CXCL16	0.590381837	8.78969E-20
AC009133.1	0.590259553	0.000480366
RAD52	0.590084132	4.25475E-10
PCGF3	0.589741169	3.64638E-35
COLQ	0.589698773	0.044216194
EXOSC1	0.589575281	1.45651E-11
TTC9C	0.589381675	1.97756E-10
HECTD4	0.589304974	1.22689E-14
BCAM	0.589223333	1.15066E-15
FAM161B	0.589020532	6.85345E-06
MINCR	0.588984606	0.000244255
SMIM29	0.588839645	5.35861E-07
PALLD	0.588732364	1.42449E-31
AHNAK	0.588630826	1.80139E-23
POLM	0.588483312	4.3922E-09
OSBPL5	0.588469659	6.29073E-16
ZNF585A	0.588378054	0.00366573
CTSK	0.588228879	0.012596938
PAG1	0.588182512	0.000316226
ZNF341	0.587327968	1.52424E-06
C3orf62	0.587219087	1.25306E-05
ALDH1L2	0.58689676	0.024659737
AKAP8L	0.586434117	1.34169E-27
MECOM	0.586401417	0.000102732
CLIP1	0.586354922	8.12498E-28
DAB2	0.585765833	1.74303E-07
AL162595.1	0.585439184	0.001070189

Table S9. List of mRNAs significantly regulated in HCT116 *miR-34a/b/c-KO* cells when treated with 5-FU.

Significantly upregulated mRNAs			Significantly downregulated mRNAs		
Gene symbol	Log_2 fold change	padj	Gene symbol	Log_2 fold change	padj
PTAFR	5.149509802	0	*RPL22L1*	-2.612541099	1.6737E-181
NECTIN4	4.969374672	2.4153E-197	*LONRF2*	-2.44739583	8.62798E-32
CEACAM1	4.675382604	0	*SEMA3D*	-2.345342496	1.55382E-25
DRAXIN	4.357540403	0	*SCD*	-2.127969365	0
COL17A1	4.249458085	0	*SPARC*	-2.08112191	1.29643E-24
H19	4.012708136	8.4887E-262	*LRRC4B*	-1.870703667	2.81706E-15
LAMP3	4.007585967	0	*SEMA3A*	-1.861708913	2.8686E-111
HMCN2	3.796611135	6.9003E-91	*PIF1*	-1.853579138	1.85227E-36
LAMA3	3.552158491	0	*OLMALINC*	-1.852570251	9.5194E-104
GPR87	3.530223962	3.5108E-132	*CAVIN3*	-1.850558253	1.27698E-61
KRTAP2-3	3.521576413	5.3653E-182	*PEG10*	-1.84887722	1.57915E-78
WNT7A	3.437282031	2.1329E-205	*PRKACB*	-1.845366989	1.3867E-215
KLHDC7A	3.410316405	3.61392E-67	*ETV1*	-1.819880468	5.37174E-55
MYH16	3.239499019	2.7867E-204	*ARMC4*	-1.7355573	2.402E-74
SERPINB5	3.168956883	0	*IFITM1*	-1.723761481	2.7739E-20
ECM1	3.119923509	1.16755E-97	*IQGAP2*	-1.721157971	1.85385E-11
CCN1	3.110504976	0	*SLC16A6*	-1.709637793	3.33627E-51
CD82	3.091540835	0	*FOXC1*	-1.702461764	9.9218E-146
PRXL2A	3.036170022	1.3396E-54	*DDN*	-1.68329436	2.46995E-41
PROM2	3.013222145	5.0308E-124	*RGS2*	-1.670257137	2.32396E-89
ACHE	2.989112497	8.402E-104	*LINC00482*	-1.668891001	3.15817E-15
GJB4	2.966738836	1.01973E-60	*NREP*	-1.663322679	5.38915E-23
PRODH	2.94978646	1.1417E-187	*PDE4B*	-1.649385682	1.5242E-120
PRDM1	2.937820381	4.77412E-51	*RORC*	-1.643342989	7.2259E-12
TP53I3	2.896489569	0	*GIHCG*	-1.637393979	2.0527E-11
SERPINE1	2.89037211	0	*SULT1C2*	-1.634103508	2.72988E-14
SFN	2.884413225	0	*ACOX2*	-1.63209568	3.14621E-19
DPYSL4	2.835815301	4.1313E-79	*DLEU2*	-1.625865459	3.88239E-40
GALNT5	2.76736722	0	*ARMH4*	-1.622482287	1.46232E-12
RRAD	2.703508239	5.80371E-66	*NR2F1*	-1.611753979	2.334E-133
SULF2	2.668781439	0	*RNASE4*	-1.608814387	5.29017E-16
PLXNB3	2.64334736	1.0657E-135	*INSIG1*	-1.60639288	2.2405E-119
TNFRSF10C	2.638358072	1.0615E-119	*VASH2*	-1.605015925	1.6166E-17
EGR2	2.63669388	1.33088E-35	*NECTIN3*	-1.601179778	9.5384E-131
PAG1	2.632151574	3.80168E-61	*L3MBTL3*	-1.598885239	1.8656E-99
NRP2	2.626655255	5.85946E-45	*ZNF775*	-1.589585815	6.71469E-17
CEL	2.625204387	5.4468E-116	*RASSF8-AS1*	-1.581024601	1.066E-12
TLR3	2.582424057	3.7136E-158	*CNPY4*	-1.579570358	1.50196E-08
WDR63	2.579486244	2.15653E-94	*VWA5A*	-1.576798098	7.65993E-21
TMEM40	2.572012881	2.728E-142	*PBX1*	-1.575378947	1.26602E-33
EPN3	2.568819019	7.2122E-189	*C22orf34*	-1.574488751	3.47876E-18
HBEGF	2.560547309	5.2074E-164	*HOXA4*	-1.562590871	1.46112E-10
ALOX5	2.559032828	1.43906E-66	*DENND5B*	-1.552694735	8.89834E-96
YPEL3	2.536260393	2.70126E-66	*SLC7A2*	-1.547406269	3.85096E-34
ETV7	2.521758241	5.42153E-32	*LINC01089*	-1.543924637	6.49417E-19
TP53INP1	2.517963643	1.5325E-295	*C1orf21*	-1.539394688	5.92816E-33
INKA2	2.509878034	3.6703E-193	*SERPINA5*	-1.53829839	1.6694E-25
FAS	2.499312924	8.0172E-289	*PLK1*	-1.534669446	9.5115E-107
CYP4F3	2.469404478	1.48661E-27	*PCDH7*	-1.528730684	1.4279E-107
CDKN1A	2.46560515	0	*MAN1A1*	-1.525286052	2.58498E-95
LTBP2	2.42346296	1.8357E-115	*LINC02458*	-1.520092986	3.53909E-13
C1orf116	2.4169318	1.3429E-171	*GRIN2B*	-1.519362702	2.08754E-42
AC007906.2	2.405803888	2.83514E-41	*NR2F1-AS1*	-1.518364604	1.12382E-59
INPP5D	2.397348996	3.1634E-86	*HFE*	-1.504681832	2.6519E-47
PPM1D	2.392627896	0	*SESTD1*	-1.503778403	1.54797E-49

LACC1	2.391779697	2.8499E-119	*ACSM3*	-1.500258534	3.59883E-44
PLK3	2.389064564	1.0989E-239	*SPACA6*	-1.498419046	5.29079E-14
GDF15	2.364308407	6.6525E-210	*BST2*	-1.495359109	6.51468E-16
ESRRB	2.347059231	3.10261E-66	*C6orf141*	-1.49223375	1.49748E-23
AP002761.4	2.340833479	8.04064E-48	*TNFSF18*	-1.487408589	6.21374E-65
PGF	2.332226956	2.68114E-97	*HSD17B8*	-1.484024278	2.22072E-28
STON2	2.312392276	1.78567E-25	*SELENOP*	-1.48361549	6.4051E-22
AL158206.1	2.312078551	6.15741E-82	*LINC00326*	-1.482610454	8.6689E-17
ACER2	2.303656755	8.1833E-101	*PTMAP5*	-1.480941161	4.17616E-06
SDCBP2	2.267798194	6.48065E-26	*SNHG19*	-1.472733671	1.19634E-07
MACC1	2.267345165	3.6273E-122	*ZDHHC2*	-1.461357473	3.19143E-89
TRIM22	2.25377355	9.97735E-32	*FBXO4*	-1.456153543	1.88151E-18
CCN2	2.250862629	8.81695E-67	*ZBTB12*	-1.455106073	3.30289E-25
SUSD6	2.246103226	5.8515E-259	*OR51B5*	-1.454942125	4.85407E-54
RINL	2.245595615	1.0882E-242	*SAMD11*	-1.453494154	8.93251E-08
LAT2	2.244269691	1.75008E-81	*RHOBTB1*	-1.444688717	1.52129E-14
BTG2	2.24149996	0	*CUTC*	-1.439700993	3.05377E-72
LYNX1	2.240821432	1.52447E-57	*ZNF704*	-1.438236166	3.9256E-85
SH2D2A	2.239535112	8.7137E-33	*VIM*	-1.429683582	1.50361E-22
CDIP1	2.221632142	4.16865E-84	*HNF4G*	-1.423221422	4.14006E-09
NRP1	2.220622096	2.70133E-63	*ARHGAP18*	-1.418817925	1.08638E-78
CYSRT1	2.199497537	4.20644E-39	*LRG1*	-1.418805617	4.09729E-08
RAPGEF3	2.187881833	2.52142E-37	*NFIB*	-1.4107044	2.2715E-58
CPA4	2.180579584	1.188E-285	*PPP1R1B*	-1.408475264	4.83024E-10
EPPK1	2.172516246	1.2842E-119	*DDIT4L*	-1.405958894	7.35758E-35
HES2	2.167432863	1.0122E-176	*KLF12*	-1.404240994	5.02797E-18
TRIML2	2.16694619	1.58933E-77	*LINC02609*	-1.399934756	2.24588E-06
C2orf88	2.164283441	4.58148E-43	*MBNL3*	-1.39981053	1.00534E-10
NANOS1	2.163429903	3.95146E-55	*SPEF2*	-1.395317653	1.10447E-16
ABCG4	2.150971453	1.7022E-74	*ANXA10*	-1.39193228	7.75575E-14
MDM2	2.139667162	2.134E-264	*KLRK1*	-1.37874883	1.61664E-06
FXYD3	2.134609636	1.58741E-76	*GBP2*	-1.37746908	9.29615E-09
BTBD19	2.126999369	1.68519E-54	*PELI2*	-1.37728761	4.85362E-15
CA12	2.122366846	5.9849E-121	*TRDMT1*	-1.376142116	1.29725E-23
EPS8L2	2.116670828	1.7421E-237	*MDH1B*	-1.375042019	1.3709E-07
GRIN2C	2.10977577	4.18286E-49	*C3orf80*	-1.372962307	1.43744E-09
PTPRE	2.108400874	1.014E-214	*HBG2*	-1.37087072	2.32534E-08
PHLDA3	2.105850423	8.3496E-137	*MKX*	-1.362837942	3.16876E-46
EFNB1	2.098598949	9.6004E-196	*MIR210HG*	-1.362704116	8.48059E-09
ZMAT3	2.09398753	2.5997E-246	*PDE11A_1*	-1.356532144	3.91565E-08
CD274	2.091115229	4.2988E-34	*SREBF1*	-1.351933541	2.8898E-148
PSTPIP2	2.083687068	1.5791E-274	*AK7*	-1.350651219	7.41539E-09
ISG15	2.079494254	1.5575E-208	*LRRCC1*	-1.342855224	6.29676E-39
UNC5B-AS1	2.072934174	1.12766E-39	*AC084018.2*	-1.339790195	1.56488E-09
HPX	2.069433606	1.30007E-21	*SAMD13*	-1.339136092	2.77333E-15
AL031587.5	2.062161336	1.44527E-72	*TMEM37*	-1.335940009	1.80976E-10
GJB3	2.059952675	7.7011E-144	*FAR2*	-1.323226	2.35237E-26
NRG1	2.043985122	2.47513E-28	*HSPE1*	-1.321038097	1.3686E-22
ATF3	2.043598156	1.3998E-192	*RHOBTB3*	-1.316759511	4.7267E-84
ALS2CL	2.041227844	2.9731E-175	*PHLDB2*	-1.311170654	2.2748E-156
FDXR	2.036163956	1.0175E-240	*ACSL3*	-1.306202653	3.1971E-110
ULBP2	2.034159615	4.5999E-114	*HOXD8*	-1.306078589	4.82825E-13
AL109976.1	2.032601952	2.10721E-30	*OBSL1*	-1.303713138	1.74518E-32
SERPINA1	2.029186898	1.368E-126	*TSHZ1*	-1.302845888	1.26602E-33
CHRNB1	2.025274101	3.9233E-224	*CKMT1B*	-1.30108676	7.7975E-34
AC005336.1	2.018706436	1.96855E-31	*PIR*	-1.300396151	6.36259E-44
SLC37A2	2.011225841	3.30793E-77	*NT5M*	-1.29946043	5.5246E-14
NKX1-2	2.005745987	2.7584E-94	*CDC20*	-1.295439169	7.37073E-69

APOBEC3D	1.997885618	2.27315E-27	*HOTAIRM1*	-1.288761644	6.95968E-07
ZNF79	1.996942014	2.67448E-77	*ANKRD22*	-1.288726477	1.22373E-09
PLCXD2	1.994173208	6.8679E-210	*METTL25*	-1.282896754	1.88378E-08
P2RY2	1.99036819	1.75939E-58	*AL590822.3*	-1.279752077	1.29477E-07
MYBL1	1.988641371	5.707E-108	*CPQ*	-1.277420853	4.41273E-08
SLFNL1-AS1	1.985257659	1.03211E-19	*KAZALD1*	-1.277013617	1.86839E-18
KRT15	1.981834795	4.5937E-194	*ADAMTS2*	-1.275416807	3.28218E-16
SESN1	1.978974747	1.5784E-198	*PALM3*	-1.272823245	9.00912E-25
MAPKBP1	1.976206273	3.9223E-189	*SLC16A13*	-1.270803709	4.69475E-10
GJB5	1.973303315	9.85295E-57	*MIR100HG*	-1.270325713	2.41853E-81
SLC31A2	1.969847947	2.7921E-33	*TMSB15A*	-1.262591384	3.17583E-13
SDC4	1.969684677	0	*EBF2*	-1.261126077	7.69682E-08
ZNF600	1.96188357	3.7203E-111	*KLF7*	-1.253883386	2.4056E-36
DGKA	1.953319424	0	*ENPP1*	-1.252311785	7.42515E-26
AC093866.1	1.952693981	1.28171E-69	*CYP24A1*	-1.252075234	1.7838E-124
AC016831.1	1.951509114	1.59821E-28	*ZFPM2-AS1*	-1.248595217	6.51297E-11
TP53INP2	1.944122767	9.65666E-92	*SLC43A1*	-1.246747581	9.55121E-11
RND3	1.941206324	2.4604E-108	*HLF*	-1.245435288	6.72222E-10
AL135905.1	1.940493367	2.61608E-22	*OR2A7*	-1.244218325	5.75419E-09
WNK4	1.940336913	3.44255E-29	*ST3GAL6*	-1.241411813	5.78188E-22
EMX1	1.938280783	6.2806E-30	*ABRAXAS1*	-1.237156808	8.29543E-31
E2F7	1.935741113	2.4817E-137	*RPL10A*	-1.232931277	4.6402E-128
SERTAD1	1.92694247	3.2626E-85	*TFPI*	-1.232623848	1.28185E-39
CYFIP2	1.925643466	0	*DPP4*	-1.230549634	1.40656E-13
SERPINB8	1.918749591	8.89181E-39	*AGA*	-1.230337404	1.40693E-11
PIDD1	1.915873852	2.8557E-138	*MARC1*	-1.226934653	2.33682E-31
NUAK2	1.915419972	2.78676E-62	*CYB5B*	-1.220046101	1.1546E-156
TBC1D2	1.909701142	5.8415E-115	*CU633904.2*	-1.215652869	0.002119803
SESN2	1.905644957	8.8093E-158	*NR4A2*	-1.211446004	6.4201E-28
WNT9A	1.896864314	1.6147E-100	*METTL7A*	-1.21067954	3.29955E-20
TINCR	1.892108047	4.10213E-74	*PTH2*	-1.209566701	4.13693E-05
ARG2	1.888388238	6.21891E-83	*TEKT4P2*	-1.208018146	4.33883E-10
IGDCC4	1.887566078	2.9238E-22	*LRRN2*	-1.207669725	5.56082E-20
ANKRA2	1.884789834	2.2116E-123	*ST8SIA6*	-1.205796099	3.78315E-16
PLCL2	1.881271435	5.1004E-61	*AC008014.1*	-1.204796802	1.28315E-12
DUSP14	1.875411488	8.9934E-192	*MPP7*	-1.201449981	1.05068E-44
COL16A1	1.861986989	7.91153E-50	*LDHD*	-1.196024855	7.19613E-13
COL7A1	1.855733975	2.28242E-70	*IL1R1*	-1.19592792	1.44853E-21
ABHD4	1.854968371	1.3242E-104	*HMMR*	-1.194076961	1.73157E-16
MICALL1	1.854263536	8.4766E-127	*GAS2L3*	-1.193888791	1.59404E-24
LCAT	1.852856756	1.63143E-38	*CAMKK1*	-1.187208471	1.82408E-34
INAVA	1.84740294	3.14148E-47	*PDE3B*	-1.185186027	3.6232E-27
ADGRG1	1.847369457	2.5152E-166	*KNDC1*	-1.184496154	7.64926E-13
INAFM2	1.84303216	9.0233E-104	*MEF2C*	-1.183270824	1.56384E-18
NGFR	1.84178801	1.4867E-144	*HNRNPA1*	-1.176763308	2.8138E-130
STAT4	1.841292155	7.8808E-48	*SLC6A20*	-1.173565686	1.7083E-14
WDR66	1.840637361	1.64717E-16	*PLCH1*	-1.172333161	3.70148E-18
ABCA1	1.833237179	6.25291E-54	*DPYSL3*	-1.171979662	9.92168E-38
LRP1	1.832994521	7.47697E-89	*KLHL35*	-1.164594673	1.33579E-07
ZBED2	1.821603015	6.8187E-48	*ACOXL*	-1.162954171	2.05563E-09
FHL2	1.821539142	1.9811E-266	*CADPS2*	-1.160489109	7.14028E-38
NLRP1	1.810756408	4.0497E-126	*LINC01011*	-1.158286597	2.10966E-05
RASSF6	1.807623371	5.01407E-32	*SLC7A11*	-1.157464118	2.57207E-21
PGAP1	1.805713416	1.66636E-61	*VGF*	-1.156227406	3.20978E-33
SEMA3F	1.805223862	7.06712E-89	*TMEFF1*	-1.156068777	4.99945E-05
TCEA3	1.796027633	3.45765E-23	*ACAA2*	-1.155774099	8.78359E-31
MDFI	1.790979707	1.17487E-86	*FZD4*	-1.155507316	1.35054E-29
REEP2	1.790583727	7.71075E-46	*SLC16A9*	-1.155055959	9.36099E-24

DUOX1	1.785512364	6.34134E-92	*MALL*	-1.149077615	1.0075E-130
RTL5	1.774327408	1.41439E-73	*LSS*	-1.148018815	8.12496E-65
TRPM6	1.771591334	1.3408E-15	*NR3C2*	-1.143984132	8.61911E-07
OVOL1	1.766053295	1.15682E-76	*IL18R1*	-1.142572735	6.57171E-06
RNF19B	1.757874739	6.7006E-153	*LINC00896*	-1.136463081	5.2836E-10
PMAIP1	1.75714458	2.0641E-102	*RASSF8*	-1.135487811	4.86491E-35
SMIM10L2A	1.754554215	7.23802E-39	*SOX4*	-1.133417222	4.8262E-113
POLH	1.753537433	9.4205E-207	*WDR4*	-1.130811944	7.43898E-28
SLC12A4	1.749904411	1.7497E-138	*GPR160*	-1.127351517	6.93495E-16
SARM1	1.74983864	6.91594E-17	*GCA*	-1.123509267	1.51253E-24
EPHB3	1.748111522	2.53432E-55	*PRKAR2B*	-1.122638048	7.23594E-41
RHOD	1.74740619	8.0199E-104	*NKX2-8*	-1.120824399	8.06023E-07
PLEKHF1	1.739140578	2.1516E-55	*RNASEK-C17orf49*	-1.118063429	0.008774974
FMN1	1.730741011	1.5823E-15	*FGGY*	-1.117627956	2.35861E-12
ATG9B	1.729060869	5.95302E-23	*KITLG*	-1.113266868	2.14983E-64
FAM210B	1.72827372	9.2783E-158	*DUSP9*	-1.110569182	1.28466E-12
DRAM1	1.719739655	1.8545E-138	*BHLHE41*	-1.109866991	6.01865E-13
SEMA7A	1.71882882	7.6252E-169	*ALDOC*	-1.109416575	2.07096E-44
SYK	1.718400978	2.47273E-63	*XXYLT1*	-1.108289695	6.40839E-19
AK1	1.717978096	1.3056E-111	*LRRC26*	-1.105147396	2.89883E-08
ATG16L2	1.712985579	1.2239E-106	*ZNF608*	-1.102002765	3.14808E-21
GASK1B	1.710226698	8.24225E-21	*GLRX*	-1.099566342	6.64323E-28
MAMDC4	1.708949703	1.42068E-51	*SNHG8*	-1.096759924	9.17682E-40
SLC48A1	1.708207202	3.2676E-151	*LCN12*	-1.092256274	1.54804E-05
ADAMTS7	1.70591118	2.035E-92	*KCNMB4*	-1.090194951	1.49561E-21
DDB2	1.69831903	3.6711E-183	*ARSG*	-1.087487354	1.10298E-26
BLCAP	1.69474554	1.7784E-167	*ANXA6*	-1.08561399	6.13052E-88
ZNF219	1.692227378	7.42901E-72	*PDK1*	-1.082445728	2.44752E-29
CCNE2	1.685708673	9.4449E-102	*SLC29A2*	-1.081052593	1.46217E-43
AC015802.6	1.684021601	6.10651E-13	*SLC44A1*	-1.079725403	2.04762E-67
NATD1	1.682170197	6.07153E-59	*CNTNAP3P2*	-1.079120312	2.33318E-09
NEURL1B	1.680080125	2.4642E-109	*NKAIN1*	-1.077981479	2.38218E-05
TNFAIP3	1.680024694	2.58445E-47	*TNIK*	-1.077950689	6.3033E-71
MR1	1.676704646	1.1507E-99	*DPY19L1*	-1.069198633	1.29156E-47
ALOXE3	1.672260332	1.29436E-22	*NAGLU*	-1.068251273	1.92947E-28
ZNF561-AS1	1.666740083	1.14079E-25	*FGF19*	-1.066678825	6.37414E-60
MIR31HG	1.66593595	1.99474E-12	*C2CD4C*	-1.066425125	1.4664E-15
EOMES	1.660551025	4.72171E-28	*SLC22A18AS*	-1.065681444	0.000403558
SPNS2	1.6589371	4.05014E-41	*KIAA0319*	-1.06313787	1.89381E-05
ERFE	1.657828274	1.11683E-59	*KCNIP3*	-1.062913958	7.19944E-07
AVPI1	1.655076056	3.0444E-87	*OCEL1*	-1.060458694	6.77297E-24
PHYHIP	1.653328517	5.02298E-27	*MCTP2*	-1.057414329	1.99591E-26
ANKRD33B	1.653327924	5.486E-110	*TBL1X*	-1.056987223	1.49071E-55
PARD6G	1.651335671	1.1832E-131	*AC025165.3*	-1.056905236	0.000305621
BIRC3	1.651183159	1.8958E-62	*MT1X*	-1.05645432	5.59014E-05
SMTN	1.647413212	1.1679E-129	*CLYBL*	-1.055180508	1.77446E-20
AREG	1.647319553	2.8606E-171	*FH*	-1.054348065	4.64799E-64
PPP1R14C	1.646435253	6.007E-118	*BEND6*	-1.052840035	6.26371E-08
KIAA1211L	1.64524255	4.08872E-14	*CREB3L4*	-1.051060234	2.23944E-14
INKA1	1.642935018	1.95024E-12	*LINC02331*	-1.04922543	1.92726E-06
NINJ1	1.639890843	6.38008E-71	*KLRC2*	-1.048493246	1.7E-10
PADI3	1.638556792	2.00393E-26	*SCUBE1*	-1.048261527	1.71187E-10
APOBEC3B	1.638434709	4.0051E-125	*STEAP1*	-1.045122336	8.10555E-20
SLC26A1	1.633184758	5.42078E-23	*WNT16*	-1.043811764	5.79099E-33
CSF1	1.632801404	4.34679E-51	*ENOX1*	-1.043122915	1.81849E-11
HSPA4L	1.631611005	6.9831E-131	*RIMKLA*	-1.04295525	1.46312E-19
SYNJ2	1.630548942	4.5473E-156	*ATG10*	-1.042182543	7.38623E-12
GSDME	1.624977505	5.5451E-102	*CEP112*	-1.041968728	2.41726E-21

SAXO2	1.616536201	8.23267E-11	*MRAP2*	-1.040736334	6.16854E-06
TMCC2	1.616104907	3.02344E-37	*IMPA2*	-1.038109832	3.05632E-32
ARHGAP23	1.616076217	7.7481E-113	*EMP3*	-1.037807772	2.57082E-24
CASZ1	1.613203153	3.63312E-58	*EML5*	-1.034238151	1.47615E-09
ZNF441	1.611992947	7.84147E-17	*CRYZ*	-1.032708842	2.17908E-35
INPP1	1.611225598	1.6066E-139	*LMF1*	-1.029754925	1.6065E-15
MIR34AHG	1.605815849	2.13383E-82	*SPATA24*	-1.027855693	0.000330714
SRGAP3	1.603099001	1.02336E-61	*TTC39B*	-1.023644726	4.13003E-15
PSD2	1.596108666	7.56228E-19	*DTD1*	-1.022924166	8.07238E-57
NOP14-AS1	1.595279714	1.8684E-75	*LMNB1*	-1.022031915	6.06312E-60
TRAF4	1.591337835	1.7619E-155	*OXCT1*	-1.021287444	2.31572E-38
ARVCF	1.587591522	3.86838E-94	*CALCRL*	-1.019186035	0.000718526
AC103718.1	1.584571757	3.03099E-12	*XK*	-1.018660184	5.28763E-16
SLC4A11	1.582389792	1.76775E-71	*HS3ST1*	-1.016899227	1.21418E-57
TMEM229B	1.581236764	1.35013E-19	*KLF6*	-1.014844358	8.42996E-56
LPIN3	1.577274798	2.157E-99	*PLTP*	-1.014002036	2.80802E-05
AC018629.1	1.564779386	1.34788E-14	*FGFBP1*	-1.013671809	5.62827E-09
MYEOV	1.561842403	1.8602E-140	*SLC2A4*	-1.013309916	5.90348E-05
VSIR	1.561169619	3.37205E-74	*PPP3CA*	-1.01179718	4.16094E-86
PTPRU	1.560481395	1.3578E-115	*CCDC171*	-1.011463755	3.39253E-07
CRIP2	1.553155983	6.97986E-66	*ANKMY2*	-1.010636182	5.36171E-35
TIGAR	1.550733379	4.56174E-86	*AC090015.1*	-1.010401101	4.87706E-05
RIN1	1.550068205	5.96941E-76	*LENG9*	-1.009268465	2.08257E-13
FRMD8	1.54086063	8.41883E-96	*RSRC1*	-1.008665184	2.9525E-33
NCEH1	1.538283534	2.83731E-98	*H2AZ1*	-1.007868523	2.39015E-69
AJUBA	1.538194881	2.7004E-141	*HTR7*	-1.007836444	1.41699E-36
RGS20	1.536172854	5.96283E-29	*AP006621.3*	-1.006962525	2.07534E-06
LOXL4	1.536164648	3.82329E-55	*POPDC3*	-1.005487311	7.2122E-11
GADD45A	1.535537674	3.3766E-208	*CHDH*	-1.00506633	1.22777E-31
DSC3	1.533714375	1.83402E-68	*PTMA*	-1.004979688	1.85116E-71
PI4K2A	1.533027598	1.3644E-136	*ANAPC15*	-1.004839755	3.07882E-23
LINC00680	1.524562086	2.69806E-11	*GALK2*	-1.004782704	3.14008E-16
PARP10	1.523825421	6.9645E-18	*ARL6IP6*	-1.004320955	9.80289E-44
PORCN	1.523601677	1.43106E-57	*C17orf49*	-1.003045131	2.94048E-15
TTYH3	1.523434576	4.1985E-82	*SRSF1*	-1.002081253	8.4706E-92
COBLL1	1.523154546	1.37441E-57	*SUPT3H*	-1.001706974	2.45695E-13
ARX	1.5215236	5.38263E-11	*TMEM256*	-1.000664478	2.48795E-07
LYPD5	1.520575186	3.00653E-19	*FUOM*	-1.000380948	1.33239E-09
IKBIP	1.516758372	6.4653E-115	*ATP2B1*	-1.000174123	4.54229E-51
CA2	1.512075415	1.296E-156	*PSRC1*	-0.999395605	1.11637E-18
MGLL	1.512072508	4.41853E-58	*ASPM*	-0.998195567	3.10408E-32
SMTNL2	1.509218365	4.89473E-27	*SGCE*	-0.996932424	2.31456E-30
METRNL	1.508769269	9.33996E-90	*SMIM19*	-0.996915851	1.42746E-18
DUSP11	1.506222697	6.51505E-89	*NAT14*	-0.996641788	7.42017E-24
FBLIM1	1.505989605	9.66459E-44	*MANF*	-0.996338254	8.22093E-74
CLP1	1.501738693	1.1288E-109	*TRIB2*	-0.995363509	2.86499E-35
CEP76	1.499475301	6.05131E-73	*TMEM143*	-0.990861916	3.04311E-12
EPHA2	1.499049699	5.9427E-170	*GXYLT2*	-0.989256435	7.28509E-07
PLAT	1.498198487	2.28177E-53	*ZMAT1*	-0.988888184	0.001095016
KBTBD8	1.497492118	2.77668E-20	*STX2*	-0.988250868	3.01663E-34
PLXDC1	1.49410464	1.17582E-09	*OGDHL*	-0.98704601	4.71811E-14
MIR22HG	1.493273264	2.00378E-26	*AC008443.1*	-0.986099042	0.000131669
AMOTL2	1.487745979	1.0725E-215	*SERPINB9P1*	-0.981005129	6.62971E-05
WNT7B	1.487129509	2.5184E-59	*DEPDC1*	-0.980879101	4.48242E-10
CPE	1.483255248	8.2905E-134	*DBP*	-0.980472715	2.69814E-23
RBPMS2	1.478429502	1.12663E-59	*STIMATE-MUSTN1*	-0.980355941	4.53234E-06
TGM1	1.477918568	2.38301E-13	*MRPL24*	-0.979912759	2.18698E-33
ABCB9	1.475465658	3.4257E-57	*NT5C*	-0.979217878	3.70184E-35

LINC02495	1.472909357	9.13728E-13	*ID1*	-0.978942134	1.88886E-62
TAP1	1.469825118	5.5834E-193	*CYP2U1*	-0.978916997	3.37988E-18
ANKRD29	1.467124471	4.72171E-28	*MEIS2*	-0.978886188	9.31672E-22
COL4A6	1.463451245	3.10689E-20	*BORA*	-0.977818005	6.96334E-22
STAG3	1.459870323	9.12277E-12	*SNHG21*	-0.977675972	3.18024E-05
TMEM249	1.458807511	6.38876E-20	*ZRANB3*	-0.977557963	1.51308E-13
PTCHD4	1.457307048	1.15369E-23	*MANEA*	-0.975919535	1.42774E-15
CRYBG2	1.451765792	1.74468E-18	*TMEM144*	-0.975777555	1.52469E-20
SLC46A1	1.451628407	3.77651E-78	*TXNRD3*	-0.97547143	2.94629E-25
PANK1	1.450926573	8.48912E-55	*C10orf91*	-0.974111915	0.000204638
AC005747.1	1.450632761	2.18945E-31	*PLA2G4A*	-0.973609217	4.68951E-20
NADSYN1	1.446720785	6.695E-113	*PCSK9*	-0.97311685	5.76942E-50
AADAC	1.446444092	1.48823E-13	*MILR1*	-0.972977368	4.27598E-05
TRIM8	1.44547591	8.2689E-118	*ARHGAP11B_2*	-0.972394576	0.001053012
TP53I11	1.445146512	3.7105E-108	*SLC25A10*	-0.971705305	2.22721E-33
SLC25A45	1.444425925	1.34852E-15	*PPP1R9A*	-0.970785185	1.90112E-16
KRT81	1.444178206	3.88544E-20	*IGF2BP3*	-0.9707803	6.0904E-57
AEN	1.441245552	5.0999E-134	*FAM72B*	-0.970131039	1.72293E-10
OASL	1.44105834	8.97679E-45	*NADK2*	-0.969747156	2.53585E-24
GBX2	1.437585143	3.12708E-22	*MIPEP*	-0.969559706	1.13449E-25
TRIM3	1.436813547	5.26683E-67	*AC022400.8*	-0.969430159	3.85155E-12
ACTA2	1.432495291	3.75669E-48	*ARHGAP42*	-0.967326772	2.9525E-33
AC004816.1	1.431360052	1.12899E-09	*FAM81A*	-0.967127473	3.98094E-12
RAB15	1.430305143	1.11505E-37	*MCEE*	-0.965297876	1.21675E-07
MEGF11	1.429861701	1.09069E-18	*CNTLN*	-0.964174359	1.01714E-13
MAFA	1.425476137	1.93743E-09	*AF127577.4*	-0.964006068	0.001713117
TNFRSF10D	1.424757197	7.5147E-229	*OPN3*	-0.960936779	1.28415E-09
NDRG4	1.42443011	1.48366E-59	*AC108488.2*	-0.958923835	3.51119E-05
CDC42BPG	1.423345081	6.97454E-71	*RBM3*	-0.958065095	2.45569E-70
KIF17	1.421459576	7.95216E-11	*METRN*	-0.957892775	5.82717E-24
RRM2B	1.418981708	4.54511E-78	*GNAL*	-0.954310597	7.4377E-18
CGB7	1.417111018	8.29034E-14	*MAN2A1*	-0.950856049	1.17919E-37
SLC27A2	1.415446083	4.77288E-93	*TMBIM4*	-0.94750318	3.60741E-21
IL4R	1.414786456	7.64401E-96	*CKMT1A*	-0.947077156	1.94189E-15
VDR	1.410863784	2.2206E-95	*CTH*	-0.94703517	8.01066E-14
UBR5-AS1	1.408988187	2.60493E-09	*FAAH2*	-0.946257329	1.68412E-08
CMBL	1.408517144	1.0023E-150	*ATP8B3*	-0.943328631	3.14808E-21
CCDC15	1.407420363	9.19622E-11	*HMGB2*	-0.942364177	2.17598E-38
SEMA3B	1.406942771	3.54443E-79	*MHENCR*	-0.941879145	0.00025296
TMEM158	1.404744097	5.26189E-25	*EBPL*	-0.941493806	2.85543E-40
ASTN2	1.403876737	4.18803E-55	*RPS6KA2*	-0.940433262	1.08912E-18
DUSP5	1.401862985	9.7495E-271	*NR5A2*	-0.940422448	8.33648E-11
PRKAB1	1.401042031	1.03081E-67	*ZNF581*	-0.939858052	4.75584E-23
GOLGA8N	1.399768187	1.84613E-11	*PRKAG1*	-0.939261647	1.48916E-45
IL10RA	1.398971196	9.75752E-09	*GNG7*	-0.937490713	1.54984E-22
IL1RAP	1.397576692	1.81031E-17	*FAM13A*	-0.936358011	4.01429E-18
FADS3	1.394999643	7.7184E-97	*KCNQ2*	-0.936058435	2.99849E-28
CLGN	1.39343514	3.79462E-21	*FZD2*	-0.935683029	7.70014E-26
CARNS1	1.391029236	4.23234E-08	*IGSF9*	-0.933698998	2.43671E-10
GAL3ST4	1.389716599	3.90248E-11	*AURKA*	-0.933422534	7.67531E-25
TCP11L1	1.388407498	2.43641E-48	*POGLUT3*	-0.930933508	4.04846E-39
ITGAX	1.388189823	2.94133E-15	*PDGFC*	-0.93077799	2.298E-23
ABCG2	1.38671508	8.7695E-22	*ARHGAP11A*	-0.929892421	9.45406E-33
JUN	1.386586997	1.24907E-53	*TMX4*	-0.928964349	1.08006E-21
PCDH1	1.384875421	7.66638E-81	*CAPS2*	-0.926777147	9.94521E-11
CYP4F11	1.384403062	1.95263E-82	*ARHGAP32*	-0.926282146	3.7607E-19
TNXB	1.384183353	8.64747E-39	*FRMD3*	-0.925430028	6.10575E-05
ELK3	1.383385089	2.1635E-128	*TMEM135*	-0.924587051	4.57223E-27

TLL2	1.383278485	2.3347E-13	*YPEL1*	-0.924403175	0.001381692
SNAI3	1.381691334	2.98033E-16	*PPA2*	-0.922240072	5.08839E-30
SH2B3	1.379879914	8.6782E-72	*CBLB*	-0.918188093	7.86439E-18
MYO7A	1.377081858	2.08398E-29	*AC012146.1*	-0.918027052	0.000272774
ZNF425	1.375601551	2.236E-28	*P2RX5*	-0.917742411	6.32456E-08
LAMB3	1.373933017	1.73334E-95	*TBCA*	-0.91702287	8.07389E-37
PROCR	1.373861313	2.3093E-132	*PLOD1*	-0.9164546	5.91028E-55
SPAG1	1.372853279	4.99028E-51	*LPCAT2*	-0.916219083	1.18959E-22
TMEM63B	1.371188747	6.4611E-156	*HOXA10-AS*	-0.916052575	2.56951E-13
CENPP	1.37061701	1.93721E-26	*LINC01515*	-0.91560766	1.90658E-09
PLXNB1	1.369692324	1.5859E-118	*KCNQ1*	-0.915355034	6.88178E-13
STK17A	1.368810714	4.0473E-108	*GLB1L*	-0.914954899	7.59619E-07
RALGDS	1.368337906	5.9775E-108	*ACCS*	-0.913623688	4.90073E-12
NOTCH1	1.367925169	9.72719E-69	*HSPD1*	-0.912990318	1.05378E-64
TGFA	1.365824734	1.8107E-121	*PDE9A*	-0.912342922	2.78058E-32
XPC	1.36253217	2.6997E-177	*GSTZ1*	-0.912330878	7.35326E-20
ITPKC	1.361874344	1.55079E-48	*GAL*	-0.910490797	1.58745E-20
HCN2	1.361384955	1.40688E-17	*AC007098.1*	-0.908651974	0.005622995
PCNA	1.355493927	1.3871E-258	*TCF7L2*	-0.907628847	2.017E-53
AC159540.2	1.35543903	2.55258E-19	*PRIMA1*	-0.907497229	0.000148857
HAS3	1.353381395	1.23939E-96	*SQLE*	-0.906876376	1.12948E-36
TMEM81	1.352755236	1.19839E-10	*CCNB1*	-0.906400636	9.78506E-36
CRISPLD2	1.351220922	9.09312E-39	*C19orf81*	-0.906258447	0.003697666
CES2	1.349092714	1.1172E-116	*FAM72C*	-0.905133795	2.2189E-10
DUSP7	1.348559396	1.31841E-72	*NUBPL*	-0.904847907	2.3729E-12
CASTOR2	1.34765528	2.14503E-39	*HSD17B11*	-0.903300271	9.10051E-41
TEP1	1.34706674	1.9332E-125	*OARD1*	-0.902547554	2.90446E-24
KCNB1	1.346494157	9.41239E-26	*MBOAT1*	-0.902265	1.24189E-15
AC005329.1	1.344876348	1.49862E-18	*CDON*	-0.902065629	2.01413E-21
KSR1	1.342650556	9.3032E-69	*B9D1*	-0.901873802	1.8804E-18
KDM4B	1.34161109	6.39757E-86	*CTSC*	-0.901563407	3.38932E-28
HES6	1.339286711	7.37951E-45	*C11orf74*	-0.901245384	1.36872E-08
IRF7	1.338403503	4.06372E-34	*MFSD3*	-0.900902801	6.05768E-18
SGK1	1.337921715	3.83139E-14	*TMEM258*	-0.900729913	4.12843E-08
ZFYVE1	1.337203059	4.62213E-59	*CA8*	-0.90052045	1.45336E-05
RNF144B	1.332468846	7.30398E-15	*HMGB1P6*	-0.900286669	7.33916E-05
INTS6L	1.331635152	5.11474E-33	*PYCR1*	-0.900138167	1.3527E-60
TRIAP1	1.331520193	4.67698E-85	*INPP4B*	-0.897468886	4.93942E-37
AC027290.3	1.323836662	3.42141E-12	*RPL3*	-0.89732288	3.5117E-117
HTR1D	1.3217508	1.27987E-26	*MAML2*	-0.89648742	2.18176E-19
RGS16	1.321449409	4.47503E-25	*EPHX2*	-0.896098717	9.84548E-26
KLK7	1.319675626	1.96052E-77	*TCEA2*	-0.893397659	1.86143E-19
FAM214A	1.319273305	5.26025E-32	*MYDGF*	-0.892848652	3.36499E-59
TMCC3	1.312070139	5.97391E-62	*CENPE*	-0.892357805	5.67658E-06
CFAP74	1.31159239	5.58085E-12	*CNTNAP3B*	-0.890997447	2.31963E-37
LRP10	1.308529616	3.82201E-89	*ZNF214*	-0.890548919	0.004342251
LAMC3	1.307670741	1.02325E-38	*MRPS30-DT*	-0.890500047	0.000361954
S100A2	1.304702983	2.36962E-24	*HSP90B1*	-0.889992549	1.45399E-70
CSRNP1	1.304460949	8.4084E-78	*AC046134.2*	-0.88993537	0.000194451
THAP10	1.303716371	1.77762E-14	*NPW*	-0.889603249	3.06521E-10
AGRN	1.302844696	3.33537E-80	*OR51B4*	-0.889427907	4.63695E-10
ACSL6	1.302655224	1.3414E-12	*LSM6*	-0.888311249	3.87256E-20
CASTOR3	1.302617527	8.93962E-32	*PTPRG*	-0.888184963	2.92322E-30
NBPF10	1.301983001	1.81908E-52	*SLC35D2*	-0.887898633	8.32725E-32
CRACR2B	1.301374254	2.07495E-34	*ADAM11*	-0.887596132	4.43356E-22
NABP1	1.301263918	8.767E-44	*TRIM52-AS1*	-0.886961681	2.96683E-07
TNFRSF10B	1.291251812	1.326E-232	*NME4*	-0.886841541	1.38571E-22
MXD1	1.290477572	1.23582E-28	*HECW2*	-0.886613961	0.001721448

PIGCP1	1.289980909	4.78789E-08	*CPT1C*	-0.886502689	2.40056E-08
CALCOCO1	1.288598838	6.47394E-72	*STARD13*	-0.886319622	2.18952E-09
AC092683.1	1.288336931	4.81385E-11	*TMTC2*	-0.88625618	7.28944E-25
TNFRSF10A	1.287863799	4.0114E-132	*STT3B*	-0.885404263	1.84952E-57
AC016831.6	1.287311259	1.01745E-16	*RNF145*	-0.885389548	1.00637E-54
PRF1	1.287040179	2.35886E-16	*TTC21B*	-0.884387828	2.03672E-13
KCTD11	1.282574162	3.25725E-38	*LFNG*	-0.883476041	1.93992E-10
ZNF561	1.282099387	2.19391E-67	*MAPK4*	-0.882913325	0.004671242
FLNC	1.280972643	4.52923E-96	*SCART1*	-0.881747525	3.21777E-05
EMC3-AS1	1.279313086	2.13623E-08	*ALKBH7*	-0.881512134	6.30069E-15
LRATD2	1.278326443	2.82852E-83	*POLR3G*	-0.878334291	1.15795E-22
APLP1	1.277167031	3.60665E-65	*LINC00460*	-0.878270771	0.0003711
FRMD4A	1.275522958	9.55403E-11	*FAM3C*	-0.877281069	1.82959E-29
GPR153	1.273840716	1.9882E-28	*CGREF1*	-0.876999643	5.92541E-07
CHST6	1.273687392	2.6789E-99	*HGH1*	-0.876971872	6.53056E-15
SLC2A12	1.272701876	7.55656E-42	*TRIM2*	-0.87627191	2.43579E-27
AC112220.2	1.272450834	4.24131E-12	*DUT*	-0.875800568	7.89592E-35
FGD6	1.272130805	4.63044E-57	*FXR1*	-0.875773044	3.83428E-67
LINC02405	1.272055883	1.74131E-06	*AL392172.1*	-0.874072755	0.010904113
RPS27L	1.270426785	6.78699E-59	*CRYL1*	-0.872890499	5.99252E-26
LATS2	1.269518206	1.09666E-58	*AUH*	-0.872034062	5.23705E-22
LINC01405	1.268600583	5.98367E-09	*PDE5A*	-0.871130176	1.27164E-12
LMO7	1.266915754	1.16412E-31	*LPAR1*	-0.870916611	5.15698E-25
LHX5	1.266867001	1.43367E-24	*LRRC27*	-0.870302667	4.80146E-06
AL161891.1	1.266301542	9.09868E-08	*AL139300.1*	-0.870285234	0.006329028
FOSL2	1.265579054	4.82323E-69	*RPL32P29*	-0.869603356	0.047887984
NOCT	1.264717352	3.10666E-44	*CAMK4*	-0.868742545	1.69397E-15
FLYWCH1	1.26341337	7.73542E-75	*MCM6*	-0.868260714	1.33092E-53
DUSP8	1.261119053	5.42985E-31	*NIPSNAP2*	-0.868104737	4.83431E-48
ABCB6	1.260178396	1.47491E-76	*MAOA*	-0.867964751	8.14255E-15
TAF3	1.258315091	9.03594E-75	*IL17RD*	-0.86762256	9.39799E-31
AC004263.2	1.257141109	5.74437E-08	*TBC1D16*	-0.866980907	3.70492E-32
IQCJ-SCHIP1	1.256167561	2.55502E-34	*WWOX*	-0.866948007	0.000272393
DNAJC18	1.254070413	1.17687E-29	*GPR180*	-0.866337189	5.0948E-26
DUOX2	1.251284209	4.55361E-07	*HNRNPA2B1*	-0.866232465	1.18193E-40
NTPCR	1.249209881	3.4615E-86	*FAT4*	-0.865606208	0.000417301
NTN1	1.248851342	4.73214E-34	*KIAA0825*	-0.865394495	0.002928008
MATN1-AS1	1.245098452	2.78977E-08	*CHN1*	-0.864678662	6.25878E-05
COL9A3	1.242550748	9.37309E-49	*ZBED3-AS1*	-0.864140506	0.00806745
PEAR1	1.242112029	5.2129E-58	*TMSB4X*	-0.863883844	4.68091E-25
BRICD5	1.242053039	1.36059E-12	*PIGBOS1*	-0.863793022	1.16613E-07
MAP3K12	1.24033695	2.85208E-78	*CPS1*	-0.861735531	2.82013E-37
DGCR11	1.239832301	6.26484E-07	*VSNL1*	-0.859975716	2.92452E-19
CEP85L	1.239378239	4.98925E-22	*SUCLG2*	-0.859707513	5.41094E-43
HHAT	1.239206736	9.25054E-29	*AC024940.2*	-0.859148649	0.000753404
TSPAN14	1.238821362	2.4257E-147	*NRM*	-0.858551406	9.8344E-10
NOTCH3	1.238107718	5.54909E-45	*BMP6*	-0.857313614	0.001016369
ARAP1	1.237235775	2.27186E-90	*DICER1-AS1*	-0.856832481	4.32127E-06
SEPTIN4	1.235022434	4.7085E-23	*NSMCE2*	-0.855827368	2.49577E-13
FP236383.3	1.234672182	8.39693E-11	*ANO5*	-0.854133816	2.63939E-06
SLC2A1	1.231279831	1.03919E-92	*BX088651.4*	-0.853673765	0.000633267
FOSB	1.230196625	4.26485E-15	*EDA*	-0.853419623	5.21797E-11
TNFAIP1	1.229892133	1.7386E-117	*AGO4*	-0.853100591	2.58764E-14
GABRD	1.229374192	1.18943E-34	*HMGB1P5*	-0.852855069	6.79779E-12
MMP14	1.228778204	3.01577E-88	*GRAMD1B*	-0.851088516	2.32704E-09
CDK5R2	1.228104356	9.95835E-09	*CCNB2*	-0.850719195	4.97341E-23
ERAP2	1.227938302	5.56846E-12	*ITPRID2*	-0.850213967	8.60507E-48
MYO6	1.227800586	2.27257E-70	*PAPLN*	-0.849454599	1.48151E-12

SLC37A1	1.227190109	3.25633E-42	*CAMK2D*	-0.849164366	1.04846E-31
SYNE1	1.225715883	7.352E-22	*HOOK1*	-0.848692902	1.30257E-24
COQ8A	1.225558803	1.26988E-97	*SEM1*	-0.848088745	2.25149E-17
THBS1	1.223824595	2.7496E-78	*SNHG6*	-0.847534498	2.41254E-26
HROB	1.223709595	2.75509E-33	*GALNT4*	-0.847528883	1.77676E-06
MAP3K9	1.220535487	2.68005E-64	*TRIB1*	-0.847369323	1.76429E-58
HMOX1	1.219981639	4.97912E-37	*POLA1*	-0.84629231	7.72999E-33
CCDC187	1.219117299	6.04451E-13	*AC073508.2*	-0.846237461	3.94105E-05
BTBD10	1.218750445	1.53696E-79	*SATB2-AS1*	-0.846019909	0.002217819
SLC44A5	1.218484577	2.60556E-17	*ZBTB14*	-0.845881481	1.03854E-14
FBXL18	1.215830721	3.3419E-62	*CDPF1*	-0.845138239	1.36329E-10
KRT80	1.213180653	4.75371E-43	*USP13*	-0.844952485	5.68928E-33
ABHD8	1.212965121	1.01403E-19	*MGAT2*	-0.844623106	1.34619E-38
DNAJB5	1.212062909	5.94074E-25	*CCSER1*	-0.844604149	0.000830697
MYLK	1.211258312	8.03948E-34	*NAT16*	-0.843397801	0.001292964
CYP4F2	1.210555584	1.19091E-05	*LRRC6*	-0.842668125	6.39059E-05
BTG3	1.20965219	1.26248E-97	*GGACT*	-0.841496131	0.00013175
MAPK13	1.208527458	1.1558E-87	*SEC61G*	-0.841132257	3.15089E-17
IQCN	1.204574717	5.88766E-10	*TSC22D3*	-0.840860927	7.56148E-16
LINC02323	1.203582925	1.94576E-05	*DLGAP1-AS1*	-0.840702581	0.001763973
RIOK3	1.201850643	2.57439E-66	*BCL2L12*	-0.840320346	7.94518E-16
SIRT2	1.201819227	2.77893E-47	*C1QL4*	-0.839914151	1.26577E-11
SERINC2	1.201332179	1.70333E-87	*C5orf30*	-0.839405084	3.52226E-16
ZGLP1	1.201126243	3.27167E-06	*AC083799.1*	-0.838811193	0.000155799
FBXW7	1.193333693	9.24972E-48	*VKORC1*	-0.837668486	1.91962E-37
GREB1	1.193087305	2.6273E-16	*CCDC121*	-0.837633086	4.77483E-06
SEMA3G	1.192429156	1.10428E-24	*SEC24D*	-0.836616245	1.00169E-37
PTGES	1.191111886	6.63245E-73	*LRRC61*	-0.835979719	8.51486E-09
ZNF440	1.189951873	4.4404E-35	*ZNF32*	-0.834596935	1.9595E-08
ANGPTL4	1.188994879	2.40056E-08	*LINC01806*	-0.834044874	2.87557E-07
AGAP3	1.18823723	7.78374E-67	*HSPA5*	-0.833898693	7.5214E-138
AC027601.2	1.186653591	1.01226E-05	*CALB2*	-0.8336582	1.52309E-16
MARCHF3	1.186318402	3.28157E-08	*ATP5ME*	-0.833647364	0.015280433
TRIM35	1.184254753	3.12051E-37	*DPM3*	-0.833618168	1.79317E-16
SMAD7	1.184003976	7.51901E-13	*AXIN2*	-0.832880461	5.4727E-12
TRPV3	1.183606737	9.99179E-09	*SORD*	-0.832605183	1.33931E-69
GRAMD2A	1.183424569	5.62144E-42	*C20orf27*	-0.832400087	7.76046E-27
ZNF337	1.183153763	4.38058E-46	*SLC27A6*	-0.832299952	2.67214E-18
AC108676.1	1.182886952	5.1231E-09	*KBTBD3*	-0.831627299	6.92241E-07
ASB16-AS1	1.181861811	4.59738E-25	*TBC1D32*	-0.831325575	2.18954E-08
BBC3	1.181727073	5.52211E-38	*ZNF280D*	-0.831171127	1.56909E-24
ULK1	1.180486987	8.92748E-58	*TMEM198B*	-0.83005775	4.88871E-11
AMIGO2	1.180039703	3.59348E-09	*FAM83E*	-0.830023606	0.00170643
TRIM54	1.179272327	1.55374E-08	*MTMR11*	-0.829370868	6.69928E-16
IQANK1	1.179250088	6.80746E-47	*CPB2-AS1*	-0.829083397	0.003728871
ICOSLG	1.178667082	3.37395E-26	*MCF2L*	-0.828393674	2.23656E-16
SLC30A1	1.177565875	1.66781E-60	*TBCD*	-0.827634652	2.97344E-50
FAM83H	1.176176524	2.73013E-58	*GALNT12*	-0.827323765	2.7762E-39
SAMD4A	1.175743614	2.03424E-37	*PIK3R1*	-0.827292817	1.36891E-17
MOSPD1	1.17549384	5.12858E-71	*PSIP1*	-0.827249011	1.74392E-31
POLN	1.175069157	9.26417E-05	*ADCK1*	-0.825798127	2.41897E-07
DPF1	1.173367211	2.42115E-23	*H2AW*	-0.825406151	1.58379E-07
APAF1	1.170119006	6.20777E-52	*THG1L*	-0.825385426	5.37766E-31
HSPG2	1.169733958	1.31265E-18	*GSTM4*	-0.825213971	1.87869E-15
ITGB4	1.168866547	4.16865E-84	*FBXO17*	-0.824535592	1.08758E-13
RDH10	1.168532235	8.64167E-69	*DHCR24*	-0.823938714	8.74683E-48
AC022137.3	1.168131216	4.55401E-06	*SPA17*	-0.8227456	1.73705E-10
NECTIN1	1.167946506	1.0013E-47	*RUNDC3B*	-0.821460319	1.4073E-05

KIF26B	1.167262231	4.3024E-09	*P4HA1*	-0.821432357	8.63763E-30
DCLK1	1.165911855	1.81635E-26	*SLC66A3*	-0.821118323	3.23151E-24
ZNF30	1.162150553	4.58877E-09	*TNNT1*	-0.820691532	1.21134E-35
FP565260.3	1.16181434	6.49305E-18	*DEPTOR*	-0.819675022	1.35735E-08
USP18	1.158825174	1.95915E-26	*SELL*	-0.819662011	0.001218191
HSPA12A	1.158787705	5.45135E-93	*RPUSD4*	-0.819094948	3.09949E-35
BLOC1S2	1.158323758	4.77128E-70	*SRR*	-0.818819608	2.33582E-08
NOL4L	1.156252857	2.14735E-29	*DNAAF5*	-0.818520119	8.0734E-36
HOXC10	1.154290674	1.83247E-14	*GVQW3*	-0.818372268	2.45537E-07
ADGRB2	1.1541774	2.39962E-39	*BMP4*	-0.818328404	6.11155E-41
CACNA1A	1.15388978	1.84477E-16	*PDGFRL*	-0.817883285	1.82193E-13
CORO1A	1.153595009	2.79194E-11	*DTWD2*	-0.81778648	8.74724E-12
TRAK1	1.152723	6.7914E-111	*GCSH*	-0.816923437	1.44175E-32
TGFBRAP1	1.150150345	1.72731E-87	*WASF3*	-0.815740356	1.11022E-06
KIAA1614	1.147265771	6.2699E-09	*PTPRN2*	-0.815667995	7.07887E-05
AL117339.5	1.147118227	5.86626E-07	*ALG14*	-0.815630641	4.6259E-07
DNASE1	1.143339838	8.95574E-25	*TTLL1*	-0.814978968	6.28801E-06
BMP7	1.140112596	2.14633E-37	*C3orf18*	-0.814622581	1.25876E-11
NR4A1	1.139717996	2.35187E-33	*LINC02361*	-0.814564914	0.006453184
SH3BP5-AS1	1.138680489	1.2884E-10	*SRSF2*	-0.814546314	5.22807E-79
LRP2BP	1.138584203	8.35624E-11	*PPP1R3E*	-0.813944456	9.57887E-05
DDIAS	1.137302831	4.11303E-44	*IDH1*	-0.813488748	3.14924E-39
TINAGL1	1.136738791	1.11122E-68	*LINC00623*	-0.813250013	2.24508E-14
CEP170B	1.135647356	2.14696E-62	*B3GALT4*	-0.813035898	0.010216741
KAT2B	1.134602584	1.89945E-25	*EXOC4*	-0.812436232	2.29875E-30
ALPK3	1.1338066	1.06767E-12	*SNHG7*	-0.811128739	1.73264E-35
PPP1R13L	1.132927629	1.77159E-62	*DPH5*	-0.810713295	7.13993E-24
PHLDB3	1.132827117	4.22955E-20	*KCNC3*	-0.810608515	1.04372E-07
MERTK	1.131551357	3.20959E-27	*PPIH*	-0.810478722	2.06388E-26
NKD2	1.130921732	3.41312E-29	*PMP22*	-0.810302976	2.84437E-29
UNC5B	1.130462553	1.89123E-55	*SPRY2*	-0.810301114	2.49331E-12
BAG3	1.129363075	2.38636E-81	*STK39*	-0.809136286	4.81551E-68
CCND1	1.128206982	5.2108E-151	*PTBP2*	-0.806873329	8.0647E-11
BAX	1.125183828	6.9698E-43	*SOCS6*	-0.80575212	3.58724E-23
PLXNA2	1.12433286	5.449E-08	*CASC2*	-0.804705941	0.000177436
ZNFX1	1.122611604	1.51458E-84	*TMCO3*	-0.804609958	5.66581E-40
GPR3	1.12091587	1.7503E-10	*AC027228.2*	-0.804343574	6.10947E-05
FCHSD2	1.12074967	2.51081E-56	*H6PD*	-0.803593432	1.47337E-21
CCND3	1.118674921	2.03779E-57	*ANKRD36C*	-0.803247209	3.30274E-07
AL391684.1	1.116602577	9.02996E-06	*SLC11A2*	-0.802960678	8.97253E-29
RBMS2	1.115873618	4.66842E-76	*MCUB*	-0.801822336	6.28932E-17
FBXO48	1.114039684	2.22332E-08	*SEPTIN10*	-0.801387472	1.80143E-35
GBE1	1.113153746	2.1953E-58	*DBI*	-0.800778667	2.05963E-31
RELT	1.112293124	9.91998E-41	*SMAD6*	-0.800438497	1.79915E-11
LRRC37A4P	1.111548599	9.48982E-45	*PAIP2B*	-0.80040401	5.85698E-19
RAD54L	1.111253266	3.69998E-41	*AL590560.2*	-0.798092978	1.20926E-06
ISCU	1.108980573	9.96681E-89	*NFE2*	-0.797285603	0.001778921
PARD6B	1.108611418	2.58677E-27	*SND1*	-0.79681586	1.03769E-71
GIT1	1.107200146	2.18456E-67	*TMCO6*	-0.796231183	1.92752E-13
SRRM2-AS1	1.106172935	9.11556E-06	*PTMS*	-0.796218732	6.36225E-11
AMOTL1	1.104369322	1.21955E-87	*TC2N*	-0.794804694	4.54345E-14
ATXN3	1.104314195	4.44898E-39	*GSEC*	-0.794304179	1.70123E-05
ZBTB5	1.103092135	5.76525E-69	*CDC25B*	-0.793188347	1.21904E-37
TNFRSF10A-AS1	1.100834683	8.54908E-08	*RNF130*	-0.791986031	1.5054E-32
TMEM51-AS1	1.100437133	1.39791E-12	*HMGN3*	-0.79163249	9.58246E-20
IFFO2	1.09871489	2.29085E-92	*ANK2*	-0.790977138	0.001742111
FBXO22	1.097739478	4.66424E-85	*CTDSP1*	-0.790941355	6.37304E-35
GCH1	1.096932699	1.03156E-59	*ERP29*	-0.79075288	1.72345E-36

NBPF14	1.096437518	1.78134E-53	*CD24*	-0.790340769	6.51344E-15
LINC02454	1.095135472	1.40925E-05	*SLC25A24*	-0.789972863	6.59426E-24
TAB3	1.094962665	2.44221E-62	*PJVK*	-0.789826932	0.001448035
AL731571.1	1.094838964	1.70142E-09	*BBS10*	-0.788909236	9.51305E-21
SLC38A3	1.094657733	1.87798E-09	*MT1E*	-0.788641786	3.95387E-05
PRKAB2	1.09458957	5.50233E-54	*EREG*	-0.788475615	2.47881E-27
VWCE	1.094399088	5.02216E-06	*RAB24*	-0.788454635	1.22032E-11
FAM83F	1.094272192	8.58925E-65	*DGKE*	-0.787870274	2.12068E-07
GPX1	1.094185317	2.33367E-70	*CORIN*	-0.787505742	0.000385118
LGALS9	1.092536767	1.62E-06	*ELOVL6*	-0.787492643	9.96473E-36
ELOVL3	1.089772211	3.49331E-20	*TBC1D19*	-0.786079286	3.07269E-06
ANKRD24	1.089178332	3.96193E-16	*PCDHAC1*	-0.785763728	0.004702353
PIM2	1.088957342	1.51995E-49	*CKS2*	-0.785452059	1.72156E-30
SIDT2	1.088531892	1.95663E-53	*ALDH6A1*	-0.785257621	2.75465E-18
SRA1	1.088004123	3.95594E-64	*RPL12*	-0.784800043	1.12303E-42
AL928654.4	1.087645215	6.47143E-12	*CWC27*	-0.784570185	3.74782E-22
AC020763.4	1.087121405	6.16087E-05	*MTR*	-0.784367445	7.41108E-31
AC007566.1	1.086952318	1.73737E-05	*ODF3B*	-0.783068937	0.000720871
RABGGTA	1.086696198	1.04463E-31	*PCDHAC2*	-0.782847716	3.04757E-11
SLX1B-SULT1A4	1.084500874	1.04867E-20	*TDRD3*	-0.781796556	1.11899E-16
PPP2R2D	1.083246764	1.77345E-46	*FRMD4B*	-0.781721805	0.001522872
SOGA1	1.082048444	2.62796E-38	*HNRNPH3*	-0.781234024	2.74611E-32
RIBC2	1.080461164	3.62947E-08	*LAMTOR2*	-0.780736303	1.0122E-12
RUSC2	1.080324047	1.97257E-39	*FOXP1*	-0.780288209	4.17889E-07
RRM2	1.079798036	2.31165E-57	*BPHL*	-0.779748074	5.12654E-16
CLN8	1.07871301	1.91217E-65	*PROM1*	-0.779226177	8.52792E-24
GAS6	1.078434411	1.22372E-64	*ANP32E*	-0.77918727	7.04842E-28
SERF1A	1.078424747	0.025377945	*ACE*	-0.778221419	3.52585E-12
CYTH4	1.077753638	2.18191E-10	*HOXA13*	-0.777336651	1.88981E-10
SHISA8	1.076685425	4.19937E-09	*CGNL1*	-0.777026232	2.3155E-06
DOK7	1.076446426	3.77692E-09	*LEPR*	-0.776719624	1.30648E-13
ALOX12B	1.076151149	9.84348E-05	*NOXO1*	-0.776382772	0.000935111
AC007192.1	1.071471866	0.000854027	*FZD7*	-0.775230064	1.66829E-15
WRAP73	1.071207306	1.5018E-67	*CDKN2A*	-0.774998766	1.02805E-17
EPAS1	1.071109277	2.82151E-86	*PIGP*	-0.774527361	8.22879E-12
TMTC3	1.070663301	7.80854E-40	*PLEKHJ1*	-0.773727568	4.42407E-33
PHPT1	1.069532178	9.49084E-43	*HMGB1*	-0.773537358	2.83218E-35
BCL10	1.067019047	6.64559E-46	*CHSY3*	-0.772989872	3.33259E-05
OVGP1	1.066744337	1.53709E-08	*TGFBR3L*	-0.772543693	8.42693E-05
IRGQ	1.064884336	4.40668E-39	*CCDC88A*	-0.772436388	7.20532E-17
LZTS1	1.064644366	1.94346E-25	*EFNA2*	-0.771594574	6.51344E-15
NPR2	1.062850748	3.86076E-16	*ERRFI1*	-0.771426296	3.44871E-50
FAM98C	1.061945598	2.89985E-36	*TIMM13*	-0.770668389	1.02905E-17
STX6	1.061937431	4.79666E-91	*NFIL3*	-0.770265715	5.15884E-21
CYP2S1	1.06179998	3.78255E-43	*NPM1*	-0.770026551	2.29E-44
NIPAL3	1.061616139	6.42938E-71	*GPR39*	-0.769571758	3.86357E-06
ENC1	1.061595286	4.31679E-71	*SDF2L1*	-0.769422699	1.42817E-16
RAP2B	1.059505489	3.8709E-104	*SGO2*	-0.768482519	2.24308E-13
PI4KAP1	1.058368469	7.42951E-28	*IFT52*	-0.768325309	1.12283E-21
FOXF1	1.057803946	1.74229E-11	*DNPH1*	-0.768320882	1.34297E-18
LINC00294	1.056013997	1.30506E-25	*GULP1*	-0.767901279	1.34961E-15
BEST1	1.055082678	5.67903E-05	*SLC22A31*	-0.76674941	1.89032E-16
HEG1	1.05430411	2.4338E-23	*TMSB10*	-0.76635106	5.99868E-07
CYP1B1	1.052246872	8.80986E-07	*PTDSS1*	-0.765711999	1.64112E-55
NIPAL1	1.051542867	3.62405E-33	*SERPINF2*	-0.764080526	3.32196E-07
YBX3	1.049821284	9.7163E-119	*NEK3*	-0.763854564	1.44541E-10
GPR137C	1.04974851	1.02885E-06	*EDRF1-DT*	-0.76364123	0.004374171
CCDC3	1.04884348	7.20645E-25	*CNTNAP3*	-0.763603616	9.59216E-19

KLK10	1.048600222	2.6536E-29	*GPR63*	-0.762674869	1.19992E-09
AC129492.1	1.048242111	1.30648E-13	*SAP30*	-0.762459175	4.37705E-08
MYO1E	1.04817041	4.55744E-64	*ZC3H6*	-0.761173386	6.70502E-09
FCGBP	1.048139986	5.76893E-16	*CBX3*	-0.761058695	3.84357E-28
ENHO	1.048076109	7.22303E-09	*MIR924HG*	-0.760460085	0.002994214
CASKIN1	1.047612382	1.52904E-17	*UBE3D*	-0.760163073	1.27628E-06
DEGS2	1.047574183	0.000142271	*SLC10A7*	-0.759571007	3.51534E-09
MAGEA2B	1.0451159	4.83856E-05	*LRRC20*	-0.759294412	2.79204E-17
BRMS1	1.044793127	6.73619E-49	*HACD1*	-0.759280957	6.10879E-10
LTB4R	1.04445483	1.09715E-12	*PTPA*	-0.759243377	1.02341E-42
CABP1	1.043918686	0.000188586	*SLC1A1*	-0.758285252	9.95159E-13
CDR2L	1.043500286	3.24676E-41	*STAMBPL1*	-0.7580231	1.41435E-08
AP000347.1	1.043051501	7.91538E-07	*PRSS12*	-0.756562049	2.0226E-32
MICAL2	1.042855736	3.29187E-42	*PDIA5*	-0.756103683	7.00694E-36
PIK3R3	1.042754981	1.09665E-13	*CACYBP*	-0.755830138	9.15956E-41
CORO2B	1.041795924	1.3006E-06	*BAHCC1*	-0.755175612	2.11727E-18
PLCD3	1.041773356	8.76513E-60	*AC092279.1*	-0.754911838	0.000542638
COL9A2	1.041530304	2.40985E-15	*SRSF3*	-0.754601534	7.17347E-52
ABHD14A-ACY1	1.039363914	3.32246E-08	*PCK2*	-0.754364028	9.094E-14
MFSD4A	1.037953309	4.51875E-07	*INVS*	-0.754329279	3.43083E-16
AIFM2	1.036950923	3.50167E-54	*IL15*	-0.754274868	0.000176335
AL162231.2	1.036767859	1.43417E-11	*CETN3*	-0.754126155	1.55151E-18
ACP7	1.036225848	7.39745E-09	*CDH15*	-0.754059253	1.44982E-06
KANK3	1.035996027	7.79633E-30	*KSR2*	-0.753101598	9.42745E-05
PRDM16-DT	1.035760266	0.000110695	*CPOX*	-0.752819849	5.02924E-45
SLC1A4	1.035463342	3.21474E-19	*EXTL2*	-0.752641371	9.31811E-20
CSNK1G1	1.034616548	1.53744E-59	*GNE*	-0.752145765	7.7577E-24
AC040160.2	1.033501239	0.000113757	*CXXC4*	-0.751312217	0.000918215
PML	1.03349788	9.48883E-49	*TSNAXIP1*	-0.751007142	0.016971055
PLD6	1.033468015	1.32997E-37	*QPCTL*	-0.750693681	4.68167E-18
MTO1	1.033114435	1.13857E-55	*DPYD*	-0.75065072	0.001120329
SORBS1	1.032109822	2.4654E-21	*LTBP1*	-0.750552019	7.61766E-11
LIMK2	1.031722819	2.30458E-46	*ATP5MC2*	-0.750436141	1.04456E-16
TTC9	1.030482726	2.12418E-20	*SLC2A13*	-0.750072794	8.13365E-12
WNT4	1.029891143	9.42835E-06	*CNTRL*	-0.749776913	3.4515E-12
GLTP	1.026693663	4.8628E-64	*CA5B*	-0.749770212	2.37293E-05
CORO6	1.023702864	3.83696E-29	*AC107027.3*	-0.749760863	0.000128799
SHANK3	1.023023107	1.61946E-31	*FARS2*	-0.749392421	9.51978E-09
CHAC1	1.0223273	1.71008E-09	*NDUFAF3*	-0.748840313	2.57718E-18
SOCS4	1.022210465	9.75561E-37	*SNHG30*	-0.748298289	0.000136462
BRMS1L	1.021995564	1.99605E-18	*ZNF385B*	-0.747429512	7.10782E-10
FYN	1.020873624	9.4989E-25	*ST6GALNAC6*	-0.747379401	2.38875E-11
E2F8	1.019114867	7.91181E-36	*CD320*	-0.747196562	2.77853E-21
AC005674.2	1.018837843	1.71514E-11	*HSP90AB1*	-0.746483039	3.59866E-80
ANO9	1.01871646	3.4116E-41	*TMEM147-AS1*	-0.746310603	8.44758E-15
ZNF654	1.018025817	4.22234E-28	*COX18*	-0.745624816	5.4538E-13
MNX1	1.018025717	2.74581E-12	*PET100*	-0.744921178	0.014250502
RAD51C	1.015351284	5.11868E-46	*GOLM1*	-0.744810243	2.30772E-58
AC092821.2	1.015228264	4.87601E-05	*FPGT*	-0.74420533	1.42048E-09
KCNJ12	1.011619137	1.51002E-32	*ZFP30*	-0.743935648	2.77426E-06
ZHX3	1.011326181	4.34305E-41	*GLG1*	-0.743602477	5.91392E-42
AC015712.2	1.009261718	7.20476E-39	*MTAP*	-0.742152306	2.57268E-35
PNP	1.006607085	2.26338E-76	*PCSK6*	-0.741690386	6.69025E-14
ALDH4A1	1.006467919	2.53967E-49	*DEPDC1B*	-0.741458932	7.72702E-13
MAST3	1.006332766	2.01714E-34	*G6PC3*	-0.741301268	3.63889E-18
CACNA1G	1.005850637	2.72354E-13	*BCL3*	-0.741093965	1.94409E-15
AP4E1	1.00578871	3.49102E-30	*LONRF1*	-0.740719862	1.24407E-17
TUFT1	1.005691312	1.69433E-28	*AP000866.1*	-0.740367851	0.012250885

INHBB	1.005063008	0.000243164	*GLCE*	-0.740239552	3.0773E-23
ZFP90	1.004142368	5.06559E-42	*GALNT14*	-0.738769716	5.99074E-12
IER5	1.00394716	1.84846E-66	*NEBL*	-0.738638797	2.82802E-16
MAGEB17	1.003765626	0.000195769	*MRPL48*	-0.737778554	1.25577E-17
CLCF1	1.003606968	1.80327E-13	*PHF14*	-0.737711431	8.65954E-06
CBR3	1.003530172	3.3967E-05	*ARMCX5-GPRASP2_1*	-0.737668534	0.000258708
SNX21	1.003310291	1.51206E-23	*CCDC85B*	-0.737333276	1.79374E-20
ZDHHC8P1	1.002537111	6.95057E-06	*CU633906.7*	-0.737156806	0.005437025
CPEB4	1.002343836	9.77593E-33	*NPM3*	-0.737117111	3.90465E-24
ASS1	1.001791536	9.68556E-63	*AC108860.2*	-0.736469052	0.00658716
AL133325.3	0.999874395	1.33477E-15	*ZNF438*	-0.736458393	8.95107E-05
AXL	0.999097701	2.75313E-96	*LMAN2L*	-0.735243548	5.08758E-18
MGRN1	0.998878602	2.43448E-34	*FKBP5*	-0.734938419	1.34167E-32
MANCR	0.998634563	1.42207E-05	*C2CD5*	-0.734592468	1.04997E-18
RELB	0.99813241	1.6266E-25	*ARHGEF9*	-0.73453221	6.59164E-20
STAT3	0.997428004	1.21581E-81	*ADAM22*	-0.734035411	2.23712E-15
SLC35E4	0.997285368	8.11838E-18	*COMMD10*	-0.733140673	2.10095E-13
NDST1	0.997275473	3.04157E-61	*OSTC*	-0.732941262	5.30112E-27
FAM95B1	0.996185555	3.36009E-08	*TESC*	-0.732737087	2.06867E-16
RBM38	0.995864098	1.03936E-55	*PIGN*	-0.732372082	3.45509E-11
MEGF6	0.993957891	1.93401E-26	*TMTC4*	-0.732111265	2.82155E-15
PISD	0.993854067	2.15918E-43	*TMED7-TICAM2*	-0.731853082	4.79882E-13
PI4KAP2	0.992040683	2.70784E-36	*AC132872.5*	-0.730774072	0.000651066
ELL	0.992011146	1.52229E-44	*LIX1L*	-0.73022861	2.8415E-26
RFTN1	0.991984366	1.16738E-12	*AC006230.1*	-0.730058783	0.000218643
NBPF26	0.989180611	9.1719E-35	*LETMD1*	-0.72985579	7.352E-22
FBF1	0.987977914	2.2341E-28	*DHCR7*	-0.729619395	2.51005E-21
SLX1A-SULT1A3	0.987091157	2.5481E-43	*SYT1*	-0.729246576	7.12175E-19
GAB2	0.987053209	5.06033E-11	*PPIB*	-0.729205655	1.39532E-51
CLIP1	0.986566061	4.20583E-80	*DRAM2*	-0.72862674	6.88987E-21
BICDL1	0.986327673	8.31317E-20	*ZC2HC1A*	-0.728541105	7.96816E-12
ATP6V1G2-DDX39B	0.984184284	2.77584E-10	*ADM5*	-0.728118765	5.42158E-07
FOXD1	0.983999732	1.03013E-22	*EEF1AKMT2*	-0.728080875	1.15145E-17
SYTL1	0.980861466	1.08957E-37	*ANP32A*	-0.727201214	4.7571E-58
FBXO32	0.979722084	1.71318E-08	*TSPAN15*	-0.726980982	1.88292E-16
PIK3AP1	0.979282677	2.3149E-27	*SLC38A6*	-0.726924812	0.003995105
MLPH	0.976439263	2.55599E-50	*TUBB*	-0.726616155	4.43497E-25
DGAT1	0.975861967	1.59467E-34	*TLR6*	-0.726614599	0.000241529
MDGA1	0.974562039	1.85285E-22	*AP003108.2*	-0.726397022	0.013576038
AC245452.1	0.974544761	5.64227E-07	*SMAD9*	-0.726341966	0.000825986
MICALL2	0.974089348	3.13671E-36	*AC006254.1*	-0.72549182	0.009531824
GAD1	0.973987202	7.05633E-23	*NEK11*	-0.725445136	0.003354648
GPC1	0.973864855	3.00566E-46	*ELFN1*	-0.725364188	1.27125E-05
RAB3B	0.973550432	6.99357E-43	*AC104389.5*	-0.725214029	5.06083E-06
NBPF20	0.972837728	4.06048E-24	*METTL15*	-0.723984489	4.42912E-20
STX1A	0.971441659	1.01399E-28	*DISC1*	-0.723760879	0.014918823
AL022069.3	0.970902962	0.000491674	*USP28*	-0.723341202	1.10589E-18
ITGA3	0.970221497	4.04927E-83	*MAP3K4*	-0.723208809	9.40167E-22
HOGA1	0.969180541	1.59204E-11	*KCNMB2-AS1*	-0.723202135	0.016034245
PPP1R3F	0.967162039	1.86137E-05	*CTSO*	-0.722302025	0.000820868
DEPP1	0.967063539	9.35754E-15	*SERGEF*	-0.721737567	9.03808E-11
COL11A2	0.966260514	3.70332E-25	*FAM117A*	-0.721726352	2.04789E-11
KIF24	0.96468065	1.53456E-14	*ATP9B*	-0.721449607	5.75368E-10
NDRG1	0.964679307	1.4007E-25	*SCMH1*	-0.721223014	1.21757E-13
BX842570.1	0.963928559	1.74396E-06	*NDUFB10*	-0.721132427	1.87083E-17
PRAG1	0.96306789	7.45831E-33	*SNRNP48*	-0.720776837	3.30739E-19
EPB41L4B	0.963046437	4.34254E-35	*PEX11A*	-0.720399577	7.52351E-08
USP2	0.962801835	8.91806E-12	*LRRC23*	-0.720352136	0.000117707

PLEKHG1	0.962485243	2.13341E-31	*RPS2P5*	-0.719351076	5.24014E-05
WDR47	0.961295765	2.51755E-31	*GANAB*	-0.719317791	4.97117E-61
ITGA2	0.960694744	6.38475E-38	*HEXIM2*	-0.719072681	3.83007E-11
PPM1A	0.95987494	4.87918E-52	*SCG2*	-0.719069183	2.3729E-12
HSPB8	0.958756657	2.52853E-07	*DNAJC3*	-0.719054376	1.37487E-31
AC080112.2	0.958684886	3.16E-05	*SNHG10*	-0.718876636	3.4153E-06
AC137932.3	0.958137221	0.000712829	*SUMF1*	-0.718276062	1.39883E-25
NMB	0.957608008	6.63816E-10	*TMEM14B*	-0.717935065	9.24681E-20
TUBA4A	0.956853257	1.94987E-35	*NAP1L1*	-0.717180221	2.89878E-39
CCP110	0.956546551	3.3859E-51	*ZSWIM5*	-0.716458019	0.005756285
ZNF195	0.954319944	2.1942E-39	*HS2ST1*	-0.715968925	1.70563E-31
CLTB	0.954001062	1.13763E-34	*IFT88*	-0.715505566	9.40966E-11
CEP250	0.952002074	4.29885E-75	*TMEM218*	-0.715073139	3.08619E-13
PNPO	0.951839965	5.91391E-83	*HAGHL*	-0.715071323	1.27176E-13
GINS4	0.95038165	1.32997E-37	*CIRBP*	-0.714739592	1.94428E-29
UPK3BL1	0.950346386	2.39452E-07	*KRTCAP3*	-0.713831023	1.5823E-15
CCDC34	0.95003212	1.93865E-29	*LRBA*	-0.713696094	1.70751E-32
TONSL	0.949369154	7.63981E-28	*PDLIM5*	-0.712037723	3.83776E-36
DMPK	0.94758838	3.11778E-38	*AL445423.3*	-0.711528583	0.018945364
CHD5	0.946834157	2.53652E-27	*NUCKS1*	-0.711464728	6.34032E-33
ZNF707	0.946767942	5.81603E-18	*RPS10-NUDT3*	-0.711159602	4.86914E-14
AL589743.1	0.946355498	4.68677E-15	*FABP5*	-0.709826557	1.34603E-21
MSX2	0.946010195	3.83318E-30	*CC2D2A*	-0.709615118	3.09658E-11
AC026401.3	0.942770275	4.89148E-08	*CDKN1C*	-0.709580493	2.81236E-08
PLK2	0.94273591	4.76357E-71	*MAGOHB*	-0.709507408	1.28229E-10
LINC00663	0.942707378	0.00072991	*CFL2*	-0.709326551	2.08288E-22
MARVELD3	0.942319322	2.58011E-43	*MSRB2*	-0.708951839	1.41877E-11
TMC7	0.94220484	1.44439E-17	*FAM104B*	-0.708843543	6.24582E-05
KRT8P33	0.941716118	6.32615E-05	*FGFR4*	-0.707924498	2.07259E-20
BAK1	0.941254146	1.16458E-30	*APIP*	-0.707555148	3.56706E-20
AC119673.3	0.940979151	3.77664E-08	*DNAJC19*	-0.707464604	1.32178E-14
C12orf4	0.940431935	1.37027E-24	*CEP70*	-0.706629559	1.6994E-17
F3	0.940270471	8.00607E-72	*NT5DC2*	-0.706612902	3.77539E-33
BORCS7	0.939844438	1.00192E-28	*SLC38A2*	-0.705306219	1.99274E-25
AC068946.2	0.939212776	4.44986E-06	*INPP5A*	-0.705190727	5.3711E-11
SUSD2	0.938761695	9.58759E-32	*ISOC2*	-0.705178649	6.08666E-15
ABCA7	0.937288639	5.30119E-47	*MAPT*	-0.705104761	1.42841E-25
CHST3	0.93645887	2.49335E-35	*NAA38*	-0.704515344	0.000366386
APLF	0.936045299	1.30395E-05	*LMBRD1*	-0.70439421	3.87205E-11
CCDC9B	0.935279382	7.3922E-36	*SEPTIN6*	-0.704136964	1.04347E-14
HR	0.935021013	6.95564E-27	*KDELR3*	-0.70407421	4.66071E-27
ZNF324	0.934114389	3.63116E-18	*ZMYM3*	-0.703172986	1.4097E-29
FP671120.5	0.933191298	1.11773E-08	*SH2D3C*	-0.703171114	0.002829202
ZNF385A	0.931658337	4.72497E-28	*AL445524.1*	-0.702811079	0.001918529
SFXN5	0.931363193	1.09441E-17	*PMFBP1*	-0.702509939	0.004814137
MEAK7	0.931250304	2.32246E-66	*RAB26*	-0.702464103	8.4063E-18
NWD1	0.931131311	5.97473E-06	*SLC30A9*	-0.701028448	2.9478E-28
LIMA1	0.92992868	1.19558E-62	*TCAF1*	-0.700988637	3.17076E-31
AC021087.5	0.929285778	0.000955671	*ARHGAP24*	-0.700899668	1.61796E-07
AP002478.1	0.92815891	4.42731E-05	*GCNT1*	-0.700140797	5.62503E-08
GJB2	0.927571699	1.6561E-44	*MAPKAPK5-AS1*	-0.699742326	7.68162E-15
GCSAM	0.925691269	0.004987601	*RALGPS2*	-0.699207971	1.21071E-14
AC022007.1	0.925612744	3.66146E-06	*SNRPF*	-0.698431156	7.14197E-17
AC055811.4	0.924113519	6.756E-07	*SSR2*	-0.697972272	2.99072E-33
ABL2	0.923632275	1.62501E-33	*ATP5MC1*	-0.697202211	2.76853E-15
ERVK13-1	0.922269141	3.64464E-12	*HNRNPL*	-0.696992346	3.48406E-53
TMEM139	0.921953109	1.11533E-06	*STX8*	-0.696946387	1.47016E-08
ACTN1	0.921907181	2.81733E-67	*TXNDC5*	-0.696628136	1.46095E-69

TIAF1	0.921745576	7.27301E-05	*CENPV*	-0.696028627	2.74357E-12
C18orf25	0.92049334	7.10617E-42	*FOXRED1*	-0.69578136	1.98863E-18
ISYNA1	0.919442277	5.40742E-37	*CENPH*	-0.695721788	5.6004E-10
ZNF746	0.918978283	3.99836E-46	*BEAN1*	-0.695048429	0.036707587
ATP10D	0.918305038	1.99009E-17	*CNTNAP1*	-0.694736798	5.20916E-11
SLFN5	0.917701202	4.66067E-30	*MINDY2*	-0.694716694	7.28685E-16
RPS6KA1	0.917459651	9.25374E-52	*ZNF100*	-0.694710545	0.000303548
HAP1	0.916764531	1.62389E-16	*NDUFA1*	-0.694687002	8.84058E-08
UBALD2	0.916753329	6.19249E-32	*RPS9*	-0.694558379	1.83552E-41
C12orf45	0.916193343	1.37146E-24	*SNRPE*	-0.694250595	2.77021E-17
AC100810.3	0.915076512	0.002267277	*DSC2*	-0.693990442	9.46724E-27
HLA-DOA	0.914226038	5.58519E-06	*CCNQ*	-0.693707431	1.41304E-10
SCARF1	0.913317739	6.16637E-07	*COMMD8*	-0.693643393	1.14107E-09
GALNT18	0.910777461	5.40991E-32	*TCFL5*	-0.693441471	2.50317E-16
RPL23AP53	0.910538126	0.000140407	*RTN2*	-0.69320775	3.75436E-10
NBPF19	0.910402254	5.13955E-10	*RABAC1*	-0.693019109	2.9572E-17
PRSS27	0.9103056	0.000205977	*ICK*	-0.692731925	7.1532E-20
NOTCH2NLC	0.909594515	2.70214E-08	*ECPAS*	-0.692707033	1.87139E-27
SHC4	0.909066947	0.00239574	*NUP35*	-0.692343305	4.08872E-14
DLK2	0.908436391	1.51678E-07	*ATP11C*	-0.692153816	6.15928E-19
SBK1	0.908057596	2.4207E-31	*GMDS*	-0.690730392	4.54912E-12
PADI2	0.907460359	2.51794E-05	*TBL1XR1*	-0.690720827	4.69137E-21
PCNX2	0.90672055	9.46283E-29	*ZNF738*	-0.690253235	0.001440976
AAK1	0.90587192	1.24975E-28	*FAM72D*	-0.690209721	4.42776E-07
DACT3	0.905206315	6.53395E-07	*SRSF7*	-0.689971464	4.06363E-28
PIM1	0.905177545	6.58713E-21	*TMEM106A*	-0.689812819	0.017867499
AC004466.1	0.904789412	0.000584218	*ARHGDIB*	-0.689721141	0.001035105
TGFB3	0.904544835	0.000426035	*HPS3*	-0.689211964	3.98649E-12
TGS1	0.904307763	2.19822E-43	*SELENOH*	-0.689204987	1.29277E-21
WHRN	0.903482118	8.83303E-25	*ZFC3H1*	-0.689133197	1.39218E-19
CLEC16A	0.902204443	1.3798E-31	*TMEM123*	-0.689095474	1.33072E-26
AC020978.5	0.902089873	2.04031E-17	*DANCR*	-0.68869507	1.51939E-20
APOBEC3H	0.901958137	0.00021466	*THOC7*	-0.688576409	2.78902E-16
SDC1	0.901501531	4.07879E-39	*TBC1D22A*	-0.688201941	1.99299E-16
LINC02298	0.901499815	5.98362E-06	*PCCA*	-0.688061432	4.13709E-13
GK	0.900183842	2.53462E-12	*CHURC1-FNTB*	-0.687805285	0.023759312
HOXB6	0.900179503	3.89815E-47	*AL357075.3*	-0.687797061	0.012345216
KLK6	0.900093277	2.39395E-31	*MAOB*	-0.687371881	3.46069E-15
TMEM255B	0.899624717	3.50891E-12	*ERMAP*	-0.687049561	7.49291E-05
ESCO2	0.899433917	4.38424E-29	*CCDC7*	-0.686275959	0.020428985
CLMN	0.899133032	6.51468E-46	*SEC11A*	-0.686243219	3.17702E-32
AC027020.2	0.898445828	0.001378302	*PTK6*	-0.686065457	1.57544E-07
RHBDF1	0.898271923	8.92496E-20	*ERI1*	-0.685764381	1.567E-23
MEX3B	0.89694895	1.53476E-08	*MRPS34*	-0.685731354	5.67163E-18
TRIM9	0.896650267	5.82233E-08	*ENOX2*	-0.685521747	1.61604E-09
TMEM175	0.89603101	3.5369E-20	*TLE6*	-0.684887915	2.72683E-06
CD83	0.895339435	1.31954E-20	*RPL38*	-0.684222888	0.002824361
AOC2	0.894831017	2.41765E-08	*POT1*	-0.684101813	6.00749E-14
ALDH1A3	0.893473924	9.15846E-73	*CCDC78*	-0.683834202	1.76067E-09
MORC3	0.892948749	2.8036E-31	*TESMIN*	-0.683830855	2.63446E-10
PRRG4	0.892695729	1.03701E-32	*DCPS*	-0.683713012	6.65358E-15
PSORS1C1	0.891910288	3.54646E-05	*NLN*	-0.683218031	9.515E-30
AATK	0.891282228	2.98479E-11	*BANK1*	-0.683188045	5.08437E-06
KLLN	0.890884979	6.15183E-09	*LIMCH1*	-0.683173004	1.09543E-11
IDUA	0.890480042	3.98301E-16	*TMEM60*	-0.682949534	1.5214E-06
GM2A	0.889360503	1.93435E-63	*SPRYD3*	-0.682659216	4.67596E-28
CD68	0.888718395	8.38944E-19	*ELP4*	-0.682531727	1.09304E-12
AC087289.3	0.888350805	0.000140428	*CA11*	-0.682330949	2.12547E-18

ZNNT1	0.888011405	5.76186E-08	*ABHD10*	-0.681632896	5.64211E-22
AC016026.1	0.887493514	2.07004E-07	*SATB2*	-0.681283739	8.35255E-12
CCNK	0.886655593	2.49712E-68	*COQ3*	-0.681249703	1.01354E-08
SOWAHB	0.886429841	3.72866E-15	*EIF3E*	-0.681201918	1.18668E-29
MARCHF2	0.885894549	7.76746E-21	*AC004803.1*	-0.680646199	0.02975775
HCP5	0.884429813	1.03904E-11	*AC068888.1*	-0.680631188	1.14092E-05
IL12A	0.883118184	2.62288E-05	*PHB2*	-0.680610151	2.52226E-50
PLEKHA6	0.882330154	3.40783E-11	*LZTFL1*	-0.680458969	7.66804E-15
NT5E	0.881183657	5.28885E-38	*COMMD6*	-0.680316934	2.67001E-08
NEU3	0.881120078	2.13721E-40	*TSEN15*	-0.67987984	2.03771E-15
SMG1P7	0.879997008	0.003786871	*PPM1N*	-0.679824163	0.000243719
MAN2B1	0.879931622	1.30223E-32	*UGDH*	-0.679487535	6.11713E-25
E2F2	0.879710208	2.93797E-27	*SARDH*	-0.679310429	1.55101E-07
TUSC2	0.879291621	3.53047E-29	*LMAN1*	-0.677400376	8.30935E-27
MXRA8	0.878632398	0.000111461	*ARSK*	-0.676989402	2.78466E-06
RCBTB2	0.878574197	5.0133E-06	*PARVB*	-0.676941131	7.33021E-16
TCF19	0.876885763	1.42217E-28	*HLA-DMB*	-0.676852201	0.00446643
RIPK4	0.876593773	8.58538E-22	*METTL18*	-0.675913034	2.75069E-05
DNAJB2	0.876401247	1.40663E-32	*C1QBP*	-0.675802493	6.67931E-59
DERA	0.875632903	3.78524E-27	*TMED2*	-0.674919607	5.9763E-39
ANKRD19P	0.875538973	2.82267E-19	*ZNF395*	-0.674795719	1.69381E-25
UBASH3B	0.875099075	1.5198E-81	*U47924.2*	-0.674705238	0.01786375
MON2	0.875062718	2.34408E-30	*APEX1*	-0.674429361	3.33674E-54
ZNF564	0.874815678	2.11578E-11	*NQO1*	-0.674303045	2.3474E-38
AC137932.2	0.873931204	0.000836017	*FKBP4*	-0.673498245	8.87062E-35
SMPDL3B	0.873900913	2.42284E-19	*PGAP4*	-0.673295173	1.64389E-20
MOB3C	0.873808493	4.45941E-22	*GORAB*	-0.673242361	5.242E-07
ARC	0.873594513	1.19533E-16	*SMDT1*	-0.672939538	6.18481E-10
NUDT4B	0.873571598	0.037124659	*SAYSD1*	-0.67266261	9.7931E-07
SYTL3	0.873536484	9.99805E-39	*LDHB*	-0.672362843	1.29507E-46
CDC42EP3	0.873232594	2.69696E-43	*BCL9*	-0.672343301	2.55394E-20
HLA-DRB1	0.872736402	8.7784E-12	*AOPEP*	-0.672193379	3.0522E-06
BX890604.2	0.871311279	2.94159E-39	*PLCB3*	-0.672058975	4.67418E-26
C17orf80	0.870670732	3.69449E-38	*TRIQK*	-0.671516603	5.85756E-17
GSTT2	0.869393043	3.13011E-05	*FUT2*	-0.671062693	1.11511E-06
CCDC84	0.869302463	9.30717E-19	*CCDC110*	-0.670935576	0.003138041
MARVELD2	0.869055844	5.31871E-37	*MIF4GD*	-0.670756689	5.15366E-14
PPP4R3A	0.868767047	1.29657E-63	*GFPT2*	-0.670449369	8.61592E-12
CNGA1	0.866794531	0.000884536	*LDHA*	-0.670095689	3.05212E-29
RNF182	0.864189767	5.97525E-22	*STRADB*	-0.669798158	6.77664E-11
CDCP1	0.863816824	1.37702E-57	*ARL2*	-0.669749924	2.01828E-13
NKX3-1	0.863799047	8.51881E-16	*SLC1A3*	-0.669425444	1.47503E-20
RAB3A	0.863522941	4.48878E-19	*FRG1HP*	-0.668589309	0.00036589
ZNF468	0.862631936	7.69308E-17	*CYP4V2*	-0.668449897	2.5925E-11
GSTT2B	0.8622502	2.22771E-22	*CKAP4*	-0.66820456	1.1175E-36
MICB	0.862185324	2.89622E-44	*MOSPD3*	-0.66755318	9.98426E-11
SHC2	0.861350755	9.443E-06	*HSPA8*	-0.66729164	5.14742E-42
GARNL3	0.86098483	4.69164E-09	*UBE2E3*	-0.667037463	7.2416E-19
PPP2R5B	0.859866804	2.89003E-23	*EFNB2*	-0.667026083	1.19276E-20
CMTM3	0.859699169	2.57738E-25	*RTN4RL2*	-0.666922404	8.03581E-09
GPRIN1	0.858550767	7.07361E-22	*CCDC12*	-0.666699333	3.75039E-14
CFAP46	0.858233028	0.005292831	*DPH6*	-0.666584134	3.48793E-07
PITPNM1	0.857072319	2.8323E-30	*CKLF*	-0.666551011	2.54888E-08
B3GNTL1	0.856851405	2.86592E-23	*COX7C*	-0.666300951	4.18747E-06
AC092338.3	0.856766879	0.006390831	*FOCAD*	-0.66628542	4.49216E-22
S100A3	0.854860088	6.505E-06	*NAGA*	-0.666247874	5.02313E-18
AC016588.2	0.854802261	1.74322E-06	*C12orf57*	-0.665970707	1.44492E-05
GPR155	0.854684517	2.34461E-14	*LOXL2*	-0.665843509	3.195E-10

KIAA1586	0.853381958	7.98889E-22	*KIF18A*	-0.665425712	2.09601E-13
PKD1P1	0.852767204	0.000476057	*RELA-DT*	-0.665393558	0.038660872
NETO2	0.85197741	2.3254E-23	*RPS29*	-0.665091563	0.001722564
C6orf132	0.851720673	9.48917E-29	*AKR1E2*	-0.665077706	0.027954336
COL27A1	0.85040611	1.81015E-21	*PECR*	-0.66482833	1.05635E-08
UNC13D	0.850116618	1.16832E-15	*SMIM30*	-0.664346536	1.71896E-17
PRKX	0.849563603	6.52413E-51	*CRELD2*	-0.663723129	8.0175E-27
ATP13A3	0.8495197	1.31474E-38	*C2orf15*	-0.662467963	0.010952015
TRIM26	0.848673414	8.35849E-52	*AC100810.1*	-0.66209584	2.0873E-06
SCRIB	0.848415041	7.56822E-29	*CELF5*	-0.661678651	0.000158813
TMPO-AS1	0.848259618	7.32245E-06	*SPDYC*	-0.6605131	0.023151646
TMEM256-PLSCR3	0.847293165	0.016127208	*C1RL*	-0.660447791	3.85849E-16
CDK1	0.846038812	1.88984E-26	*EIF3FP3*	-0.660304874	1.37761E-05
ATP6AP2	0.845954396	5.25285E-46	*REEP6*	-0.660198988	1.82215E-11
TAX1BP3	0.845131432	7.4873E-26	*CTPS2*	-0.660041754	1.07361E-14
SECTM1	0.844837702	1.60927E-14	*ANO6*	-0.659386037	2.71194E-24
C11orf24	0.844779926	1.70358E-27	*FRG1BP*	-0.659371161	0.006270786
PLEKHN1	0.844713433	2.58573E-16	*PAFAH1B3*	-0.659339509	1.63569E-15
NEDD4L	0.843152543	2.96241E-50	*ENPP5*	-0.65876086	2.40056E-08
SOCS1	0.842729224	1.15473E-05	*MPND*	-0.658153866	3.51313E-11
BHLHE40	0.842242431	4.03468E-35	*TCAF1P1*	-0.657977144	3.84045E-05
C2CD2L	0.841483915	1.62192E-26	*AKAP6*	-0.657690589	0.011618242
F8	0.841392274	2.16345E-11	*DYNC2H1*	-0.657471699	0.005250634
HRAS	0.840806784	2.96655E-23	*NAV3*	-0.656417371	9.12071E-06
NDUFAF8	0.840507008	1.14251E-18	*TNS2*	-0.655761673	6.8481E-24
SYNJ1	0.839714428	2.7764E-18	*LIFR*	-0.6551504	2.02207E-10
PLEC	0.838913754	8.22259E-30	*C15orf41*	-0.655002434	5.59446E-08
ATP6V1D	0.838662722	4.34285E-33	*PAGR1*	-0.654973948	2.9187E-18
BLVRB	0.838607992	1.46111E-30	*PROS1*	-0.654498058	1.6129E-12
DOC2B	0.838592103	2.3919E-22	*ZNF22*	-0.654370131	5.99279E-11
IDH3A	0.837680473	1.55257E-47	*XYLT1*	-0.654240353	1.23674E-06
AC012676.1	0.837619694	0.000393297	*WDR18*	-0.65423426	6.74266E-14
BTBD11	0.8372541	1.15377E-22	*ATP5MD*	-0.65416657	0.000228743
CARD10	0.836679137	2.23046E-31	*RASA1*	-0.653750077	2.51353E-18
GNA11	0.836507735	4.07935E-35	*MBNL1*	-0.653709625	8.21301E-23
PPP1R15A	0.834749972	5.68106E-34	*SRD5A3*	-0.653347455	5.54213E-14
AC145098.2	0.834713775	0.002670174	*AC010442.1*	-0.653339722	3.9366E-05
C6orf226	0.833600593	0.000285997	*DCLK2*	-0.653088825	5.9103E-05
FGF18	0.833012091	0.002213905	*EGLN3*	-0.653031077	1.49715E-06
SENP3-EIF4A1	0.832519656	0.024657302	*CRYM*	-0.652932818	5.06883E-05
ATG4D	0.832258604	2.64123E-17	*FAM20C*	-0.652709348	5.51712E-11
KCNG3	0.832219676	0.000695886	*TWIST1*	-0.652521502	0.001005405
TRHDE-AS1	0.831177278	2.70306E-11	*CEP128*	-0.652379699	2.28038E-06
RTCA-AS1	0.831109263	0.000965983	*TPD52L1*	-0.652136437	3.29111E-20
HSD17B7	0.830291304	3.83547E-18	*ERH*	-0.652020439	1.48948E-15
CD33	0.830016143	0.000204638	*EMC2*	-0.651939933	3.09292E-11
VPS13D	0.829108366	1.7605E-34	*ACVR2A*	-0.65173204	2.58148E-10
BBS2	0.829082866	1.19713E-43	*FPGS*	-0.651723424	3.29731E-20
IRF5	0.827897417	1.10256E-24	*RHOT1*	-0.651383239	1.61648E-22
DDX12P	0.826983603	3.05453E-09	*EIF5A*	-0.65098382	8.1349E-49
TGFBR1	0.826349098	7.69306E-29	*CMC1*	-0.650878084	4.29812E-10
TFEB	0.826075262	1.58569E-09	*SKIL*	-0.650645297	2.53506E-12
GRAMD4	0.825408706	3.90396E-24	*NMU*	-0.65060135	4.6562E-15
CFLAR	0.825348534	1.15145E-32	*FKBP7*	-0.650451715	0.001214746
DENND2C	0.825041839	1.14901E-08	*TRA2B*	-0.650271316	8.38231E-42
KREMEN2	0.825007507	4.61649E-19	*TTC30A*	-0.650189929	0.005366969
AL022311.1	0.823989609	9.12754E-05	*SCN1B*	-0.650031325	3.40245E-08
SLC41A1	0.823618059	4.0132E-39	*AMMECR1*	-0.649992406	7.24726E-09

FAM111A	0.822671694	1.71069E-29	*TIAM1*	-0.6499451	1.0215E-22
PMEL	0.822515886	4.74789E-07	*ADAM10*	-0.64961929	3.66074E-24
NPM2	0.822052954	5.66101E-09	*NDUFAF2*	-0.649498464	4.63733E-07
AC108488.3	0.821822542	0.003055009	*RFXAP*	-0.649373288	0.000130891
ZNF843	0.82125319	0.000491067	*GRID1*	-0.64911543	1.70737E-06
HSD17B7P2	0.820515024	0.000123993	*ATP1B1*	-0.649063793	1.97633E-28
SEPTIN5	0.81887831	0.000231153	*FGF11*	-0.648805382	0.007397859
BCL2L11	0.818747634	1.17666E-29	*PPFIA4*	-0.648747065	4.95281E-06
FOXD2-AS1	0.818710638	1.80641E-06	*CAT*	-0.648656628	5.29685E-26
EI24	0.818667718	4.85059E-93	*PTER*	-0.64863585	2.11618E-17
IDI2-AS1	0.818013254	0.014469346	*RFXANK*	-0.648523373	4.60404E-15
NLRX1	0.817665707	8.55239E-21	*PCSK4*	-0.648266003	0.00020674
AC003681.1	0.81754754	0.001316544	*MPP6*	-0.648187611	3.91702E-12
PRKD2	0.817166845	1.14536E-33	*FER*	-0.647880389	1.64063E-16
TAGLN	0.815997176	1.35821E-05	*AL356019.2*	-0.647537065	0.013603502
PCCA-DT	0.815393572	7.14773E-07	*PCBD1*	-0.647225752	1.46123E-20
CCDC150	0.814881476	1.59505E-14	*VWA1*	-0.647170923	2.42181E-10
FAM229A	0.814737561	2.45613E-09	*AC006333.1*	-0.646497869	0.026153504
AC016629.3	0.813761036	0.000202212	*PDS5B*	-0.646249297	1.44825E-12
UPP1	0.811768937	2.05617E-10	*FAM201A*	-0.646209668	3.57571E-05
KRT8	0.81142306	6.27682E-62	*FBL*	-0.646209064	9.59995E-22
LINC02591	0.810684194	0.001149013	*HCFC1R1*	-0.645430682	2.40985E-15
ZNF185	0.8105105	9.78713E-39	*BTBD2*	-0.645199541	9.97493E-20
LINC00638	0.810445961	0.002693309	*FKBP9*	-0.645166132	5.9973E-39
STXBP3	0.810208945	7.95809E-29	*PEMT*	-0.645058195	1.06535E-10
AC138150.2	0.810008713	0.003738206	*HNRNPD*	-0.64497081	1.62481E-32
RETSAT	0.809764806	1.44024E-64	*GALNT6*	-0.644512763	1.74643E-11
MED20	0.809175588	5.99823E-30	*SLC35B2*	-0.64436926	8.31596E-26
NF2	0.807900827	3.86743E-58	*EHBP1*	-0.643368663	1.17387E-22
COL13A1	0.807620243	1.22623E-26	*ARHGAP19*	-0.642756221	5.95388E-23
PARD6A	0.807605986	2.73449E-08	*MAGI1*	-0.642359936	6.3555E-13
MRVI1	0.807208702	8.00433E-20	*AK8*	-0.642300751	0.01085558
KRT18	0.806515851	1.52196E-86	*CMTM8*	-0.64228432	3.69667E-08
ZGRF1	0.805860328	8.06347E-17	*LANCL1*	-0.642064204	8.0701E-20
DENND3	0.804838833	2.33702E-19	*SDR16C5*	-0.641791376	0.001248467
GMIP	0.804431407	1.44431E-17	*OSBPL10*	-0.641741794	4.63925E-17
PDLIM3	0.804145803	5.76106E-10	*ECSIT*	-0.641650187	9.90587E-11
IP6K2	0.803975594	2.39651E-61	*WDR60*	-0.641180446	4.84336E-10
OCLN	0.803735937	1.08529E-29	*WASHC3*	-0.640975812	8.18554E-06
LHX6	0.803444364	4.6153E-09	*TMA7*	-0.640955737	0.00090567
FAM104A	0.803226424	7.34463E-53	*CANX*	-0.640537575	6.20687E-36
SLC35G2	0.802717573	1.59817E-05	*AC092919.3*	-0.640193251	0.009798057
CEP97	0.802187658	1.53547E-18	*SNRPD1*	-0.639757421	1.52865E-23
PLIN2	0.801613738	1.64766E-21	*PPP1R14B*	-0.639727177	2.44599E-12
NEMP1	0.801528361	1.84551E-24	*UGT8*	-0.639718473	8.03564E-15
OAS3	0.801505707	5.70568E-35	*MRPL3*	-0.639320465	1.97899E-37
AHDC1	0.801149514	8.64536E-23	*LINC00869*	-0.639273386	1.35856E-05
RASSF5	0.800925878	1.8695E-11	*SNRPG*	-0.639228374	8.38839E-05
IGF2BP1	0.800380771	0.001473069	*ANXA1*	-0.638887272	2.59461E-32
PLIN4	0.800301703	1.66938E-06	*HPF1*	-0.638807768	1.34746E-11
AP002026.1	0.800218644	1.80694E-07	*SGPP2*	-0.638423103	0.00041754
ITGA2B	0.799575028	1.71127E-05	*TRPM4*	-0.63745133	7.031E-18
SYTL2	0.799404523	1.01744E-18	*CEP170*	-0.637194784	6.74378E-19
ITPKA	0.799060278	8.14987E-10	*RAB30-DT*	-0.63696943	0.001108622
MRPL49	0.799058608	1.56574E-49	*NARS2*	-0.636895396	1.11899E-16
BMP1	0.798671803	1.46413E-46	*MRPL11*	-0.635567044	1.39111E-16
POM121C	0.798535007	8.11348E-31	*FAM124A*	-0.635437642	0.00046029
QRICH2	0.798520259	1.6959E-05	*JADE1*	-0.635325172	3.95367E-17

ELL3	0.797838752	2.45326E-17	*NDUFB7*	-0.635034973	8.48805E-11
AC093827.4	0.797560801	0.005083383	*GLT8D1*	-0.63434409	4.16234E-20
LINC00174	0.797315246	6.83223E-11	*GPNMB*	-0.633966162	0.010665793
MCC	0.797286439	2.412E-34	*UFL1*	-0.633907709	2.78323E-16
AC022167.2	0.796925582	0.000603661	*GAR1*	-0.633852432	3.26771E-13
IL11	0.796704091	4.91156E-05	*STARD8*	-0.633733784	9.60015E-05
RALGAPB	0.796442342	9.37075E-30	*PAQR8*	-0.633448119	4.26589E-09
OSBPL7	0.795946249	1.64759E-25	*ZKSCAN3*	-0.632764849	1.0231E-09
STARD10	0.795127773	7.21277E-18	*KCTD15*	-0.632654937	8.62824E-16
TFCP2L1	0.794508406	2.05998E-20	*MARCKSL1*	-0.632647252	2.89146E-20
GSE1	0.794227341	6.39273E-45	*ELOA-AS1*	-0.632305944	0.020109047
STING1	0.794070042	2.98569E-32	*ZNF497*	-0.632195625	0.019043072
ADPRHL1	0.792806672	5.00841E-18	*ALPK1*	-0.631693016	1.84214E-08
MOAP1	0.792774861	4.50291E-23	*PLK4*	-0.631204525	5.3474E-14
MBD6	0.792492834	1.72352E-17	*NUS1*	-0.630986108	1.75638E-22
PEA15	0.792097473	9.50271E-36	*USE1*	-0.630821307	7.16493E-08
HRH1	0.791815565	1.96579E-12	*MTUS1*	-0.63042765	3.00486E-29
SLC7A6	0.791790207	1.34902E-27	*LBR*	-0.630067637	4.64173E-22
ZNF774	0.791277384	2.13496E-05	*MUC1*	-0.629998957	0.004891608
MICAL3	0.791219581	2.54017E-26	*STT3A*	-0.629577501	4.09242E-56
PGPEP1	0.791116513	3.67495E-42	*ANP32B*	-0.629221462	6.19912E-27
PERP	0.790684427	8.8234E-70	*TNFRSF18*	-0.629145718	8.06878E-08
TACC1	0.790647075	2.29632E-36	*CDCA7*	-0.629117018	1.33386E-16
TMEM201	0.790614818	3.61806E-19	*TMC3-AS1*	-0.629085154	0.048731553
CELSR1	0.789685351	5.31443E-24	*PRMT3*	-0.629068017	1.77314E-11
FRS3	0.789319231	3.48776E-09	*GNB1L*	-0.628754431	6.58539E-06
SMAD3	0.788704891	2.60952E-56	*KLHL13*	-0.628117858	1.57966E-05
AC068587.4	0.788374364	4.09793E-10	*ASMTL*	-0.627008586	8.03121E-21
RPS6KL1	0.788370762	1.07331E-07	*MACROD1*	-0.626888547	2.82472E-10
AQP3	0.788163682	2.7542E-06	*VPS9D1*	-0.626875805	3.7309E-05
VOPP1	0.787934467	5.69743E-37	*LINC01003*	-0.626817035	0.043968738
ZBTB17	0.78783924	4.10436E-24	*PTPN18*	-0.626796932	6.39126E-13
URB1	0.787333935	9.86728E-39	*KTN1*	-0.626658406	2.63388E-22
TSPAN1	0.787124171	4.01405E-31	*C11orf98*	-0.62662036	4.43472E-14
COQ6	0.787029085	1.07095E-18	*MORN1*	-0.626356232	2.20624E-06
CEACAM19	0.786617242	0.000559776	*LARP1B*	-0.626143828	4.02478E-15
MYH9	0.786363677	2.42875E-46	*PLCB2*	-0.625859817	0.034170273
UBXN11	0.785790571	8.3592E-17	*CYB5R2*	-0.625765559	2.02063E-05
RNF114	0.785505587	5.07797E-61	*TRIM59*	-0.625639297	2.50951E-10
CEMIP	0.783739244	7.81006E-07	*RMND1*	-0.62560201	6.65397E-14
RHOC	0.783419254	3.9704E-36	*SLC18B1*	-0.62553839	2.46434E-12
CDH24	0.782984801	2.09087E-17	*C10orf95*	-0.62547569	0.038149457
LINC00265	0.782527075	7.98855E-06	*TMEM147*	-0.62525015	2.10854E-15
ZNF383	0.782377688	2.75378E-09	*GATB*	-0.624959022	7.47466E-12
CRLF1	0.781305766	2.44429E-24	*RPL34*	-0.624105321	3.36713E-06
NCOR2	0.780975969	8.4402E-20	*NUDCD2*	-0.624073002	2.84305E-18
CERS5	0.779852999	3.81806E-31	*MCM5*	-0.623407311	4.03474E-26
AL669830.2	0.779730787	0.042108217	*PXMP2*	-0.623228794	2.26861E-07
RCL1	0.778542925	9.88604E-33	*PAICS*	-0.623174738	1.95465E-38
LINC01719	0.778272002	1.02052E-06	*PGLS*	-0.622491932	2.76611E-11
AC008966.1	0.777675993	2.79585E-09	*CHCHD6*	-0.622447464	1.81372E-11
GOLGA8B	0.776461412	5.59393E-20	*THAP9*	-0.621517808	0.000102289
NR2F2	0.776035973	1.53773E-36	*RPL23*	-0.620618447	1.01079E-24
VPS37B	0.775380009	7.60587E-46	*RPL31*	-0.620256669	2.43805E-15
CYP4F12	0.774930462	2.53236E-05	*HNRNPR*	-0.619957582	5.79651E-43
SUPT7L	0.774577411	4.47333E-37	*ALMS1*	-0.619317344	4.1734E-12
AC007128.1	0.774122876	0.004264504	*BEND3*	-0.618564385	4.43566E-08
MRI1	0.774086783	2.41629E-20	*EIF4E3*	-0.618545091	9.10548E-11

CHRFAM7A	0.773951509	0.005463959	*ADGRV1*	-0.618455497	0.000439646
RASGRF1	0.773318822	5.97779E-05	*GNL3*	-0.618161819	5.8704E-30
TCAF2	0.773228705	0.000260036	*CCDC191*	-0.617825782	2.93324E-05
CTSV	0.773199201	9.49169E-23	*G2E3*	-0.61724086	7.4986E-12
TNS4	0.772968207	4.23464E-30	*TBC1D5*	-0.61710854	3.66016E-21
GFOD1	0.772727311	6.64314E-10	*APRT*	-0.617097976	1.16454E-15
PROSER2	0.77258236	1.02528E-26	*ATXN1*	-0.616992972	5.49885E-09
MPZL2	0.772335808	3.2243E-20	*FAM241B*	-0.616756744	8.44839E-09
SAMHD1	0.77206677	5.34084E-28	*PDIA3*	-0.615155917	4.44091E-57
UNKL	0.77133875	4.89097E-16	*PRXL2C*	-0.615140646	5.22274E-11
SPRED3	0.770949813	7.2951E-05	*ARL4C*	-0.615114862	2.1581E-16
FSCN1	0.770758982	8.73531E-25	*PCTP*	-0.614367572	8.1007E-15
SLC35D1	0.770146	3.38223E-23	*B4GALT1*	-0.614259301	7.67475E-25
LINC02086	0.76934329	2.14494E-07	*CLIC3*	-0.614198233	3.14796E-05
MAP3K14	0.768793901	7.20283E-18	*SEC23A*	-0.61416517	1.02815E-27
TCF7	0.767341448	5.56191E-27	*TMEM18*	-0.613996013	8.1237E-12
NSMF	0.767047928	1.0291E-32	*FOLR1*	-0.613690617	3.00146E-05
DSE	0.76653468	2.31174E-41	*KAT5*	-0.613304639	5.79999E-15
SLC9A3R1	0.766129753	2.96023E-34	*SPCS2*	-0.61311915	7.63219E-33
BCL7A	0.765616451	3.36272E-23	*IPO5*	-0.612982032	9.23919E-27
SLC25A25-AS1	0.765590751	3.63139E-11	*TMEM223*	-0.612490107	3.09043E-08
SAC3D1	0.765053168	9.5236E-12	*MTDH*	-0.612308709	4.66604E-28
SCAT1	0.764942441	1.30879E-05	*LPAR3*	-0.611652545	3.96429E-20
TUBBP5	0.764172167	4.18223E-08	*MYCL*	-0.611542681	3.28561E-07
TEAD3	0.763978898	5.11281E-27	*AGER*	-0.611361129	0.000630728
CHRNA7	0.763780572	7.13514E-11	*PDIA6*	-0.611209127	9.13486E-35
IRF1	0.763278729	1.6439E-25	*KDELR1*	-0.610731736	2.84192E-33
ZNF761	0.763228701	1.98466E-14	*RRAS*	-0.610248631	1.65228E-14
LAMC2	0.763198397	5.72436E-43	*DPY19L3*	-0.610217525	4.79992E-10
SCD5	0.762612786	3.16956E-33	*TGFBR2*	-0.609861362	2.83865E-16
RFK	0.762217044	6.89925E-27	*FANK1*	-0.609808184	0.003863763
PALLD	0.762167971	2.92739E-54	*ACAT1*	-0.609473408	1.03839E-18
JAG2	0.7618229	2.66762E-27	*LHPP*	-0.609127784	4.55144E-13
ANKLE1	0.7615523	5.98236E-15	*SLC9A6*	-0.609083176	3.52668E-12
GDPD5	0.760857123	2.78887E-21	*MVD*	-0.60906534	1.14343E-14
TICAM1	0.76062958	7.21277E-18	*WWC3*	-0.608564916	4.24623E-10
S100A11	0.759295025	3.31768E-25	*CADM4*	-0.608522136	3.89529E-21
NAB2	0.759242631	1.00287E-27	*LYRM4*	-0.608267282	1.33663E-10
SLC16A10	0.759229876	1.50168E-15	*ERMARD*	-0.608150789	1.25754E-07
ABALON	0.759126015	8.92352E-18	*AC105460.1*	-0.607926968	2.1803E-10
TMEM68	0.759025402	1.16747E-15	*RNF141*	-0.607837144	7.85108E-18
YAP1	0.758648746	4.15928E-38	*LNCOC1*	-0.607807628	0.021454939
ZNF132	0.758570904	0.005674544	*SNHG3*	-0.607751755	1.10037E-12
MAFF	0.758504802	1.33579E-22	*HOXB8*	-0.6076997	8.43071E-17
TJAP1	0.758218711	3.48657E-28	*RPL36AL*	-0.607556922	8.92546E-14
MFGE8	0.758173515	6.54136E-38	*RSBN1L*	-0.607538068	1.01302E-18
TMOD3	0.757930071	2.95805E-24	*RIDA*	-0.607536688	2.44341E-09
AL157392.3	0.756036361	5.39743E-05	*RAN*	-0.607161841	4.13175E-50
LAMC1	0.755768068	6.45698E-62	*C11orf65*	-0.6069099	0.0475105
PLEKHM1P1	0.755117505	3.34661E-12	*LCORL*	-0.606774889	5.20459E-11
LUZP1	0.754959553	6.8601E-37	*WAC-AS1*	-0.606179493	5.5577E-06
ELF3-AS1	0.754813042	5.59874E-08	*CD55*	-0.606100957	8.55239E-21
PLCXD1	0.754365567	2.03793E-16	*DCBLD2*	-0.605837821	1.31746E-27
PLXNB2	0.754314942	1.28014E-27	*TCTEX1D2*	-0.6054361	0.008322099
TCN2	0.754205263	1.30591E-06	*TMEM39B*	-0.605193721	1.77628E-09
AC120114.3	0.753343095	0.009512367	*HEIH*	-0.605135691	0.010859602
MAPK11	0.753327352	8.48089E-23	*ARL4A*	-0.605099266	2.20794E-10
PNPLA2	0.753175957	2.55707E-22	*FAM149B1*	-0.60498841	1.01066E-09

SLC9A1	0.753135619	5.22288E-14	*VWA8*	-0.604675338	2.51911E-14
ZCCHC10	0.753068792	7.32454E-12	*RPGRIP1L*	-0.604626413	3.12306E-10
RFNG	0.752971226	1.62057E-22	*DOCK7*	-0.604509751	1.22584E-20
CDKN2B-AS1	0.752667242	0.002495693	*MTSS1*	-0.60415369	0.00498782
CHST14	0.752117924	3.11041E-21	*STAG1*	-0.604044581	9.83691E-18
ZNF211	0.751735821	1.70059E-07	*TMEM126A*	-0.604040881	1.4605E-08
C4B	0.751605491	0.001300479	*GRM4*	-0.603919758	0.004084152
MCAM	0.750437797	1.39147E-36	*HLCS*	-0.603377648	4.31954E-13
TSPAN10	0.750355477	0.0005639	*BDNF*	-0.603354148	3.40162E-19
CTSD	0.749651127	3.40491E-24	*CFAP43*	-0.603207058	0.001398075
AF117829.1	0.749532411	4.32606E-08	*BRK1*	-0.603205057	3.62981E-28
LAMA5	0.749408665	4.25994E-28	*GYS1*	-0.602600324	1.31097E-21
SHROOM3	0.748378357	2.07181E-36	*EPHA10*	-0.602599821	9.06381E-10
LONRF3	0.747514744	9.63702E-06	*LINC00173*	-0.602558748	1.068E-06
LTB4R2	0.74738693	9.09805E-05	*GABRG2*	-0.60255109	0.000368571
HIP1R	0.747000963	4.62403E-34	*FHOD3*	-0.601706979	5.14686E-07
HELZ2	0.746782231	2.86124E-18	*CATSPER2*	-0.60128206	0.015826055
MT2A	0.746686361	0.000740695	*ACOT13*	-0.601003837	1.82748E-13
POMZP3	0.746445613	5.02915E-17	*TNFRSF21*	-0.600435975	4.94183E-21
ST6GALNAC5	0.744590453	5.53657E-07	*RNF170*	-0.600344697	3.5296E-08
AOC3	0.742759512	2.02393E-05	*PTBP1*	-0.600109759	1.12869E-24
SSPO	0.742734639	9.57808E-07	*PRAME*	-0.600051345	6.85514E-18
VAMP8	0.742241991	1.52904E-33	*SLC9B2*	-0.599847621	4.61855E-10
BTG1	0.741245196	6.13629E-37	*SNX17*	-0.599442372	8.04816E-23
FAM131C	0.741240375	7.06812E-10	*CCDC68*	-0.598981803	1.22795E-05
ATP6V0C	0.741217004	6.18523E-25	*KBTBD7*	-0.598770074	2.82816E-10
ZFYVE16	0.741174268	3.85847E-20	*UNC5CL*	-0.59867718	0.03274854
NFATC3	0.740671238	8.23153E-28	*ZFP14*	-0.59866839	8.39845E-05
TERF2IP	0.740424328	1.36884E-45	*MARC2*	-0.598568752	5.85697E-09
SRPX2	0.739359138	5.09979E-07	*AK6*	-0.598331684	6.21277E-07
ANKRD11	0.739161545	1.57646E-49	*MTMR2*	-0.598306953	2.13021E-17
NPIPB11	0.739034973	0.000272228	*ATP5MC3*	-0.598293287	7.99686E-22
CDH1	0.738774172	7.98858E-39	*DIAPH3*	-0.597972056	3.56489E-12
OTULINL	0.738608363	3.71661E-15	*NPTN*	-0.597509385	4.7328E-24
APTX	0.738405225	1.10619E-28	*USP24*	-0.597195132	1.30992E-24
RADX	0.738323909	4.70455E-11	*HMGA2*	-0.597167354	3.28297E-21
MFSD4B	0.738213083	1.33089E-14	*LAMB1*	-0.597145162	1.05455E-25
FAM167A	0.738161815	1.31961E-08	*CHEK1*	-0.596811113	3.16845E-18
IL18BP	0.73811458	1.59756E-05	*GPRC5B*	-0.595854844	6.06373E-11
SIN3B	0.738083056	2.04178E-27	*DENND1B*	-0.595515902	2.92826E-06
TMEM64	0.737316702	1.1471E-24	*RPS14*	-0.595455104	6.94276E-18
IRS2	0.737024062	3.37154E-18	*KIF5A*	-0.595367757	1.21792E-07
DAPK1	0.736743112	2.59486E-10	*MIOS*	-0.595174566	4.47301E-12
ZNF845	0.736476401	3.90563E-07	*ANG*	-0.595081721	0.000275481
AC122688.3	0.736443361	0.00066667	*TLCD2*	-0.594982412	7.98758E-05
KIAA0513	0.735938769	7.12175E-19	*DLAT*	-0.594797828	6.57608E-27
KLF4	0.735567151	7.58185E-16	*LINC01184*	-0.594407003	2.53298E-11
AC026471.1	0.734009389	4.52795E-06	*PHGDH*	-0.594405599	1.57104E-21
RIMBP3B	0.73372756	0.004224413	*EML4*	-0.594347566	6.31392E-24
TNIP1	0.73364358	1.1387E-34	*PDIA4*	-0.594295016	5.91648E-44
MTURN	0.733042567	5.58931E-16	*C2orf76*	-0.594157971	0.000320359
EXO1	0.732673206	5.64724E-16	*MEX3A*	-0.593797929	3.24444E-09
PAK6	0.732125102	1.04186E-17	*FZD3*	-0.593766788	3.68811E-08
DCP1B	0.732031804	1.28863E-17	*ABHD14B*	-0.593672097	1.00659E-21
MINK1	0.73050404	5.31015E-33	*TAF15*	-0.593342915	1.4995E-25
POLA2	0.730456452	1.44758E-13	*CTTNBP2*	-0.593074238	0.000399803
RASAL1	0.730339257	2.89073E-14	*NDUFA7*	-0.592750423	9.33215E-08
CACNB4	0.729940656	0.00015692	*LINC01232*	-0.592692958	0.013155867

AL049840.2	0.729426459	0.001976865
EFR3B	0.729245517	0.00087431
SLC39A4	0.729007667	1.87114E-14
ZNF416	0.728003329	0.001916333
PRR5-ARHGAP8	0.727852716	0.000480308
PCBP4	0.727252033	9.06892E-30
RBM14	0.726249744	1.52519E-26
FAM50A	0.724903843	4.51541E-33
PICK1	0.724408647	1.6994E-17
TMEM59L	0.724207415	5.48411E-15
AC009090.6	0.723981676	0.000147289
ZBTB7C	0.723956704	0.005602499
CDH3	0.723798906	2.18761E-66
STOM	0.723295198	3.61087E-25
HERC5	0.722853904	1.42223E-18
ENO2	0.721294441	6.49855E-31
GOLGA8O	0.720485534	9.58535E-05
ZFPM1	0.720409884	2.11249E-09
CDK18	0.719833978	6.69015E-26
SMAD5	0.718701864	3.58366E-29
SF3A3	0.718552505	1.20942E-39
PODXL	0.718433399	1.40188E-26
COL4A5	0.717381538	3.47735E-27
PJA1	0.717317331	1.78716E-24
CTNNBIP1	0.716745792	7.21277E-18
FUCA1	0.716170261	1.16429E-39
NSD1	0.716043851	9.26943E-44
STARD5	0.716032246	7.14212E-06
STARD9	0.71572706	6.22414E-15
GRAMD2B	0.715452806	4.03568E-11
AC040162.3	0.714249096	0.000278662
STRADA	0.714095478	3.37976E-20
CECR7	0.713607992	1.67404E-07
BIK	0.71277446	5.91802E-13
TMEM121	0.712545368	9.94594E-07
AC093724.1	0.712312243	0.035085551
TOB1	0.711804917	4.81129E-37
TEX9	0.710861551	5.11768E-07
NDUFV3	0.710829966	8.52724E-30
AL049840.6	0.710133476	0.000561608
PRRT4	0.709754528	1.66704E-05
STRIP2	0.709603966	7.74294E-15
ATP6V0E2	0.709439158	2.11401E-16
FOSL1	0.709327272	7.22407E-27
DVL3	0.709226557	2.26084E-30
TRIP6	0.709010631	5.89007E-25
KDM4D	0.707965157	0.000464885
SP6	0.707624558	3.5741E-24
LDB1	0.707064221	5.60915E-42
IGSF9B	0.705402514	4.73371E-17
ZDHHC9	0.704656226	8.13979E-24
AL136295.5	0.703754225	0.026435821
ZNF814	0.702949833	2.89675E-05
STPG1	0.702849663	1.06391E-12
RBM18	0.702416774	1.14826E-30
SLC17A7	0.701063132	0.000865238
ZNF701	0.700916068	0.001448035
SIX1	0.700784936	2.10466E-08
CYP3A5	-0.592683013	0.000438784
SLC16A7	-0.592563033	3.7564E-07
ARL13B	-0.592478722	2.85165E-06
SLC37A4	-0.591965176	1.46564E-18
NBEA	-0.591808885	5.08734E-06
DYNC2LI1	-0.59109643	8.45835E-08
HMGN5	-0.590958483	6.33849E-09
FEZF1-AS1	-0.590201209	8.90338E-05
PTRHD1	-0.59017233	3.64163E-06
COL4A3	-0.589962747	2.74984E-06
EGFL7	-0.589943182	1.48162E-11
CDKN3	-0.589750874	1.32338E-11
PSMG4	-0.589307715	5.44338E-07
PHLPP1	-0.588542999	2.10196E-11
DECR1	-0.588360657	2.93406E-13
PPP1R3B	-0.588357326	2.4847E-16
TRIM69	-0.58807461	4.52103E-11
BUB1	-0.58771418	5.26641E-13
KANK1	-0.587625542	2.46266E-13
KHDRBS1	-0.587615311	1.82654E-52
ABCB10	-0.586280308	2.03344E-15
NEDD1	-0.586010537	3.99469E-12
PIK3CD-AS2	-0.585862235	0.002446329
EIF2A	-0.585795292	9.63407E-36
FKBP3	-0.585451058	5.79397E-17
SYNPO	-0.585288862	5.4662E-10
NUDT18	-0.58525288	6.12952E-05
RP9P	-0.585098803	0.000175869

SPHK1	0.700743208	4.4895E-06
MASP2	0.699699734	0.022231376
LCP1	0.699637529	3.32689E-05
CMIP	0.699437635	3.98393E-34
KCTD13	0.698990817	4.49173E-11
SLC12A7	0.698882093	1.27462E-18
TENT4B	0.698516775	1.38334E-21
DTX2P1-UPK3BP1-PMS2P11	0.698336562	5.54966E-05
AL118506.1	0.69766313	0.000142748
DCAKD	0.697625813	9.09264E-20
GPSM1	0.697279264	4.57599E-16
TNFAIP8	0.697120575	4.67766E-13
TNRC6C	0.696988969	6.22084E-23
ADAT1	0.696404515	6.27801E-15
IRF2BP2	0.696161275	1.34803E-34
SNX18P3	0.695383498	0.002451173
KDM7A	0.695185739	3.83318E-08
AC091230.1	0.694802286	5.57396E-27
SRSF8	0.694762561	2.12486E-30
GPR176	0.693925659	0.003764211
BCL11B	0.693465401	0.00440585
AC068580.4	0.692577982	1.25294E-06
PI4KB	0.692454949	4.59685E-37
BORCS8	0.692312885	2.79714E-06
TTN	0.692234699	0.016733651
RRM1	0.691811172	3.96697E-34
ZNF674	0.691377592	9.24878E-08
CYP2D7	0.690994433	0.000404492
KLHL21	0.690282001	1.22847E-22
RHBDL1	0.689610273	3.51755E-06
PHOSPHO1	0.688954266	0.000563135
ZRSR2	0.688827451	1.48418E-09
VWF	0.688802724	0.009846225
FA2H	0.688641631	2.54637E-09
ZNF655	0.688631008	2.34008E-17
HSPBAP1	0.688416421	1.95987E-08
TNNT2	0.68796827	0.014664059
ZNF672	0.687642344	4.12072E-14
SP110	0.687466039	1.95602E-09
WDR37	0.686981886	1.93187E-16
SKA1	0.686819128	2.09047E-10
GOLGA8A	0.686171271	2.42661E-17
MYBL2	0.685933749	1.13244E-14
RIMBP3	0.685918264	0.000470921
USP48	0.685463409	1.22014E-36
VASN	0.685372885	1.75655E-06
ZNF296	0.685245329	1.06114E-07
ARID3A	0.684932213	1.78408E-20
AC079922.2	0.684805226	0.006323993
UBR1	0.684569043	1.60945E-21
NPIPA9	0.684551737	4.45011E-08
MORC4	0.68432729	3.49441E-34
MST1P2	0.68413485	0.000728236
WHAMM	0.684121913	2.09127E-14
AL022328.4	0.684103691	0.006071354
AL627309.6	0.684059769	0.009457232
NOTCH2NLA	0.683935228	4.10153E-07
TFAP2A	0.683179485	2.77613E-15

CCDC90B	0.683080706	3.28558E-24
ST6GALNAC2	0.682899467	1.0362E-06
ARRDC1	0.682619656	4.15548E-22
IGFBP6	0.681757497	4.89251E-19
CSPG4P10	0.681310981	1.92618E-05
POLR2A	0.681276239	9.46724E-27
C17orf100	0.680786041	0.004526159
SIPA1L3	0.680595037	7.56804E-13
MAP6D1	0.680395911	3.38615E-05
OSBP2	0.680367768	8.06942E-21
RAD51AP1	0.680232127	1.26517E-15
RPL23AP82	0.677402979	7.29948E-20
KRT19	0.677055587	7.79388E-29
STK10	0.676912272	1.59841E-23
RMC1	0.676665126	1.59851E-18
KIAA1549L	0.67617957	0.015920744
NUP62CL	0.675853835	5.60439E-08
RPAP2	0.675829857	1.07673E-20
SLC43A2	0.675423704	2.76875E-11
AC138969.3	0.675176854	4.83524E-05
ASCC3	0.675104171	5.52991E-24
TMBIM1	0.674879778	4.4802E-47
SNORC	0.674338231	0.000334714
KISS1R	0.674317604	0.026136059
C21orf58	0.674281842	1.18242E-05
PTK2B	0.674268906	1.02951E-14
UBALD1	0.673291278	1.05354E-10
CDKN2B	0.673164867	2.29038E-15
KCNC4	0.673026454	1.78948E-12
ANKRD13B	0.672859466	2.6499E-16
CROCCP3	0.672485458	0.00045552
PPP2CB	0.672408194	3.36773E-30
FBXO44	0.671740983	2.1351E-16
ZBTB4	0.671548338	1.08916E-35
DTX2	0.671496922	4.90724E-15
ZNF888	0.670971343	2.55783E-07
SLF2	0.670750674	3.87923E-21
ELMOD3	0.670750479	6.56357E-09
CITED4	0.670479434	0.000684089
LMF2	0.670413109	3.63352E-17
ORAI3	0.670131835	3.28254E-11
B4GALT6	0.669837622	7.96078E-15
MIR1915HG	0.669767023	1.65922E-09
C3orf62	0.669627071	3.38719E-07
AHNAK2	0.669539712	7.31489E-20
ARMC5	0.669194423	1.87019E-10
OSR2	0.668233086	3.63139E-11
ZSCAN20	0.667567617	2.3261E-06
ADRM1	0.667349985	1.60714E-18
PRSS22	0.667028844	6.86417E-07
FUT1	0.666668448	2.03866E-12
MPZL3	0.665785289	8.1721E-09
C11orf68	0.665341905	2.4783E-15
FAM185A	0.664640214	1.9817E-05
MFSD2A	0.664545739	7.87238E-06
SH2D3A	0.664277909	5.74761E-22
SMIM13	0.664225191	3.44331E-21
AC138028.4	0.66378095	1.33983E-05

CDKN2C	0.663597931	1.95368E-11
C3orf67	0.663292391	3.33552E-06
WWC1	0.662976336	2.10561E-44
SHFL	0.662931589	2.48207E-14
BCAR3	0.662669532	1.41449E-37
PPM1J	0.662397773	1.52722E-07
GPR162	0.662243237	1.42131E-05
SMPD3	0.661780079	0.000203002
ARHGEF19	0.661764707	1.64755E-23
EPS8L1	0.661602437	2.7869E-13
PDLIM1	0.661507069	1.16904E-33
RGS12	0.661055787	1.68458E-14
KAZN	0.660932503	8.87893E-13
HMOX2	0.660595279	2.27932E-25
DUSP10	0.660154218	0.000826706
SHC1	0.660130733	2.31342E-45
CTNNAL1	0.659930598	5.64756E-24
AC012676.5	0.659787525	0.007737111
ADGRA2	0.65914661	9.02606E-17
PHLDB1	0.658845598	1.2144E-30
TP53RK	0.657960619	1.09628E-13
ZNF767P	0.65765203	2.42923E-07
RHGAP27P1-BPTFP1-KPNA2F	0.657314719	6.32235E-06
RB1	0.656804091	1.09033E-24
MCTP1	0.656508109	0.032404111
ARIH2	0.655939148	1.40574E-37
PKMYT1	0.655649442	2.91924E-10
FZD5	0.655240479	6.15039E-18
DEPDC7	0.655065736	3.58438E-08
SMPD1	0.654810268	3.11335E-07
DGKZ	0.654514846	8.73568E-20
MOB3A	0.654341138	1.84121E-16
NTN4	0.654050649	2.60409E-09
AC116562.4	0.653595305	0.014070782
PLEKHM1	0.653058871	2.86317E-18
KCNIP2	0.652917945	4.66717E-05
FAM111B	0.652565929	1.03823E-08
LINC01588	0.652528545	0.001219807
CXCL16	0.652518816	5.41121E-23
SP100	0.652232602	1.28661E-23
CCDC43	0.651946042	2.01501E-19
LINC02035	0.651362764	2.15889E-07
DISP2	0.651225461	2.60415E-07
MAPK6	0.651062621	1.29255E-25
PTPRB	0.650967842	0.022631257
SOWAHA	0.650877212	0.019410728
MAFK	0.650609989	7.47821E-17
MGME1	0.650270924	5.81755E-28
PDGFA	0.650035078	1.59901E-17
SBDS	0.650034414	1.83732E-21
LINP1	0.650007946	2.4161E-07
GXYLT1	0.649922842	3.70126E-14
SH3TC1	0.649470145	1.67933E-07
LOXL3	0.649188673	0.003046527
KLHL7	0.649181428	7.80072E-20
ARHGAP40	0.649091332	0.004012445
AC037459.2	0.649064471	0.003135894
ZNF212	0.648709059	5.02578E-10

WDR1	0.648539341	1.24377E-40
SH3BP1	0.64847278	2.34968E-11
AC092299.1	0.647217355	0.000855332
WNT11	0.646836215	0.012930851
ALDH1L2	0.646819501	0.017215666
ZNF274	0.646615922	9.80936E-07
MYO5A	0.646170479	5.67004E-19
SLC5A5	0.645761632	0.019043072
C10orf88	0.644956302	8.30945E-15
MED15	0.644641551	1.66116E-20
KCND1	0.644505415	2.47941E-05
YPEL5	0.644490381	1.16585E-16
LHX4	0.643740618	0.00666031
GAMT	0.643260035	1.32283E-16
ZSWIM8	0.641744658	1.09785E-26
AC132812.1	0.64170458	0.003172809
PLEKHA8	0.641673683	8.48295E-13
PDGFB	0.641387617	5.9167E-22
AC020916.1	0.641384639	1.30792E-05
GLIPR2	0.641246199	7.98716E-17
GRB7	0.640888325	3.92829E-17
SLC4A8	0.64086446	0.000559776
NME1	0.640738648	9.4533E-25
LYG1	0.640734226	0.013678348
TUBG1	0.640678276	2.25708E-20
SKI	0.640508547	3.06244E-20
ITGA1	0.63992282	0.034551149
CENPU	0.639804364	1.99918E-14
CELSR2	0.639574008	2.54223E-13
TMEM151A	0.639062014	1.12518E-07
PABPC1L	0.639018715	4.939E-21
PELI3	0.638632596	6.50628E-06
CCDC18-AS1	0.638546379	2.72756E-08
DOCK3	0.638498376	4.00262E-14
FAM13A-AS1	0.638457202	0.027755559
SLC7A8	0.637886457	9.62981E-08
INPP5J	0.63782744	1.97702E-08
SLC27A4	0.637357628	7.45922E-18
IDS	0.636998175	4.93457E-24
EAF1	0.63685462	5.03776E-31
CAPN1	0.636814655	2.38377E-29
ZCCHC12	0.636533537	0.014318176
AC018638.4	0.636290397	0.004564053
PXN	0.636130734	2.16934E-37
MOK	0.6361253	1.90889E-08
DAGLA	0.63514556	7.50488E-08
CAPN2	0.634832676	2.00054E-59
MAPK8IP2	0.634776846	1.13268E-05
ZNF699	0.634497274	7.30521E-06
PHACTR4	0.634089772	5.84656E-28
PANK2	0.633410364	7.7523E-26
ELL2	0.632880504	1.87869E-15
POLR3GL	0.63267272	1.18076E-15
TJP1	0.632213031	7.38596E-28
ANXA4	0.631979323	4.47161E-25
PANK4	0.631117026	1.53464E-13
PHRF1	0.630830269	1.6408E-18
NANP	0.63076068	2.83066E-11

MSANTD2	0.630458442	4.04987E-07
RAB27B	0.630409809	7.06215E-08
SF3A1	0.630391987	4.57229E-41
PLEKHG6	0.630238712	1.5945E-15
THEMIS2	0.630073458	2.12927E-08
PRR14	0.629729008	1.13142E-13
ABCC10	0.629502131	1.29848E-23
PRDM11	0.629454266	3.00836E-12
PCNP	0.629267396	8.53611E-29
CTPS1	0.629138137	7.27804E-43
IRF6	0.629136128	8.3534E-28
DOP1A	0.629081662	1.40613E-07
TMEM52	0.628796609	1.28777E-05
WASH2P	0.628524781	1.2477E-08
SNRNP27	0.628463325	3.32859E-20
AC126755.1	0.62823289	2.00678E-10
WTIP	0.628121022	3.54477E-06
DCTN5	0.627711954	6.35108E-35
AC008267.5	0.627397205	0.015695142
TBC1D10A	0.627084238	4.54947E-12
LINC00205	0.626971474	2.83422E-14
NACC2	0.626952523	1.40253E-18
IFIT3	0.626920229	1.13286E-05
IRAK1	0.626850355	5.92211E-18
HIC2	0.626546737	5.16195E-12
ZNF513	0.625910289	8.40563E-10
AC245060.5	0.625725687	0.027781917
S1PR2	0.625428006	7.3254E-09
SGSH	0.625323696	8.98577E-13
FAM110C	0.625301962	1.08912E-18
ZNF343	0.625080242	1.27073E-12
UBE2J2	0.625075386	2.85196E-17
MTSS2	0.625051603	8.53341E-15
TENT5C	0.624894511	6.14482E-05
WDR81	0.624187306	1.43716E-12
SDSL	0.624184448	3.24196E-11
AC016876.3	0.623745213	1.47054E-05
CCNG2	0.623379633	3.8895E-16
ESRP2	0.623260623	1.3193E-22
UBTD1	0.623256733	6.06675E-09
KIAA1217	0.622976861	2.31901E-14
CGRRF1	0.622865487	4.69089E-07
AP001931.2	0.622804442	6.13485E-05
DDIT3	0.622567779	1.02291E-06
SUGCT	0.622268038	5.15721E-05
TTLL6	0.622143167	0.002876242
TM7SF3	0.621900032	8.30059E-28
TSKU	0.621571885	3.54505E-13
AC009779.4	0.62100819	0.000817386
DHRS7	0.620962845	8.50039E-30
RGL1	0.620690029	0.000225739
SLC25A30	0.620247514	6.55065E-17
SNRK	0.620052884	6.65711E-16
N4BP2	0.619470981	5.72564E-09
ZNF567	0.619227363	0.00011946
OPTN	0.619183478	3.17434E-24
GABRQ	0.619132221	2.42753E-07
CUL9	0.619116284	6.58713E-21

OSBPL3	0.618762954	8.32292E-23
SLC6A6	0.618756571	7.66972E-27
SCML1	0.617872614	1.95323E-07
SPG7	0.617758962	8.12429E-32
PSME4	0.61773903	5.83808E-23
ASPSCR1	0.617664378	1.01998E-21
SYNE3	0.617543316	2.34743E-09
SNN	0.61753506	7.05239E-20
ZNF143	0.616273164	1.46008E-17
MAP3K6	0.616219447	1.62842E-12
APOBEC3C	0.61586477	2.22642E-38
WSB1	0.615571318	2.3212E-13
AL513477.1	0.614767099	0.034055594
AC110079.1	0.614461697	3.11722E-06
CTAGE8	0.614293964	0.000190224
PGBD2	0.614274509	1.46007E-06
HAGH	0.614013236	7.72807E-12
ZBTB34	0.61368791	5.17855E-11
SARS1	0.613531913	1.60026E-28
SEPTIN9	0.613350483	5.16074E-25
HSDL2	0.61308509	3.80745E-22
OSR1	0.612726637	0.012737604
IFI6	0.612079255	9.26993E-09
AKIP1	0.612056997	3.30549E-15
ARHGAP8	0.611963202	2.81954E-16
DUBR	0.611640658	0.001786258
COL1A1	0.610964348	2.31287E-13
USH1C	0.610543375	2.71915E-08
IKBKB	0.610489794	8.33585E-19
ESRRA	0.610459913	7.21277E-18
NDUFA6-DT	0.609996608	0.000406309
ZNF488	0.609885839	5.03749E-08
REV3L	0.609367227	2.48159E-19
MTA2	0.609064791	2.36092E-33
NCOA4	0.609011516	6.49129E-35
CTSK	0.608988461	0.00498272
AC026412.1	0.608393196	4.23234E-08
TUBB2B	0.608056446	0.004911449
RAB21	0.607856866	1.4007E-25
FGFR1OP	0.607798875	2.80671E-11
PDLIM2	0.607433875	7.90349E-12
SLC25A15	0.607269575	1.44776E-20
HUS1	0.607137292	1.11794E-10
SASH1	0.607116015	7.20518E-12
SVIL	0.607036984	7.292E-19
CASP10	0.606737384	2.52703E-06
SIRT4	0.606466343	0.029791525
PINK1	0.606344741	4.56249E-13
STX12	0.606219835	6.77219E-14
UFD1	0.606038771	1.58745E-20
COQ10A	0.606009905	1.06703E-07
ZNF469	0.605792662	2.54782E-06
SPSB1	0.604923008	6.45329E-10
TMEM127	0.604903555	3.38223E-23
ERCC1	0.604750803	7.72777E-20
TSPYL2	0.604289738	6.46379E-20
RRN3P3	0.603860351	0.004496215
ANKFY1	0.603438919	1.95475E-26

GOLT1A	0.602909855	0.009472107
CYSTM1	0.602831162	4.06456E-12
GPATCH11	0.602712802	2.21391E-12
NCAPH2	0.602554601	8.26529E-14
ZNF341	0.601968963	8.90881E-07
FCHO2	0.601317354	3.12066E-08
HYAL3	0.601234056	2.20789E-06
GCC2	0.601099236	9.55845E-14
BRSK1	0.600912382	3.41584E-10
HS1BP3	0.600911821	6.41506E-16
MAP1LC3B	0.600865962	6.49157E-30
FTX	0.600540587	6.49785E-07
TP53I13	0.599186223	1.28307E-12
PARP3	0.599005001	1.32338E-11
RXRA	0.598975593	3.80054E-23
GUSBP11	0.598442808	4.44578E-11
CEP72	0.598246858	3.50061E-09
ZNF83	0.598246232	5.67085E-07
NRAV	0.598086947	4.14591E-07
IL10RB	0.597932679	6.91841E-14
DNAJC5	0.59791743	1.1182E-27
CYHR1	0.597786478	2.79775E-15
TICRR	0.597738356	1.27788E-12
ZNF611	0.597420425	0.000480204
AL358472.6	0.59721328	0.003019811
BVES	0.597047229	7.45778E-06
STAT2	0.596397568	8.6362E-21
JMJD7-PLA2G4B	0.59639658	7.6804E-09
PVT1	0.596360791	8.42609E-15
HDAC9	0.595890226	6.09231E-05
LINC00857	0.595004195	5.73568E-09
PDE12	0.593346559	7.35307E-19
REC8	0.593261795	0.005343027
FAM184A	0.593117573	0.016586759
METTL8	0.593070229	1.81413E-20
TRAPPC2B	0.592511208	0.017174768
GTF2IP20	0.592229659	6.59622E-05
IFRD1	0.591979583	7.16053E-14
MRE11	0.591134246	6.21346E-10
HLA-DRA	0.59101005	0.000235113
AL390195.3	0.590898851	0.00979864
ASAP1	0.590618323	4.07265E-19
TASOR2	0.590271617	4.2329E-28
PRKG2	0.589868085	0.0013739
FGFBP3	0.58899844	0.010453282
ASIC3	0.58857029	0.027182919
RRP12	0.588377486	1.87298E-25
NCR3LG1	0.58809042	0.003984261
PAXBP1-AS1	0.587978103	0.006476694
AP001062.1	0.587703583	0.019207612
SPATS2	0.587654285	2.49859E-29
ZNF703	0.586860751	2.88226E-05
JCAD	0.585497283	7.30261E-12
EMP2	0.584973611	1.79859E-18

Table S10. List of mRNAs to generate the K-means clustering heatmap.

Gene symbol	Cluster	MiR34_targets	DREAM_targets	E2F_targets
PADI3	1	No	No	No
ID3	1	No	No	No
IFI6	1	No	No	No
PIFO	1	No	No	No
SH2D1B	1	No	No	No
DUSP10	1	No	No	No
LINC01814	1	No	No	No
ID2-AS1	1	No	No	No
LBH	1	No	No	No
AC019069.1	1	No	No	No
PROM2	1	No	No	No
FN1	1	Predicted	No	No
GRIP2	1	No	No	No
VILL	1	No	No	No
TMEM158	1	No	No	No
HYAL1	1	No	No	No
TNNC1	1	No	No	No
MITF	1	No	No	No
ZBED2	1	No	No	No
SPICE1	1	No	No	No
MUC13	1	No	No	No
RBP1	1	No	No	No
LIPH	1	No	No	No
WDR53	1	No	No	No
APBB2	1	No	No	No
DAPP1	1	No	No	No
LINC02273	1	No	No	No
GASK1B	1	No	No	No
CEP72	1	No	Yes	No
DOK3	1	No	No	No
JARID2	1	No	No	No
CLIC5	1	No	No	No
AL135905.2	1	No	No	No
RAET1E	1	No	No	No
SUGCT	1	No	No	No
ERV3-1	1	No	No	No
AC016831.6	1	No	No	No
MNX1	1	No	No	No
MYOM2	1	No	No	No
AC018398.1	1	No	No	No
BAALC-AS1	1	No	No	No
SYBU	1	No	No	No
MTBP	1	No	Yes	No
TBC1D31	1	No	Yes	No
AC016074.2	1	No	No	No
LRATD2	1	No	No	No
CASC8	1	No	No	No
ADGRB1	1	No	No	No

LY6K	1	No	No	No
MINCR	1	No	No	No
MROH6	1	No	No	No
WDR97	1	No	No	No
KIFC2	1	No	No	No
RECQL4	1	No	Yes	No
DMRT1	1	No	No	No
PSAT1	1	No	No	No
FGD3	1	No	No	No
LCN2	1	No	No	No
LINP1	1	No	No	No
HKDC1	1	No	No	No
PRXL2A	1	No	No	No
NEURL1	1	No	No	No
ACSL5	1	No	No	No
LMNTD2-AS1	1	No	No	No
DGAT2	1	No	No	No
TMPRSS4	1	No	No	No
NINJ2-AS1	1	No	No	No
VWF	1	No	No	No
C1R	1	No	No	No
GPRC5A	1	No	No	No
KRT80	1	No	No	No
HSD17B6	1	No	No	No
AC025419.1	1	No	No	No
CPM	1	No	No	No
OAS1	1	No	No	No
EPSTI1	1	No	No	No
AL606834.1	1	No	No	No
DGLUCY	1	No	No	No
IFI27	1	No	No	No
RASGRF1	1	No	No	No
ALPK3	1	No	No	No
NR2F2	1	No	No	No
HAS3	1	No	No	No
FOXL1	1	No	No	No
WNK4	1	No	No	No
EPN3	1	No	No	No
CACNA1G	1	No	No	No
MYO15B	1	No	No	No
UNC13D	1	No	No	No
SPHK1	1	No	No	No
RAB27B	1	No	No	No
HCN2	1	No	No	No
FUT3	1	No	No	No
ZNF567	1	No	No	No
CD33	1	No	No	No
ZNF350	1	No	No	No
ZNF525	1	No	No	No
ZNF530	1	No	Yes	No

AL035661.1	1	No	No	No
FER1L4	1	No	No	No
WFDC3	1	No	No	No
B3GALT5-AS1	1	No	No	No
SH3BP1	1	No	No	No
CSDC2	1	No	No	No
PRR34-AS1	1	No	No	No
KLF8	1	No	No	No
PWWP3B	1	No	No	No
FHL1	1	Predicted	No	No
HAUS7	1	No	Yes	No
AL691432.2	2	No	No	No
GABRD	2	No	No	No
AL139423.1	2	No	No	No
DRAXIN	2	No	No	No
OTUD3	2	No	No	No
HTR1D	2	No	No	No
E2F2	2	No	Yes	No
IL22RA1	2	No	No	No
IFNLR1	2	No	No	No
GRHL3	2	No	No	No
GPR3	2	Predicted	No	No
PTAFR	2	No	No	No
LAPTM5	2	No	No	No
GJB3	2	No	No	No
MFSD2A	2	No	No	No
ZNF684	2	No	Yes	No
RAD54L	2	No	Yes	No
JUN	2	No	No	No
STXBP3	2	No	No	No
WDR47	2	No	No	No
TENT5C	2	No	No	No
TMEM81	2	No	No	No
TMCC2	2	No	No	No
SLC41A1	2	No	No	No
YOD1	2	No	No	No
GRHL1	2	Predicted	No	No
RRM2	2	Predicted	Yes	Yes
SLC30A3	2	Predicted	No	No
CYP1B1	2	No	No	No
EMX1	2	No	No	No
SFXN5	2	No	No	No
BCL2L11	2	No	No	No
TFCP2L1	2	Predicted	No	No
DHRS9	2	No	No	No
C2orf88	2	No	No	No
NRP2	2	No	No	No
VIL1	2	No	No	No
TUBA4A	2	No	No	No
DOCK10	2	No	No	No

GBX2	2	No	No	No
ERFE	2	No	No	No
HES6	2	No	No	No
AC022007.1	2	No	No	No
EOMES	2	No	No	No
SNRK	2	No	No	No
UBA7	2	No	No	No
SEMA3F	2	No	No	No
SLC38A3	2	No	No	No
DUSP7	2	Predicted	No	No
KBTBD8	2	No	No	No
CEP97	2	No	Yes	No
C3orf52	2	No	No	No
GCSAM	2	No	No	No
HEG1	2	Predicted	No	No
LAMP3	2	No	No	No
EPHB3	2	No	No	No
IL1RAP	2	No	No	No
N4BP2	2	No	No	No
NIPAL1	2	No	No	No
CNGA1	2	No	No	No
AREG	2	Published	No	No
LIN54	2	No	Yes	No
ABCG2	2	No	No	No
ZGRF1	2	No	Yes	No
CLGN	2	No	No	No
OTULINL	2	No	No	No
TNFAIP8	2	No	No	No
MARCHF3	2	No	No	No
SOWAHA	2	No	No	No
HBEGF	2	No	No	No
GPRIN1	2	No	No	No
MAK	2	No	No	No
SMIM13	2	No	No	No
CD83	2	No	No	No
TCF19	2	No	Yes	Yes
ATP6V1G2-DDX39B	2	No	No	No
C2	2	No	No	No
C4B	2	No	No	No
ETV7	2	No	No	No
MDFI	2	No	No	No
ULBP2	2	Published	No	No
SYNJ2	2	Predicted	No	No
AC007566.1	2	No	No	No
AC105052.2	2	No	No	No
STRIP2	2	No	No	No
KDM7A	2	No	No	No
EZH2	2	No	Yes	Yes
NKX3-1	2	No	No	No
TACC1	2	No	No	No

GINS4	2	Predicted	Yes	Yes
MYBL1	2	No	Yes	No
RDH10	2	No	No	No
PAG1	2	No	No	No
FSBP	2	No	No	No
SPAG1	2	No	No	No
MAFA	2	No	No	No
TONSL	2	No	Yes	No
CD274	2	Published	No	No
KIF24	2	No	Yes	No
SERPINH1P1	2	No	No	No
AL162231.2	2	No	No	No
GLIPR2	2	No	No	No
ANKRD20A1	2	No	No	No
CENPP	2	No	Yes	No
ZBTB34	2	No	No	No
HSPA14_1	2	No	No	No
CDK1	2	No	Yes	Yes
ARID5B	2	No	No	No
EGR2	2	No	No	No
UNC5B	2	No	No	No
PANK1	2	No	No	No
ENTPD1-AS1	2	No	No	No
SLF2	2	No	Yes	No
ELOVL3	2	No	No	No
PLEKHA1	2	Predicted	No	No
NKX1-2	2	No	No	No
ADAM12	2	Published	No	No
DPYSL4	2	Predicted	No	No
DUSP8	2	No	No	No
H19	2	No	No	No
RRM1	2	No	Yes	No
E2F8	2	No	Yes	Yes
CCDC34	2	No	Yes	No
PRRG4	2	No	No	No
TP53I11	2	No	No	No
CHRM4	2	No	No	No
CLP1	2	No	No	No
FAM111A	2	No	Yes	No
NXF1	2	No	No	No
FRMD8	2	No	No	No
OVOL1	2	No	No	No
P2RY2	2	No	No	No
AP002761.4	2	No	No	No
RELT	2	No	No	No
DDIAS	2	No	Yes	No
BIRC3	2	No	No	No
MPZL2	2	No	No	No
SCN3B	2	Predicted	No	No
CCDC15	2	No	Yes	No

DDX12P	2	No	No	No
AC092821.2	2	No	No	No
RAPGEF3	2	No	No	No
VDR	2	No	No	No
FMNL3	2	Predicted	No	No
PMEL	2	No	No	No
NEMP1	2	No	Yes	No
E2F7	2	No	Yes	No
TMTC3	2	Predicted	No	No
TMPO-AS1	2	No	No	No
OAS3	2	No	No	No
BICDL1	2	No	No	No
BCL7A	2	No	No	No
GJB2	2	No	No	No
LACC1	2	No	No	No
CDH24	2	No	No	No
BRMS1L	2	No	No	Yes
TRIM9	2	No	No	No
GPR137C	2	No	Yes	No
GCH1	2	No	No	No
PPM1A	2	Predicted	No	No
SIX1	2	No	No	No
ARG2	2	Predicted	No	No
STON2	2	No	No	No
CHRFAM7A	2	No	No	No
INAFM2	2	No	No	No
OIP5	2	No	Yes	No
ITPKA	2	No	No	No
CEP152	2	No	Yes	No
CA12	2	No	No	No
CORO2B	2	Predicted	No	No
THAP10	2	No	No	No
CEMIP	2	No	No	No
MEX3B	2	No	No	No
PKMYT1	2	No	Yes	No
AC026401.3	2	No	No	No
CCP110	2	No	No	Yes
SBK1	2	No	No	No
NETO2	2	Predicted	No	No
RRAD	2	No	No	No
AC020763.4	2	No	No	No
MARVELD3	2	No	No	No
AC092718.8	2	No	No	No
FOXF1	2	No	No	No
DOC2B	2	No	No	No
TRPV3	2	No	No	No
SLC52A1	2	No	No	No
LINC00324	2	No	No	No
AC005747.1	2	No	No	No
SARM1	2	No	No	No

SLC46A1	2	No	No	No
TOP2A	2	No	Yes	Yes
TUBG1	2	No	Yes	Yes
HROB	2	No	Yes	No
SP6	2	No	No	No
NGFR	2	Predicted	No	No
COIL	2	No	No	No
SEPTIN4	2	No	No	No
RAD51C	2	No	Yes	Yes
C17orf80	2	No	No	No
CDR2L	2	No	No	No
FBF1	2	No	No	No
TEN1-CDK3	2	No	No	No
AC015802.6	2	No	No	No
METRNL	2	No	No	No
LPIN2	2	No	No	No
CEP76	2	No	No	No
AP005482.4	2	No	No	No
PMAIP1	2	No	Yes	No
RNF152	2	No	No	No
SERPINB8	2	No	No	No
PARD6G	2	No	No	No
DOT1L	2	No	No	No
TINCR	2	No	No	No
ANGPTL4	2	No	No	No
ZNF699	2	No	No	No
SPC24	2	Predicted	Yes	Yes
ZNF441	2	No	No	No
ZNF440	2	No	No	No
ZNF564	2	No	No	No
C19orf57	2	No	No	No
NOTCH3	2	Predicted	No	No
AC005336.1	2	No	No	No
CYP4F11	2	No	No	No
ANKLE1	2	Predicted	No	No
AC008397.2	2	No	No	No
ZNF101	2	No	No	No
ZNF506	2	No	No	No
CCNE1	2	Published	Yes	Yes
DPF1	2	No	No	No
SERTAD1	2	No	No	No
PLAUR	2	No	No	No
LYPD5	2	No	No	No
RELB	2	No	No	No
LIG1	2	No	No	Yes
KCNA7	2	No	No	No
ZNF845	2	No	No	No
PCNA	2	No	Yes	Yes
MGME1	2	No	No	No
NANP	2	No	No	No

NOL4L	2	No	No	No
KCNB1	2	No	No	No
HELZ2	2	No	No	No
FP565260.3	2	No	No	No
BTG3	2	No	No	No
SYNJ1	2	Predicted	No	No
ICOSLG	2	No	No	No
PDXP	2	No	No	No
NCAPH2	2	No	Yes	No
GK	2	No	No	No
PIM2	2	No	No	No
RADX	2	No	No	No
LONRF3	2	No	No	No
ELF4	2	No	No	No
INTS6L	2	No	No	No
F8A3	2	No	No	No
RNF207	3	No	No	No
SPCS2P4	3	No	No	No
AZIN2	3	No	No	No
BMP8A	3	No	No	No
PPIEL	3	No	No	No
ABCA4	3	No	No	No
AC093157.1	3	No	No	No
PBXIP1	3	No	No	No
RIT1	3	No	No	No
TGFB2	3	Predicted	No	No
TRIM67	3	No	No	No
SLC35F3	3	No	No	No
FAM49A	3	No	No	No
DNAJC27-AS1	3	No	No	No
WBP1	3	No	No	No
LINC00342	3	No	No	No
LIMS2	3	No	No	No
COL4A3	3	No	No	No
DNER	3	Predicted	No	No
ARL4C	3	No	No	No
PLCD1	3	No	No	No
TCTA	3	No	No	No
DNAH12	3	No	No	No
TMEM45A	3	No	No	No
PLD1	3	No	No	No
NAALADL2	3	No	No	No
CXCL8	3	No	No	No
ARHGAP24	3	No	No	No
INPP4B	3	No	No	No
FSTL5	3	No	No	No
AC138866.2	3	No	No	No
AC010501.2	3	No	No	No
VCAN	3	No	No	No
EFNA5	3	No	No	No

EPB41L4A	3	No	No	No
SIL1	3	No	No	No
NRG2	3	No	No	No
PCDHGB7	3	No	No	No
STK32A	3	No	No	No
ADAM19	3	Predicted	No	No
DRD1	3	No	No	No
H3C6	3	No	No	No
AL645929.1	3	No	No	No
AL662795.2	3	No	No	No
PHF1	3	No	No	No
EFHC1	3	No	Yes	No
AL359715.1	3	No	No	No
TARID	3	No	No	No
TCF21	3	No	No	No
TMEM184A	3	No	No	No
AC007009.1	3	No	No	No
HDAC9	3	No	No	No
ITGB8	3	No	No	No
HECW1	3	No	No	No
STAG3L1	3	No	No	No
BHLHA15	3	No	No	No
FOXP2	3	Published	No	No
AASS	3	No	No	No
LINC01006	3	No	No	No
AC027117.1	3	No	No	No
DUSP4	3	No	No	No
AC090578.3	3	No	No	No
SNTB1	3	No	No	No
KCNQ3	3	No	No	No
GLIS3	3	No	No	No
FAM189A2	3	No	No	No
MAMDC2	3	Predicted	No	No
CEMIP2	3	No	No	No
TNFSF15	3	No	No	No
TTLL11	3	Predicted	No	No
AL162586.1	3	No	No	No
AL157935.3	3	No	No	No
ST6GALNAC4	3	No	No	No
PIP5KL1	3	No	No	No
CERCAM	3	No	No	No
QRFP	3	No	No	No
STKLD1	3	No	No	No
SLC2A6	3	No	No	No
DBH-AS1	3	No	No	No
AJM1	3	No	No	No
CLIC3	3	No	No	No
PNPLA7	3	No	No	No
VIM	3	No	No	No
SGMS1-AS1	3	No	No	No

SPOCK2	3	No	No	No
ANKRD1	3	No	No	No
PAOX	3	No	No	No
SPON1	3	No	No	No
SERPING1	3	No	No	No
SLC3A2	3	No	Yes	No
ALDH3B1	3	No	No	No
AP001972.5	3	No	No	No
MMP13	3	No	No	No
PDE3A	3	No	No	No
SLCO1B3	3	No	No	No
CNTN1	3	No	No	No
GLT8D2	3	No	No	No
DCLK1	3	No	No	No
KCTD12	3	No	No	No
GAS6-AS1	3	No	No	No
AL138479.2	3	No	No	No
LTK	3	No	No	No
HOMER2	3	No	No	No
ADAMTS17	3	No	No	No
MSLN	3	No	No	No
ENO3	3	No	No	No
ZNF594	3	No	No	No
ARL4D	3	No	No	No
SPATA20	3	No	No	No
CUEDC1	3	Predicted	No	No
WIPI1	3	Predicted	No	No
DTNA	3	Predicted	No	No
CCDC102B	3	No	No	No
CYB5A	3	No	No	No
AC004623.1	3	No	No	No
ANO8	3	No	No	No
ZNF91	3	No	No	No
ZNF30	3	No	No	No
THAP8	3	No	No	No
MIA	3	No	No	No
GFY	3	No	No	No
ZNF134	3	No	No	No
AC020915.3	3	No	No	No
L3MBTL1	3	No	No	No
LINC01271	3	No	No	No
FNDC11	3	No	No	No
AL121845.1	3	No	No	No
RUNX1	3	No	Yes	No
AP001066.1	3	No	No	No
SCARF2	3	No	No	No
CASTOR1	3	No	No	No
RNF215	3	No	No	No
C1QTNF6	3	Predicted	No	No
NHS	3	No	No	No

WAS	3	No	No	No
TSC22D3	3	No	No	No
ARHGEF6	3	No	No	No
L1CAM	3	Published	No	No
SCNN1D	4	No	No	No
MXRA8	4	No	No	No
HES2	4	No	No	No
TNFRSF9	4	No	No	No
KLHDC7A	4	No	No	No
HSPG2	4	No	No	No
NCMAP	4	No	No	No
SERINC2	4	No	No	No
TINAGL1	4	No	No	No
COL16A1	4	No	No	No
COL9A2	4	No	No	No
BTBD19	4	No	No	No
TSPAN1	4	No	No	No
ECHDC2	4	No	No	No
WDR78	4	No	No	No
SLC44A5	4	No	No	No
ST6GALNAC5	4	No	No	No
SLC44A3-AS1	4	No	No	No
INKA2-AS1	4	No	No	No
S100A10	4	No	No	No
HRNR	4	No	No	No
S100A2	4	No	No	No
LMNA	4	Predicted	No	No
AL590560.3	4	No	No	No
GPA33	4	No	No	No
PTPRVP	4	No	No	No
MFSD4A	4	No	No	No
C1orf116	4	No	No	No
LAMB3	4	No	No	No
PXDN	4	No	No	No
TP53I3	4	No	No	No
EFR3B	4	No	No	No
KIF3C	4	Predicted	No	No
UCN	4	No	No	No
XDH	4	No	No	No
CRIM1	4	No	No	No
TTC7A	4	No	No	No
DYSF	4	No	No	No
AC159540.2	4	No	No	No
AC092683.1	4	No	No	No
EDAR	4	No	No	No
MIR4435-2HG	4	No	No	No
GALNT5	4	No	No	No
AC009299.1	4	No	No	No
SCN2A	4	No	No	No
MAP2	4	Predicted	No	No

ABCA12	4	No	No	No
AC068946.1	4	No	No	No
GPR55	4	No	No	No
TMPPE	4	No	No	No
TRANK1	4	No	No	No
KIF9-AS1	4	No	No	No
LAMB2	4	No	No	No
AC104452.1	4	No	No	No
NICN1	4	No	No	No
DOCK3	4	No	No	No
NR1I2	4	No	No	No
CSTA	4	No	No	No
ALDH1L1	4	No	No	No
AMOTL2	4	No	No	No
IL20RB	4	No	No	No
LPP-AS2	4	No	No	No
MYL5	4	No	No	No
IDUA	4	No	No	No
CTBP1-DT	4	No	No	No
MXD4	4	No	No	No
HGFAC	4	No	No	No
DOK7	4	No	No	No
MSX1	4	No	No	No
SORCS2	4	Predicted	No	No
AC093827.4	4	No	No	No
SPRY1	4	No	No	No
TRIML2	4	No	No	No
ADAMTS16	4	No	No	No
DAB2	4	No	No	No
AC025171.2	4	No	No	No
PLK2	4	No	No	No
KRT8P33	4	No	No	No
AC011346.1	4	No	No	No
SH3TC2	4	No	No	No
GM2A	4	No	No	No
FAT2	4	No	No	No
CPLX2	4	No	No	No
MYLK4	4	No	No	No
C6orf52	4	No	No	No
EDN1	4	No	No	No
CDKN1A	4	No	No	Yes
MDGA1	4	Predicted	No	No
AL365205.1	4	No	No	No
PTCHD4	4	No	No	No
AL590428.1	4	No	No	No
COL12A1	4	Predicted	No	No
LAMA4	4	No	No	No
AL096711.2	4	No	No	No
SYNE1	4	No	No	No
AC073957.3	4	No	No	No

AP5Z1	4	No	No	No
RASA4CP	4	No	No	No
LINC02604	4	No	No	No
PMS2P3	4	No	No	No
SEMA3C	4	No	No	No
MYH16	4	No	No	No
SLC12A9	4	No	No	No
IFRD1	4	No	Yes	No
CICP14	4	No	No	No
CPA4	4	No	No	No
ATP6V0A4	4	No	No	No
TMEM139	4	No	No	No
SSPO	4	No	No	No
ATG9B	4	No	No	No
FGF17	4	No	No	No
CLU	4	No	No	No
LINC00589	4	No	No	No
AC131254.1	4	No	No	No
AC027702.2	4	No	No	No
DNAJC5B	4	No	No	No
AC132219.2	4	No	No	No
AF117829.1	4	No	No	No
AC087752.3	4	No	No	No
AC067930.5	4	No	No	No
TMEM249	4	No	No	No
ABCA1	4	No	No	No
AL138756.1	4	No	No	No
SUSD1	4	No	No	No
CEL	4	No	No	No
NALT1	4	No	No	No
CYSRT1	4	No	No	No
MIR1915HG	4	No	No	No
NRP1	4	No	No	No
HSD17B7P2	4	No	No	No
AL022344.1	4	No	No	No
ANXA8	4	No	No	No
AL591684.2	4	No	No	No
ZNF365	4	No	No	No
COL13A1	4	No	No	No
PPP3CB-AS1	4	No	No	No
C10orf55	4	No	No	No
KLLN	4	No	No	No
FGFBP3	4	No	No	No
MORN4	4	Predicted	No	No
LOXL4	4	No	No	No
PYROXD2	4	No	No	No
SEC31B	4	No	No	No
SFXN3	4	No	No	No
COL17A1	4	No	No	No
DMBT1	4	No	No	No

AL731571.1	4	No	No	No
CEND1	4	No	No	No
TRIM22	4	No	No	No
KIAA1549L	4	No	No	No
DAGLA	4	No	No	No
ROM1	4	No	No	No
LBHD1	4	No	No	No
CCDC88B	4	No	No	No
AP003068.2	4	No	No	No
CST6	4	No	No	No
USP35	4	No	No	No
UPK2	4	No	No	No
SNX19	4	No	No	No
LINC02827	4	No	No	No
LRMP	4	No	No	No
MUC19	4	No	No	No
GPD1	4	No	No	No
KRT7	4	No	No	No
KRT81	4	No	No	No
AC121757.2	4	No	No	No
IGFBP6	4	No	No	No
AC076968.2	4	No	No	No
TAFA2	4	No	No	No
MDM2	4	No	No	No
BTBD11	4	Predicted	No	No
SDSL	4	No	No	No
ABCB9	4	No	No	No
DNAH10	4	No	No	No
GAS6-DT	4	No	No	No
RTN1	4	No	No	No
TMEM229B	4	No	No	No
DPF3	4	No	No	No
AHNAK2	4	Predicted	No	No
CRIP1	4	No	No	No
HERC2P3	4	No	No	No
THBS1	4	Predicted	No	No
CCDC9B	4	No	No	No
CHAC1	4	No	No	No
AC087482.1	4	No	No	No
SEMA7A	4	No	No	No
ST20-AS1	4	No	No	No
ISG20	4	No	No	No
VPS33B-DT	4	No	No	No
MSLNL	4	No	No	No
EME2	4	No	No	No
PDPK2P	4	No	No	No
AC092117.1	4	No	No	No
PRSS30P	4	No	No	No
ABAT	4	No	No	No
NUPR1	4	No	No	No

AC026471.1	4	No	No	No
AC007906.2	4	No	No	No
KIFC3	4	No	No	No
NDRG4	4	No	No	No
FBXL8	4	No	No	No
HSF4	4	No	No	No
C17orf107	4	No	No	No
AC004771.4	4	No	No	No
ALOX12P2	4	No	No	No
TOM1L2	4	Predicted	No	No
SLC47A2	4	No	No	No
LGALS9B	4	No	No	No
AC022916.2	4	No	No	No
MMP28	4	No	No	No
TBC1D3L	4	No	No	No
KRT16	4	No	No	No
JUP	4	No	No	No
OSBPL7	4	No	No	No
LINC02086	4	No	No	No
TTLL6	4	No	No	No
LRRC37A3	4	No	No	No
AC018665.1	4	No	No	No
TSPAN10	4	No	No	No
AC132938.6	4	No	No	No
AP000919.2	4	No	No	No
AC016588.2	4	No	No	No
GZMM	4	No	No	No
VMAC	4	No	No	No
CAPS	4	No	No	No
NWD1	4	No	No	No
IQCN	4	No	No	No
C19orf33	4	No	No	No
PRKCG	4	No	No	No
LENG8	4	No	No	No
BCL2L1	4	No	No	No
ABALON	4	No	No	No
MYL9	4	Predicted	No	No
NNAT	4	No	No	No
ARHGAP40	4	No	No	No
PABPC1L	4	No	No	No
TMEM189-UBE2V1	4	No	No	No
LIME1	4	No	No	No
SSR4P1	4	No	No	No
ZNF70	4	No	No	No
SUSD2	4	No	No	No
POM121L9P	4	No	No	No
TCN2	4	No	No	No
APOBEC3D	4	No	No	No
APOBEC3H	4	No	No	No
PDGFB	4	No	No	No

MIRLET7BHG	4	No	No	No
ADM2	4	No	No	No
ARSA	4	No	No	No
RPL23AP82	4	No	No	No
ASMTL-AS1	4	No	No	No
GRPR	4	No	No	No
AC078993.1	4	No	No	No
OPHN1	4	No	No	No
SRPX2	4	No	No	No
COL4A6	4	No	No	No
SMIM10L2A	4	No	No	No
PLXNB3	4	No	No	No
LINC01770	5	No	No	No
SMIM1	5	No	No	No
TNFRSF8	5	No	No	No
CCDC24	5	No	No	No
FPGT-TNNI3K	5	No	No	No
AC097059.1	5	No	No	No
VTCN1	5	No	No	No
PHGDH	5	No	No	No
LINC00623	5	No	No	No
LINC00869	5	No	No	No
SELL	5	No	No	No
CRYZL2P	5	No	No	No
ZNF648	5	No	No	No
HLX	5	No	No	No
ODC1-DT	5	No	No	No
ATP6V1C2	5	No	No	No
CCDC121	5	No	No	No
C2orf81	5	No	No	No
TMSB10	5	No	No	No
SH2D6	5	No	No	No
C2orf15	5	No	No	No
IL18R1	5	No	No	No
SULT1C2	5	No	No	No
ACOXL	5	No	No	No
MZT2A	5	No	No	No
GPR39	5	No	No	No
DPP4	5	No	No	No
TTC30A	5	No	No	No
ZNF385B	5	No	No	No
IKZF2	5	No	No	No
AC012512.1	5	No	No	No
AC077690.1	5	No	No	No
PRRT3-AS1	5	No	No	No
SLC6A20	5	No	No	No
C3orf18	5	No	No	No
ACOX2	5	No	No	No
AC093010.3	5	No	No	No
PLCH1	5	No	No	No

C4orf48	5	No	No	No
CC2D2A	5	No	No	No
FGFBP1	5	No	No	No
DANCR	5	No	No	No
EREG	5	No	No	No
CXXC4	5	No	No	No
SPEF2	5	No	No	No
AC008957.1	5	No	No	No
GPX8	5	No	No	No
MEF2C	5	No	No	No
KIAA0825	5	No	No	No
SPATA24	5	No	No	No
FABP6	5	No	No	No
RAB24	5	No	No	No
HULC	5	No	No	No
LST1	5	No	No	No
ARMC12	5	No	No	No
SUPT3H	5	No	No	No
EYS	5	No	No	No
CD24	5	Published	No	No
STX7	5	No	No	No
PACRG	5	No	No	No
RNASET2	5	No	No	No
ELFN1	5	No	No	No
ELFN1-AS1	5	No	No	No
BBS9	5	No	No	No
TRG-AS1	5	No	No	No
ASNS	5	No	No	No
LAMB1	5	No	No	No
LRRC61	5	No	No	No
AC022239.1	5	No	No	No
NCALD	5	Predicted	No	No
MAL2	5	No	No	No
CCN3	5	No	No	No
GSDMC	5	No	No	No
MAPK15	5	No	No	No
MFSD3	5	No	No	No
FRG1HP	5	No	No	No
TMC1	5	No	No	No
ANXA1	5	No	No	No
SUSD3	5	No	No	No
AL590705.1	5	No	No	No
KIF12	5	No	No	No
ECHDC3	5	No	No	No
FAM107B	5	No	No	No
ADAMTS14	5	No	No	No
SCD	5	Predicted	No	No
FANK1	5	No	No	No
LRRC27	5	No	No	No
AL451069.1	5	No	No	No

FUOM	5	No	No	No
RPL26P30	5	No	No	No
LINC02724	5	No	No	No
CDC42EP2	5	No	No	No
NEAT1	5	No	No	No
FOLR1	5	No	No	No
GSEC	5	No	No	No
GRIN2B	5	No	No	No
TSPAN8	5	No	No	No
AC090015.1	5	No	No	No
AC089983.1	5	No	No	No
TCP11L2	5	No	No	No
LINC00173	5	No	No	No
ADGRD1	5	No	No	No
AC126564.1	5	No	No	No
FLT3	5	No	No	No
GGACT	5	No	No	No
ITGBL1	5	No	No	No
LINC00460	5	No	No	No
CARMIL3	5	No	No	No
PCK2	5	No	No	No
AKAP6	5	Predicted	No	No
NKX2-8	5	No	No	No
AL365295.1	5	No	No	No
PAPLN	5	Predicted	No	No
PIF1	5	No	Yes	No
AP3B2	5	No	No	No
AC013489.1	5	No	No	No
NPW	5	No	No	No
HCFC1R1	5	No	No	No
MT1E	5	No	No	No
MT1F	5	No	No	No
TSNAXIP1	5	No	No	No
CTRL	5	No	No	No
SLC16A13	5	No	No	No
TMEM256	5	No	No	No
STX8	5	No	No	No
LYRM9	5	No	No	No
GHDC	5	No	No	No
CNTNAP1	5	Predicted	No	No
IFI35	5	Predicted	No	No
RND2	5	No	No	No
CCDC103	5	No	No	No
HEXIM2	5	No	No	No
HOXB8	5	Predicted	No	No
LIMD2	5	Published	No	No
BAIAP2-DT	5	No	No	No
AC027601.1	5	No	No	No
OXLD1	5	No	No	No
MRO	5	No	No	No

CCDC68	5	No	No	No
PCSK4	5	No	No	No
TJP3	5	No	No	No
BST2	5	No	No	No
MAP4K1	5	No	No	No
PPM1N	5	No	No	No
SULT2B1	5	No	No	No
FCGRT	5	No	No	No
CPT1C	5	No	No	No
TMEM74B	5	No	No	No
FRG1BP	5	No	No	No
HNF4A	5	Published	No	No
PLTP	5	No	No	No
AL133335.2	5	No	No	No
HMGB1P1	5	No	No	No
PPP1R3D	5	No	No	No
RPS21	5	No	No	No
LINC01694	5	No	No	No
GAL3ST1	5	No	No	No
ATF4	5	No	Yes	No
ASB9	5	No	No	No
AC112493.1	5	No	No	No
MAOB	5	No	No	No
RRAGB	5	No	No	No
TCEAL4	5	No	No	No
DUSP9	5	No	No	No
TP73	6	Predicted	No	No
LINC01355	6	No	No	No
AUNIP	6	No	Yes	No
CEP85	6	No	Yes	No
RPA2	6	No	Yes	Yes
IQCC	6	No	No	No
ZBTB8B	6	No	No	No
CLSPN	6	No	Yes	No
BMP8B	6	No	No	No
NASP	6	No	Yes	Yes
STIL	6	No	Yes	No
CDKN2C	6	No	Yes	Yes
ORC1	6	No	Yes	No
USP1	6	No	Yes	Yes
ALG6	6	No	No	No
ITGB3BP	6	No	Yes	No
CDC7	6	No	Yes	No
DIPK1A	6	No	No	No
TLCD4	6	No	No	No
SASS6	6	No	Yes	No
RBM15	6	No	No	No
DCLRE1B	6	No	Yes	Yes
H2BC19P	6	No	No	No
C1orf61	6	No	No	No

C1orf112	6	No	Yes	No
CENPL	6	Predicted	Yes	No
KIF14	6	No	Yes	No
UBE2T	6	No	Yes	Yes
ELK4	6	No	No	No
DYRK3	6	No	No	No
INTS7	6	No	Yes	No
DTL	6	No	Yes	No
LIN9	6	Predicted	Yes	No
CCSAP	6	No	Yes	No
EXO1	6	No	Yes	No
KLF11	6	No	No	No
GEN1	6	No	Yes	No
CENPO	6	No	Yes	No
GPATCH11	6	No	Yes	No
PRKD3	6	No	No	No
MSH2	6	No	Yes	Yes
MSH6	6	No	Yes	No
CHAC2	6	No	No	No
AC010733.2	6	No	No	No
PELI1	6	No	No	No
GMCL1	6	No	No	No
ALMS1-IT1	6	No	No	No
NCAPH	6	Predicted	Yes	No
ZC3H6	6	Predicted	No	No
TTL	6	No	No	No
CKAP2L	6	No	Yes	No
LRP1B	6	No	No	No
KLHL23	6	Predicted	Yes	No
HAT1	6	No	Yes	No
CDCA7	6	No	Yes	No
NAB1	6	No	No	No
STK17B	6	No	Yes	No
TMEM237	6	No	No	No
BARD1	6	No	Yes	Yes
USP37	6	No	Yes	No
NHEJ1	6	No	No	No
HJURP	6	No	Yes	No
PASK	6	No	Yes	No
SETMAR	6	No	No	No
RAD18	6	No	Yes	No
SGO1	6	No	Yes	No
ZNF620	6	No	No	No
ABHD5	6	No	No	No
KIF15	6	No	Yes	No
CDC25A	6	Published	Yes	Yes
ATRIP	6	No	No	No
PFKFB4	6	No	No	No
TRAIP	6	No	Yes	No
IL17RB	6	No	No	No

KCTD6	6	No	No	No
NFKBIZ	6	No	No	No
LRRC58	6	No	No	No
POLQ	6	No	Yes	No
OSBPL11	6	No	No	No
MCM2	6	Predicted	Yes	Yes
GATA2	6	Published	No	No
TOPBP1	6	No	Yes	No
DIPK2A	6	No	No	No
ARHGEF26	6	No	No	No
SMC4	6	No	Yes	Yes
B3GNT5	6	No	No	No
RFC4	6	No	Yes	No
RNF168	6	No	No	No
FBXO45	6	No	No	No
ANKRD18DP	6	No	No	No
SLBP	6	No	Yes	Yes
NSD2	6	No	Yes	No
HAUS3	6	No	Yes	No
NCAPG	6	No	Yes	No
RFC1	6	No	Yes	Yes
CEP135	6	No	Yes	No
DCK	6	No	Yes	Yes
PAQR3	6	Predicted	No	No
HADH	6	No	Yes	No
FAM241A	6	No	No	No
TIFA	6	No	No	No
CCNA2	6	Predicted	Yes	No
PLK4	6	No	Yes	Yes
MND1	6	No	Yes	No
C4orf46	6	No	Yes	No
FNIP2	6	No	No	No
NEIL3	6	Predicted	Yes	No
ING2	6	No	No	No
CASP3	6	No	No	No
CENPU	6	No	Yes	No
PDLIM3	6	No	No	No
OTULIN	6	No	No	No
RAD1	6	No	Yes	Yes
NUP155	6	No	Yes	No
C5orf34	6	No	Yes	No
HCN1	6	No	No	No
DEPDC1B	6	No	Yes	No
CENPH	6	No	Yes	No
ARRDC3	6	No	Yes	No
SLF1	6	No	Yes	No
TRIM36	6	No	No	No
KIF20A	6	Predicted	Yes	No
CDC25C	6	No	Yes	No
EGR1	6	No	No	No

SPRY4	6	No	No	No
N4BP3	6	No	No	No
SNRNP48	6	No	No	No
DEK	6	No	Yes	Yes
GMNN	6	Predicted	Yes	No
ZNF165	6	No	No	No
PGBD1	6	No	No	No
HSPA1B	6	No	No	No
KIFC1	6	No	Yes	No
FKBP5	6	Predicted	Yes	No
BRPF3	6	Predicted	No	No
CENPQ	6	No	Yes	No
MCM3	6	Predicted	Yes	Yes
RAB23	6	No	No	No
CASP8AP2	6	No	Yes	No
MMS22L	6	No	Yes	Yes
MYB	6	Published	No	No
HECA	6	No	No	No
EPM2A	6	No	No	No
FBXO5	6	No	Yes	No
ZNRF2	6	No	No	No
LINC00997	6	No	No	No
ANLN	6	No	Yes	No
FIGNL1	6	No	Yes	No
ZNF273	6	No	Yes	No
ZNF92	6	No	Yes	No
RFC2	6	No	Yes	Yes
DBF4	6	No	Yes	No
MCM7	6	No	Yes	Yes
TMEM209	6	No	Yes	No
XRCC2	6	No	Yes	No
NCAPG2	6	No	Yes	No
AGPAT5	6	No	Yes	No
LONRF1	6	No	No	No
EGR3	6	No	No	No
CDCA2	6	No	Yes	No
ESCO2	6	No	Yes	No
PBK	6	No	Yes	No
MCM4	6	No	Yes	Yes
JPH1	6	No	No	No
IMPA1	6	No	No	No
SNX16	6	No	No	No
GEM	6	No	No	No
RAD54B	6	No	Yes	No
CCNE2	6	Published	No	No
DSCC1	6	No	Yes	Yes
AC021945.1	6	No	No	No
ATAD2	6	No	Yes	Yes
HAUS6	6	No	Yes	No
MYORG	6	No	No	No

MELK	6	No	Yes	Yes
AL513165.2	6	No	No	No
BX664727.3	6	No	No	No
CEP78	6	No	Yes	No
RMI1	6	No	Yes	No
ZNF367	6	No	Yes	No
SMC2	6	No	Yes	No
TMEM38B	6	No	No	No
PTPN3	6	No	No	No
ZNF483	6	No	No	No
KIAA1958	6	No	No	No
ZFP37	6	No	No	No
MIR600HG	6	No	No	No
LHX2	6	Predicted	No	No
NUP188	6	No	No	No
TUBB4B	6	No	No	No
NRARP	6	No	No	No
PRKCQ	6	Predicted	No	No
MCM10	6	No	Yes	No
HSPA14_2	6	No	No	No
SUV39H2	6	No	Yes	No
MASTL	6	No	Yes	No
ZWINT	6	No	Yes	No
TFAM	6	No	No	No
DNA2	6	No	Yes	No
DNAJC9	6	No	Yes	No
KIF20B	6	No	Yes	No
KIF11	6	No	Yes	No
HHEX	6	No	No	No
PLCE1	6	No	No	No
HELLS	6	Predicted	Yes	Yes
ARHGAP19	6	No	Yes	No
CHUK	6	No	No	No
PCGF6	6	No	No	No
TAF5	6	Predicted	Yes	No
ITPRIP	6	No	No	No
SMC3	6	No	Yes	Yes
ZDHHC6	6	No	No	No
ZRANB1	6	No	No	No
MKI67	6	No	Yes	Yes
WEE1	6	No	Yes	Yes
NUP160	6	No	Yes	No
SLC43A3	6	No	No	No
FAM111B	6	No	Yes	No
FEN1	6	No	Yes	No
INCENP	6	Predicted	Yes	No
CDCA5	6	No	Yes	No
POLA2	6	No	Yes	Yes
AP000944.5	6	No	No	No
TIGD3	6	No	No	No

PGM2L1	6	No	No	No
POLD3	6	No	Yes	Yes
EMSY	6	No	No	No
EED	6	No	No	Yes
SLC36A4	6	No	No	No
SMCO4	6	No	No	No
CEP295	6	No	Yes	No
NPAT	6	No	Yes	No
CHEK1	6	No	Yes	Yes
PRDM10	6	No	No	No
CCDC77	6	No	Yes	No
FOXM1	6	Published	Yes	No
RAD51AP1	6	No	Yes	Yes
GPR19	6	Predicted	Yes	No
TMEM106C	6	No	No	No
SENP1	6	Predicted	Yes	No
RHEBL1	6	No	No	No
RACGAP1	6	No	Yes	Yes
ESPL1	6	No	Yes	Yes
CDK2	6	No	Yes	No
TIMELESS	6	Predicted	Yes	Yes
PRIM1	6	No	Yes	No
ATP23	6	No	Yes	No
YEATS4	6	No	Yes	No
TBC1D15	6	No	No	No
CSRP2	6	No	No	No
TMPO	6	No	Yes	Yes
UHRF1BP1L	6	No	No	No
UNG	6	No	Yes	Yes
FAM222A	6	No	No	No
RFC5	6	No	Yes	No
KNTC1	6	No	Yes	No
MPHOSPH9	6	No	Yes	No
AC073857.1	6	No	No	No
BRI3BP	6	No	No	No
POLE	6	No	Yes	Yes
SKA3	6	No	Yes	No
CENPJ	6	No	Yes	No
NUP58	6	No	Yes	No
B3GLCT	6	No	No	No
BRCA2	6	No	Yes	Yes
RFC3	6	No	Yes	Yes
DGKH	6	No	No	No
CKAP2	6	No	Yes	No
FBXL3	6	No	No	No
CLYBL	6	No	No	No
TEX30	6	No	Yes	No
ING1	6	No	Yes	No
TUBGCP3	6	Predicted	No	No
CHAMP1	6	No	No	No

PARP2	6	No	Yes	No
LINC00641	6	No	No	No
AL132780.1	6	No	No	No
BAZ1A	6	No	No	No
FANCM	6	No	Yes	No
LRR1	6	No	Yes	No
POLE2	6	No	Yes	No
TXNDC16	6	No	No	No
WDHD1	6	No	Yes	No
HSPA2	6	No	No	No
LIN52	6	No	Yes	No
ACYP1	6	No	No	No
FOS	6	No	No	No
GON7	6	No	No	No
UBR7	6	No	Yes	Yes
XRCC3	6	No	Yes	No
CDCA4	6	No	Yes	No
TUBGCP5	6	No	No	No
BUB1B	6	No	Yes	Yes
KNL1	6	No	Yes	No
RAD51	6	Published	Yes	No
NUSAP1	6	Predicted	Yes	No
HAUS2	6	No	Yes	No
TUBGCP4	6	No	No	No
WDR76	6	Predicted	Yes	No
CTDSPL2	6	No	Yes	No
PCLAF	6	No	Yes	No
PARP16	6	No	No	No
CALML4	6	No	No	No
KIF23	6	No	Yes	No
LRRC49	6	No	No	No
CHRNA5	6	No	No	No
FANCI	6	Predicted	Yes	No
TICRR	6	No	Yes	No
BLM	6	No	Yes	No
PRC1	6	No	Yes	No
AC068831.8	6	No	No	No
CCNF	6	Predicted	Yes	No
EMP2	6	Predicted	No	No
REXO5	6	No	No	No
ZKSCAN2-DT	6	No	No	No
SHCBP1	6	Predicted	Yes	No
ORC6	6	No	Yes	Yes
GINS3	6	Predicted	Yes	Yes
RFWD3	6	No	Yes	No
ZFP1	6	No	No	No
TMEM170A	6	No	No	No
CENPN	6	No	Yes	No
AC092718.4	6	No	No	No
GAN	6	No	No	No

GINS2	6	No	Yes	No
CDT1	6	No	Yes	No
FANCA	6	Predicted	Yes	No
RFLNB	6	No	No	No
SMYD4	6	No	No	No
HASPIN	6	No	Yes	No
USP6	6	No	No	No
MIS12	6	No	Yes	No
WRAP53	6	No	Yes	No
TOP3A	6	No	Yes	No
SPAG5	6	No	Yes	Yes
ATAD5	6	No	Yes	No
SUZ12	6	No	Yes	No
RHBDL3	6	No	No	No
CDK5R1	6	No	No	No
MYO19	6	Predicted	Yes	No
PPP1R1B	6	No	No	No
CDC6	6	No	Yes	No
PSMC3IP	6	No	Yes	Yes
BRCA1	6	No	Yes	Yes
KIF18B	6	No	Yes	Yes
EME1	6	Predicted	Yes	No
TUBD1	6	No	Yes	No
BRIP1	6	No	Yes	No
TCAM1P	6	No	No	No
GNA13	6	No	No	No
SLC25A19	6	No	Yes	No
CBX2	6	No	No	No
YES1	6	No	No	No
METTL4	6	No	Yes	No
NDC80	6	No	Yes	No
RBBP8	6	No	No	No
SKA1	6	Predicted	Yes	No
C18orf54	6	Predicted	Yes	No
ONECUT2	6	No	No	No
BCL2	6	Published	No	No
EFNA2	6	No	No	No
ZNF555	6	No	No	No
CHAF1A	6	No	Yes	No
UHRF1	6	Predicted	Yes	No
ZNF266	6	No	No	No
ZNF562	6	No	No	No
DNMT1	6	No	Yes	Yes
KANK2	6	Predicted	No	No
ZNF887P	6	No	No	No
ZNF823	6	No	No	No
ZNF700	6	No	No	No
ZNF136	6	No	No	No
ZNF44	6	No	No	No
ZNF799	6	No	No	No

ZNF443	6	No	No	No
RNASEH2A	6	No	Yes	Yes
IL27RA	6	No	No	No
SAMD1	6	No	No	No
ASF1B	6	No	Yes	Yes
HAUS8	6	No	Yes	No
UNC13A	6	No	No	No
UBA2	6	No	No	No
WDR62	6	No	Yes	No
ZNF45	6	No	Yes	No
ZNF230	6	No	No	No
AC021092.2	6	No	No	No
RNF24	6	No	No	No
MCM8	6	No	Yes	No
GINS1	6	Predicted	Yes	Yes
AL035071.1	6	No	No	No
E2F1	6	Published	Yes	No
DSN1	6	Predicted	Yes	No
RBL1	6	No	Yes	No
MYBL2	6	Published	Yes	Yes
CSE1L	6	No	Yes	Yes
SNAI1	6	Published	No	No
CABLES2	6	No	No	No
AL121832.3	6	No	No	No
SIK1B	6	No	No	No
CU638689.4	6	No	No	No
AP000295.1	6	No	No	No
DONSON	6	No	No	Yes
CHAF1B	6	No	No	No
ZBTB21	6	No	No	No
PKNOX1	6	Predicted	Yes	No
CDC45	6	No	Yes	No
CHEK2	6	No	Yes	Yes
CCDC117	6	No	No	No
YWHAH	6	No	No	No
CENPM	6	No	Yes	Yes
RIBC2	6	No	No	No
GTSE1	6	Predicted	Yes	No
FANCB	6	No	Yes	No
PIGA	6	No	Yes	No
SCML1	6	No	No	No
SCML2	6	Predicted	No	No
KLHL15	6	No	No	No
SUV39H1	6	No	Yes	Yes
SMC1A	6	No	Yes	Yes
SPIN4	6	No	No	No
ERCC6L	6	No	Yes	No
TAF9B	6	No	No	No
GABRQ	6	No	No	No
AL590822.3	7	No	No	No

EPHA10	7	No	No	No
FGGY	7	No	No	No
PDE4B	7	Predicted	No	No
ST6GALNAC3	7	No	No	No
SAMD13	7	No	No	No
AP4B1-AS1	7	No	No	No
S100A5	7	No	No	No
DPM3	7	No	No	No
AL590560.2	7	No	No	No
PBX1	7	No	No	No
HMCN1	7	No	No	No
AC108488.2	7	No	No	No
EIF3FP3	7	No	No	No
PAIP2B	7	No	No	No
KCNIP3	7	No	No	No
LONRF2	7	No	No	No
NR4A2	7	Published	No	No
PDE11A_1	7	No	No	No
KLF7	7	Predicted	No	No
IL17RD	7	No	No	No
MAGI1	7	No	No	No
AC046134.2	7	No	No	No
SLC9A9	7	No	No	No
TLR6	7	No	No	No
FRAS1	7	No	No	No
GPRIN3	7	No	No	No
CCSER1	7	No	No	No
PDE5A	7	No	No	No
SLC7A11	7	No	No	No
NR3C2	7	No	No	No
TRIM2	7	No	No	No
CTSO	7	No	No	No
PDGFC	7	No	No	No
GUSBP1	7	No	No	No
SELENOP	7	No	No	No
ADAMTS6	7	No	No	No
IQGAP2	7	No	No	No
ZBED3-AS1	7	No	No	No
ATP6AP1L	7	No	No	No
GLRX	7	No	No	No
STARD4-AS1	7	No	No	No
NREP	7	No	No	No
ZNF608	7	No	No	No
SYNPO	7	Predicted	No	No
ADAMTS2	7	No	No	No
GMDS-DT	7	No	No	No
SAPCD1	7	No	No	No
HLA-DMB	7	No	No	No
RCAN2	7	No	No	No
AL080250.1	7	No	No	No

RPS6KA2	7	No	No	No
PRKAR1B	7	No	No	No
GPER1	7	No	No	No
HOXA4	7	Predicted	No	No
SEMA3A	7	No	No	No
SEMA3D	7	No	No	No
PPP1R9A	7	No	No	No
TECPR1	7	No	No	No
UFSP1	7	No	No	No
WNT16	7	No	No	No
FAM180A	7	No	No	No
ZNF467	7	No	No	No
NUDT18	7	No	No	No
HAS2	7	No	No	No
AL354707.1	7	No	No	No
FAM201A	7	No	No	No
FRG1JP	7	No	No	No
AL161457.2	7	No	No	No
SHC3	7	No	No	No
SARDH	7	No	No	No
LCN12	7	No	No	No
AL136982.1	7	No	No	No
KAZALD1	7	No	No	No
SLC22A18	7	No	No	No
OR51B4	7	No	No	No
CAVIN3	7	No	No	No
ACCS	7	No	No	No
SLC43A1	7	No	No	No
ARHGAP42	7	No	No	No
KBTBD3	7	No	No	No
SPSB2	7	No	No	No
BHLHE41	7	No	No	No
FAR2	7	No	No	No
AC008014.1	7	No	No	No
METTL7A	7	No	No	No
TNS2	7	No	No	No
METTL7B	7	No	No	No
TMEM198B	7	No	No	No
CAPS2	7	No	No	No
RPS2P5	7	No	No	No
AC004812.2	7	No	No	No
LINC01089	7	No	No	No
AC084018.2	7	No	No	No
STARD13	7	No	No	No
LINC02331	7	No	No	No
BMP4	7	No	No	No
SERPINA5	7	No	No	No
DICER1-AS1	7	No	No	No
PLCB2	7	No	No	No
AC090517.4	7	No	No	No

AC009065.3	7	No	No	No
LDHD	7	No	No	No
CDH15	7	No	No	No
VPS9D1	7	No	No	No
TLCD2	7	No	No	No
SERPINF2	7	No	No	No
SREBF1	7	No	No	No
AC073508.2	7	No	No	No
NAGLU	7	No	No	No
FZD2	7	No	No	No
ACE	7	No	No	No
MILR1	7	No	No	No
CEP112	7	No	No	No
ARSG	7	No	No	No
SLC16A6	7	No	No	No
LINC00482	7	No	No	No
FGF22	7	No	No	No
TLE6	7	No	No	No
NDUFB7	7	No	No	No
KCNN1	7	No	No	No
AC020910.6	7	No	No	No
ETV2	7	No	No	No
RABAC1	7	No	No	No
LRRC4B	7	No	No	No
SPACA6	7	No	No	No
NDUFV2P1	7	No	No	No
BFSP1	7	No	No	No
CYP24A1	7	No	No	No
LSS	7	Predicted	No	No
LINC00896	7	No	No	No
SCUBE1	7	No	No	No
ODF3B	7	No	No	No
CA5B	7	No	No	No
FAAH2	7	No	No	No
AMOT	7	No	No	No
MBNL3	7	No	No	No
DNASE1L1	7	No	No	No
CDA	8	Predicted	No	No
EXTL1	8	No	No	No
SPOCD1	8	No	No	No
TMEM234	8	No	No	No
TMEM125	8	No	No	No
SLC6A9	8	Predicted	No	No
BEST4	8	No	No	No
AL604028.1	8	No	No	No
TMEM61	8	No	No	No
GSTM2	8	No	No	No
BCL2L15	8	No	No	No
PEX11B	8	No	No	No
PDIA3P1	8	No	No	No

MINDY1	8	No	No	No
TRIM46	8	No	No	No
THBS3	8	No	No	No
AL592435.1	8	No	No	No
AL645568.1	8	No	No	No
CAPN8	8	No	No	No
ZNF670-ZNF695	8	No	No	No
INO80B	8	No	No	No
AC022210.2	8	No	No	No
CYTOR	8	No	No	No
LINC01123	8	No	No	No
ZBTB45P2	8	No	No	No
AC105402.3	8	No	No	No
AC012443.2	8	No	No	No
TTC30B	8	No	No	No
ALPP	8	No	No	No
EFHD1	8	No	No	No
LMCD1-AS1	8	No	No	No
PRRT3	8	No	No	No
CMTM7	8	No	No	No
FBXL2	8	No	No	No
TTC21A	8	No	No	No
PRKAR2A-AS1	8	No	No	No
HEMK1	8	No	No	No
AC097358.2	8	No	No	No
TMCC1-AS1	8	No	No	No
NUDT16P1	8	No	No	No
NME9	8	No	No	No
ERICH6-AS1	8	No	No	No
VEPH1	8	No	No	No
TMEM44-AS1	8	No	No	No
CFAP99	8	No	No	No
FGFBP2	8	No	No	No
UBA6-AS1	8	No	No	No
ANK2	8	No	No	No
LINC02236	8	No	No	No
AC008957.3	8	No	No	No
LINC02065	8	No	No	No
AC022107.1	8	No	No	No
LINC02163	8	No	No	No
MIR3936HG	8	No	No	No
ATOX1	8	No	No	No
STC2	8	No	No	No
RGS14	8	No	No	No
DDAH2	8	No	No	No
PRRT1	8	No	No	No
AL451165.2	8	No	No	No
PLA2G7	8	No	No	No
GSTA2	8	No	No	No
CITED2	8	Predicted	No	No

UTRN	8	No	No	No
C6orf99	8	No	No	No
AGPAT4	8	No	No	No
AL596442.3	8	No	No	No
AC004982.2	8	No	No	No
HOXA1	8	No	No	No
NPC1L1	8	No	No	No
PSPH	8	No	No	No
ZNF117	8	No	No	No
SAMD9	8	No	No	No
AC254629.1	8	No	No	No
CDHR3	8	No	No	No
AC002066.1	8	No	No	No
LINC00513	8	No	No	No
FMC1	8	No	No	No
WEE2-AS1	8	No	No	No
CLCN1	8	No	No	No
NOS3	8	Predicted	No	No
CDK5	8	No	No	No
AC021218.1	8	No	No	No
AC022784.1	8	No	No	No
PIWIL2	8	No	No	No
SCARA5	8	No	No	No
SLC20A2	8	No	No	No
AP002851.1	8	No	No	No
SAMD12	8	No	No	No
ERICD	8	No	No	No
AC067930.8	8	No	No	No
AC109322.1	8	No	No	No
AC233992.2	8	No	No	No
LRRC24	8	No	No	No
SPATA6L	8	No	No	No
LURAP1L	8	No	No	No
TLE1	8	No	No	No
PTCH1	8	Predicted	No	No
OLFML2A	8	No	No	No
PLPP7	8	No	No	No
SKIDA1	8	No	No	No
ITGB1-DT	8	No	No	No
RASSF4	8	No	No	No
AL356056.2	8	No	No	No
BEND3P1	8	No	No	No
ZNF503-AS1	8	No	No	No
IFIT1	8	No	No	No
PDZD7	8	No	No	No
AFAP1L2	8	No	No	No
LRRC56	8	No	No	No
LMNTD2	8	No	No	No
GATD1	8	No	No	No
CARS1	8	No	No	No

OR51B3P	8	No	No	No
AP001453.4	8	No	No	No
ZNHIT2	8	No	No	No
POLD4	8	No	No	No
LINC02701	8	No	No	No
LINC02700	8	No	No	No
LINC02732	8	No	No	No
TMEM25	8	Predicted	No	No
C11orf45	8	No	No	No
CACNA2D4	8	No	No	No
SCNN1A	8	No	No	No
ING4	8	No	No	No
FAM90A1	8	No	No	No
ARHGDIB	8	Published	No	No
PLEKHA8P1	8	No	No	No
AC021066.1	8	No	No	No
KRT83	8	No	No	No
ITGA7	8	No	No	No
AC009779.3	8	No	No	No
NDUFA4L2	8	No	No	No
SLC26A10	8	No	No	No
PTPRR	8	No	No	No
AC078923.1	8	No	No	No
AC078820.3	8	No	No	No
NAV3	8	Predicted	No	No
TRPV4	8	No	No	No
HRK	8	No	No	No
AC131009.3	8	No	No	No
AC148477.3	8	No	No	No
AC148477.2	8	No	No	No
TPTE2P1	8	No	No	No
UBAC2-AS1	8	No	No	No
LINC00346	8	No	No	No
NFATC4	8	Published	No	No
ATL1	8	No	No	No
JDP2	8	No	No	No
FOXN3-AS1	8	No	No	No
RPS3AP6	8	No	No	No
ANXA2	8	No	No	No
SMAD6	8	No	No	No
NOX5	8	No	No	No
GDPGP1	8	No	No	No
RPL3L	8	No	No	No
ELOB	8	No	No	No
AC108134.1	8	No	No	No
AC126755.7	8	No	No	No
AC008915.3	8	No	No	No
AC106782.1	8	No	No	No
CES3	8	No	No	No
AC027682.6	8	No	No	No

PKD1L2	8	No	No	No
MLYCD	8	No	No	No
SLC7A5	8	No	No	No
TNFSF12	8	No	No	No
TRIM16	8	No	No	No
AC104024.4	8	No	No	No
MYO15A	8	No	No	No
AC004477.1	8	No	No	No
TOB1-AS1	8	No	No	No
AC007114.1	8	No	No	No
SOX9-AS1	8	No	No	No
TTYH2	8	No	No	No
MGAT5B	8	No	No	No
TNRC6C-AS1	8	No	No	No
TMC8	8	No	No	No
C1QTNF1	8	No	No	No
PCYT2	8	No	No	No
AC132872.5	8	No	No	No
LINC01910	8	No	No	No
ZNF516	8	No	No	No
AC018413.1	8	No	No	No
AC005775.1	8	No	No	No
AC005256.1	8	No	No	No
IZUMO4	8	No	No	No
FXYD1	8	No	No	No
AC002398.1	8	No	No	No
LINC01534	8	No	No	No
SNHG11	8	No	No	No
PTK6	8	No	No	No
B3GALT5	8	No	No	No
TMEM191B	8	No	No	No
AIFM3	8	No	No	No
SLC2A11	8	No	No	No
RHBDD3	8	No	No	No
LINC01521	8	No	No	No
TMPRSS6	8	No	No	No
EFCAB6	8	No	No	No
AC207130.1	8	No	No	No
CSF2RA	8	No	No	No
CLCN4	8	No	No	No
SH3KBP1	8	No	No	No
YY2	8	No	No	No
SLC35A2	8	No	No	No
